# Differentiated Neuroprogenitor Cells Incubated with Human or Canine Adenovirus, or Lentiviral Vectors Have Distinct Transcriptome Profiles

**DOI:** 10.1371/journal.pone.0069808

**Published:** 2013-07-26

**Authors:** Stefania Piersanti, Letizia Astrologo, Valerio Licursi, Rossella Costa, Enrica Roncaglia, Aurelie Gennetier, Sandy Ibanes, Miguel Chillon, Rodolfo Negri, Enrico Tagliafico, Eric J. Kremer, Isabella Saggio

**Affiliations:** 1 Dipartimento di Biologia e Biotecnologie “Chrales Darwin”, Sapienza, Università di Roma, Roma, Italy; 2 Dipartimento di Scienze Biomediche, Università degli Studi di Modena e Reggio Emilia, Modena, Italy; 3 Institut de Génétique Moléculaire de Montpellier, University Montpellier I & II, Montpellier, France; 4 Institució Catalana de Recerca i Estudis Avançats (ICREA), Biochemistry and Molecular Biology Department, Centre de Biotecnologia Animal i Teràpia Gènica (CBATEG) Universitat Autònoma de Barcelona, Bellaterra, Spain; 5 Istituto Pasteur Fondazione Cenci Bolognetti, Roma, Italy; 6 Istituto di Biologia e Patologia Molecolari del CNR, Roma, Italy; University of Chicago, United States of America

## Abstract

Several studies have demonstrated the potential for vector-mediated gene transfer to the brain. Helper-dependent (HD) human (HAd) and canine (CAV-2) adenovirus, and VSV-G-pseudotyped self-inactivating HIV-1 vectors (LV) effectively transduce human brain cells and their toxicity has been partly analysed. However, their effect on the brain homeostasis is far from fully defined, especially because of the complexity of the central nervous system (CNS). With the goal of dissecting the toxicogenomic signatures of the three vectors for human neurons, we transduced a *bona fide* human neuronal system with HD-HAd, HD-CAV-2 and LV. We analysed the transcriptional response of more than 47,000 transcripts using gene chips. Chip data showed that HD-CAV-2 and LV vectors activated the innate arm of the immune response, including Toll-like receptors and hyaluronan circuits. LV vector also induced an IFN response. Moreover, HD-CAV-2 and LV vectors affected DNA damage pathways - but in opposite directions - suggesting a differential response of the p53 and ATM pathways to the vector genomes. As a general response to the vectors, human neurons activated pro-survival genes and neuron morphogenesis, presumably with the goal of re-establishing homeostasis. These data are complementary to in *vivo* studies on brain vector toxicity and allow a better understanding of the impact of viral vectors on human neurons, and mechanistic approaches to improve the therapeutic impact of brain-directed gene transfer.

## Introduction

Gene transfer in the central nervous system (CNS) is particularly challenging due to the post-mitotic nature of neuronal cells, the sensitivity of these cells to injury, and the highly complex nature of CNS. Given their ability to enter terminally differentiated cells, and to exploit axonal transport, a handful of virus-derived vectors have been tested for brain gene therapy and for the study of brain-related functions ([1] and references therein). Each viral vector has its specific advantages and drawbacks such as cloning capacity, memory or induced immunity, specificity, safety, titer, or efficacy, and there is no single gene transfer vector that can be used to treat all brain diseases. In general terms, however, for clinical applications it is important to identify vector candidates with the best efficacy versus toxicity ratio. HAd vectors, derived from serotype 5, preferentially transduce glia after inoculation of various brain areas of adult rodents [2], dogs and nonhuman primates [3], [4]. Although the clinical use of HD-HAd vectors in the CNS may, under some conditions, be restricted by an innate immune response, there are scenarios where they may be the best tools available [1]. An alternative to HD-HAd vectors are those derived from CAV-2, which share the ∼30 kb cloning capacity and ability for long-term (≥1 year) transgene expression. CAV-2 vectors preferentially transduce neurons in human organotypic cortical slices, in rodents, dogs and nonhuman primate CNS [5], [6]. In addition, CAV-2 vectors are capable of long-range bidirectional motility in the neuron [7], humans do not harbour significant titers of anti-CAV-2 neutralizing antibodies [8] and CAV-2 vectors appear to be poorly immunogenic in the CNS of most animals [9]. Taken together, HD-CAV-2 vectors may be particularly relevant for therapy of some brain diseases. However, a better understanding of their effect on human neurons is a prerequisite for their clinical use.

HIV-1-derived vectors also lead to efficient transduction of post-mitotic cells (including neurons) and long-term expression [10]. After delivery into the CNS of rodents, HIV vectors can induce a modest immune response [11], and in a single human trial a nonhuman LV has been used for injection into the striatum in Parkinson patients with a global safety profile [12]. Yet, in a systemic gene delivery method LV vectors activated the IFN αβ pathway [13], and vesicular stomatitis virus glycoprotein G (VSV-G) pseudotyped LV vectors activated Toll-like receptor (TLR) pathways [14]. In addition, although several studies have characterized the insertional mutagenesis risks related to the use of HIV-1 derived vectors [15], the downstream effects of the LV integration process have not been clarified [16].

An approach to dissect the biological pathways linked to vector interaction is to perform a genome wide transcriptome analysis. This approach is contributing to the understanding of vector safety and biology ([17] and references therein). In this study we generated comparative and cell-specific information on the effect of HD-HAd, HD-CAV-2 and LV on human brain cells. We incubated cultures of differentiated human midbrain neuroprogenitor cells (hmNPCs) with the vectors. hmNPCs acquired morphological and functional properties of neurons, with 15–20% having the hallmarks of dopaminergic (DA) neurons. These cells are a powerful prototype for human CNS therapy and neuronal disease modelling ([18] and references therein). The transcriptome analysis of the HD-HAd-, HD-CAV-2- and LV-transduced hmNPCs led to a better understanding of the biology of the vectors, their impact on the intracellular trafficking, cell remodelling pathways, the immune response, and, more globally, their toxicogenomic signature for brain gene therapy.

## Materials and Methods

### Cells

hmNPCs were isolated from embryonic midbrain tissue under compliance with the German Arztekammer government and NECTAR guidelines. For expansion, the cells were cultured according to previously described protocols [18]–[20]. Cells were cultured in coated flasks with a serum-free medium consisting in DMEM (high glucose)/F-12 mixture (1∶1) (Invitrogen, Carlsbad, CA), supplemented with 2% B27 (Invitrogen), 20 ng/mL human epidermal growth factor (EGF), 20 ng/ml human fibroblast growth factor-2 (FGF-2; all supplements from Peprotech, Rocky Hill, NJ), 10 µg/mL Gentamycin (Invitrogen), 1 µg/mL Tocopherole (Sigma-Aldrich, St. Louis, MO, USA) and 1 µg/mL Tocopherole acetate (Sigma). Cultures were placed in a humidified incubator at 37°C, 5% CO_2_ and 3% O_2_. For DA differentiation, cells were incubated with Neurobasal medium (Invitrogen) supplemented with 2% B27, 2 mM Glutamax (Invitrogen), 10 µM Forskolin (Calbiochem, EMD Biosciences, Darmstadt, Germany), 100 µM dibutiryl c-AMP (Sigma), and 10 µg/ml Gentamycin (Invitrogen), at 37°C, 5% CO_2_ and 3% O_2_. 293T (ATCC CRL-11268) and 293Cre (provided by Merck & Co., Westpoint, PA) were maintained in DMEM (Invitrogen), 10% FBS, 100 U/ml penicillin, and 100 µg/ml streptomycin. 293Cre were supplemented with 0.4 mg/mL G418 (Sigma).

### Vectors

HD-HAd was produced as described in [21]. LV and LV GFP(-), devoid of GFP, were prepared by combined transfection of 293T cells with the following plasmids: pRRLSIN.cPPT.PGK-GFP.WPRE, pMDLg/pRRE, pRSV-Rev and pMD2G (all from Addgene, http://www.addgene.org/) for LV and pLKO.1 puro control vector (Sigma), pR8.74 and pMD2G for LV GFP(-). Cell supernatants were collected at 48 and 72 h and successively purified as described in [22]. HD-CAV-2 was produced as in [6]. The titers of HD-HAd, LV, LV GFP(-) and HD-CAV-2 were determined by qPCR on vector genomes. We used the following primers for GFP amplification: GFP1 For 5′- CAACAGCCACAACGTCTATATCATG -3′, GFP1 Rev 5′- ATGTTGTGGCGGATCTTGAAG -3′, GFP2 For 5′-GCCGACCATTATCAACAGAACA-3′, and GFP2 Rev 5′-TGGTTGTCTGGGAGGAGCAC-3′; and the following primer pairs for puromycin amplification: Puro For 5′-CACCGAGCTGCAAGAACTCTT-3′ and Puro Rev 5′-CCCACACCTTGCCGATGT-3′. A reference curve was generated by amplifying serial dilutions of each vector plasmid using GFP and Puromycin primer sets (R^2^ = 0.99). qPCRs were performed using the Applied Biosystems 7300 Real Time PCR system. The MOI is expressed as vector genomes per cell.

### Transduction of differentiated human neuronal progenitors cells

hmNPCs were cultured in 25 or 12.5 cm^2^ flasks (Nunc, Roskilde, Denmark), coated with 100 µg/mL poly- L- ornithine (Sigma) and 1 mg/mL human fibronectin (Millipore, Billerica, MA, USA). Following treatment with DA differentiation medium, cells were transduced 2 h with the different vectors at the indicated MOIs and then washed twice with PBS. GFP expression was evaluated by FACS analysis (FACS Calibur, Becton Dickinson). At the indicated time postinfection cells were collected and RNA was isolated by using the RNeasy Mini Kit (Qiagen, Valencia, CA, USA) following manufacturer's recommendations. Total RNA was treated with DNA-se (Qiagen) and reverse transcribed using the Super Script III First Strand synthesis system (Invitrogen). For intracellular vector genomes quantification, cells were transduced at an MOI of 1000 vector genomes/cell and harvested after 2 h, 5 days and 10 days. Total DNA was extracted with the DNeasy kit (Qiagen). Vector DNA copy number was calculated by qPCR using GFP primers (GFP1 and GFP2 For/Rev couples), and beta-actin primers (BACT For 5′-CGGCATCGTCACCAACTG-3′ and BACT Rev 5′-GGCACACGCAGCTCATTG-3′) to normalize for genomic DNA copy number. qPCRs were performed fivefold using the Applied Biosystems 7300 Real Time PCR system and SYBR®Green PCR master Mix. Data are expressed as ratio to the genomic copies of beta-actin.

### Gene chip and data analysis

Total RNA extracted from transduced cells at 2 h and 5 days postinfection was tested on disposable RNA chips (Agilent RNA 6000 Nano LabChip kit) to determine the concentration and purity/integrity of RNA samples using Agilent 2100 bioanalyser. cDNA synthesis, biotin-labeled target synthesis, hybridization to HG-U133 plus 2.0 GeneChip (Affymetrix) arrays, staining and scanning were performed according to the standard protocol supplied by Affymetrix. For each probe set on each array, a detection call of Present, Absent or Marginal was made. Detection calls were made using the *affy* R/Bioconductor package [23]. Background corrected raw data were Log2-transformed and quantile-normalized following the Robust Multichip Average (RMA) procedure using R (Bioconductor) [24]. Differentially expressed genes were obtained with *limma* package, performing paired pair wise comparison between the mock and vector transduced hmNPCs and picking up probe sets showing a present call and a fold change of at least ±1.5 in all the replicates. A paired t-test was performed between transduced and untreated groups selecting genes with a p-value≤0.05. The data set containing the Affymetrix probe identifiers, selected as differentially expressed in transduced hmNPCs, and the corresponding fold changes, were uploaded into Ingenuity Pathways Analysis (www.ingenuity.com). Each Affymetrix probe identifier was mapped to its corresponding gene in the Ingenuity Pathways Knowledge Base. Significant Molecular and Physiological functions were determined querying the Ingenuity Pathways Knowledge Base and a score was computed for each group selecting a scoring method based on the Fisher's exact test used to calculate the p-value. Heat maps of differentially expressed genes and belonging to selected enriched molecular and physiological functions were constructed by using Excel 2007 (Microsoft Office package). Genes were categorized based on the annotations on gProfiler [25] and on described functions in the literature. Genes covered by multiple probe sets on the array were represented by the lowest p-value. The entire microarray data set was submitted to the Gene Expression Omnibus repository with the accession number GSE47130.

### qPCR quantification of gene expression

cDNAs from mock and transduced hmNPCs were used for validation of selected genes by qPCR. mRNA expression was measured by TaqMan (Universal PCR Master Mix, Applied Biosystems), using the following TaqMan® Gene Expression Assays (Applied Biosystems): FANCD2, batch ID Hs00945455_g1, BIRC5, batch ID Hs04194392_s1, MAD2L1, batch ID Hs01554514_g1, RAD51, batch ID Hs00947969_s1, NBN, batch ID Hs00159537_m1, HIP1B, batch ID Hs01034862_m1, MYO6, batch ID Hs01568216_m1, CLTC, batch ID Hs00191535_m1, CD44, batch ID Hs01075862_m1, TLR3, batch ID Hs01551078_m1, TLR4, batch ID Hs00152939_m1, HAS3, batch ID Hs00193436_m1, FXN , batch Hs00175940_m1. In addition, we used the following primers pairs and SYBR green analysis, for p53 For 5′-GCGTGAGCGCTTCGAGAT-3′ and Rev 5′-AGCCTGGGCATCCTTGAGT-3′, for CDKN1A For 5′-TGGAGACTCTCAGGGTCGAAA-3′ and Rev 5′-GGCGTTTGGAGTGGTAGAAATC-3′, for GAPDH For 5′-TGGGCTACACTGAGCACCAG-3′ and Rev 5′-GGGTGTCGCTGTTGAAGTCA-3′. GFAP, TUJ-1 and TH expressions were analysed with the TaqMan probes Hs00909233_m1, Hs00801390_s1 and Hs00165941_m1, respectively. Sample normalization was carried out on the basis of the GAPDH or RPL22 expression (Applied Biosystems, TaqMan® Gene Expression Assay, batch IDs Hs99999905_m1 and Hs01865331_s1, respectively). GAPDH and RPL22 were unchanged in transduced as compared to mock on all chip samples, and comparable results were obtained by qPCR when both normalizers were used in the same analysis. Reactions were performed using the Applied Biosystems PRISM 7300 Real Time PCR System. To obtain relative quantification with respect to the undifferentiated mock cells, quantification cycle values (Cq, [16]) were exported directly into an EXCEL worksheet for analysis, and the data were calculated with the 2^−ΔΔCq^ method [26].

### Statistical analyses of qPCR data

qPCR data are reported as means ± standard deviation (SD) of triplicates or more data obtained from at least three independent experiments. Data were analysed using two-tailed Student's t test or one-way ANOVA followed by Tukey's post- hoc test for pair-wise comparison using GraphPad Prism 5 software. A p-value≤0.05 was considered significant.

## Results

### Human neurons

**Figure 1:**
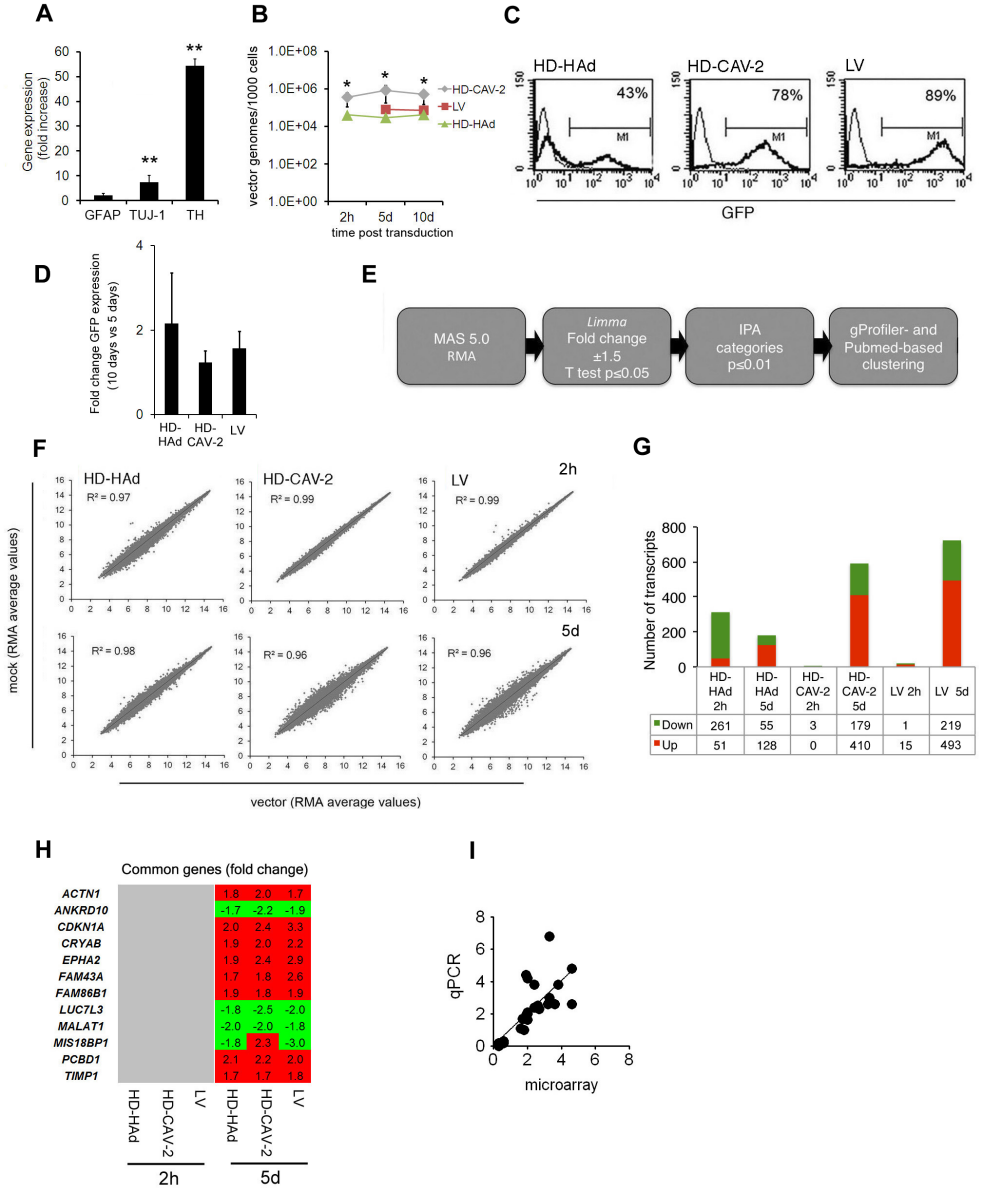
hmNPCs differentiation and transduction. (**a**) qPCR quantification of the markers GFAP, TUJ-1 and TH in differentiated hmNPCs presented as fold changes relative to undifferentiated cells. Values are averages from two independent experiments and SD is shown. p-values are determined by a two-tailed paired Student's t test from the ΔCq values of differentiated with respect to the undifferentiated condition; ** p<0.01. (**b**) qPCR quantification of internalized vector copies performed on differentiated hmNPCs transduced with HD-HAd, HD-CAV-2 and LV vectors at an MOI of 1000 vector genomes/cell. Results are presented as average of two independent experiments; SD is shown. Data were compared by one-way ANOVA followed by Tukey's post- hoc test between vectors for each time point; * p<0.05. (**c–d**) Differentiated hmNPCs were transduced with HD-HAd, HD-CAV-2 and LV at an MOI of 1000 vector genomes/cell and were analysed for GFP expression by FACS at 5 days (c), and by qPCR at 5 and 10 days (d). FACS results are representative of three independent experiments. qPCR data are the average of triplicate samples and are presented as fold change values of 10 days values versus 5 days. SD is shown. (**e**) Schematic representation of chip analysis workflow. (**f**) Scatter plots showing on the y axis the Robust Multichip Average (RMA) expression values for mock cells, and in x RMA values for of HD-HAd, HD-CAV-2 and LV transduced samples, at the 2 h and 5 days posttransduction time points. To obtain RMA, background corrected raw intensity values were Log2 transformed and quantile normalized. All absent and control probes were excluded from this analysis. Values represent the average of the three independent replica experiments. Trend lines and R^2^ value are indicated. (**g**) Number of genes upregulated and downregulated with a fold change ≤−1.5 or ≥+1.5 in response to HD-HAd, HD-CAV-2 and LV at 2 h and 5 days. Red: upregulated genes; green: downregulated genes. (**h**) Heat maps of genes commonly modulated by HD-HAd, HD-CAV-2 and LV and at 2 h and 5 days posttransduction. The relative fold change values are indicated; in red, upregulated genes, in green, downregulated, and in grey, genes with unmodified expression with respect to mock. (**i**) Correlation between relative fold changes measured by microarray and qPCR. Each point represents the fold change of a single gene relative to the mock at a given time point; x and y axes are microarray and qPCR fold changes, respectively. Number of xy pairs = 28. Pearson's *r* correlation = 0.75; p<0.0001 (2-tailed). All samples were tested in triplicate and data are reported as mean value. Tested genes were the following: *FANCD2*, *BIRC5*, *MAD2L1*, *RAD51*, *NBN*, *HIP1B*, *MYO6*, *CLTC*, *CD44*, *TLR3*,*TLR4*, *HAS3*, *FXN*, *p53*, *CDKN1A.*

To assess whether differentiated hmNPCs acquired a neuron phenotype, we analysed the expression of three markers: tyrosine hydroxylase (TH), a marker predictive of dopaminergic (DA) neurons, β III tubulin (TUJ-1), a neuron-specific marker, and glial fibrillary acidic protein (GFAP), a marker of neuroprogenitors and astrocytes. The transcription of all markers was increased post-differentiation, with TH transcript exhibiting the most prominent upregulation (>50 fold) (**Fig. 1a**). Differentiated hmNPCs were incubated with HD-HAd, HD-CAV-2, LV and adeno-associated virus (AAV) 2/9 with the goal of quantifying the effects of the vectors on the transcriptome as in a pharmacological drug test. When setting up the transduction protocol we prioritized the vector dose giving, for the three vectors, the best transduction/dose ratio, which corresponded to an MOI of 1000 vector genomes/cell (not shown). Under these conditions, AAV2/9, which has been suggested for brain gene therapy [27], transduced differentiated hmNPCs with poor efficiency (∼4%, data not shown), and therefore was unsuitable for comparative microarray analysis. At 2 h, HD-CAV-2 and HD-HAd genomes were present in transduced cells while LV DNA was not yet detectable (**Fig. 1b**), which is concordant with the levels of LV retrotranscription at this time [28]. At 5 and 10 days, vector genomes were detected in all cells, and were stable over time (**Fig. 1b**). At day 5 GFP expression was robust for the three vectors, and remained at comparable levels until day 10 (**Fig. 1c,d**). Together these data showed that differentiated hmNPCs activated the hallmarks of DA neurons and, to a minor extent, of glial cells, consistent with the applied differentiation protocol. The results also showed that, although with different profiles, differentiated hmNPCs can be effectively transduced with HD-HAd, HD-CAV-2 and LV at an MOI of 1000.

### Global analysis of HD-HAd, HD-CAV-2 and LV induced transcriptome alterations

To assay early and late processes related to viral entry and cell rearrangements following completed internalization and transduction, we profiled gene expression of HD-HAd-, HD-CAV-2- and LV-transduced cells at 2 h and 5 days post-vector treatment. Indeed, we believe that this bi-phase temporal response would be informative to distinguish between an acute response, mainly triggered by vector entry signalling and chronic or delayed effect, mostly generated by the presence of vector genomes within the cells. Transductions were performed with two independent viral batches/vectors on three independent human cell batches and RNAs were hybridized to Affymetrix HG U133 plus 2 arrays (for a total number of 24 chips). Raw results were submitted to serial analyses according to the workflow depicted on **Fig. 1e**. The three vectors modulated the transcriptome at each time in different ways (**Fig. 1f**). At 2 h, differentiated hmNPCs sensed HD-HAd more than the other vectors (R^2^ 0.97). At 5 days, the bulk of HD-CAV-2 and LV induced modulation was detected (R^2^ 0.962 and 0.961, respectively). HD-HAd significantly modulated 312 transcripts at 2 h (261 down, 51 up), and 183 at 5 days (55 down, 128 up). HD-CAV-2 altered 3 transcripts at 2 h (all down) and 589 at 5 days (179 down, 410 up). In LV samples, 16 transcripts were altered at 2 h (1 down, 15 up), and 712 at 5 days (219 down, 493 up) (**Fig. 1g** **, Table S1 in FileS1**). Few genes were commonly modulated by the three vectors: none at 2 h, and 12 at 5 days (**Fig. 1h**). Twenty-eight independent transcripts, corresponding to 21 upregulated and 7 downregulated genes in the microarray analysis, were then tested by qPCR for validation. 26 out of the 28 tested genes were qualitatively modulated as on chips. The extent of the modulation was quantitatively similar (i.e. ratio between chip and qPCR values ranging from 0.5 to 2fold) for 78.6% of the tested genes, with a Pearson's correlation coefficient between microarray and qPCR values of 0.75 (p-value<1.0E-04) (**Fig. 1i**). These data indicated that HD-HAd, HD-CAV-2 and LV induced significant change in gene expression of differentiated hmNPCs.

### Biological signatures of HD-HAd, HD-CAV-2 and LV in differentiated hmNPCs

**Figure 2:**
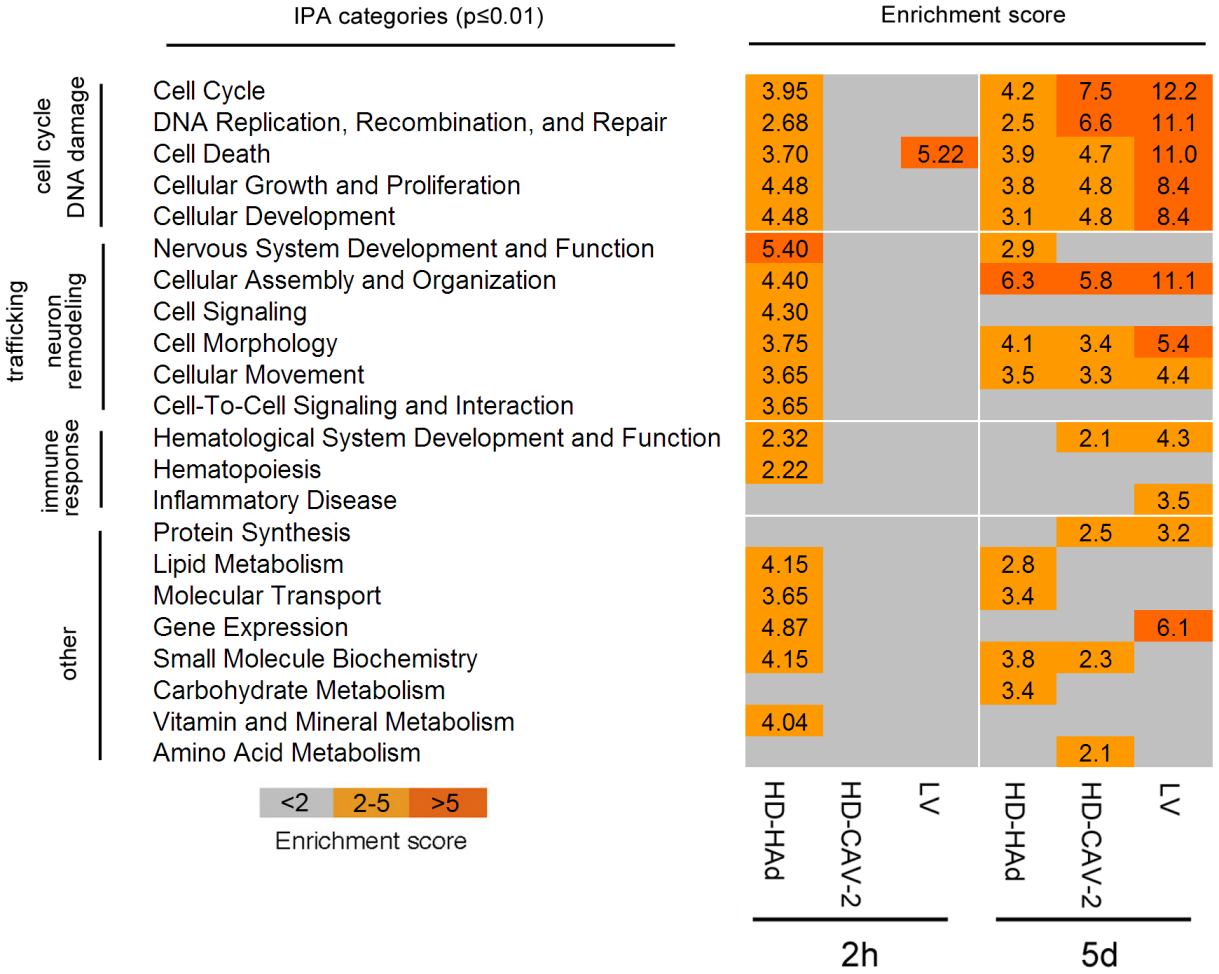
Functional gene categories significantly enriched by HD-HAd, HD-CAV-2 and LV. Data sets containing the Affymetrix probe identifiers selected as differentially expressed in transduced cells and the corresponding fold changes were uploaded onto IPA. Significant molecular and physiological functions were determined querying the IPA Knowledge Base and selecting a scoring method based on the Fisher's exact test applying the threshold of p-value≤0.01. Significantly enriched IPA categories containing at least 10 genes are listed and the relative score is indicated. Categories were further manually classified into four groups: i) cell cycle and DNA damage, ii) trafficking and neuron remodeling and iii) immune response, iv) other.

To define the signatures of the three vectors beyond the single gene level, we performed a cluster analysis with IPA on *limma* statistically restricted genes, and selected the functional categories scoring a p-value≤0.01, including a gene number ≥10 (**Fig. 2**). The response of differentiated hmNPCs to vectors involved a significant modulation of a number of genes and gene groups. Nonetheless, most of the modulated genes could be assigned to a restricted number of key biological events: cell cycle and DNA damage processes, neuron intracellular trafficking and remodelling, and activation of immune pathways (**Fig. 2**). At 2 h, we identified significantly regulated functional gene groups in HD-HAd cells, but not in HD-CAV-2 cells. At 2 h LV modulated one cluster, the IPA category “cell death”. At 5 days all vectors modulated five common cell cycle and DNA damage related categories, and LV attained the highest enrichment scores within these groups. All vectors regulated neuron remodelling gene groups. HD-CAV-2 and LV significantly modulated immune response genes, but for LV the “inflammatory disease” IPA category was the only one significantly enriched (**Fig. 2**). Taken together, these analyses showed a more structured picture than that obtained by direct single gene comparisons and demonstrated that HD-HAd, HD-CAV-2 and LV vectors had overlapping, yet distinct transcriptome signatures.

### HD-HAd, HD-CAV-2 and LV induced modulation of genes implicated in the cell cycle and DNA damage response

**Figure 3:**
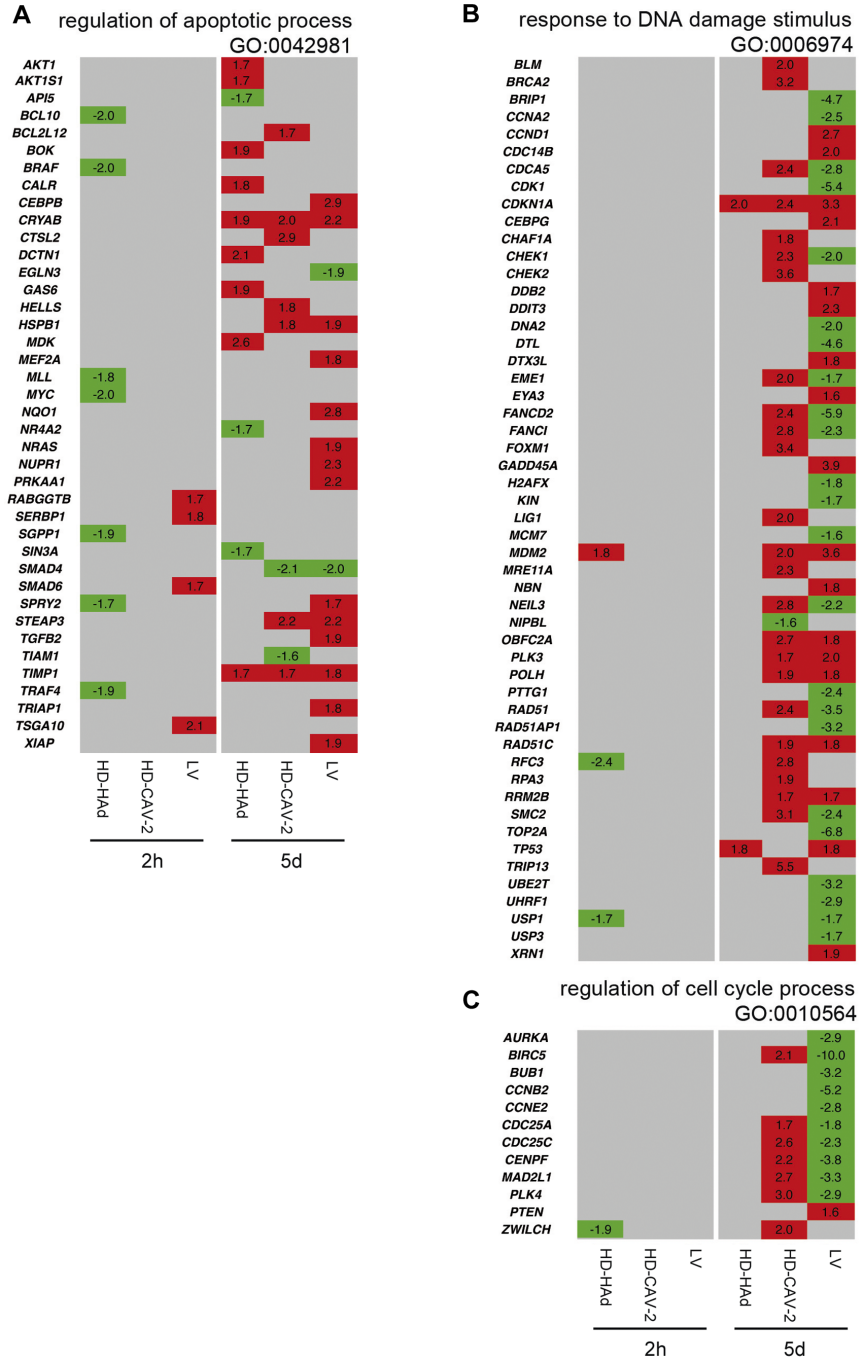
Comparative analysis of cell cycle and DNA damage gene expression profiles induced by HD-HAd, HD-CAV-2 and LV. (**a–c**) Heat maps of genes identified by combined IPA and GO analysis with a threshold set at p≤0.01. The relative gene modulation fold change values are indicated; in red, upregulated genes, in green, downregulated, and in grey, genes with unmodified expression with respect to mock. The most prominent trait of this analysis is the divergent modulation of the subgroup GO: 00006974 (“Regulation of DNA damage stimulus”) and GO: 0010564 (“Regulation of cell cycle process”) by HD-CAV-2 and LV at 5 days posttransduction.

**Figure 4:**
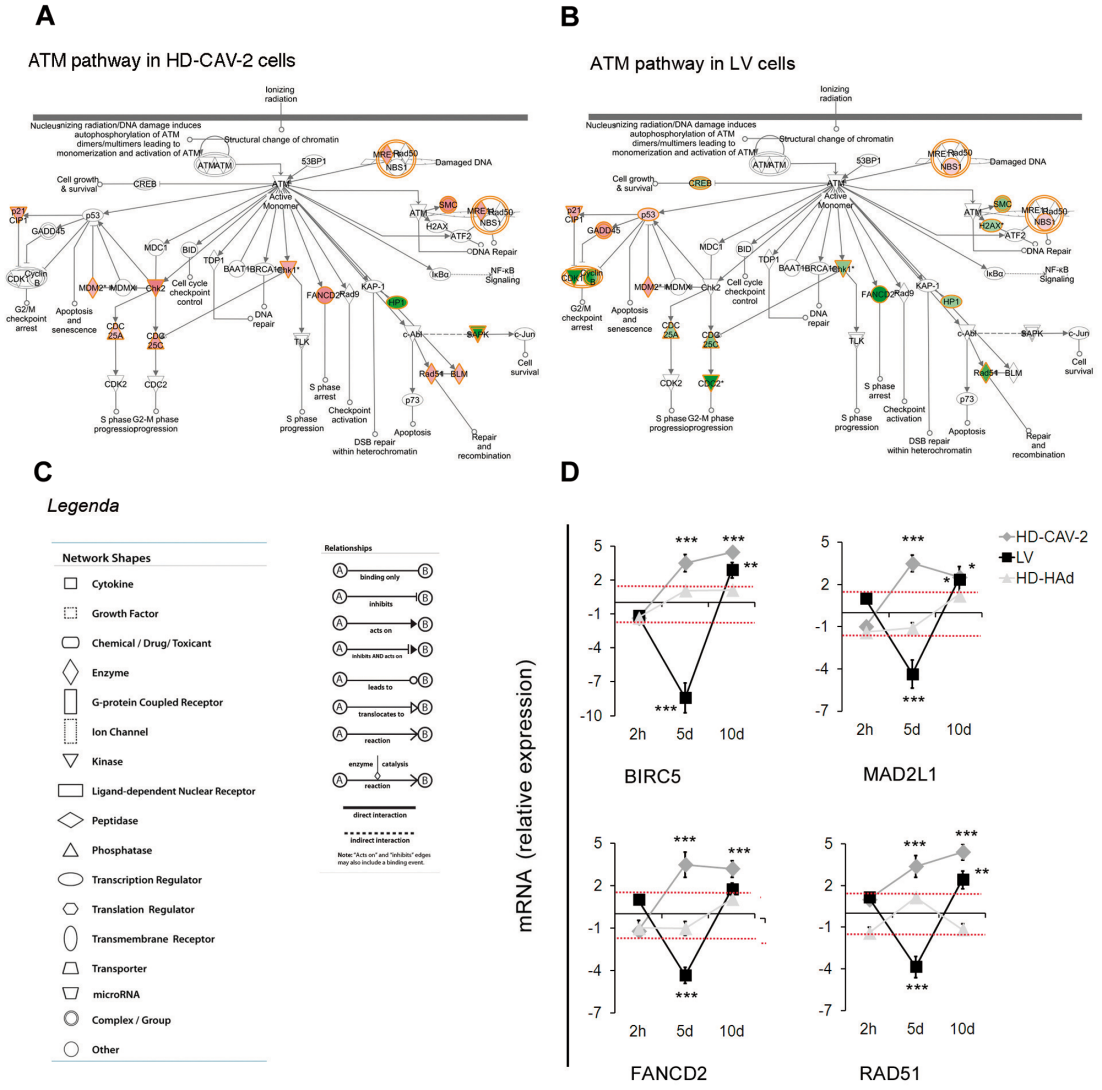
ATM signaling modulation by HD-CAV-2 and LV. (**a,b**) ATM pathway in HD-CAV-2 (a) and LV (b) cells at 5 days posttransduction. In red are highlighted the upregulated genes, in green, the downregulated ones, in white genes not modulated in the chip; IPA p-value, HD-CAV-2 1.68×10^−8^, LV 5.11×10^−11^. (**c**) The legend in the panels is valid for (a) and (b). (**d**) Single gene alterations were evaluated by qPCR 2 h, 5 and 10 days posttransduction. Data are presented as fold change expression of each transcript normalized with respect to the endogenous control and calculated as compared to the mock sample considered as 1. Red dashed lines display the threshold set at ±1.5, as for microarray data. Data are reported as mean of three independent experiments with SD; one-way ANOVA (vectors versus mock), * p<0.05, **p<0.01, ***p<0.001.

To refine the biological profile of transduced cells, we integrated IPA screening with PubMed and g:Profiler database analysis and report the data as Gene Ontology (GO) subgroups (full list on **Table S2 in File S1**). At 2 h, HD-HAd and LV modulated genes belonging to the “regulation of apoptotic process” subgroup (**Fig. 3a**). At 5 days, the number of modulated genes was overall more significant and concerned the “regulation of apoptotic process” (**Fig. 3a**), the “response to DNA damage stimulus” (**Fig. 3b**) and the “cell cycle process” (**Fig. 3c**). Genes belonging to the apoptotic group were in most cases upregulated (**Fig. 3a**). Because many of the upregulated genes had anti-apoptotic functions (e.g. *TIMP1*, *HSPB1*, *AKT1*), these results suggested a prosurvival response. The cell cycle regulation and the response to DNA damage were vector specific (**Fig. 3b,c**). Their modulation was significantly detected only in HD-CAV-2 and LV-transduced cells. In addition, most of the genes that were modulated in HD-CAV-2 and LV-treated cells were divergently regulated as a result of treatment with different vectors. The divergent action of LV and HD-CAV-2 was further highlighted when we analysed the pathway of ATM, a master gene of the DNA damage response. The ATM circuit was mostly positively regulated by HD-CAV-2 and negatively by LV (**Fig. 4a–c**). We then performed qPCR on samples collected at the 2 h, 5 and 10 days to validate and extend the data. qPCR confirmed arrays results at 2 h and 5 days .Indeed, *BIRC5*, *MAD2L1*, *FANCD2* and *RAD51* were significantly modulated by HD-CAV-2 and LV at 5 days - yet with opposite signs. At 10 days postinfection the downregulation effect of LV was lost, and HD-CAV-2 maintained a trend of upregulation. This suggested that the 5-day point represented vector specific signatures. On the other hand, the effect of LV on the DNA damage response was rescued once the early phase of viral transduction was concluded (**Fig. 4d**).

Together the data showed that dominant aspects of the response to HD-HAd, HD-CAV-2 and LV, mainly observed at 5 days, were: i) the upregulation of pro-survival genes, induced by all vectors, ii) the induction of the ATM pathway and of DNA damage response genes by HD-CAV-2, and iii) the downregulation of ATM signalling by LV. This suggested that while the pro-survival response was activated independently from the intracellular concentration of the vector and from its characteristics, the specific functional and structural properties of the vectors controlled the induction of DNA damage response genes.

### HD-HAd, HD-CAV-2, and LV vectors differentially altered genes implicated in immune response

**Figure 5:**
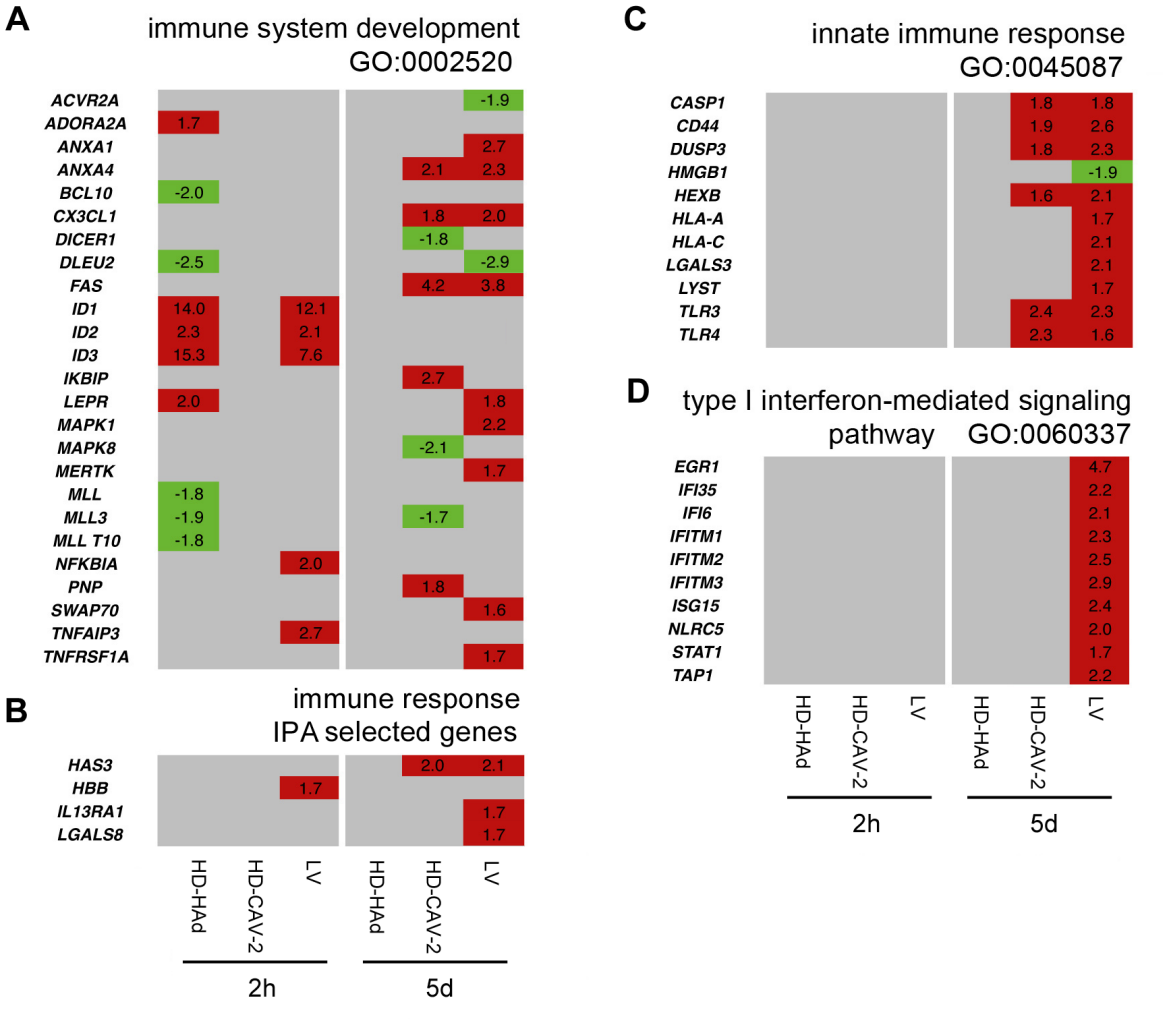
Comparative analysis of immune response gene expression profiles induced by HD-HAd, HD-CAV-2 and LV. (**a–d**) Heat maps of genes identified by combined IPA and GO analysis with a threshold set at p≤0.01, clustering in immune response categories. The relative gene modulation fold change values are indicated; in red, upregulated genes, in green, downregulated, and in grey, genes with unmodified expression with respect to mock. Of note is the LV dependent activation of the IFN subgroup (GO: 0060337) and the LV and HD-CAV-2 induced activation of the TLRs and HA related signaling pathway components at 5 days posttransduction.

**Figure 6:**
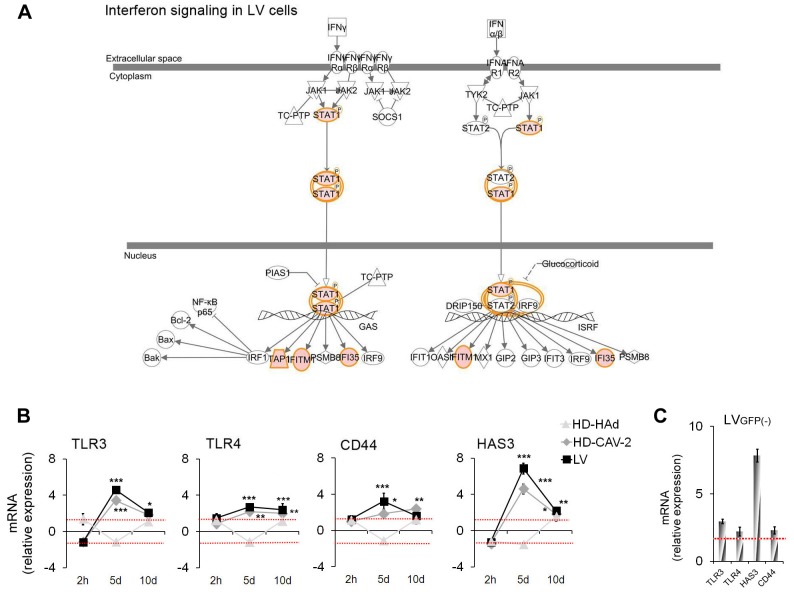
IFN and TLR genes in transduced cells. (**a**) IFN pathway in LV cells at 5 days posttransduction. In red are highlighted the upregulated genes, in green the downregulated ones, in white genes not modulated in the chip; IPA p-value: 3.56×10^−2^. Detailed legend is in figure 4c. (**b–c**) Single gene alterations evaluated by qPCR at 2 h, 5 and 10 days posttransduction for HD-hAd, HD-CAV-2 and LV (b), and at 5 days for LVGFP(-). (c). Red dashed lines display the threshold set at ±1.5, as for microarray data. Data are presented as fold change expression of each transcript normalized with respect to the endogenous control and calculated as compared to the mock sample considered as 1. Data are reported as mean of three independent experiments with SD; one-way ANOVA (vectors versus mock); * p<0.05, ** p<0.01, ***p<0.001.

Combined functional analyses were used to dissect the modulation of genes related to the immune response. We identified three significantly enriched categories by GO (immune system development, innate immune response, type I interferon-mediated signalling pathways) and also highlighted four immune response genes identified only by IPA (*HAS3, HBB, IL13RA1 and LGALS8*) which are of interest in the analysis of the immune reaction to vectors and are related to the genes included in the gene ontology enriched categories (**Fig. 5**, **Table S2 in File S1**). Specifically, at 2 h, in LV transduced cells, we did not observe a significant enrichment of genes belonging to these groups, but we detected the upregulation of *NFKBIA*, *TNFAIP3* and of *HBB*, recently found to have protective role in response to brain injury [29] (**Fig. 5a,b**). HD-HAd significantly altered genes belonging to the “immune system development” GO subgroup. At 5 days, both HD-CAV-2 and LV activated the “immune system development” and the “innate immune response” (**Fig. 5a,c**), including *TLR3*, *TLR4* (**Fig. 5c**) and *TLR-*activated *XBP1* (**Table S2 in File S1**). They stimulated the expression of *CASP1*, which induces the secretion of the inflammatory cytokine IL1β, and of *HAS3* and *CD44*, respectively the enzyme implicated in the synthesis of the hyaluronan (HA) and the HA receptor (**Fig. 5c**). In LV cells only, besides the genes described above, we observed the activation of IFN-related molecules (**Fig. 5d**). The dynamics of IFN signalling was further supported by pathway analysis, which showed the interrelationships among the different molecules activated by IFN (γ and α/β) in LV transduced cells (**Fig. 6a**). We confirmed by qPCR the induction of the expression of *TLR3*, *TLR4*, *HAS3* and *CD44* in HD-CAV-2 and LV cells at 5 days. qPCR performed at 2 h and 10 days showed that the peak of the immune response was indeed at 5 days postinduction as compared to the earlier and later times (**Fig. 6b**). Transgene expression has been taken into consideration in numerous gene therapy contexts to evaluate its contribution to adverse reactions, both in *vitro* and in *vivo* models [30], [31]. To assess whether the modulation of *TLR3*, *TLR4*, *HAS3* and *CD44* in hmNPCs was a vector specific signature in hmNPCs rather than a response of these cells to GFP expression, we also performed qPCR analysis using a virus devoid of transgene (LV GFP(-)). The modulation by LV GFP(-) indicated that the regulation of these molecules was related to the virion components and/or to the viral infection process, rather than to GFP immunogenicity/toxicity (**Fig. 6c**).

These data indicated that the dominant aspects, comparing the 2 h, 5 and 10 days points, of the immune response happened at 5 days. At this time, LV and HD-CAV-2 cells generated a common and antigen-independent reaction, consisting of the induction of genes of the innate response including *TLRs* and *CD44*. Notably, in LV-transduced cells genes related to the IFN response were upregulated. The effect of HD-HAd on the immune response genes was weak, which could be ascribed to the less effective transduction of hmNPCs as compared to LV and HD-CAV-2 vectors.

### Activation of genes involved in neuron trafficking and remodelling

**Figure 7:**
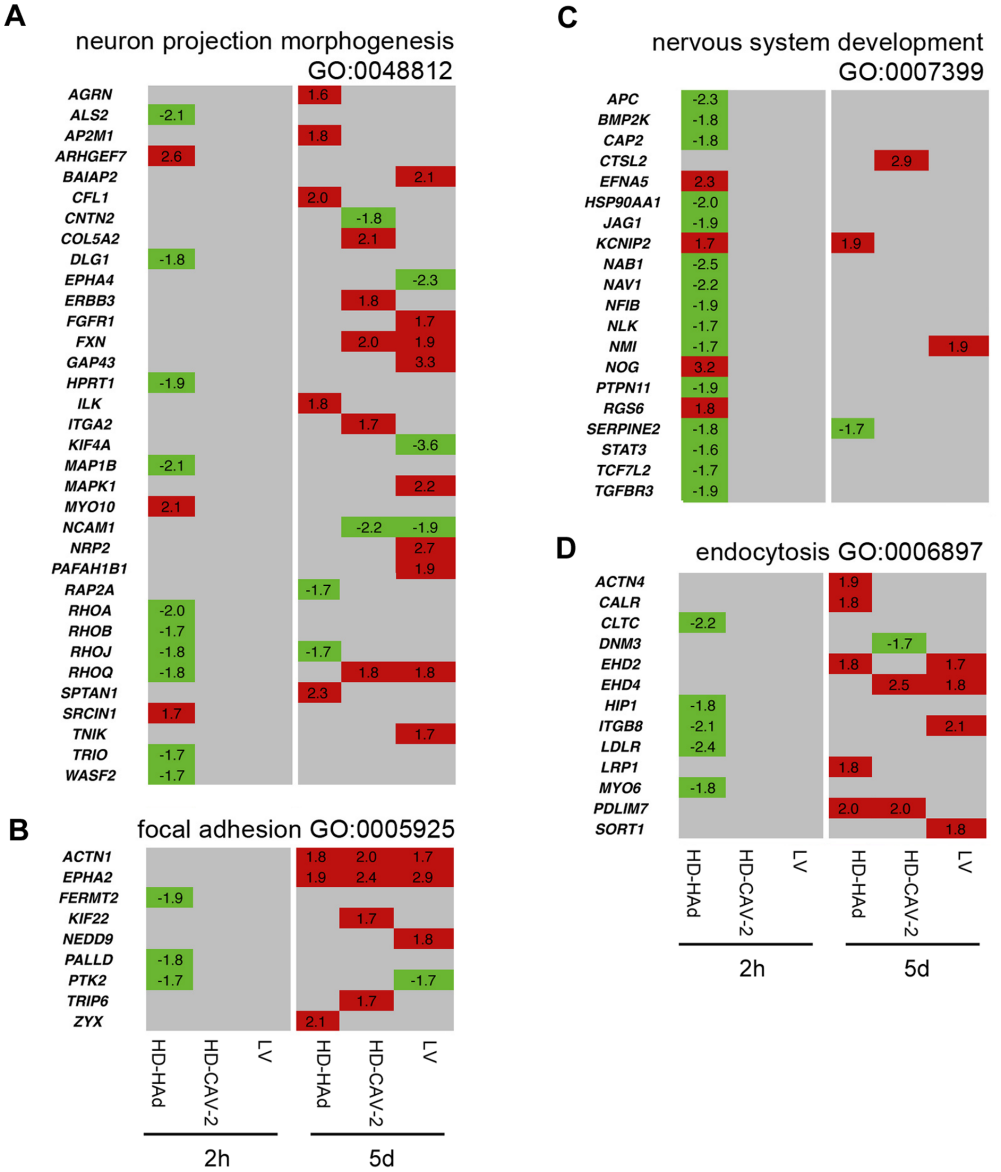
Comparative analysis of trafficking and neuron remodeling gene expression profiles. (**a–d**) Heat maps of genes identified by combined IPA and GO analysis with a threshold set at p≤0.01, clustering in trafficking and neuron remodeling categories. The relative gene modulation change values are indicated; in red upregulated genes, in green downregulated genes, and in grey genes with unmodified expression with respect to mock treated samples.

**Figure 8:**
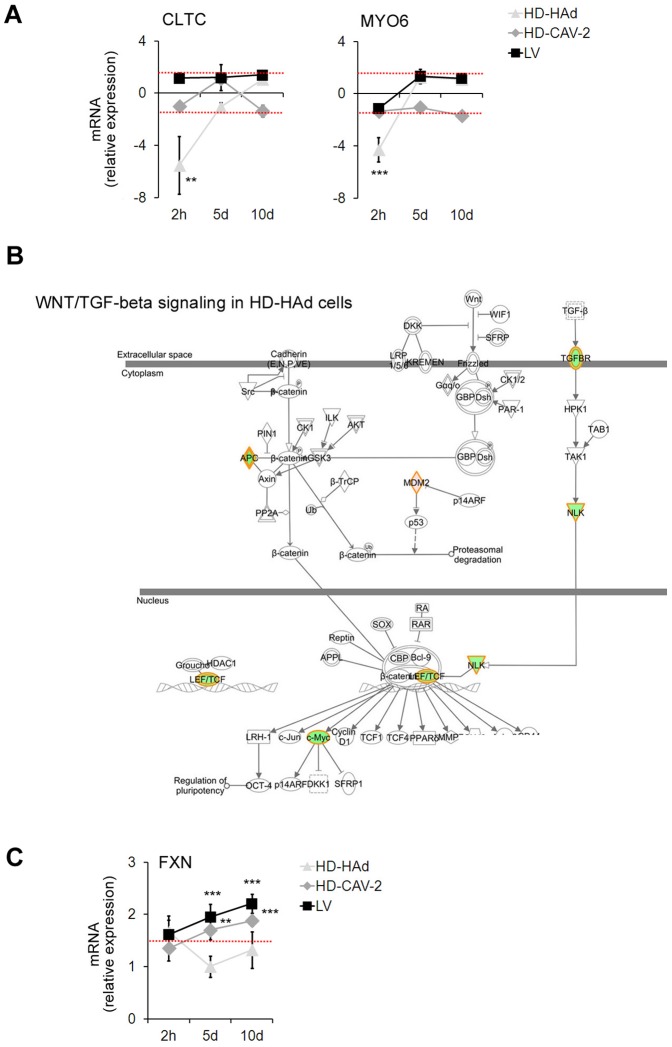
Downregulation of endocytotic and Wnt genes in HD-HAd cells. (**a**) *CLTC* and *MYO6* modulations were evaluated by qPCR at 2 h, 5 and 10 days posttransduction. Data are presented as fold change expression of each transcript normalized with respect to the endogenous control and calculated as compared to the mock sample considered as 1. Red dashed lines display the threshold set at ±1.5, as for microarray data. Data are reported as mean with SD. All samples were tested in triplicate; one-way ANOVA (vectors versus mock), ** p<0.01, , *** p<0.001. (**b**) Wnt/TGF-β pathway in HD-HAd cells 2 h posttransduction. In red are highlighted the upregulated genes, in green the downregulated ones, in white genes not modulated in the chip; IPA p-value 4.01×10^−2^. A detailed legend is in figure 4c. (**c**) *FXN* modulation quantified by qPCR at 2 h, 5 and 10 days days posttransduction. Data are presented as fold change expression of each transcript normalized with respect to the endogenous control as in (a).

The next biological feature that we analysed was the regulation of genes related to intracellular trafficking and neuron remodelling. We found significant enrichment of modulated genes involved in the control of “neuron projection morphogenesis”, “nervous system development”, “focal adhesion” and of “endocytosis” (**Fig. 7a–d** **, Table S2 in File S1**). HD-HAd induced a strikingly widespread transcriptional repression of these genes at 2 h. By qPCR, we confirmed the decrease of the expression of two crucial components of the endocytosis, *CLTC* and *MYO6*, by HD-HAd at 2 h, an effect that was not present at 5 and 10 days (**Fig. 8a**). A further notable aspect of HD-HAd-induced early modulation was the downregulation of genes involved in the signalling cascade of TGFβ and Wnt, a network controlling cell-to-cell communication and development (**Fig. 8b** **, Table S2 in File S1**).

Five days after transduction, all vectors affected, mostly with positive sign, the “neuron projection morphogenesis”, “focal adhesion” and “endocytosis” GO subgroups (**Fig. 7a,b,d**). In LV-transduced neurons we observed the modulation of *ACTN1*, and of ephrin receptor related genes, important for axon guidance and cell migration. In HD-CAV-2 neurons, we found the positive regulation of molecules related to the integrin and actin signalling (*ITGA2*, *ACTN1*, *RHOQ*, *FXN*), the altered transcription of specific cell-adhesion components (*CNTN2*, *NCAM1*), and the activation of *EPHA2*. In HD-HAd cells at 5 days, molecules having a role in the cytoskeletal and vesicle reorganization (including *SPTAN1*, *CFL1*, *EPHA2*, *ILK*, *RAP2A*) along with *ACTN1*, *RHOJ* and *ZYX*, involved in integrin signalling were modulated. *FXN* was upregulated by HD-CAV-2 and by LV at 5 days postincubation, and remained upregulated also at later times (10 days, **Fig. 8c**).

Taken together, these data suggested that at 2 h only HD-HAd altered neuronal development and trafficking, and, at later times, all vectors influenced cytoskeletal reorganization and neuron remodelling involving the integrin and ephrin pathways.

## Discussion and Conclusions

In this study we analysed the transcriptional response of human neurons to three vectors that have distinct characteristics relevant for clinical gene therapy for the brain. Our goal was to produce an absolute and relative toxicogenomic profile of these vectors in a clinically relevant model, i.e. in differentiated human midbrain neuroprogenitor cells that acquire morphological and functional properties of dopaminergic neurons. The cells were readily transduced with the fixed amount of 1000 vector genomes/cell of HD-HAd, HD-CAV-2 or LV vectors. At this vector dose AAV2/9 vector was inefficient and therefore could not be compared. HD-CAV-2 and LV were more efficient than HD-HAd. Globally, the impact of HD-HAd, HD-CAV-2 and LV on the transcriptome was moderate, suggesting that human midbrain neurons may be relatively tolerant to vector-mediated transduction. The vectors however significantly affected three main biological pathways, the cell cycle and DNA damage response, the neuron trafficking and remodelling processes, and the immune response, and the profiles of these functions were distinct and partly overlapping for the three vectors.

### ATM signalling

A strong and clear-cut effect of incubation of differentiated hmNPCs with HD-CAV-2 and LV was the activation of the DNA damage response. HD-CAV-2 and LV induced several genes of the p53 network, but, at 5 days posttransduction, the pattern of the downstream and linked effectors diverged. In cells transduced with HD-CAV-2, the upregulation of a plethora of genes strongly indicated the activation of an ATM-dependent signalling pathway. ATM is a kinase activated by DNA damage and in particular by double strand breaks (DSBs). The association of ATM and Ad was expected, considering that the infection with Ad exposes the host cell to exogenous linear episomal DNA [32]. Consistent with this, Ad proteins counteract the cellular response by preventing the recognition of the viral free DNA termini and targeting the Mre11-Rad50-Nbs1 complex to proteasome degradation [32]. Given that HD vectors are devoid of all viral genes, it is not surprising that HD-CAV-2 activated DNA damage processes. Surprisingly, HD-HAd-transduced cells did not display the same strong modulation of the ATM pathway as HD-CAV-2- transduced cells. The most likely explanation to this result is the relative level of intracellular vector copies, which was >10-fold higher for HD-CAV-2 than for HD-HAd at 5 days posttransduction. However, we cannot exclude that the nature of HD-CAV-2 differentially impacts on human neurons compared to HD-HAd. Indeed, HD-CAV-2 receptor engagement and internalization in neurons is likely different from that of HD-HAd [33]. Whether the different human stuffer sequences, inverted terminal repeats and packaging domains (<500 bp) in HD-CAV-2 and HD-HAd differentially affects is possible, but we believe unlikely.

In contrast to HD-CAV-2, a dominant response of LV-transduced cells at 5 days posttransduction was the repression of DNA damage and cell cycle genes, which was rescued at later times. The downregulation has at least two possible interpretations: one is that DNA repair processes is a passive response of neurons to LV-induced stress; or that an LV component actively modulated the DNA damage and cell cycle circuits to facilitate LV propagation. In support of the first hypothesis is that HIV-1 can create damage in the cell genome [34]. Indeed, we observed in LV cells the activation of p53 signalling, the modulation of cell cycle genes towards cell arrest and the suppression of homologous recombination genes. However, we favour the possibility of an active role of LV components. In this regard, it is worth noting that RAD51 was downregulated in LV-transduced neurons. Enhanced homologous recombination mediated by the stabilization of RAD51 and the formation of RAD51 nucleofilaments on the integration complex is detrimental for the HIV-1 integrase activity [35]. In addition, the homologous recombination mechanism, strongly downregulated in LV cells, is responsible of the formation of 1-LTR HIV-1 DNA, a circular form that does not give rise to infectious progeny [36]. In line with this second hypothesis, is also the upregulation of *NBN*. Indeed, NBN may be important for LV integration, for filling in the single strand gaps and for sealing the nicks left at the sites of viral insertion and for chromatin remodelling [37], [38]. At 10 days, the effect was rescued with a modest upregulation of DNA repair genes induced by LV. This can be explained by the fact that the active integration process was overcome at 10 days and residual unintegrated copies represented a DNA damage stimulus.

These data indicated that sensing vector genomes was a dominant feature of the cellular response to vectors. HD-CAV-2 strongly induced the DNA damage response, which can be ascribed to the relative quantity of episomal genomes in our system. LV caused a strong and complex repression of the DNA damage and cell cycle pathways, for which it remains to be determined if all the aspects observed were due to the stress of viral genome integration, or if part of the response was actively modulated by the vector.

### Toll-like receptors, hyaluronan circuit activation

Differentiated hmNPCs had a moderate induced immune response to the vectors. One notable aspect of the response to HD-CAV-2 and LV was the upregulation of TLR3 and TLR4 transcripts. TLRs play a role in the innate immune system and represent the first line of defence against pathogens through recognition of conserved microbial structures [39]. Although TLR activation by HAd and HIV-1 vectors has been described in other models ([40] and references therein), this is the first study analysing the TLR response to viral vectors in human neurons. In HD-HAd transduced differentiated hmNPCs, the absence of a detectable TLR activation was likely due to the lower intracellular viral copy number as compared to HD-CAV-2 and LV cells. Intriguingly, together with the modulation of TLRs, we found that HD-CAV-2 and LV robustly induced the HA network, at 5 days posttransduction. HA accumulation in the extracellular matrix triggers chemokine release and recruitment of inflammatory cells [41], [42]. Taken together, our data demonstrates that, in human midbrain neurons, HD-CAV-2 and LV activated innate, nonspecific arms of the immune response.

### Interferon signalling and MHC class I modulation by LV

In neurons incubated with LV, but not HD-HAd and HD-CAV-2 vectors, we identified a robust activation of type I IFN signalling. These results are in accordance with data showing that LV triggers an IFN response [13], [43]. Because we observed that LV induced the upregulation of MHC class I elements (*HLA-A*, *HLA-C*, *TAP1*) and that of *NLRC5*, which can transcriptionally activate MHC class I genes and related components [44], we propose that the MHC class I LV-induced response could be controlled by *NLRC5*. Given the link between TLRs and IFN, and that between DNA damage and IFN, the IFN response to LV was likely related both to LV genomic RNA, and to the genotoxic stress generated by integration, which was also LV specific.

### Wnt signalling repression by HD-HAd

At 2 h postincubation, a main response to HD-HAd was the widespread downregulation of differentiation and cell assembly related genes, consistently with what we previously reported [17]. HD-HAd provoked the decline of transcripts from genes implicated in neuronal development, including factors involved in the TGF-β/Wnt signalling, which has a pivotal impact in midbrain DA neuron development [45]. For cell binding and internalization, HAd serotype 5 uses integrins [46], which are cell adhesion molecules essential for establishing neuronal networks and projections [47], [48]. These interactions could explain the HAd-induced interference on neuronal differentiation processes, which deserves further investigation.

### Prosurvival genes and neuron remodeling processes

At 5 days, all vectors induced the positive modulation of pro-survival genes, including *TIMP1*. *HSPB1*, which was stimulated by HD-CAV-2 and LV, *HSPB1* overexpression has been described as a cytoprotective response in traumatic nerve injury [49]. The lack of the *TIMP1* leads to neuronal cell death [50]. *AKT1*, whose activation was observed in neurons incubated with HD-HAd, is also a cell survival factor activated in response to DNA insults [51].

The three vectors also modulated the actin, integrin and ephrin circuits, which are inter-connected and related to growth cone collapse and cell attachment [52]. Rather than being related to the interaction of the vectors with specific receptors, which were different for HD-HAd, HD-CAV-2 and for LV, these events can be ascribed to viral internalization and intracellular trafficking. We propose that all transduced cells responded to vector interaction with neurite outgrowth and neuron remodeling. Taken together, these data showed that LV, HD-CAV-2 and HD-HAd, independently of the entry route and of their specific impact on differentiated hmNPCs, activated a pro-survival response, and presumably as a consequence to viral-induced membrane perturbation and neuron remodeling. Both responses may play a role in re-establishing neuron homeostasis.

### Conclusions

Chip array analysis of HD-HAd, HD-CAV-2 and LV vector-transduced cells that have the hallmarks of DA neurons resulted in an extensive picture of their molecular interaction. In no case was the effect of the vector neutral, although the extent of the alterations was never severe. Vector-specific responses were the negative effect of HD-HAd on the progression of the neuronal differentiation and the IFN response to LV. Common to HD-CAV-2 and to LV were the activation of the innate arm of the immune response and the divergent modulation of the DNA damage pathways at 5 days posttransduction. As a general response to vector interaction, human neurons activated pro-survival genes and neuron morphogenesis. Considering the global transcriptional impact and effectiveness of transduction, HD-CAV-2 arguably had the most promising profile in human midbrain neurons. Indeed, HD-CAV-2 did not negatively affect neuronal development, especially if compared to HD-HAd, and induced a milder immune response as compared to LV, at equal transduction efficiencies. Gene transfer to neurons holds significant clinical promise, thus knowing the specific response of human neurons to vectors is important. Our data give insights on the properties of the three viral vectors, on the specific tolerance of human neurons to vector treatment, and can contribute to the safer use of these vectors in brain gene therapy experiments.

## Supporting Information

File S1 **Supporting Information.**
(DOCX)
