# Supplementary material for: Differentiated Neuroprogenitor Cells Incubated with Human or Canine Adenovirus, or Lentiviral Vectors Have Distinct Transcriptome Profiles
Source: PLoS One. 2013 Jul 26;8(7):e69808. doi: 10.1371/journal.pone.0069808 (PMC3724896; doi:10.1371/journal.pone.0069808)
Supplement: File S1 — Supporting Information. (DOCX) [file pone.0069808.s001.docx]

**Supporting information**

**Table S1:** Full list of modulated genes scoring a p value ≤ 0.05 for the three vectors (HD-HAd, HD-CAV-2 and LV) at the 2 h and 5 days (5d) time points.

HD-HAd 2h

| **Probe Set ID** | **Gene Symbol** | **Gene Title** | **Accession number** | **Entrez Gene** | **Fold Change** | **PValue** |
| --- | --- | --- | --- | --- | --- | --- |
| 210230_at | --- | CDNA: FLJ23438 fis, clone HRC13275 | BC003629 |  | -2.811869252 | 0.005563544 |
| 242228_at | --- | gb:AA825721 /DB_XREF=gi:2899033 /DB_XREF=od29f04.s1 /CLONE=IMAGE:1369375 /FEA=EST /CNT=5 /TID=Hs.246973.0 /TIER=ConsEnd /STK=1 /UG=Hs.246973 /UG_TITLE=ESTs | AA825721 |  | -2.325462748 | 0.00404021 |
| 227762_at | --- | Transcribed locus | AW244016 |  | -2.189037003 | 0.018396861 |
| 239735_at | --- | MRNA (fetal brain cDNA g6_1g) | N67106 |  | -2.147486668 | 0.02369774 |
| 236521_at | --- | Transcribed locus | BF196060 |  | -2.012725687 | 0.01129373 |
| 228854_at | --- | Transcribed locus | AI492388 |  | -1.985254704 | 0.024322415 |
| 240165_at | --- | Transcribed locus, weakly similar to XP_209041.2 PREDICTED: similar to KIAA1503 protein [Homo sapiens] | AI678013 |  | -1.892926071 | 0.003527869 |
| 226834_at | --- | Transcribed locus, strongly similar to NP_079045.1 adipocyte-specific adhesion molecule; CAR-like membrane protein [Homo sapiens] | BG112263 |  | -1.845901806 | 0.015702527 |
| 239231_at | --- | CDNA FLJ41910 fis, clone PEBLM2007834 | BE464819 |  | -1.728485364 | 0.009716067 |
| 1557630_s_at | --- | CDNA clone IMAGE:5273415 | BC037874 |  | -1.710960362 | 0.005653122 |
| 237476_at | --- | Transcribed locus | BE463995 |  | 1.657121272 | 0.00225846 |
| 239278_at | --- | CDNA clone IMAGE:5301129 | AI471969 |  | 1.67330691 | 0.029424109 |
| 239423_at | --- | gb:AW043836 /DB_XREF=gi:5904365 /DB_XREF=wy81d11.x1 /CLONE=IMAGE:2554965 /FEA=EST /CNT=5 /TID=Hs.212460.0 /TIER=ConsEnd /STK=4 /UG=Hs.212460 /UG_TITLE=ESTs | AW043836 |  | 1.739105944 | 0.032310568 |
| 239866_at | --- | CDNA FLJ14392 fis, clone HEMBA1003166 | AA705933 |  | 1.749948963 | 0.022224016 |
| 236712_at | --- | Transcribed locus | AI668706 |  | 1.785928603 | 0.012969338 |
| 214353_at | --- | Transcribed locus | AW241864 |  | 1.79679501 | 0.019145999 |
| 1560973_a_at | --- | Full length insert cDNA clone ZD78G02 | AF086424 |  | 2.081266934 | 0.008894596 |
| 213403_at | --- | Clone 23908 mRNA sequence | BF223370 |  | 2.085754841 | 0.000191266 |
| 236576_at | --- | Transcribed locus | N63005 |  | 2.141623751 | 0.021115738 |
| 231383_at | --- | Transcribed locus | AW016250 |  | 2.345302949 | 0.000334575 |
| 241156_at | --- | MRNA; cDNA DKFZp686B06256 (from clone DKFZp686B06256) | AI939588 |  | 2.34976027 | 0.010271969 |
| 241721_at | --- | CDNA FLJ37844 fis, clone BRSSN2012622 | AW515022 |  | 2.402964303 | 0.002895492 |
| 1554878_a_at | ABCD3 | ATP-binding cassette, sub-family D (ALD), member 3 | BC009712 | 5825 | -2.375515038 | 0.013149939 |
| 201660_at | ACSL3 | Acyl-CoA synthetase long-chain family member 3 | AL525798 | 2181 | -1.81764371 | 0.00591045 |
| 201661_s_at | ACSL3 | acyl-CoA synthetase long-chain family member 3 | NM_004457 | 2181 | -1.706505838 | 0.031293294 |
| 205013_s_at | ADORA2A /// CYTSA | adenosine A2a receptor | NM_000675 | 135 | 1.722742171 | 7.71826E-05 |
| 229309_at | ADRB1 | Beta-1 adrenergic receptor mRNA, 3' UTR | AI625747 | 153 | -1.930105402 | 0.039867914 |
| 222458_s_at | AKIRIN1 | chromosome 1 open reading frame 108 | AI205764 | 79647 | -2.208553627 | 0.017300614 |
| 219015_s_at | ALG13 | glycosyltransferase 28 domain containing 1 | NM_018466 | 55849 | -1.743586944 | 0.043026831 |
| 232184_at | ALS2 | amyotrophic lateral sclerosis 2 (juvenile) | AK023024 | 57679 | -2.136658944 | 0.017150819 |
| 216933_x_at | APC | adenomatosis polyposis coli | S67788 | 324 | -2.255916945 | 0.033253925 |
| 210067_at | AQP4 | aquaporin 4 | D63412 | 361 | -1.789202233 | 0.023359166 |
| 241701_at | ARHGAP21 | Rho GTPase activating protein 21 | BF369489 | 57584 | -1.939842274 | 0.016920404 |
| 227911_at | ARHGAP28 | Rho GTPase activating protein 28 | AI935647 | 79822 | -2.03158933 | 0.002858878 |
| 203910_at | ARHGAP29 | Rho GTPase activating protein 29 | NM_004815 | 9411 | -3.396439693 | 0.010550893 |
| 235412_at | ARHGEF7 | Rho guanine nucleotide exchange factor (GEF) 7 | AI040887 | 8874 | 2.594381495 | 0.008741634 |
| 201879_at | ARIH1 | ariadne homolog, ubiquitin-conjugating enzyme E2 binding protein, 1 (Drosophila) | AI694332 | 25820 | -1.732855163 | 0.00264091 |
| 242727_at | ARL5B | ADP-ribosylation factor-like 5B | BG032269 | 221079 | -1.646385744 | 0.047841354 |
| 210971_s_at | ARNTL | aryl hydrocarbon receptor nuclear translocator-like | AB000815 | 406 | -1.82792825 | 0.002605897 |
| 1555419_a_at | ASAH1 | N-acylsphingosine amidohydrolase (acid ceramidase) 1 | BC016828 | 427 | -1.782396438 | 0.030106468 |
| 236533_at | ASAP1 | development and differentiation enhancing factor 1 | AW236958 | 50807 | -1.71907495 | 0.020424837 |
| 227014_at | ASPHD2 | aspartate beta-hydroxylase domain containing 2 | BE550881 | 57168 | -1.926784529 | 0.039776351 |
| 228890_at | ATOH8 | atonal homolog 8 (Drosophila) | BF434995 | 84913 | 1.939095812 | 0.007730017 |
| 201971_s_at | ATP6V1A | ATPase, H+ transporting, lysosomal 70kDa, V1 subunit A | NM_001690 | 523 | -2.310915559 | 0.011857163 |
| 1557257_at | BCL10 | B-cell CLL/lymphoma 10 | AA994334 | 8915 | -1.958775244 | 0.009128082 |
| 223566_s_at | BCOR | BCL6 co-repressor | AF317391 | 54880 | -1.948504245 | 0.039117698 |
| 1554020_at | BICD1 | bicaudal D homolog 1 (Drosophila) | BC010091 | 636 | -2.060646271 | 0.013237839 |
| 214806_at | BICD1 | gb:U90030.1 /DB_XREF=gi:2745977 /GEN=BICD /FEA=mRNA /CNT=14 /TID=Hs.164975.2 /TIER=ConsEnd /STK=0 /UG=Hs.164975 /LL=636 /DEF=Homo sapiens bicaudal-D (BICD) mRNA, alternatively spliced, partial cds. /PROD=bicaudal-D | U90030 | 636 | -1.819154635 | 0.009301485 |
| 59644_at | BMP2K | BMP2 inducible kinase | AI735391 | 55589 | -1.785581782 | 0.003064629 |
| 37170_at | BMP2K | BMP2 inducible kinase | AB015331 | 55589 | -1.719017348 | 0.018393254 |
| 243829_at | BRAF | v-raf murine sarcoma viral oncogene homolog B1 | AW613053 | 673 | -1.974302277 | 0.000144028 |
| 238946_at | C11orf54 | Chromosome 11 open reading frame 54 | AW469495 | 28970 | -1.978586302 | 0.005696239 |
| 235026_at | C12orf66 | hypothetical protein FLJ32549 | AI885871 | 144577 | -1.829392793 | 0.025730828 |
| 233106_at | C14orf82 | chromosome 14 open reading frame 82 | AU148054 | 145438 | -1.695495473 | 0.004837976 |
| 227963_at | C17orf76-AS1 | Chromosome 17 open reading frame 45 | BF515913 | 125144 | 2.050826108 | 0.006121312 |
| 223404_s_at | C1orf25 | chromosome 1 open reading frame 25 | AW512122 | 81627 | -1.657550533 | 0.007863579 |
| 1553711_a_at | C4orf39 | hypothetical protein FLJ31659 | NM_153027 | 152756 | -1.751486075 | 0.012128016 |
| 213872_at | C6orf62 | Chromosome 6 open reading frame 62 | BE465032 | 81688 | 1.692919358 | 0.037286136 |
| 227534_at | C9orf21 | chromosome 9 open reading frame 21 | AI655189 | 195827 | -1.783285221 | 0.026056418 |
| 238435_at | CA5B /// CA5BP | carbonic anhydrase VB-like | AA521288 | 340591 | -1.910338974 | 0.020492935 |
| 214845_s_at | CALU | calumenin | AF257659 | 813 | -1.843466945 | 0.047041444 |
| 212551_at | CAP2 | CAP, adenylate cyclase-associated protein, 2 (yeast) | NM_006366 | 10486 | -1.783095025 | 0.011236124 |
| 241360_at | CCDC15 | Coiled-coil domain containing 15 | BG413368 | 80071 | -1.810669315 | 0.003487887 |
| 220466_at | CCDC15 | coiled-coil domain containing 15 | NM_025004 | 80071 | -1.768927317 | 0.002506635 |
| 213183_s_at | CDKN1C | Cyclin-dependent kinase inhibitor 1C (p57, Kip2) | N95363 | 1028 | -2.122493558 | 0.036267303 |
| 238154_at | CEP70 | Centrosomal protein 70kDa | AI285884 | 80321 | 1.64821647 | 0.010648154 |
| 1559590_at | CHDH | choline dehydrogenase | AA609488 | 55349 | -2.143660683 | 0.004896577 |
| 1570165_at | CHST11 | Carbohydrate (chondroitin 4) sulfotransferase 11 | BC027983 | 50515 | -1.755428974 | 0.001399742 |
| 238646_at | CLTC | Clathrin, heavy polypeptide (Hc) | AA747756 | 1213 | -2.237923751 | 0.003708908 |
| 225009_at | CMTM4 | CKLF-like MARVEL transmembrane domain containing 4 | AA191708 | 146223 | -1.914750886 | 0.008347163 |
| 1560225_at | CNR1 | Cannabinoid receptor 1 (brain) | AI434253 | 1268 | -2.101429003 | 0.0486765 |
| 1559005_s_at | CNTLN | chromosome 9 open reading frame 39 | AK098502 | 54875 | -2.048213751 | 0.043190228 |
| 224828_at | CPEB4 | cytoplasmic polyadenylation element binding protein 4 | AV704132 | 80315 | -2.250850604 | 0.006245163 |
| 224829_at | CPEB4 | cytoplasmic polyadenylation element binding protein 4 | AA772278 | 80315 | -2.037183631 | 0.006341252 |
| 238733_at | CPM | Carboxypeptidase M | AI422414 | 1368 | 1.928065341 | 0.029284692 |
| 239034_at | CXorf24 | chromosome X open reading frame 24 | BE548277 | 203414 | -1.693241786 | 0.001822054 |
| 216607_s_at | CYP51A1 | cytochrome P450, family 51, subfamily A, polypeptide 1 | U40053 | 1595 | -1.695898832 | 0.046670619 |
| 205371_s_at | DBT | dihydrolipoamide branched chain transacylase E2 | M27093 | 1629 | -1.689040515 | 0.012045349 |
| 221745_at | DCAF7 | WD repeat domain 68 | BE538424 | 10238 | -1.761162629 | 0.012486275 |
| 230180_at | DDX17 | DEAD (Asp-Glu-Ala-Asp) box polypeptide 17 | AA521056 | 10521 | -2.199860544 | 0.021890424 |
| 212834_at | DDX52 | DEAD (Asp-Glu-Ala-Asp) box polypeptide 52 | BE963238 | 11056 | 1.992207655 | 0.000762556 |
| 207431_s_at | DEGS1 | degenerative spermatocyte homolog 1, lipid desaturase (Drosophila) | NM_003676 | 8560 | -1.949863653 | 0.020524173 |
| 239730_at | DGCR14 | DiGeorge syndrome critical region gene 13 | AA662761 | 26221 | 2.239292061 | 0.004982475 |
| 201790_s_at | DHCR7 | 7-dehydrocholesterol reductase | AW150953 | 1717 | -2.002719747 | 0.010049031 |
| 201791_s_at | DHCR7 | 7-dehydrocholesterol reductase | NM_001360 | 1717 | -1.839778568 | 0.017655944 |
| 231240_at | DIO2 | deiodinase, iodothyronine, type II | AI038059 | 1734 | 3.74978711 | 0.02715151 |
| 1560763_at | DIP2C | DIP2 disco-interacting protein 2 homolog C (Drosophila) | BC033548 | 22982 | -2.079627758 | 0.044002829 |
| 231954_at | DKFZP434I0714 | hypothetical protein DKFZP434I0714 | AL137273 | 54553 | -1.627328991 | 0.003123427 |
| 1569476_at | DKFZP434L187 | DKFZP434L187 protein | BC033224 | 26082 | -2.80114684 | 0.016576916 |
| 216870_x_at | DLEU2 | deleted in lymphocytic leukemia, 2 | AF264787 | 8847 | -2.576247257 | 0.045362846 |
| 215629_s_at | DLEU2 /// DLEU2L | deleted in lymphocytic leukemia, 2 /// deleted in lymphocytic leukemia 2-like | AA905286 | 79469 /// 8847 | -2.49326754 | 0.043690779 |
| 217208_s_at | DLG1 | discs, large homolog 1 (Drosophila) | AL121981 | 1739 | -1.759921413 | 0.028533872 |
| 223553_s_at | DOK3 | docking protein 3 | BC004564 | 79930 | 1.721200493 | 0.037636668 |
| 238532_at | DPF3 | D4, zinc and double PHD fingers, family 3 | AI125562 | 8110 | -1.877470997 | 0.002016931 |
| 210611_s_at | DTNA | dystrobrevin, alpha | U26744 | 1837 | -2.31598428 | 0.004257029 |
| 210091_s_at | DTNA | dystrobrevin, alpha | U46745 | 1837 | -2.255107991 | 0.003752485 |
| 202971_s_at | DYRK2 | dual-specificity tyrosine-(Y)-phosphorylation regulated kinase 2 | NM_006482 | 8445 | -1.828893049 | 0.049269942 |
| 233814_at | EFNA5 | CDNA: FLJ22256 fis, clone HRC02860 | AK025909 | 1946 | 2.332092273 | 0.030973248 |
| 225827_at | EIF2C2 | eukaryotic translation initiation factor 2C, 2 | AI832074 | 27161 | -1.94928682 | 0.003389434 |
| 211776_s_at | EPB41L3 | erythrocyte membrane protein band 4.1-like 3 /// erythrocyte membrane protein band 4.1-like 3 | BC006141 | 23136 | -2.034309376 | 0.036177681 |
| 206710_s_at | EPB41L3 | erythrocyte membrane protein band 4.1-like 3 | NM_012307 | 23136 | -1.941707757 | 0.014763141 |
| 231576_at | ETNK1 | Ethanolamine kinase 1 | AA829940 | 55500 | 2.606614733 | 0.049137111 |
| 208963_x_at | FADS1 | fatty acid desaturase 1 | BG165833 | 3992 | -1.659158196 | 0.041814182 |
| 1555948_s_at | FAM120A | chromosome 9 open reading frame 10 | AU116818 | 23196 | -1.866934233 | 0.039203696 |
| 1554132_a_at | FAM190B | KIAA1128 | BC030528 | 54462 | -1.829285605 | 0.017344061 |
| 238785_at | FAM208A | Chromosome 3 open reading frame 63 | AI632091 | 23272 | -2.147786486 | 0.014284626 |
| 232063_x_at | FARSB | phenylalanine-tRNA synthetase-like, beta subunit | AW024617 | 10056 | -1.980083032 | 0.012421262 |
| 1554360_at | FCHSD2 | FCH and double SH3 domains 2 | BC010394 | 9873 | 1.75685633 | 0.00788161 |
| 210950_s_at | FDFT1 | farnesyl-diphosphate farnesyltransferase 1 | BC003573 | 2222 | -1.838311935 | 0.01956126 |
| 209209_s_at | FERMT2 | pleckstrin homology domain containing, family C (with FERM domain) member 1 | AW469573 | 10979 | -1.864310991 | 0.018838172 |
| 223263_s_at | FGFR1OP2 | FGFR1 oncogene partner 2 | AF161472 | 26127 | -1.834323201 | 0.020411908 |
| 210298_x_at | FHL1 | four and a half LIM domains 1 | AF098518 | 2273 | -1.92209437 | 0.032802071 |
| 214505_s_at | FHL1 | four and a half LIM domains 1 | AF220153 | 2273 | -1.798612808 | 0.043359823 |
| 201539_s_at | FHL1 | four and a half LIM domains 1 | U29538 | 2273 | -1.725202129 | 0.046590851 |
| 1554424_at | FIP1L1 | FIP1 like 1 (S. cerevisiae) | BC017724 | 81608 | -1.999743212 | 0.045450207 |
| 219871_at | FLJ13197 | hypothetical protein FLJ13197 | NM_024614 | 79667 | -2.170022355 | 0.010197348 |
| 211074_at | FOLR1 | folate receptor 1 (adult) /// folate receptor 1 (adult) | AF000381 | 2348 | 1.633347116 | 0.024637719 |
| 225464_at | FRMD6 | FERM domain containing 6 | N30138 | 122786 | -1.668405393 | 0.035338549 |
| 1554260_a_at | FRYL | furry homolog-like (Drosophila) | BC021803 | 23045 | -1.77910698 | 0.010001971 |
| 202593_s_at | GDE1 | membrane interacting protein of RGS16 | NM_016641 | 51573 | -1.811401119 | 0.049131098 |
| 229274_at | GNAS | GNAS complex locus | AI693143 | 2778 | -1.84663363 | 0.032746996 |
| 242224_at | GPATCH2 | G patch domain containing 2 | R40111 | 55105 | -2.412558458 | 0.010517146 |
| 236026_at | GPATCH2 | G patch domain containing 2 | AA160529 | 55105 | -2.213990351 | 0.007682682 |
| 212487_at | GPATCH8 | KIAA0553 | AI673812 | 23131 | -1.809982702 | 0.030753376 |
| 224835_at | GPCPD1 | hypothetical protein KIAA1434 | AL109935 | 56261 | -2.698347226 | 0.023537448 |
| 224826_at | GPCPD1 | hypothetical protein KIAA1434 | AK001947 | 56261 | -2.466381751 | 0.001960438 |
| 230492_s_at | GPCPD1 | hypothetical protein KIAA1434 | BE328402 | 56261 | -2.093095336 | 0.010428844 |
| 206972_s_at | GPR161 | G protein-coupled receptor 161 | NM_007369 | 23432 | -1.677344604 | 0.048201622 |
| 222830_at | GRHL1 | grainyhead-like 1 (Drosophila) | BE566136 | 29841 | -1.744792997 | 0.040390918 |
| 223758_s_at | GTF2H2 | general transcription factor IIH, polypeptide 2, 44kDa | BC005345 | 2966 | -1.70146844 | 0.018765906 |
| 215599_at | GUSBP3 | SMA4 /// region containing SMA4; hypothetical protein LOC153561 /// region containing hypothetical protein LOC153561; SMA4 /// SMA4 /// similar to Beta-glucuronidase precursor | X83300 | 11039 /// 643367 /// 643373 /// 652924 /// 653869 | -2.046515839 | 0.035754051 |
| 232975_at | HCG18 | HLA complex group 18 | AK023334 | 414777 | -1.805943961 | 0.005540678 |
| 205425_at | HIP1 | huntingtin interacting protein 1 | NM_005338 | 3092 | -1.754618773 | 0.018419137 |
| 206110_at | HIST1H3H | histone 1, H3h | NM_003536 | 8357 | -2.527855903 | 0.021319238 |
| 226651_at | HOMER1 | homer homolog 1 (Drosophila) | AW052119 | 9456 | -1.818415849 | 0.037799592 |
| 202854_at | HPRT1 | hypoxanthine phosphoribosyltransferase 1 (Lesch-Nyhan syndrome) | NM_000194 | 3251 | -1.914178966 | 0.030272041 |
| 211968_s_at | HSP90AA1 | heat shock protein 90kDa alpha (cytosolic), class A member 1 | AI962933 | 3320 | -1.952827586 | 0.010995564 |
| 208937_s_at | ID1 | inhibitor of DNA binding 1, dominant negative helix-loop-helix protein | D13889 | 3397 | 14.09902633 | 0.000233479 |
| 201565_s_at | ID2 | inhibitor of DNA binding 2, dominant negative helix-loop-helix protein | NM_002166 | 3398 | 1.887536491 | 0.026292719 |
| 201566_x_at | ID2 | inhibitor of DNA binding 2, dominant negative helix-loop-helix protein /// inhibitor of DNA binding 2B, dominant negative helix-loop-helix protein | D13891 | 3398 /// 84099 | 2.304964989 | 0.005782525 |
| 207826_s_at | ID3 | inhibitor of DNA binding 3, dominant negative helix-loop-helix protein | NM_002167 | 3399 | 15.30390572 | 0.000931033 |
| 208881_x_at | IDI1 | isopentenyl-diphosphate delta isomerase 1 | BC005247 | 3422 | -1.733252295 | 0.007417258 |
| 204615_x_at | IDI1 | isopentenyl-diphosphate delta isomerase 1 | NM_004508 | 3422 | -1.693236533 | 0.010836957 |
| 208931_s_at | ILF3 | interleukin enhancer binding factor 3, 90kDa | AF147209 | 3609 | -1.88012693 | 0.015481113 |
| 201627_s_at | INSIG1 | insulin induced gene 1 | NM_005542 | 3638 | -3.041683129 | 0.000145217 |
| 201625_s_at | INSIG1 | insulin induced gene 1 | BE300521 | 3638 | -2.930285408 | 0.001448747 |
| 201626_at | INSIG1 | insulin induced gene 1 | BG292233 | 3638 | -2.241108347 | 0.007455856 |
| 213446_s_at | IQGAP1 | IQ motif containing GTPase activating protein 1 | AI679073 | 8826 | -2.17712208 | 0.00442648 |
| 210840_s_at | IQGAP1 | IQ motif containing GTPase activating protein 1 | D29640 | 8826 | -1.777588546 | 0.002572664 |
| 238775_at | ITFG1 | Integrin alpha FG-GAP repeat containing 1 | AA088543 | 81533 | -1.68232172 | 0.017140781 |
| 211488_s_at | ITGB8 | integrin, beta 8 | BC002630 | 3696 | -2.154395692 | 5.69541E-05 |
| 209098_s_at | JAG1 | jagged 1 (Alagille syndrome) | U61276 | 182 | -1.904325263 | 0.048768203 |
| 238774_at | KANSL1 | KIAA1267 | AW960454 | 284058 | -2.430458349 | 0.035861146 |
| 229144_at | KAZ | Kazrin | AA989362 | 23254 | -1.915102949 | 0.021710787 |
| 223727_at | KCNIP2 | Kv channel interacting protein 2 | AL136722 | 30819 | 1.739200943 | 0.039479588 |
| 228414_at | KCNMA1 | potassium large conductance calcium-activated channel, subfamily M, alpha member 1 | AI832576 | 3778 | 1.955860775 | 0.007793237 |
| 212492_s_at | KDM4B | jumonji domain containing 2B | AW237172 | 23030 | -1.702140569 | 0.026388503 |
| 212634_at | KIAA0776 | KIAA0776 | AW298092 | 23376 | -1.83178552 | 0.004531114 |
| 215936_s_at | KIAA1033 | KIAA1033 | AK001657 | 23325 | -1.652328943 | 0.003241634 |
| 203849_s_at | KIF1A | kinesin family member 1A | BG473130 | 547 | -1.679380487 | 0.030328473 |
| 224606_at | KLF6 | Kruppel-like factor 6 | BG250721 | 1316 | -1.689986395 | 0.008752827 |
| 241790_at | KLHL7 | Kelch-like 7 (Drosophila) | T57946 | 55975 | -1.660411275 | 0.03421965 |
| 1554679_a_at | LAPTM4B | lysosomal associated protein transmembrane 4 beta | AF317417 | 55353 | -2.252141531 | 0.024831332 |
| 208767_s_at | LAPTM4B | lysosomal associated protein transmembrane 4 beta | AW149681 | 55353 | -1.81026299 | 0.019968382 |
| 223380_s_at | LATS2 | LATS, large tumor suppressor, homolog 2 (Drosophila) | AF207547 | 26524 | -1.628501731 | 0.005281477 |
| 202068_s_at | LDLR | low density lipoprotein receptor (familial hypercholesterolemia) | NM_000527 | 3949 | -2.385192259 | 0.033151968 |
| 1556997_a_at | LEPR | leptin receptor | BC036581 | 3953 | 2.046323381 | 0.014964757 |
| 231866_at | LNPEP | leucyl/cystinyl aminopeptidase | AA767440 | 4012 | -1.858346331 | 0.004874024 |
| 235443_at | LOC100131067 | Creatine kinase, mitochondrial 2 (sarcomeric) | BG284827 | 1160 | -1.993035862 | 0.009802508 |
| 243785_at | LOC100272217 | CDNA FLJ31475 fis, clone NT2NE2001598 | AW085489 |  | -2.579230838 | 0.000653626 |
| 230894_s_at | LOC100506728 | hypothetical LOC100506728 | BE672557 |  | 1.636069585 | 0.027120247 |
| 235014_at | LOC147727 | Hypothetical protein LOC147727 | BF345728 | 147727 | -2.162246127 | 0.006397242 |
| 229090_at | LOC220930 | hypothetical protein LOC220930 | AK026657 | 220930 | -1.783271712 | 0.011119756 |
| 230351_at | LOC283481 | hypothetical protein LOC283481 | AW070248 | 283481 | -1.787455727 | 0.000684999 |
| 243225_at | LOC283481 | hypothetical protein LOC283481 | AI042341 | 283481 | -1.715341079 | 0.006614302 |
| 230930_at | LOC338620 | hypothetical protein LOC338620 | AI435839 | 338620 | 1.690535856 | 0.020319246 |
| 238893_at | LOC338758 | hypothetical protein LOC338758 | AI377324 | 338758 | -1.779995117 | 0.041133995 |
| 239151_at | LOC399753 | hypothetical protein LOC399761 /// hypothetical protein LOC643564 | BG427809 | 399761 /// 643564 | -1.741715929 | 0.001045986 |
| 1552622_s_at | LOC441259 /// POLR2J2 | DNA directed RNA polymerase II polypeptide J-related gene /// similar to postmeiotic segregation increased 2-like 2 | BQ613856 | 246721 /// 441259 | -2.198617162 | 0.016247564 |
| 1558404_at | LOC644242 | hypothetical protein LOC644242 /// hypothetical protein LOC650429 /// hypothetical protein LOC650446 | BC015390 | 644242 /// 650429 /// 650446 | 3.207912947 | 0.001403093 |
| 239154_at | LOC646144 | Similar to tousled-like kinase 2 | AW962705 | 646144 | -1.661149198 | 0.011029504 |
| 239343_at | LOC728705 | CDNA clone IMAGE:4821804 | AW451176 |  | -1.694483491 | 0.002936227 |
| 238833_at | LOC729088 | Full-length cDNA clone CS0DC001YL20 of Neuroblastoma Cot 25-normalized of Homo sapiens (human) | AW139053 |  | -1.907971787 | 0.038992223 |
| 241471_at | LOC730236 | gb:AL520533 /DB_XREF=gi:12784026 /DB_XREF=AL520533 /CLONE=CS0DB006YB20 (3 prime) /FEA=EST /CNT=5 /TID=Hs.212491.0 /TIER=ConsEnd /STK=4 /UG=Hs.212491 /UG_TITLE=ESTs | AL520533 |  | -1.638581785 | 0.030285585 |
| 230738_at | LOC730631 | CDNA clone IMAGE:6342029 | BE858063 |  | -1.879330922 | 0.013087274 |
| 242778_at | LPXN | leupaxin | AA250935 | 9404 | -1.684181459 | 0.027610964 |
| 1568634_a_at | LRRC66 | similar to hypothetical protein MGC38937 | BU620718 | 339977 | 1.669120512 | 0.027310621 |
| 214577_at | MAP1B | microtubule-associated protein 1B | BG164365 | 4131 | -2.069784686 | 0.022540926 |
| 202654_x_at | MARCH7 | membrane-associated ring finger (C3HC4) 7 | NM_022826 | 64844 | -1.744631133 | 0.043413393 |
| 1558093_s_at | MATR3 | matrin 3 | BI832461 | 9782 | -1.843499562 | 0.027856759 |
| 232740_at | MCM3APAS | MCM3 minichromosome maintenance deficient 3 (S. cerevisiae) associated protein antisense | BC002458 | 114044 | -1.759894486 | 0.009640178 |
| 220459_at | MCM3APAS | MCM3 minichromosome maintenance deficient 3 (S. cerevisiae) associated protein antisense | NM_018118 | 114044 | -1.758973174 | 0.0297427 |
| 225160_x_at | MDM2 | hypothetical protein MGC5370 | AI952357 | 84825 | 1.769753572 | 0.03741392 |
| 225253_s_at | METTL2A /// METTL2B | methyltransferase like 2B /// methyltransferase like 2A | AI632244 | 339175 /// 55798 | -1.665061212 | 0.011684093 |
| 232102_at | METTL6 | Methyltransferase like 6 | AK027185 | 131965 | 1.946782973 | 0.01019381 |
| 235409_at | MGA | MAX gene associated | AU149225 | 23269 | -1.638978329 | 0.021980987 |
| 1553881_at | MGC16142 | hypothetical protein MGC16142 | NM_032763 | 84849 | 1.789333731 | 0.00135192 |
| 1554450_s_at | MIER3 | mesoderm induction early response 1, family member 3 | BC041348 | 166968 | -1.822930516 | 0.027502138 |
| 212079_s_at | MLL | myeloid/lymphoid or mixed-lineage leukemia (trithorax homolog, Drosophila) | AA715041 | 4297 | -1.982209089 | 0.012598642 |
| 212076_at | MLL | myeloid/lymphoid or mixed-lineage leukemia (trithorax homolog, Drosophila) | AI701430 | 4297 | -1.763167687 | 0.005667515 |
| 222413_s_at | MLL3 | myeloid/lymphoid or mixed-lineage leukemia 3 | AW137099 | 58508 | -1.850475106 | 0.031187688 |
| 1563321_s_at | MLLT10 | myeloid/lymphoid or mixed-lineage leukemia (trithorax homolog, Drosophila); translocated to, 10 | AF272384 | 8028 | -1.836628026 | 0.037124 |
| 240271_at | MTMR3 | Myotubularin related protein 3 | AL038191 | 8897 | -2.085020294 | 0.012094532 |
| 242996_at | MTRF1 | mitochondrial translational release factor 1 | AI341686 | 9617 | -1.6986322 | 0.030121224 |
| 212095_s_at | MTUS1 | mitochondrial tumor suppressor 1 | BE552421 | 57509 | -2.118827768 | 0.01977918 |
| 212093_s_at | MTUS1 | mitochondrial tumor suppressor 1 | AI695017 | 57509 | -1.977176391 | 0.029197178 |
| 202431_s_at | MYC | v-myc myelocytomatosis viral oncogene homolog (avian) | NM_002467 | 4609 | -2.022766607 | 0.009230382 |
| 236718_at | MYO10 | Transcribed locus, weakly similar to NP_055301.1 neuronal thread protein AD7c-NTP [Homo sapiens] | AI278445 | 4651 | 2.092455719 | 0.043404038 |
| 203215_s_at | MYO6 | myosin VI | AA877789 | 4646 | -1.810068598 | 0.031474527 |
| 208047_s_at | NAB1 | NGFI-A binding protein 1 (EGR1 binding protein 1) | NM_005966 | 4664 | -2.457622004 | 0.015730417 |
| 224771_at | NAV1 | neuron navigator 1 | AI937060 | 89796 | -2.198219233 | 0.002908504 |
| 1554106_at | NBEAL1 | neurobeachin-like 1 /// amyotrophic lateral sclerosis 2 (juvenile) chromosome region, candidate 16 | AB053318 | 130029 /// 65065 | -1.694065575 | 0.013552486 |
| 1552309_a_at | NEXN | nexilin (F actin binding protein) | NM_144573 | 91624 | -1.69430116 | 0.006718461 |
| 210631_at | NF1 | neurofibromin 1 (neurofibromatosis, von Recklinghausen disease, Watson disease) | D42072 | 4763 | -1.69269488 | 0.031586767 |
| 230791_at | NFIB | Nuclear factor I/B | AU146924 | 4781 | -2.014854681 | 0.029979269 |
| 211466_at | NFIB | nuclear factor I/B | U70862 | 4781 | -1.911638206 | 0.03824947 |
| 241797_at | NFIX | Nuclear factor I/X (CCAAT-binding transcription factor) | AI904095 | 4784 | -1.789704644 | 0.025539731 |
| 204108_at | NFYA | nuclear transcription factor Y, alpha | AL031778 | 4800 | -1.634268361 | 0.0098653 |
| 244704_at | NFYB | nuclear transcription factor Y, beta | AW083948 | 4801 | -2.00720883 | 0.026228113 |
| 1556568_a_at | NLK | Nemo-like kinase | N46436 | 51701 | -1.797940346 | 0.032012332 |
| 222590_s_at | NLK | nemo-like kinase | AF180819 | 51701 | -1.680421429 | 0.027136076 |
| 203964_at | NMI | N-myc (and STAT) interactor | NM_004688 | 9111 | -1.708114968 | 0.037894222 |
| 231798_at | NOG | Noggin | AL575177 | 9241 | 3.186858421 | 7.90906E-05 |
| 209750_at | NR1D2 | nuclear receptor subfamily 1, group D, member 2 | N32859 | 9975 | -1.993298716 | 0.018807604 |
| 214962_s_at | NUP160 | nucleoporin 160kDa | AK026236 | 23279 | -1.708958135 | 0.041717265 |
| 1564494_s_at | P4HB | procollagen-proline, 2-oxoglutarate 4-dioxygenase (proline 4-hydroxylase), beta polypeptide | AK075503 | 5034 | -1.705622763 | 0.035668346 |
| 200906_s_at | PALLD | palladin, cytoskeletal associated protein | AK025843 | 23022 | -1.925304559 | 0.025453554 |
| 200907_s_at | PALLD | palladin, cytoskeletal associated protein | AU157932 | 23022 | -1.809327284 | 0.013654016 |
| 238706_at | PAPD4 | PAP associated domain containing 4 | BG168850 | 167153 | -2.726937986 | 0.025624019 |
| 218543_s_at | PARP12 | poly (ADP-ribose) polymerase family, member 12 | NM_022750 | 64761 | -1.967060397 | 0.013525283 |
| 210170_at | PDLIM3 | PDZ and LIM domain 3 | BC001017 | 27295 | -1.916366151 | 0.017507993 |
| 213984_at | PDS5A | SCC-112 protein | AW991219 | 23244 | -1.809498504 | 0.049353698 |
| 213983_s_at | PDS5A | SCC-112 protein | AW991219 | 23244 | -1.684720084 | 0.003447038 |
| 225366_at | PGM2 | phosphoglucomutase 2 | AI652855 | 55276 | -1.648427385 | 0.003469392 |
| 1554472_a_at | PHF20L1 | PHD finger protein 20-like 1 | BC015211 | 51105 | -2.203236087 | 0.031976332 |
| 205281_s_at | PIGA | phosphatidylinositol glycan, class A (paroxysmal nocturnal hemoglobinuria) /// phosphatidylinositol glycan, class A (paroxysmal nocturnal hemoglobinuria) | NM_002641 | 5277 | -1.637704868 | 0.028579084 |
| 229713_at | PIP4K2A | CDNA FLJ13267 fis, clone OVARC1000964 | AW665227 | 5305 | 1.77195097 | 0.008354441 |
| 224883_at | PLDN | pallidin homolog (mouse) | AI344311 | 26258 | -1.698948481 | 0.004415876 |
| 219024_at | PLEKHA1 | pleckstrin homology domain containing, family A (phosphoinositide binding specific) member 1 | NM_021622 | 59338 | -1.678715135 | 0.023340746 |
| 227148_at | PLEKHH2 | pleckstrin homology domain containing, family H (with MyTH4 domain) member 2 | AI913749 | 130271 | -1.928108475 | 0.004424496 |
| 1552621_at | POLR2J2 | DNA directed RNA polymerase II polypeptide J-related gene | BQ613856 | 246721 | -1.905106156 | 0.027018413 |
| 205277_at | PRDM2 | PR domain containing 2, with ZNF domain | NM_012231 | 7799 | 1.961336114 | 0.004226338 |
| 211090_s_at | PRPF4B | PRP4 pre-mRNA processing factor 4 homolog B (yeast) /// PRP4 pre-mRNA processing factor 4 homolog B (yeast) | Z25435 | 8899 | -1.739816144 | 0.028482373 |
| 226574_at | PSPC1 | similar to paraspeckle protein 1 /// similar to paraspeckle protein 1 | AI872384 | 642395 /// 649009 | -1.824020321 | 0.006109301 |
| 235484_at | PTAR1 | protein prenyltransferase alpha subunit repeat containing 1 | BE892889 | 375743 | -1.812329467 | 0.002091466 |
| 1559529_at | PTK2 | PTK2 protein tyrosine kinase 2 | BC043202 | 5747 | -1.721748706 | 0.032083943 |
| 227741_at | PTPLB | protein tyrosine phosphatase-like (proline instead of catalytic arginine), member b | AI813654 | 201562 | -1.685738119 | 0.026692282 |
| 209896_s_at | PTPN11 | protein tyrosine phosphatase, non-receptor type 11 (Noonan syndrome 1) | AF119855 | 5781 | -1.906058339 | 0.005711035 |
| 242188_at | PTPRG | Protein tyrosine phosphatase, receptor type, G | AI743332 | 5793 | -2.020055786 | 0.003084731 |
| 218700_s_at | RAB7L1 | RAB7, member RAS oncogene family-like 1 | BC002585 | 8934 | -1.876154424 | 0.004770692 |
| 201711_x_at | RANBP2 | RAN binding protein 2 | AI681120 | 5903 | -1.809225198 | 0.001593024 |
| 1556808_at | RAP2A | RAP2A, member of RAS oncogene family | BQ007743 | 5911 | 1.644029026 | 0.015046581 |
| 230466_s_at | RASSF3 | Ras association (RalGDS/AF-6) domain family 3 | AI092770 | 283349 | -2.238253922 | 0.033509096 |
| 217301_x_at | RBBP4 | retinoblastoma binding protein 4 | X71810 | 5928 | -1.76772019 | 0.034454294 |
| 204128_s_at | RFC3 | replication factor C (activator 1) 3, 38kDa | NM_002915 | 5983 | -2.396564293 | 0.036047364 |
| 230243_at | RG9MTD2 | RNA (guanine-9-) methyltransferase domain containing 2 | BE671949 | 93587 | -1.772965124 | 0.015295056 |
| 236726_at | RGS6 | CDNA FLJ43552 fis, clone PROST2017972 | H41121 | 9628 | 1.841597094 | 0.017869056 |
| 1555814_a_at | RHOA | ras homolog gene family, member A | AF498970 | 387 | -2.041083189 | 0.043584747 |
| 212099_at | RHOB | ras homolog gene family, member B | AI263909 | 388 | -1.710678786 | 0.041214417 |
| 216048_s_at | RHOBTB3 | Rho-related BTB domain containing 3 | AK023621 | 22836 | -2.555723678 | 0.014456648 |
| 235489_at | RHOJ | ras homolog gene family, member J | AI583530 | 57381 | -1.81655934 | 0.031528524 |
| 214449_s_at | RHOQ | ras homolog gene family, member Q | NM_012249 | 23433 | -1.783025499 | 0.030266089 |
| 220985_s_at | RNF170 | ring finger protein 170 /// ring finger protein 170 | NM_030954 | 81790 | -1.64861453 | 0.014073847 |
| 244418_at | RTN3 | Reticulon 3 | AA223929 | 10313 | -1.774857206 | 0.028738291 |
| 243031_at | RTN4 | Reticulon 4 | N90377 | 57142 | -2.182241807 | 0.034860558 |
| 209146_at | SC4MOL | sterol-C4-methyl oxidase-like | AV704962 | 6307 | -1.90001701 | 0.009861658 |
| 206668_s_at | SCAMP1 | secretory carrier membrane protein 1 | NM_004866 | 9522 | -1.755021975 | 0.007486658 |
| 214298_x_at | SEPT6 | septin 6 | AL568374 | 23157 | -1.823081706 | 0.033107069 |
| 212190_at | SERPINE2 | serpin peptidase inhibitor, clade E (nexin, plasminogen activator inhibitor type 1), member 2 | AL541302 | 5270 | -1.812094054 | 0.016088606 |
| 220200_s_at | SETD8 | SET domain containing (lysine methyltransferase) 8 | NM_020382 | 387893 | -1.764420468 | 0.027638971 |
| 240709_at | SEZ6L | seizure related 6 homolog (mouse)-like | AW204757 | 23544 | -2.517018257 | 0.006859629 |
| 214305_s_at | SF3B1 | splicing factor 3b, subunit 1, 155kDa | AW003030 | 23451 | -1.684451097 | 0.012046063 |
| 201070_x_at | SF3B1 | splicing factor 3b, subunit 1, 155kDa | AI739389 | 23451 | -1.629646749 | 0.016702075 |
| 238794_at | SFR1 | SWI5-dependent recombination repair 1 | N23586 | 119392 | -2.425734201 | 0.005777535 |
| 201129_at | SFRS7 | splicing factor, arginine/serine-rich 7, 35kDa | NM_006276 | 6432 | -1.741256195 | 0.005668763 |
| 221268_s_at | SGPP1 | sphingosine-1-phosphate phosphatase 1 /// sphingosine-1-phosphate phosphatase 1 | NM_030791 | 81537 | -1.863138166 | 0.049407019 |
| 202236_s_at | SLC16A1 | solute carrier family 16 (monocarboxylic acid transporters), member 1 | NM_003051 | 6566 | -1.775163024 | 0.014188959 |
| 230748_at | SLC16A6 | solute carrier family 16 (monocarboxylic acid transporters), member 6 | AI873273 | 9120 | -4.907831438 | 0.022305855 |
| 223296_at | SLC25A33 | PNC1 protein | BC004991 | 84275 | -1.855506943 | 0.021939681 |
| 1554148_a_at | SLC33A1 | solute carrier family 33 (acetyl-CoA transporter), member 1 | BC014416 | 9197 | -1.65595865 | 0.007478195 |
| 213624_at | SMPDL3A | sphingomyelin phosphodiesterase, acid-like 3A | AA873600 | 10924 | -1.625111504 | 0.009955844 |
| 233713_at | SMYD2 | SET and MYND domain containing 2 | AK022181 | 56950 | -2.190492519 | 0.033054084 |
| 1560741_at | SNRPN | small nuclear ribonucleoprotein polypeptide N | AL832250 | 6638 | -1.670227027 | 0.027000723 |
| 201085_s_at | SON | SON DNA binding protein | AA664291 | 6651 | -1.727816763 | 0.006909467 |
| 201996_s_at | SPEN | spen homolog, transcriptional regulator (Drosophila) | AL524033 | 23013 | -1.975097124 | 0.038668464 |
| 236127_at | SPEN | spen homolog, transcriptional regulator (Drosophila) | AW082221 | 23013 | -1.650489185 | 0.014913174 |
| 1559517_a_at | SPIRE1 | spire homolog 1 (Drosophila) | AL833817 | 56907 | -1.67254004 | 0.028676614 |
| 204011_at | SPRY2 | sprouty homolog 2 (Drosophila) | NM_005842 | 10253 | -1.713367274 | 0.037690181 |
| 1557352_at | SQLE | Squalene epoxidase | BU754109 | 6713 | -1.766864484 | 0.045619767 |
| 209218_at | SQLE | squalene epoxidase | AF098865 | 6713 | -1.689538148 | 0.011708097 |
| 213577_at | SQLE | squalene epoxidase | AA639705 | 6713 | -1.656049812 | 0.031743591 |
| 236838_at | SRCIN1 | Homo sapiens, clone IMAGE:4940467, mRNA | H11609 | 80725 | 1.697661229 | 0.041920813 |
| 208610_s_at | SRRM2 | serine/arginine repetitive matrix 2 | AI655799 | 23524 | -1.778572523 | 0.032435172 |
| 1569788_at | ST8SIA1 | ST8 alpha-N-acetyl-neuraminide alpha-2,8-sialyltransferase 1 | BC039019 | 6489 | -2.703212636 | 0.028809979 |
| 208992_s_at | STAT3 | signal transducer and activator of transcription 3 (acute-phase response factor) | BC000627 | 6774 | -1.639628203 | 0.035353948 |
| 227179_at | STAU2 | staufen, RNA binding protein, homolog 2 (Drosophila) | AK002152 | 27067 | 1.708293296 | 0.003991586 |
| 219262_at | SUV39H2 | suppressor of variegation 3-9 homolog 2 (Drosophila) | NM_024670 | 79723 | -1.81817099 | 0.03883684 |
| 242646_at | SUV420H1 | Suppressor of variegation 4-20 homolog 1 (Drosophila) | AA702946 | 51111 | -1.779712135 | 0.040539554 |
| 230285_at | SVIP | hypothetical protein DKFZp313A2432 | BF447829 | 258010 | -2.427433549 | 0.007061538 |
| 222634_s_at | TBL1XR1 | transducin (beta)-like 1X-linked receptor 1 | AF314544 | 79718 | -1.83895992 | 0.03039838 |
| 216511_s_at | TCF7L2 | transcription factor 7-like 2 (T-cell specific, HMG-box) | AJ270770 | 6934 | -1.736547439 | 0.041473942 |
| 204731_at | TGFBR3 | transforming growth factor, beta receptor III (betaglycan, 300kDa) | NM_003243 | 7049 | -1.915172651 | 0.003711463 |
| 220702_at | TLK1 | tousled-like kinase 1 | NM_018616 | 9874 | -2.363581516 | 0.002642589 |
| 218897_at | TMEM177 | hypothetical protein MGC10993 | NM_030577 | 80775 | -1.768839896 | 0.011531529 |
| 223784_at | TMEM27 | transmembrane protein 27 | AF229179 | 57393 | -2.006594218 | 0.032395255 |
| 238468_at | TNRC6B | trinucleotide repeat containing 6B | AA214704 | 23112 | -1.969344239 | 0.006348821 |
| 1557227_s_at | TPR | translocated promoter region (to activated MET oncogene) | AW235355 | 7175 | -1.719715913 | 0.034066567 |
| 242473_at | TRAF4 | TNF receptor-associated factor 4 | BF000155 | 9618 | -1.851032489 | 0.03838354 |
| 208178_x_at | TRIO | triple functional domain (PTPRF interacting) | NM_007118 | 7204 | -1.654549492 | 0.001960067 |
| 224218_s_at | TRPS1 | trichorhinophalangeal syndrome I | AF264784 | 7227 | -2.082602927 | 0.026685762 |
| 235315_at | TSC22D1 | TSC22 domain family, member 1 | AI809519 | 8848 | -2.553600864 | 0.035135921 |
| 231853_at | TUBD1 | tubulin, delta 1 | AK022771 | 51174 | 1.847809 | 0.010922002 |
| 233303_at | UBE2D3 | Ubiquitin-conjugating enzyme E2D 3 (UBC4/5 homolog, yeast) | AL110175 | 7323 | -2.124899816 | 0.014166058 |
| 218521_s_at | UBE2W | ubiquitin-conjugating enzyme E2W (putative) | NM_018299 | 55284 | -1.811493477 | 0.038110368 |
| 235003_at | UHMK1 | U2AF homology motif (UHM) kinase 1 | AI249980 | 127933 | -1.6828172 | 0.02418757 |
| 202412_s_at | USP1 | ubiquitin specific peptidase 1 | AW499935 | 7398 | -1.680636678 | 0.044327002 |
| 224563_at | WASF2 | WAS protein family, member 2 | BG338758 | 10163 | -1.707765713 | 0.034031578 |
| 210935_s_at | WDR1 | WD repeat domain 1 | AF274954 | 9948 | -1.683273866 | 0.045847957 |
| 212638_s_at | WWP1 | WW domain containing E3 ubiquitin protein ligase 1 | BF131791 | 11059 | -1.763292017 | 0.009444824 |
| 235927_at | XPO1 | gb:BE350122 /DB_XREF=gi:9261975 /DB_XREF=ht09f03.x1 /CLONE=IMAGE:3146237 /FEA=EST /CNT=10 /TID=Hs.157367.0 /TIER=ConsEnd /STK=1 /UG=Hs.157367 /UG_TITLE=ESTs | BE350122 | 7514 | -2.436503048 | 0.017665213 |
| 234032_at | ZCCHC7 | Zinc finger, CCHC domain containing 7 | AF119847 | 84186 | -2.382129546 | 0.01983795 |
| 241906_at | ZFN708 | Zinc finger protein 708 (KOX8) | BF001773 | 7562 | -2.137398173 | 0.016780347 |
| 206652_at | ZMYM5 | zinc finger, MYM-type 5 | NM_016384 | 9205 | -1.822927287 | 0.039910716 |
| 1552634_a_at | ZNF101 | zinc finger protein 101 | NM_033204 | 94039 | -1.915571955 | 0.012020026 |
| 1552947_x_at | ZNF114 | zinc finger protein 114 | NM_153608 | 163071 | -1.924994581 | 0.001001386 |
| 1552946_at | ZNF114 | zinc finger protein 114 | NM_153608 | 163071 | -1.786974701 | 0.004529122 |
| 241738_at | ZNF250 | Zinc finger protein 250 | AA478429 | 58500 | 1.671361088 | 0.044974553 |
| 1559449_a_at | ZNF254 | Zinc finger protein 539 | BF679633 | 399655 | -1.68579045 | 0.021408856 |
| 220055_at | ZNF287 | zinc finger protein 287 | NM_020653 | 57336 | -1.673176557 | 0.007593707 |
| 232753_at | ZNF346 | Zinc finger protein 346 | AU147613 | 23567 | 1.657513751 | 0.014103171 |
| 215359_x_at | ZNF44 | zinc finger protein 44 | AI758888 | 51710 | -1.922096352 | 0.027698238 |
| 1553192_at | ZNF441 | zinc finger protein 441 | NM_152355 | 126068 | -3.6007825 | 0.011804551 |
| 208081_s_at | ZNF442 | zinc finger protein 442 /// zinc finger protein 442 | NM_030824 | 79973 | -2.429482907 | 0.010330503 |
| 215307_at | ZNF529 | Zinc finger protein 529 | AL109722 | 57711 | -1.633579894 | 0.02519675 |
| 211721_s_at | ZNF551 | zinc finger protein 551 /// zinc finger protein 551 | BC005868 | 90233 | -1.678825575 | 0.02575632 |
| 1554628_at | ZNF57 | zinc finger protein 57 | BC028974 | 126295 | -2.301877219 | 0.022613098 |
| 1557270_at | ZNF69 | Zinc finger protein 69 | AA632049 | 7620 | -2.040713848 | 0.010002723 |
| 223590_at | ZNF700 | zinc finger protein 700 | AL136732 | 90592 | -1.670529861 | 0.029663406 |
| 1558755_x_at | ZNF763 | zinc finger protein 440 like | AA484731 | 284390 | -3.698682615 | 0.014376434 |
| 1558754_at | ZNF763 | zinc finger protein 440 like | AA484731 | 284390 | -3.497338184 | 0.042226781 |
| 244552_at | ZNF788 | FLJ46419 protein /// hypothetical protein LOC642089 | AI190287 | 388507 /// 642089 | -2.386313488 | 0.003819345 |
| 231902_at | ZNF827 | Hypothetical protein LOC152485 | R34396 | 152485 | -1.649880064 | 0.010090312 |
| 228346_at | ZNF844 | Hypothetical protein FLJ14959 | H47630 | 284391 | -1.835849048 | 0.021962254 |
| 1569241_a_at | ZNF93 | Zinc finger protein 93 | BC020837 | 81931 | -3.529895779 | 0.008485564 |
| 218349_s_at | ZWILCH | Zwilch, kinetochore associated, homolog (Drosophila) | NM_017975 | 55055 | -1.907382496 | 0.044159834 |

HD-HAd 5d

| **Probe Set ID** | **Gene Symbol** | **Gene Title** | **Accession number** | **EntrezGene** | **Fold Change** | **PValue** |
| --- | --- | --- | --- | --- | --- | --- |
| 240467_at | --- | gb:AA947873 /DB_XREF=gi:3109126 /DB_XREF=ok21h03.s1 /CLONE=IMAGE:1508501 /FEA=EST /CNT=4 /TID=Hs.134501.0 /TIER=ConsEnd /STK=4 /UG=Hs.134501 /UG_TITLE=ESTs | AA947873 |  | -1.858574007 | 0.044105669 |
| 1558236_at | --- | gb:BC014318.1 /DB_XREF=gi:15680014 /TID=Hs2.298058.1 /CNT=16 /FEA=mRNA /TIER=ConsEnd /STK=0 /UG=Hs.298058 /UG_TITLE=Homo sapiens, clone IMAGE:3684608, mRNA /DEF=Homo sapiens, clone IMAGE:3684608, mRNA. | BC014318 |  | -1.760074816 | 0.003296347 |
| 240020_at | --- | Transcribed locus, moderately similar to NP_055301.1 neuronal thread protein AD7c-NTP [Homo sapiens] | BF059537 |  | -1.694253398 | 0.025136707 |
| 1554878_a_at | ABCD3 | ATP-binding cassette, sub-family D (ALD), member 3 | BC009712 | 5825 | -2.099410924 | 0.030596785 |
| 208637_x_at | ACTN1 | actinin, alpha 1 | BC003576 | 87 | 1.762631868 | 0.015632917 |
| 200601_at | ACTN4 | actinin, alpha 4 | U48734 | 81 | 1.857004748 | 0.003635425 |
| 1555487_a_at | ACTR3B /// LOC100290215 | ARP3 actin-related protein 3 homolog B (yeast) /// similar to actin-related protein 3-beta | BC015207 | 57180 /// 648792 | 1.734250138 | 0.016664827 |
| 226997_at | ADAMTS12 | CDNA FLJ10196 fis, clone HEMBA1004776 | W74476 | 81792 | 1.679941549 | 0.039188957 |
| 215535_s_at | AGPAT1 | 1-acylglycerol-3-phosphate O-acyltransferase 1 (lysophosphatidic acid acyltransferase, alpha) | AF007145 | 10554 | 1.670707114 | 0.012326602 |
| 32836_at | AGPAT1 | 1-acylglycerol-3-phosphate O-acyltransferase 1 (lysophosphatidic acid acyltransferase, alpha) | U56417 | 10554 | 1.674394502 | 0.011135952 |
| 217419_x_at | AGRN | agrin | AK021586 | 375790 | 1.636461656 | 0.005660774 |
| 207163_s_at | AKT1 | v-akt murine thymoma viral oncogene homolog 1 | NM_005163 | 207 | 1.722699618 | 0.013724169 |
| 224982_at | AKT1S1 | AKT1 substrate 1 (proline-rich) | BE790884 | 84335 | 1.72815867 | 0.021873212 |
| 226663_at | ANKRD10 | ankyrin repeat domain 10 | BE670056 | 55608 | -1.64907385 | 0.011590399 |
| 200613_at | AP2M1 | adaptor-related protein complex 2, mu 1 subunit | NM_004068 | 1173 | 1.779345328 | 0.005728229 |
| 205678_at | AP3B2 | adaptor-related protein complex 3, beta 2 subunit | NM_004644 | 8120 | 1.625308108 | 0.028551535 |
| 233078_at | API5 | apoptosis inhibitor 5 | AK021649 | 8539 | -1.711998135 | 0.006467138 |
| 211110_s_at | AR | androgen receptor (dihydrotestosterone receptor; testicular feminization; spinal and bulbar muscular atrophy; Kennedy disease) | AF162704 | 367 | 1.957929667 | 0.006697595 |
| 210649_s_at | ARID1A | AT rich interactive domain 1A (SWI- like) | AF231056 | 8289 | 2.306904443 | 0.017253636 |
| 204966_at | BAI2 | brain-specific angiogenesis inhibitor 2 | NM_001703 | 576 | 1.843214594 | 0.009844933 |
| 210125_s_at | BANF1 | barrier to autointegration factor 1 | AF044773 | 8815 | 1.99194835 | 0.016059619 |
| 200041_s_at | BAT1 | HLA-B associated transcript 1 /// HLA-B associated transcript 1 | NM_004640 | 7919 | 2.12477385 | 0.008853034 |
| 212081_x_at | BAT2 | HLA-B associated transcript 2 | AF129756 | 7916 | 1.761644639 | 0.0017971 |
| 214052_x_at | BAT2L2 | BAT2 domain containing 1 | AW301305 | 23215 | -1.824152222 | 0.005939664 |
| 205363_at | BBOX1 | butyrobetaine (gamma), 2-oxoglutarate dioxygenase (gamma-butyrobetaine hydroxylase) 1 | NM_003986 | 8424 | -1.961662216 | 0.027755222 |
| 210653_s_at | BCKDHB | branched chain keto acid dehydrogenase E1, beta polypeptide (maple syrup urine disease) | M55575 | 594 | -1.964893487 | 0.027436895 |
| 206956_at | BGLAP | bone gamma-carboxyglutamate (gla) protein (osteocalcin) | NM_000711 | 632 | 1.716160498 | 0.020824687 |
| 223349_s_at | BOK | BCL2-related ovarian killer | BE614255 | 666 | 1.902146738 | 0.006021479 |
| 238545_at | BRD7 | Bromodomain containing 7 | AA214369 | 29117 | -1.756707764 | 0.04274548 |
| 220987_s_at | C11orf17 /// NUAK2 | chromosome 11 open reading frame 17 /// chromosome 11 open reading frame 17 /// NUAK family, SNF1-like kinase, 2 /// NUAK family, SNF1-like kinase, 2 | NM_030952 | 56672 /// 81788 | 1.696150368 | 0.040476806 |
| 207996_s_at | C18orf1 | chromosome 18 open reading frame 1 | NM_004338 | 753 | -1.812618014 | 0.044557288 |
| 221764_at | C19orf22 | chromosome 19 open reading frame 22 | AL574186 | 91300 | 1.706749793 | 0.027999433 |
| 225473_at | C20orf117 | chromosome 20 open reading frame 117 | BE044516 | 140710 | 1.89832362 | 0.030878049 |
| 204480_s_at | C9orf16 | chromosome 9 open reading frame 16 | NM_024112 | 79095 | 1.912731613 | 0.002189292 |
| 200935_at | CALR | calreticulin | NM_004343 | 811 | 1.764412564 | 0.027261501 |
| 205166_at | CAPN5 | calpain 5 | NM_004055 | 726 | 1.724271947 | 0.00053011 |
| 200001_at | CAPNS1 | calpain, small subunit 1 /// calpain, small subunit 1 | NM_001749 | 826 | 1.642366267 | 0.015019432 |
| 206724_at | CBX4 | chromobox homolog 4 (Pc class homolog, Drosophila) | NM_003655 | 8535 | 3.015109205 | 0.018421341 |
| 225454_at | CCDC124 | hypothetical protein BC013949 | AW248770 | 115098 | 1.62538375 | 0.01976938 |
| 208824_x_at | CDK16 | PCTAIRE protein kinase 1 | BC001048 | 5127 | 1.88341664 | 0.011521543 |
| 207239_s_at | CDK16 | PCTAIRE protein kinase 1 | NM_006201 | 5127 | 2.069605025 | 0.009321609 |
| 202284_s_at | CDKN1A | cyclin-dependent kinase inhibitor 1A (p21, Cip1) | NM_000389 | 1026 | 2.025645831 | 0.005582384 |
| 1555730_a_at | CFL1 | cofilin 1 (non-muscle) | D00682 | 1072 | 1.987204817 | 0.032321118 |
| 211031_s_at | CLIP2 | cytoplasmic linker 2 /// cytoplasmic linker 2 | BC006259 | 7461 | 1.87086611 | 0.007283816 |
| 211136_s_at | CLPTM1 | cleft lip and palate associated transmembrane protein 1 | BC004865 | 1209 | 2.01874228 | 0.016754136 |
| 203239_s_at | CNOT3 | CCR4-NOT transcription complex, subunit 3 | NM_014516 | 4849 | 2.080415584 | 0.009690875 |
| 1560225_at | CNR1 | Cannabinoid receptor 1 (brain) | AI434253 | 1268 | -2.204722001 | 0.036847957 |
| 201264_at | COPE | coatomer protein complex, subunit epsilon | NM_007263 | 11316 | 1.689497048 | 0.026402126 |
| 223500_at | CPLX1 | complexin 1 | BC002471 | 10815 | 3.440777373 | 0.00980546 |
| 209283_at | CRYAB | crystallin, alpha B | AF007162 | 1410 | 1.942581216 | 0.035802923 |
| 227069_at | CUX1 | cut-like homeobox 1 | AA806989 | 1523 | 2.006944141 | 0.019881921 |
| 218892_at | DCHS1 | dachsous 1 (Drosophila) | NM_024542 | 8642 | 1.724148582 | 0.009184145 |
| 201082_s_at | DCTN1 | dynactin 1 (p150, glued homolog, Drosophila) | NM_004082 | 1639 | 2.086041544 | 0.023854205 |
| 212649_at | DHX29 | gb:AL079292.1 /DB_XREF=gi:5102732 /FEA=mRNA /CNT=133 /TID=Hs.95665.0 /TIER=ConsEnd /STK=0 /UG=Hs.95665 /LL=54505 /UG_GENE=LOC54505 /DEF=Homo sapiens mRNA full length insert cDNA clone EUROIMAGE 48814. /PROD=hypothetical protein, similar to (AC007017)putat | AL079292 | 54505 | -1.803303997 | 0.010583542 |
| 33768_at | DMWD | dystrophia myotonica-containing WD repeat motif | L19267 | 1762 | 1.63326621 | 0.013721278 |
| 206782_s_at | DNAJC4 | DnaJ (Hsp40) homolog, subfamily C, member 4 | NM_005528 | 3338 | 1.673467705 | 0.002362163 |
| 55583_at | DOCK6 | dedicator of cytokinesis 6 | AI198543 | 57572 | 1.801483341 | 0.030048955 |
| 201843_s_at | EFEMP1 | EGF-containing fibulin-like extracellular matrix protein 1 | NM_004105 | 2202 | 2.161527437 | 0.02047503 |
| 201842_s_at | EFEMP1 | EGF-containing fibulin-like extracellular matrix protein 1 | AI826799 | 2202 | 2.45274379 | 0.033733359 |
| 45297_at | EHD2 | EH-domain containing 2 | AI417917 | 30846 | 1.746311307 | 0.008064931 |
| 202326_at | EHMT2 | euchromatic histone-lysine N-methyltransferase 2 | NM_006709 | 10919 | 1.774716647 | 0.041078722 |
| 214313_s_at | EIF5B | Eukaryotic translation initiation factor 5B | BE138647 | 9669 | -1.662084734 | 0.028585295 |
| 203729_at | EMP3 | epithelial membrane protein 3 | NM_001425 | 2014 | 2.209709031 | 0.000795517 |
| 201313_at | ENO2 | enolase 2 (gamma, neuronal) | NM_001975 | 2026 | 1.648412552 | 0.018828988 |
| 203499_at | EPHA2 | EPH receptor A2 | NM_004431 | 1969 | 1.935443263 | 0.011034108 |
| 208973_at | ERI3 | prion protein interacting protein | BC001072 | 79033 | 1.94811768 | 0.018849687 |
| 227410_at | FAM43A | family with sequence similarity 43, member A | AW264102 | 131583 | 1.675368343 | 0.048775668 |
| 1553749_at | FAM76B | family with sequence similarity 76, member B | NM_144664 | 143684 | -2.503220993 | 0.024396631 |
| 65585_at | FAM86B1 | family with sequence similarity 86, member C /// family with sequence similarity 86, member B1 /// similar to CG7889-PA /// similar to CG7889-PA /// similar to CG7889-PA /// similar to family with sequence similarity 86, member B1 /// family with sequence | AA527515 | 55199 /// 648808 /// 653113 /// 653333 /// 653726 /// 692099 /// 85002 | 1.872093132 | 0.004064845 |
| 200894_s_at | FKBP4 | FK506 binding protein 4, 59kDa | AA894574 | 2288 | 1.62592955 | 0.040060586 |
| 204437_s_at | FOLR1 | folate receptor 1 (adult) | NM_016725 | 2348 | 1.837950526 | 0.000642189 |
| 201564_s_at | FSCN1 | fascin homolog 1, actin-bundling protein (Strongylocentrotus purpuratus) | NM_003088 | 6624 | 1.754277824 | 0.002416102 |
| 205384_at | FXYD1 | FXYD domain containing ion transport regulator 1 (phospholemman) | NM_005031 | 5348 | 1.738280849 | 0.016667979 |
| 202177_at | GAS6 | growth arrest-specific 6 | NM_000820 | 2621 | 1.919568482 | 0.04332728 |
| 201040_at | GNAI2 | guanine nucleotide binding protein (G protein), alpha inhibiting activity polypeptide 2 | NM_002070 | 2771 | 1.678743062 | 0.008163548 |
| 204324_s_at | GOLIM4 | golgi phosphoprotein 4 | NM_014498 | 27333 | -1.738469669 | 0.037763233 |
| 212090_at | GRINA | glutamate receptor, ionotropic, N-methyl D-asparate-associated protein 1 (glutamate binding) | AL571424 | 2907 | 1.698470356 | 0.021572818 |
| 204550_x_at | GSTM1 | glutathione S-transferase M1 | NM_000561 | 2944 | 1.676294195 | 0.00514148 |
| 201673_s_at | GYS1 | glycogen synthase 1 (muscle) | NM_002103 | 2997 | 1.912908853 | 0.040629103 |
| 234665_x_at | HHLA3 | HERV-H LTR-associating 3 | AF126163 | 11147 | 2.138131418 | 0.003019005 |
| 220387_s_at | HHLA3 | HERV-H LTR-associating 3 | NM_007071 | 11147 | 2.526872367 | 0.016671104 |
| 209675_s_at | HNRNPUL1 | heterogeneous nuclear ribonucleoprotein U-like 1 | BC004242 | 11100 | 2.143209086 | 0.002819656 |
| 37512_at | HSD17B6 | hydroxysteroid (17-beta) dehydrogenase 6 | U89281 | 8630 | -1.983734474 | 0.041027683 |
| 211958_at | IGFBP5 | insulin-like growth factor binding protein 5 | R73554 | 3488 | 1.971046934 | 0.020102016 |
| 201234_at | ILK | integrin-linked kinase | NM_004517 | 3611 | 1.803659222 | 0.008733489 |
| 202621_at | IRF3 | interferon regulatory factor 3 | NM_001571 | 3661 | 1.941189301 | 0.007264556 |
| 224749_at | ITFG3 | integrin alpha FG-GAP repeat containing 3 | AI688331 | 83986 | 1.625386799 | 0.009899828 |
| 223727_at | KCNIP2 | Kv channel interacting protein 2 | AL136722 | 30819 | 1.938363353 | 0.015639498 |
| 241790_at | KLHL7 | Kelch-like 7 (Drosophila) | T57946 | 55975 | -1.712257589 | 0.025557452 |
| 212193_s_at | LARP1 | La ribonucleoprotein domain family, member 1 | BE881529 | 23367 | 2.914441269 | 0.011275584 |
| 230057_at | LOC285178 | hypothetical protein LOC285178 | AI609832 | 285178 | -1.79448965 | 0.009032591 |
| 203570_at | LOXL1 | lysyl oxidase-like 1 | NM_005576 | 4016 | 1.712709773 | 0.002949068 |
| 64899_at | LPPR2 | lipid phosphate phosphatase-related protein type 2 | AA209463 | 64748 | 1.712730339 | 0.001364961 |
| 200785_s_at | LRP1 | low density lipoprotein-related protein 1 (alpha-2-macroglobulin receptor) | NM_002332 | 4035 | 1.821901913 | 0.000628218 |
| 242389_at | LUC7L3 | LUC7-like 3 (S. cerevisiae) | BE887449 | 51747 | -1.766946212 | 0.027636779 |
| 223578_x_at | MALAT1 | PRO1073 protein | AF113016 | 29005 | -2.070174099 | 0.016290398 |
| 203151_at | MAP1A | microtubule-associated protein 1A | AW296788 | 4130 | 1.82205765 | 0.020467304 |
| 235740_at | MCTP1 | multiple C2 domains, transmembrane 1 | BG250585 | 79772 | -2.016646752 | 0.02203675 |
| 209035_at | MDK | midkine (neurite growth-promoting factor 2) | M69148 | 4192 | 2.556874576 | 0.003258782 |
| 222175_s_at | MED15 | PC2 (positive cofactor 2, multiprotein complex) glutamine/Q-rich-associated protein | AK000003 | 51586 | 1.661838806 | 0.028897622 |
| 226220_at | METTL9 | DORA reverse strand protein 1 | BE551054 | 51108 | -2.066424177 | 0.005834641 |
| 206500_s_at | MIS18BP1 | chromosome 14 open reading frame 106 | NM_018353 | 55320 | -1.835959055 | 0.037996056 |
| 200948_at | MLF2 | myeloid leukemia factor 2 | NM_005439 | 8079 | 1.786692767 | 0.005172361 |
| 239562_at | MTHFD2L | methylenetetrahydrofolate dehydrogenase (NADP+ dependent) 2-like | AW272411 | 441024 | -1.878005465 | 0.000925685 |
| 238762_at | MTHFD2L | methylenetetrahydrofolate dehydrogenase (NADP+ dependent) 2-like | AA702016 | 441024 | -1.783561652 | 0.035766406 |
| 1553575_at | ND6 | NADH dehydrogenase, subunit 6 (complex I) | NM_173714 | 4541 | -1.828132302 | 0.02510409 |
| 204622_x_at | NR4A2 | nuclear receptor subfamily 4, group A, member 2 | NM_006186 | 4929 | -1.733160051 | 0.025684848 |
| 222027_at | NUCKS1 | Nuclear casein kinase and cyclin-dependent kinase substrate 1 | AW515443 | 64710 | -2.148361314 | 0.033275135 |
| 226643_s_at | NUDCD2 | NudC domain containing 2 | AI291200 | 134492 | -1.629932923 | 0.04726608 |
| 220911_s_at | NYNRIN | KIAA1305 | NM_025081 | 57523 | 2.005812362 | 0.030426962 |
| 215399_s_at | OS9 | amplified in osteosarcoma | AI683900 | 10956 | 1.657747776 | 0.008349281 |
| 212720_at | PAPOLA | poly(A) polymerase alpha | AI670847 | 10914 | -1.736773485 | 0.018010659 |
| 203557_s_at | PCBD1 | pterin-4 alpha-carbinolamine dehydratase/dimerization cofactor of hepatocyte nuclear factor 1 alpha (TCF1) | NM_000281 | 5092 | 2.051556435 | 0.009346372 |
| 215836_s_at | PCDHGA1 /// PCDHGA10 /// PCDHGA11 /// PCDHGA12 /// PCDHGA2 /// PCDHGA3 /// PCDHGA4 /// PCDHGA5 /// PCDHGA6 /// PCDHGA7 /// PCDHGA8 /// PCDHGA9 /// PCDHGB1 /// PCDHGB2 /// PCDHGB3 /// PCDHGB4 /// PCDHGB5 /// PCDHGB6 /// PCDHGB7 /// PCDHGC3 /// PCDHGC4 /// PCDHGC5 | protocadherin gamma subfamily C, 3 /// protocadherin gamma subfamily B, 4 /// protocadherin gamma subfamily A, 8 /// protocadherin gamma subfamily A, 12 /// protocadherin gamma subfamily C, 5 /// protocadherin gamma subfamily C, 4 /// protocadherin gamma | AK026188 | 26025 /// 5098 /// 56097 /// 56098 /// 56099 /// 56100 /// 56101 /// 56102 /// 56103 /// 56104 /// 56105 /// 56106 /// 56107 /// 56108 /// 56109 /// 56110 /// 56111 /// 56112 /// 56113 /// 56114 /// 8641 /// 9708 | 1.636141324 | 0.022396817 |
| 231213_at | PDE1A | phosphodiesterase 1A, calmodulin-dependent | AU146305 | 5136 | -1.939156796 | 0.029747467 |
| 214121_x_at | PDLIM7 | PDZ and LIM domain 7 (enigma) | AA086229 | 9260 | 2.04273451 | 0.026032213 |
| 200634_at | PFN1 | profilin 1 | NM_005022 | 5216 | 2.557433423 | 0.006074672 |
| 1557948_at | PHLDB3 | pleckstrin homology-like domain, family B, member 3 /// similar to pleckstrin homology-like domain, family B, member 1 | BC007947 | 284345 /// 653583 | 1.697291372 | 0.037971955 |
| 212249_at | PIK3R1 | phosphoinositide-3-kinase, regulatory subunit 1 (p85 alpha) | AI934473 | 5295 | -1.661104827 | 0.003244651 |
| 229713_at | PIP4K2A | CDNA FLJ13267 fis, clone OVARC1000964 | AW665227 | 5305 | -1.99396203 | 0.002027329 |
| 202328_s_at | PKD1 | polycystic kidney disease 1 (autosomal dominant) | NM_000296 | 5310 | 1.641310263 | 0.011370564 |
| 201251_at | PKM2 | pyruvate kinase, muscle | NM_002654 | 5315 | 2.702910781 | 0.011006471 |
| 224505_s_at | PLCD4 | phospholipase C, delta 4 /// phospholipase C, delta 4 | BC006355 | 84812 | 2.54172013 | 0.030117569 |
| 201050_at | PLD3 | phospholipase D family, member 3 | NM_012268 | 23646 | 2.212838616 | 0.002954927 |
| 1560556_a_at | PLEKHA8 | Pleckstrin homology domain containing, family A (phosphoinositide binding specific) member 8 | BC016002 | 84725 | -2.546334135 | 0.014052312 |
| 225727_at | PLEKHH1 | pleckstrin homology domain containing, family H (with MyTH4 domain) member 1 | AB033026 | 57475 | 1.672932981 | 0.043386274 |
| 1567213_at | PNN | pinin, desmosome associated protein | U59479 | 5411 | -1.800473288 | 0.026489399 |
| 203718_at | PNPLA6 | neuropathy target esterase | NM_006702 | 10908 | 1.697811719 | 0.03595795 |
| 219152_at | PODXL2 | podocalyxin-like 2 | NM_015720 | 50512 | 1.874346638 | 0.043340504 |
| 227994_x_at | PPDPF | chromosome 20 open reading frame 149 | AA548838 | 79144 | 1.67155227 | 0.000587125 |
| 218010_x_at | PPDPF | chromosome 20 open reading frame 149 | NM_024299 | 79144 | 1.73156395 | 0.001626929 |
| 217841_s_at | PPME1 | protein phosphatase methylesterase 1 | NM_016147 | 51400 | 1.70770339 | 0.000507687 |
| 200695_at | PPP2R1A | protein phosphatase 2 (formerly 2A), regulatory subunit A (PR 65), alpha isoform | NM_014225 | 5518 | 2.195467965 | 0.011259368 |
| 203456_at | PRAF2 | PRA1 domain family, member 2 | NM_007213 | 11230 | 1.641702248 | 0.027572394 |
| 200707_at | PRKCSH | protein kinase C substrate 80K-H | NM_002743 | 5589 | 2.351553103 | 0.00723095 |
| 234340_at | PROCR | Protein C receptor, endothelial (EPCR) | AK021431 | 10544 | 1.790117412 | 0.002114925 |
| 230300_at | PSMA5 | proteasome (prosome, macropain) subunit, alpha type, 5 | N22849 | 5686 | -1.920007574 | 0.039830673 |
| 218045_x_at | PTMS | parathymosin | NM_002824 | 5763 | 2.02254186 | 0.002727157 |
| 221960_s_at | RAB2A | RAB2, member RAS oncogene family | AI189609 | 5862 | -1.737930784 | 0.012656585 |
| 218700_s_at | RAB7L1 | RAB7, member RAS oncogene family-like 1 | BC002585 | 8934 | -1.802473786 | 0.007612254 |
| 221830_at | RAP2A | RAP2A, member of RAS oncogene family | AI302106 | 5911 | -1.716676431 | 0.009938343 |
| 205740_s_at | RBM42 | hypothetical protein MGC10433 | NM_024321 | 79171 | 1.686119644 | 0.010341299 |
| 212398_at | RDX | radixin | AI057093 | 5962 | -1.702052175 | 0.0182368 |
| 243481_at | RHOJ | ras homolog gene family, member J | AA181207 | 57381 | -1.725243168 | 0.045046824 |
| 233819_s_at | RNF160 | zinc finger protein 294 | AK023499 | 26046 | -1.894225724 | 0.030193042 |
| 229420_at | RPL23A | ribosomal protein L23a | AI557425 | 6147 | -1.657685584 | 0.019445593 |
| 225650_at | SAMD1 | sterile alpha motif domain containing 1 | BF207100 | 90378 | 1.801528077 | 0.005192322 |
| 227511_at | SAMD4B | Sterile alpha motif domain containing 4B | BE963280 | 55095 | 1.837432346 | 0.033222508 |
| 205464_at | SCNN1B | sodium channel, nonvoltage-gated 1, beta (Liddle syndrome) | NM_000336 | 6338 | 2.20229874 | 0.008306313 |
| 219194_at | SEMA4G | sema domain, immunoglobulin domain (Ig), transmembrane domain (TM) and short cytoplasmic domain, (semaphorin) 4G | NM_017893 | 57715 | 2.228586587 | 0.000656114 |
| 220454_s_at | SEMA6A | sema domain, transmembrane domain (TM), and cytoplasmic domain, (semaphorin) 6A | NM_020796 | 57556 | 1.929468878 | 0.035285083 |
| 224659_at | SEPN1 | selenoprotein N, 1 | AL020996 | 57190 | 2.110882886 | 0.003868686 |
| 212190_at | SERPINE2 | serpin peptidase inhibitor, clade E (nexin, plasminogen activator inhibitor type 1), member 2 | AL541302 | 5270 | -1.740131717 | 0.023777986 |
| 221269_s_at | SH3BGRL3 | SH3 domain binding glutamic acid-rich protein like 3 /// SH3 domain binding glutamic acid-rich protein like 3 | NM_031286 | 83442 | 1.867830322 | 0.004623025 |
| 238005_s_at | SIN3A | Transcribed locus | AI760013 | 25942 | -1.737796488 | 0.003961397 |
| 209352_s_at | SIN3B | SIN3 homolog B, transcription regulator (yeast) | AB014600 | 23309 | 1.901055264 | 0.015003705 |
| 202856_s_at | SLC16A3 | solute carrier family 16 (monocarboxylic acid transporters), member 3 | NM_004207 | 9123 | 2.332605184 | 0.033610971 |
| 210010_s_at | SLC25A1 | solute carrier family 25 (mitochondrial carrier; citrate transporter), member 1 | U25147 | 6576 | 1.671260955 | 0.027685662 |
| 218494_s_at | SLC2A4RG | SLC2A4 regulator | NM_020062 | 56731 | 1.843884329 | 0.018704302 |
| 1553126_a_at | SLC39A12 | solute carrier family 39 (zinc transporter), member 12 | NM_152725 | 221074 | -3.786214565 | 0.041025529 |
| 203183_s_at | SMARCD1 | SWI/SNF related, matrix associated, actin dependent regulator of chromatin, subfamily d, member 1 | NM_003076 | 6602 | 1.779047574 | 0.008410808 |
| 208611_s_at | SPTAN1 | spectrin, alpha, non-erythrocytic 1 (alpha-fodrin) | U83867 | 6709 | 2.281736324 | 0.024859082 |
| 223635_s_at | SSBP3 | single stranded DNA binding protein 3 | BC003605 | 23648 | 2.05451695 | 0.005830548 |
| 217991_x_at | SSBP3 | single stranded DNA binding protein 3 | NM_018070 | 23648 | 2.207350303 | 0.002432647 |
| 217903_at | STRN4 | striatin, calmodulin binding protein 4 | NM_013403 | 29888 | 1.635835897 | 0.013033921 |
| 210247_at | SYN2 | synapsin II | AW139618 | 6854 | 2.113778108 | 0.042913179 |
| 205691_at | SYNGR3 | synaptogyrin 3 | NM_004209 | 9143 | 1.686632423 | 0.020783006 |
| 201666_at | TIMP1 | TIMP metallopeptidase inhibitor 1 | NM_003254 | 7076 | 1.684355545 | 0.031026677 |
| 219892_at | TM6SF1 | transmembrane 6 superfamily member 1 | NM_023003 | 53346 | -1.642121267 | 0.009853044 |
| 227356_at | TMCC1 | transmembrane and coiled-coil domain family 1 | BG499974 | 23023 | -1.735799377 | 0.009028851 |
| 223523_at | TMEM108 | transmembrane protein 108 | BC000568 | 66000 | -2.007435502 | 0.017298167 |
| 201746_at | TP53 | tumor protein p53 (Li-Fraumeni syndrome) | NM_000546 | 7157 | 1.813848119 | 0.016307669 |
| 1557227_s_at | TPR | translocated promoter region (to activated MET oncogene) | AW235355 | 7175 | -2.036069624 | 0.007111713 |
| 225294_s_at | TRAPPC1 | trafficking protein particle complex 1 | BG340967 | 58485 | 2.227535139 | 0.00188888 |
| 205150_s_at | TRIL | KIAA0644 gene product | AV724192 | 9865 | -2.302370999 | 0.013531512 |
| 205151_s_at | TRIL | KIAA0644 gene product | NM_014817 | 9865 | -2.026099392 | 0.024322587 |
| 1569316_at | TRIM24 | tripartite motif-containing 24 | BC009590 | 8805 | -1.648238858 | 0.048232997 |
| 200990_at | TRIM28 | tripartite motif-containing 28 | NM_005762 | 10155 | 1.794442134 | 0.014391107 |
| 224674_at | TTYH3 | tweety homolog 3 (Drosophila) | AI934753 | 80727 | 1.717184325 | 0.028254748 |
| 212664_at | TUBB4 | tubulin, beta 4 | AL567012 | 10382 | 1.898661069 | 0.010635879 |
| 216609_at | TXN | Thioredoxin | AF065241 | 7295 | -1.72772681 | 0.0026817 |
| 200964_at | UBA1 | ubiquitin-activating enzyme E1 (A1S9T and BN75 temperature sensitivity complementing) | NM_003334 | 7317 | 2.35020743 | 0.013227431 |
| 201378_s_at | UBAP2L | ubiquitin associated protein 2-like | NM_014847 | 9898 | 1.950618734 | 0.008856014 |
| 232219_x_at | USP21 | ubiquitin specific peptidase 21 | AL157417 | 27005 | 1.675856677 | 0.013449823 |
| 218367_x_at | USP21 | ubiquitin specific peptidase 21 | NM_012475 | 27005 | 1.79773688 | 0.011613453 |
| 222941_at | USP46 | hypothetical protein FLJ11850 | AW172493 | 64752 | -1.803832832 | 0.009375433 |
| 201557_at | VAMP2 | vesicle-associated membrane protein 2 (synaptobrevin 2) | NM_014232 | 6844 | 1.934448088 | 0.007923171 |
| 214792_x_at | VAMP2 | Vesicle-associated membrane protein 2 (synaptobrevin 2) | AI955119 | 6844 | 1.966484059 | 0.024038355 |
| 226029_at | VANGL2 | vang-like 2 (van gogh, Drosophila) | AB033041 | 57216 | 1.63140205 | 0.013890193 |
| 209117_at | WBP2 | WW domain binding protein 2 | U79458 | 23558 | 1.733232283 | 0.022489705 |
| 208033_s_at | ZFHX3 | AT-binding transcription factor 1 | NM_006885 | 463 | 1.968828811 | 0.00365423 |
| 60794_f_at | ZNF814 | similar to Zinc finger protein 418 | AI400621 | 400721 | -1.652250964 | 0.042913309 |
| 200808_s_at | ZYX | zyxin | NM_003461 | 7791 | 2.124900375 | 0.022475651 |

HD-CAV-2 2h

| **Probe Set ID** | **Gene Symbol** | **Gene Title** | **Accession number** | **EntrezGene** | **Fold Change** | **PValue** |
| --- | --- | --- | --- | --- | --- | --- |
| 215555_at | C1orf63 | Chromosome 1 open reading frame 63 | AU158442 | 57035 | -1.635656459 | 0.048780488 |
| 227174_at | WDR72 | WD repeat domain 72 | Z98443 | 256764 | -1.717974796 | 0.040419028 |
| 243827_at | --- | Transcribed locus | AL038125 |  | -1.813411085 | 0.041082915 |

HD-CAV-2 5d

| **Probe Set ID** | **Gene Symbol** | **Gene Title** | **Accession number** | **EntrezGene** | **Fold Change** | **PValue** |
| --- | --- | --- | --- | --- | --- | --- |
| 235227_at | --- | CDNA clone IMAGE:5287121 | AI025829 |  | -1.792220246 | 0.048530864 |
| 239049_at | --- | CDNA FLJ13202 fis, clone NT2RP3004503 | BF514509 |  | -2.054845097 | 0.036715088 |
| 239050_s_at | --- | CDNA FLJ13202 fis, clone NT2RP3004503 | BF514509 |  | -1.683535564 | 0.049411963 |
| 233068_at | --- | CDNA FLJ13202 fis, clone NT2RP3004503 | AK023264 |  | -1.681881178 | 0.035931913 |
| 229351_at | --- | CDNA FLJ13620 fis, clone PLACE1010947 | AA634138 |  | -1.70940411 | 0.006879601 |
| 238492_at | --- | CDNA FLJ33148 fis, clone UTERU2000238 | BF941414 |  | -1.636060336 | 0.04344223 |
| 241721_at | --- | CDNA FLJ37844 fis, clone BRSSN2012622 | AW515022 |  | -1.942410091 | 0.018935021 |
| 230596_at | --- | CDNA FLJ39261 fis, clone OCBBF2009391 | AI692499 |  | -3.260087939 | 0.024764929 |
| 227508_at | --- | CDNA FLJ40982 fis, clone UTERU2014601 | AI302271 |  | -1.640086356 | 0.009974566 |
| 235028_at | --- | CDNA FLJ42313 fis, clone TRACH2019425 | BG288330 |  | -2.339473968 | 0.002314874 |
| 214376_at | --- | Clone 24626 mRNA sequence | AI263044 |  | -1.925779199 | 0.021116596 |
| 233506_at | --- | Full length insert cDNA clone ZB81B12 | N95440 |  | -1.988951396 | 0.004590057 |
| 228914_at | --- | gb:AI769673 /DB_XREF=gi:5236182 /DB_XREF=wj25e06.x1 /CLONE=IMAGE:2403874 /FEA=EST /CNT=16 /TID=Hs.6694.0 /TIER=Stack /STK=11 /UG=Hs.6694 /UG_TITLE=ESTs | AI769673 |  | -1.62530075 | 0.010272537 |
| 238842_at | --- | gb:BE000242 /DB_XREF=gi:8260475 /DB_XREF=MR0-BN0070-260400-017-b09 /FEA=EST /CNT=8 /TID=Hs.192068.0 /TIER=ConsEnd /STK=0 /UG=Hs.192068 /UG_TITLE=ESTs | BE000242 |  | -1.688059799 | 0.040034588 |
| 242346_x_at | --- | gb:BF222929 /DB_XREF=gi:11130106 /DB_XREF=7q25b10.x1 /CLONE=IMAGE:3699402 /FEA=EST /CNT=3 /TID=Hs.290585.0 /TIER=ConsEnd /STK=3 /UG=Hs.290585 /UG_TITLE=ESTs | BF222929 |  | 2.736395059 | 0.000940131 |
| 217166_at | --- | gb:J04798 /DB_XREF=gi:190368 /FEA=DNA /CNT=1 /TID=Hs.247931.0 /TIER=ConsEnd /STK=0 /UG=Hs.247931 /UG_TITLE=Human prothymosin-alpha pseudogene, complete sequence /DEF=Human prothymosin-alpha pseudogene, complete sequence | J04798 |  | -1.646967438 | 0.007192646 |
| 221043_at | --- | gb:NM_013395.1 /DB_XREF=gi:7019320 /GEN=AD013 /FEA=FLmRNA /CNT=2 /TID=Hs.125294.0 /TIER=FL /STK=0 /UG=Hs.125294 /LL=29962 /DEF=Homo sapiens proteinx0008 (AD013), mRNA. /PROD=proteinx0008 /FL=gb:NM_013395.1 gb:AF150735.1 | NM_013395 |  | -2.179903901 | 0.032396962 |
| 220711_at | --- | gb:NM_024978.1 /DB_XREF=gi:13376479 /GEN=FLJ12121 /FEA=FLmRNA /CNT=5 /TID=Hs.287487.0 /TIER=FL /STK=0 /UG=Hs.287487 /LL=80043 /DEF=Homo sapiens hypothetical protein FLJ12121 (FLJ12121), mRNA. /PROD=hypothetical protein FLJ12121 /FL=gb:NM_024978.1 | NM_024978 |  | -1.825777156 | 0.047514331 |
| 233142_at | --- | Homo sapiens, clone IMAGE:4401608, mRNA | AK022793 |  | 2.496913945 | 1.23547E-05 |
| 235534_at | --- | Homo sapiens, clone IMAGE:5723825, mRNA | AI624156 |  | 1.647677611 | 0.029852359 |
| 239735_at | --- | MRNA (fetal brain cDNA g6_1g) | N67106 |  | -1.951419473 | 0.04507407 |
| 233481_at | --- | MRNA; cDNA DKFZp566O1624 (from clone DKFZp566O1624) | AL117559 |  | -2.450024071 | 0.042067498 |
| 237299_at | --- | Transcribed locus | T71642 |  | -1.744288487 | 0.022883096 |
| 240000_at | --- | Transcribed locus | AA621615 |  | 1.792484082 | 0.016767881 |
| 235363_at | --- | Transcribed locus | BF108778 |  | 2.353684756 | 0.001365856 |
| 239587_at | --- | Transcribed locus | AI686890 |  | 2.745412667 | 0.001016514 |
| 229490_s_at | --- | Transcribed locus, strongly similar to NP_839943.2 IQ motif containing GTPase activating protein 3 [Homo sapiens] | AW271106 |  | 1.896186415 | 0.039289334 |
| 236312_at | --- | Transcribed locus, weakly similar to NP_071385.1 hypothetical protein FLJ20958 [Homo sapiens] | AA938184 |  | 2.542726254 | 0.009872438 |
| 228955_at | --- | Transcribed locus, weakly similar to NP_990560.1 very low density lipoprotein (VLDL)/vitellogenin receptor [Gallus gallus] | AL041761 |  | -2.097581599 | 0.01966901 |
| 209994_s_at | ABCB1 /// ABCB4 | ATP-binding cassette, sub-family B (MDR/TAP), member 1 /// ATP-binding cassette, sub-family B (MDR/TAP), member 4 | AF016535 | 5243 /// 5244 | 2.022196137 | 0.007162413 |
| 206068_s_at | ACADL | acyl-CoA dehydrogenase, long chain | AI367275 | 33 | 1.7870551 | 0.0395662 |
| 206069_s_at | ACADL | acyl-CoA dehydrogenase, long chain | NM_001608 | 33 | 1.826047059 | 0.004614495 |
| 200974_at | ACTA2 | actin, alpha 2, smooth muscle, aorta | NM_001613 | 59 | 3.855647611 | 0.014457716 |
| 208636_at | ACTN1 | actinin, alpha 1 | AI082078 | 87 | 1.645348619 | 0.027483648 |
| 208637_x_at | ACTN1 | actinin, alpha 1 | BC003576 | 87 | 1.978911877 | 0.004559153 |
| 236817_at | ADAT2 | adenosine deaminase, tRNA-specific 2, TAD2 homolog (S. cerevisiae) | AI336346 | 134637 | 1.976468601 | 0.010716707 |
| 235441_at | ADCY3 | Adenylate cyclase 3 | BF217471 | 109 | 1.713930775 | 0.000655968 |
| 228771_at | ADRBK2 | adrenergic, beta, receptor kinase 2 | AI651212 | 157 | -2.221294363 | 0.042009414 |
| 222126_at | AGFG2 | ArfGAP with FG repeats 2 | AI247494 | 3268 | 1.74053761 | 0.022524325 |
| 1555736_a_at | AGTRAP | angiotensin II receptor-associated protein | AF165187 | 57085 | 1.642523159 | 0.042568079 |
| 204151_x_at | AKR1C1 | aldo-keto reductase family 1, member C1 (dihydrodiol dehydrogenase 1; 20-alpha (3-alpha)-hydroxysteroid dehydrogenase) | NM_001353 | 1645 | -2.690748559 | 0.04556514 |
| 212607_at | AKT3 | v-akt murine thymoma viral oncogene homolog 3 (protein kinase B, gamma) | N32526 | 10000 | -1.767900601 | 0.003403134 |
| 212609_s_at | AKT3 | V-akt murine thymoma viral oncogene homolog 3 (protein kinase B, gamma) | U79271 | 10000 | -1.712658003 | 0.009467066 |
| 209646_x_at | ALDH1B1 | aldehyde dehydrogenase 1 family, member B1 | BC001619 | 219 | 1.680167112 | 0.006819174 |
| 203545_at | ALG8 | asparagine-linked glycosylation 8, alpha-1,3-glucosyltransferase homolog (S. cerevisiae) | NM_024079 | 79053 | 2.044144114 | 0.024708938 |
| 219082_at | AMDHD2 | amidohydrolase domain containing 2 | NM_015944 | 51005 | 1.736178958 | 0.049003162 |
| 223092_at | ANKH | ankylosis, progressive homolog (mouse) | AA854943 | 56172 | -1.713927529 | 0.004669028 |
| 226663_at | ANKRD10 | ankyrin repeat domain 10 | BE670056 | 55608 | -2.160509749 | 0.000324863 |
| 218093_s_at | ANKRD10 | ankyrin repeat domain 10 | NM_017664 | 55608 | -1.712640148 | 0.017907093 |
| 216563_at | ANKRD12 | Ankyrin repeat domain 12 | X80821 | 23253 | -1.869921957 | 0.021950764 |
| 201302_at | ANXA4 | annexin A4 | NM_001153 | 307 | 2.059267557 | 0.000899816 |
| 201301_s_at | ANXA4 | annexin A4 | BC000182 | 307 | 2.235571436 | 0.008365566 |
| 206632_s_at | APOBEC3B | apolipoprotein B mRNA editing enzyme, catalytic polypeptide-like 3B | NM_004900 | 9582 | 2.654988167 | 0.004159314 |
| 204492_at | ARHGAP11A | Rho GTPase activating protein 11A | NM_014783 | 9824 | 2.428780993 | 0.019574519 |
| 227911_at | ARHGAP28 | Rho GTPase activating protein 28 | AI935647 | 79822 | -1.686678747 | 0.021707396 |
| 230047_at | ARHGAP42 | Rho GTPase activating protein 42 | BF439533 | 143872 | 1.761348084 | 0.011167537 |
| 240008_at | ARID1B | AT rich interactive domain 1B (SWI1-like) | AI955765 | 57492 | -1.732703011 | 0.046416064 |
| 238043_at | ARID1B | AT rich interactive domain 1B (SWI1-like) | AI913123 | 57492 | -2.387296507 | 0.047650783 |
| 231269_at | ASCC3 | activating signal cointegrator 1 complex subunit 3 | AU153330 | 10973 | 1.634607512 | 0.004704322 |
| 212815_at | ASCC3 | activating signal cointegrator 1 complex subunit 3 | AA156961 | 10973 | 1.954490427 | 7.04436E-05 |
| 202024_at | ASNA1 | arsA arsenite transporter, ATP-binding, homolog 1 (bacterial) | NM_004317 | 439 | 1.643794759 | 0.044925537 |
| 220223_at | ATAD5 | ATPase family, AAA domain containing 5 | NM_024857 | 79915 | 1.626585177 | 0.000320196 |
| 239825_at | ATF6 | Activating transcription factor 6 | R17746 | 22926 | 1.915251082 | 0.011661339 |
| 213106_at | ATP8A1 | ATPase, aminophospholipid transporter (APLT), class I, type 8A, member 1 | AI769688 | 10396 | -2.569218401 | 0.023113422 |
| 235240_at | ATXN3 | ataxin 3 | N51479 | 4287 | -1.656159698 | 0.001935087 |
| 212599_at | AUTS2 | autism susceptibility candidate 2 | AK025298 | 26053 | -1.777721967 | 0.007762247 |
| 229715_at | B7H6 | CDNA FLJ41663 fis, clone FEBRA2027297 | AW006182 | 374383 | 2.679387212 | 0.002435877 |
| 243302_at | BCKDHB | Branched chain keto acid dehydrogenase E1, beta polypeptide (maple syrup urine disease) | AI452738 | 594 | 1.650068544 | 0.0072002 |
| 222895_s_at | BCL11B | B-cell CLL/lymphoma 11B (zinc finger protein) | AA918317 | 64919 | -4.729683341 | 0.031064977 |
| 219528_s_at | BCL11B | B-cell CLL/lymphoma 11B (zinc finger protein) | NM_022898 | 64919 | -4.221860158 | 0.036288166 |
| 233110_s_at | BCL2L12 | BCL2-like 12 (proline rich) | AF289220 | 83596 | 1.700195841 | 0.027733257 |
| 223915_at | BCOR | BCL6 co-repressor | AF317392 | 54880 | -1.939518702 | 0.022086119 |
| 206956_at | BGLAP | bone gamma-carboxyglutamate (gla) protein | NM_000711 | 632 | 1.662226156 | 0.028635685 |
| 210334_x_at | BIRC5 | baculoviral IAP repeat-containing 5 | AB028869 | 332 | 2.134879609 | 0.01602456 |
| 202094_at | BIRC5 | baculoviral IAP repeat-containing 5 | AA648913 | 332 | 2.664015677 | 0.035050334 |
| 205733_at | BLM | Bloom syndrome, RecQ helicase-like | NM_000057 | 641 | 1.972363292 | 0.002994933 |
| 208368_s_at | BRCA2 | breast cancer 2, early onset | NM_000059 | 675 | 3.247695462 | 0.001005784 |
| 214727_at | BRCA2 | breast cancer 2, early onset | X95152 | 675 | 3.715671638 | 0.001096005 |
| 243509_at | BTG1 | B-cell translocation gene 1, anti-proliferative | AI475680 | 694 | -2.604921702 | 0.049408977 |
| 226892_at | C10orf12 | chromosome 10 open reading frame 12 | AK025166 | 26148 | -1.687213485 | 0.01401988 |
| 241198_s_at | C11orf70 | chromosome 11 open reading frame 70 | BE645435 | 85016 | 1.687654248 | 0.001350113 |
| 228281_at | C11orf82 | chromosome 11 open reading frame 82 | BF343258 | 220042 | 4.880602529 | 0.000375808 |
| 204521_at | C12orf24 | chromosome 12 open reading frame 24 | NM_013300 | 29902 | 1.704034491 | 0.018397384 |
| 227152_at | C12orf35 | chromosome 12 open reading frame 35 | AI979334 | 55196 | -2.107190165 | 0.022296951 |
| 220060_s_at | C12orf48 | chromosome 12 open reading frame 48 | NM_017915 | 55010 | 2.644789744 | 0.003076182 |
| 227928_at | C12orf48 | chromosome 12 open reading frame 48 | AI224977 | 55010 | 4.084982197 | 0.001477946 |
| 219099_at | C12orf5 | chromosome 12 open reading frame 5 | NM_020375 | 57103 | 1.847191817 | 0.008814462 |
| 235026_at | C12orf66 | chromosome 12 open reading frame 66 | AI885871 | 144577 | 1.757024496 | 0.036144101 |
| 225105_at | C12orf75 | chromosome 12 open reading frame 75 | BF969397 | 387882 | 2.760176838 | 7.24632E-05 |
| 206500_s_at | C14orf106 | chromosome 14 open reading frame 106 | NM_018353 | 55320 | 1.793170603 | 0.045363311 |
| 214264_s_at | C14orf143 | chromosome 14 open reading frame 143 | AI656610 | 90141 | 2.130141443 | 0.010702726 |
| 1557755_at | C14orf145 | chromosome 14 open reading frame 145 | AW028337 | 145508 | 2.041150676 | 7.02961E-05 |
| 232635_at | C14orf145 | chromosome 14 open reading frame 145 | AV703868 | 145508 | 2.045002912 | 0.004469976 |
| 1557756_a_at | C14orf145 | chromosome 14 open reading frame 145 | AW028337 | 145508 | 2.472493056 | 0.000408941 |
| 244033_at | C14orf145 | chromosome 14 open reading frame 145 | AI937080 | 145508 | 3.379051444 | 0.000152175 |
| 227699_at | C14orf149 | chromosome 14 open reading frame 149 | BF511003 | 112849 | 1.93781429 | 0.021361234 |
| 232094_at | C15orf29 | chromosome 15 open reading frame 29 | AU144048 | 79768 | -1.67674071 | 0.041930164 |
| 1553440_at | C18orf16 | chromosome 18 open reading frame 16 | NM_153010 | 147429 | 2.480748552 | 0.024626847 |
| 229442_at | C18orf54 | chromosome 18 open reading frame 54 | BF059556 | 162681 | 1.755698274 | 0.023874425 |
| 244324_at | C18orf54 | chromosome 18 open reading frame 54 | BG283921 | 162681 | 1.861578654 | 0.016188026 |
| 228989_at | C18orf56 | chromosome 18 open reading frame 56 | AW291159 | 494514 | 2.882675753 | 2.19526E-05 |
| 214816_x_at | C19orf40 | chromosome 19 open reading frame 40 | BC003535 | 91442 | 1.762079808 | 0.002782196 |
| 224468_s_at | C19orf48 | chromosome 19 open reading frame 48 | BC006151 | 84798 | 1.693590908 | 0.004687792 |
| 220840_s_at | C1orf112 | chromosome 1 open reading frame 112 | NM_018186 | 55732 | 2.472851494 | 0.010746255 |
| 222946_s_at | C1orf135 | chromosome 1 open reading frame 135 | BC000209 | 79000 | 2.537794843 | 0.001056942 |
| 220011_at | C1orf135 | chromosome 1 open reading frame 135 | NM_024037 | 79000 | 3.363230735 | 0.000622009 |
| 223459_s_at | C1orf56 | chromosome 1 open reading frame 56 | BE222214 | 54964 | 1.696039088 | 0.037052918 |
| 215555_at | C1orf63 | Chromosome 1 open reading frame 63 | AU158442 | 57035 | -2.37470657 | 0.001321008 |
| 1558693_s_at | C1orf85 | chromosome 1 open reading frame 85 | AW090182 | 112770 | 1.685727213 | 0.009568461 |
| 225401_at | C1orf85 | chromosome 1 open reading frame 85 | BF977145 | 112770 | 1.740722715 | 0.003582679 |
| 225890_at | C20orf72 | chromosome 20 open reading frame 72 | AI678096 | 92667 | 1.722863644 | 0.026978701 |
| 228597_at | C21orf45 | chromosome 21 open reading frame 45 | AW151538 | 54069 | 2.247234468 | 0.038749092 |
| 219004_s_at | C21orf45 | chromosome 21 open reading frame 45 | NM_018944 | 54069 | 2.539413721 | 5.16574E-05 |
| 224523_s_at | C3orf26 | chromosome 3 open reading frame 26 | BC006475 | 84319 | 2.013764777 | 0.011161601 |
| 1554176_a_at | C3orf33 | chromosome 3 open reading frame 33 | AF115515 | 285315 | 1.641085637 | 0.005956139 |
| 227599_at | C3orf59 | chromosome 3 open reading frame 59 | AU157304 | 151963 | -1.678415986 | 0.049046172 |
| 221935_s_at | C3orf64 | chromosome 3 open reading frame 64 | AK023140 | 285203 | 1.648727276 | 0.016653418 |
| 228859_at | C4orf21 | chromosome 4 open reading frame 21 | BF056790 | 91431 | 2.269104422 | 0.009112051 |
| 230424_at | C5orf13 | chromosome 5 open reading frame 13 | AU144860 | 9315 | -2.031246514 | 0.047491144 |
| 229886_at | C5orf34 | chromosome 5 open reading frame 34 | AA502768 | 375444 | 4.710479065 | 2.86977E-06 |
| 204238_s_at | C6orf108 | chromosome 6 open reading frame 108 | NM_006443 | 10591 | 1.650970148 | 0.046017878 |
| 204215_at | C7orf23 | chromosome 7 open reading frame 23 | NM_024315 | 79161 | 1.729265121 | 0.01124786 |
| 225915_at | CAB39L | calcium binding protein 39-like | AL138875 | 81617 | -1.761480541 | 0.039260536 |
| 219928_s_at | CABYR | calcium binding tyrosine-(Y)-phosphorylation regulated | NM_012189 | 26256 | 2.086525494 | 0.005430659 |
| 34726_at | CACNB3 | calcium channel, voltage-dependent, beta 3 subunit | U07139 | 784 | -1.782287036 | 0.031432151 |
| 204392_at | CAMK1 | calcium/calmodulin-dependent protein kinase I | NM_003656 | 8536 | 1.658409892 | 0.026721488 |
| 212710_at | CAMSAP1 | calmodulin regulated spectrin-associated protein 1 | AL043774 | 157922 | -1.628859549 | 0.038843231 |
| 1552703_s_at | CARD16 /// CASP1 | caspase recruitment domain family, member 16 /// caspase 1, apoptosis-related cysteine peptidase (interleukin 1, beta, convertase) | NM_052889 | 114769 /// 834 | 1.813840944 | 0.031989506 |
| 226085_at | CBX5 | chromobox homolog 5 (HP1 alpha homolog, Drosophila) | AA181060 | 23468 | -1.943316664 | 0.027121073 |
| 235644_at | CCDC138 | coiled-coil domain containing 138 | BF213953 | 165055 | 1.831996548 | 0.009037696 |
| 220466_at | CCDC15 | coiled-coil domain containing 15 | NM_025004 | 80071 | 1.781299474 | 0.002267353 |
| 241360_at | CCDC15 | coiled-coil domain containing 15 | BG413368 | 80071 | 1.968705843 | 0.001144642 |
| 232398_at | CCDC150 | coiled-coil domain containing 150 | AK001064 | 284992 /// 642077 | 4.411882032 | 0.002786023 |
| 226287_at | CCDC34 | coiled-coil domain containing 34 | AI458313 | 91057 | 2.461202676 | 0.005491801 |
| 213644_at | CCDC46 | coiled-coil domain containing 46 | AI979276 | 201134 | 1.766288324 | 0.029427749 |
| 234995_at | CCDC52 | coiled-coil domain containing 52 | AA668779 | 152185 | 1.705961289 | 0.001288076 |
| 1559222_at | CCDC99 | Coiled-coil domain containing 99 | AA781731 | 54908 | 1.738989104 | 0.006669353 |
| 221685_s_at | CCDC99 | coiled-coil domain containing 99 | AF269167 | 54908 | 1.624525853 | 0.032166025 |
| 213523_at | CCNE1 | cyclin E1 | AI671049 | 898 | 1.883768722 | 0.012771309 |
| 209835_x_at | CD44 | CD44 molecule (Indian blood group) | BC004372 | 960 | 1.845638658 | 0.008855587 |
| 204490_s_at | CD44 | CD44 molecule (Indian blood group) | M24915 | 960 | 1.906140551 | 0.027670991 |
| 212014_x_at | CD44 | CD44 molecule (Indian blood group) | AI493245 | 960 | 1.941587619 | 0.010191555 |
| 204489_s_at | CD44 | CD44 molecule (Indian blood group) | NM_000610 | 960 | 2.092663787 | 0.012673081 |
| 208783_s_at | CD46 | CD46 molecule, complement regulatory protein | AL570661 | 4179 | -1.701052667 | 0.021836933 |
| 243931_at | CD58 | CD58 molecule | R64696 | 965 | 1.626236825 | 0.026853615 |
| 211744_s_at | CD58 | CD58 molecule | BC005930 | 965 | 1.697520498 | 0.005167216 |
| 216942_s_at | CD58 | CD58 molecule | D28586 | 965 | 1.775771157 | 0.002366352 |
| 204695_at | CDC25A | cell division cycle 25 homolog A (S. pombe) | AI343459 | 993 | 1.688475226 | 0.020518442 |
| 201853_s_at | CDC25B | cell division cycle 25 homolog B (S. pombe) | NM_021873 | 994 | 2.316088119 | 0.000767347 |
| 205167_s_at | CDC25C | cell division cycle 25 homolog C (S. pombe) | NM_001790 | 995 | 2.617989316 | 0.008702734 |
| 224753_at | CDCA5 | cell division cycle associated 5 | BE614410 | 113130 | 2.422935543 | 0.005833901 |
| 203252_at | CDK2AP2 | cyclin-dependent kinase 2 associated protein 2 | NM_005851 | 10263 | 1.935665705 | 0.012561728 |
| 202284_s_at | CDKN1A | cyclin-dependent kinase inhibitor 1A (p21, Cip1) | NM_000389 | 1026 | 2.448700567 | 0.000762859 |
| 213348_at | CDKN1C | cyclin-dependent kinase inhibitor 1C (p57, Kip2) | N33167 | 1028 | -2.312525775 | 0.049445167 |
| 209832_s_at | CDT1 | chromatin licensing and DNA replication factor 1 | AF321125 | 81620 | 1.71483921 | 0.026881907 |
| 205165_at | CELSR3 | cadherin, EGF LAG seven-pass G-type receptor 3 (flamingo homolog, Drosophila) | NM_001407 | 1951 | -1.844400223 | 0.023395985 |
| 40020_at | CELSR3 | cadherin, EGF LAG seven-pass G-type receptor 3 (flamingo homolog, Drosophila) | AB011536 | 1951 | -1.731062818 | 0.045468999 |
| 207828_s_at | CENPF | centromere protein F, 350/400ka (mitosin) | NM_005196 | 1063 | 2.208202311 | 0.031103981 |
| 231772_x_at | CENPH | centromere protein H | AL572471 | 64946 | 2.397199733 | 0.008930197 |
| 214804_at | CENPI | centromere protein I | BF793446 | 2491 | 2.041419503 | 0.017810609 |
| 232065_x_at | CENPL | centromere protein L | N29457 | 91687 | 1.866744151 | 0.038589096 |
| 1554271_a_at | CENPL | centromere protein L | BC019022 | 91687 | 2.414343117 | 0.02067776 |
| 218741_at | CENPM | centromere protein M | NM_024053 | 79019 | 2.627907334 | 0.00922267 |
| 228559_at | CENPN | centromere protein N | BF111626 | 55839 | 2.460137741 | 0.038976827 |
| 222118_at | CENPN | centromere protein N | AK023669 | 55839 | 2.837366709 | 0.006619985 |
| 226118_at | CENPO | centromere protein O | BE326728 | 79172 | 1.885885742 | 0.005649854 |
| 242207_at | CENPP /// LOC100128361 | centromere protein P /// similar to hCG1985411 | AW292595 | 64768 | 2.015973498 | 0.000234177 |
| 219294_at | CENPQ | centromere protein Q | NM_018132 | 55166 | 2.194179881 | 0.012642855 |
| 226936_at | CENPW | centromere protein W | BG492359 | 387103 | 2.877342204 | 0.000443036 |
| 206003_at | CEP135 | centrosomal protein 135kDa | NM_014645 | 9662 | 2.077710856 | 0.007528457 |
| 219531_at | CEP72 | centrosomal protein 72kDa | NM_018140 | 55722 | 2.048592562 | 0.000999488 |
| 218421_at | CERK | ceramide kinase | NM_022766 | 64781 | -1.700356286 | 0.035044139 |
| 238299_at | CERS5 | LAG1 longevity assurance homolog 5 (S. cerevisiae) | AW005866 | 91012 | -2.108022038 | 0.043701906 |
| 1555564_a_at | CFI | complement factor I | BC020718 | 3426 | 2.217154717 | 0.016292671 |
| 203854_at | CFI | complement factor I | NM_000204 | 3426 | 4.391778727 | 0.016335001 |
| 214426_x_at | CHAF1A | chromatin assembly factor 1, subunit A (p150) | BF062223 | 10036 | 1.658123749 | 0.040095235 |
| 203976_s_at | CHAF1A | chromatin assembly factor 1, subunit A (p150) | NM_005483 | 10036 | 1.80197514 | 0.01964468 |
| 224932_at | CHCHD10 | coiled-coil-helix-coiled-coil-helix domain containing 10 | AI814909 | 400916 | 1.890585012 | 0.029926006 |
| 205394_at | CHEK1 | CHK1 checkpoint homolog (S. pombe) | NM_001274 | 1111 | 2.219139372 | 0.030309576 |
| 205393_s_at | CHEK1 | CHK1 checkpoint homolog (S. pombe) | NM_001274 | 1111 | 2.261648751 | 0.012451168 |
| 210416_s_at | CHEK2 | CHK2 checkpoint homolog (S. pombe) | BC004207 | 11200 | 3.615618257 | 0.000520392 |
| 32094_at | CHST3 | carbohydrate (chondroitin 6) sulfotransferase 3 | AB017915 | 9469 | 1.74942674 | 0.006600711 |
| 209834_at | CHST3 | carbohydrate (chondroitin 6) sulfotransferase 3 | AB017915 | 9469 | 1.802067745 | 0.019977319 |
| 226569_s_at | CHTF18 | CTF18, chromosome transmission fidelity factor 18 homolog (S. cerevisiae) | AK024476 | 63922 | 1.969745216 | 0.002364473 |
| 201897_s_at | CKS1B | CDC28 protein kinase regulatory subunit 1B | NM_001826 | 1163 | 2.744873661 | 0.001182461 |
| 204170_s_at | CKS2 | CDC28 protein kinase regulatory subunit 2 | NM_001827 | 1164 | 2.208780333 | 0.0213342 |
| 1558924_s_at | CLIP1 | CAP-GLY domain containing linker protein 1 | BF673049 | 6249 | -1.83203205 | 0.037558325 |
| 241403_at | CLK4 | CDC-like kinase 4 | AA468591 | 57396 | -1.84812519 | 0.023350886 |
| 227522_at | CMBL | carboxymethylenebutenolidase homolog (Pseudomonas) | AA209487 | 134147 | 2.063362359 | 0.000101559 |
| 1552977_a_at | CNPY3 | canopy 3 homolog (zebrafish) | NM_006586 | 10695 | 2.00670935 | 0.024630968 |
| 230045_at | CNTN2 | contactin 2 (axonal) | BF740264 | 6900 | -1.828694181 | 0.02123793 |
| 223796_at | CNTNAP3 | contactin associated protein-like 3 | AF333769 | 389734 /// 642342 /// 642373 /// 643792 /// 79937 | 2.053069796 | 0.00325179 |
| 244065_at | CNTNAP3B | contactin associated protein-like 3B | AW016751 | 389734 /// 642373 /// 643792 | 1.987529766 | 0.004335469 |
| 221730_at | COL5A2 | collagen, type V, alpha 2 | NM_000393 | 1290 | 1.979726877 | 0.002489248 |
| 221729_at | COL5A2 | collagen, type V, alpha 2 | AL575735 | 1290 | 2.163344426 | 0.000539028 |
| 213379_at | COQ2 | coenzyme Q2 homolog, prenyltransferase (yeast) | AF091086 | 27235 | 1.698025678 | 0.023351932 |
| 214277_at | COX11 | COX11 cytochrome c oxidase assembly homolog (yeast) | AI376724 | 1353 /// 140468 | 2.037678008 | 0.011643572 |
| 238733_at | CPM | Carboxypeptidase M | AI422414 | 1368 | 2.813034679 | 0.001305048 |
| 204920_at | CPS1 | carbamoyl-phosphate synthase 1, mitochondrial | AF154830 | 1373 | 2.093287663 | 0.000582178 |
| 208146_s_at | CPVL | carboxypeptidase, vitellogenic-like | NM_031311 | 54504 | 1.72397817 | 0.032571905 |
| 201380_at | CRTAP | cartilage associated protein | NM_006371 | 10491 | 1.909388193 | 0.007419415 |
| 1554464_a_at | CRTAP | cartilage associated protein | BC008745 | 10491 | 2.191226221 | 0.012777878 |
| 209283_at | CRYAB | crystallin, alpha B | AF007162 | 1410 | 1.995763143 | 0.029537265 |
| 201161_s_at | CSDA | cold shock domain protein A | NM_003651 | 8531 | 2.023714377 | 0.000664183 |
| 201160_s_at | CSDA | cold shock domain protein A | AL556190 | 8531 | 2.176182299 | 0.000667094 |
| 220462_at | CSRNP3 | cysteine-serine-rich nuclear protein 3 | NM_024969 | 80034 | -1.787737159 | 0.041883686 |
| 235017_s_at | CSRNP3 | cysteine-serine-rich nuclear protein 3 | BF697865 | 80034 | -1.701267426 | 0.046730455 |
| 225647_s_at | CTSC | cathepsin C | AI246687 | 1075 | 2.987531965 | 0.015221676 |
| 210074_at | CTSL2 | cathepsin L2 | AF070448 | 1515 | 2.872159857 | 0.002040265 |
| 823_at | CX3CL1 | chemokine (C-X3-C motif) ligand 1 | U84487 | 6376 | 1.772569558 | 0.014917142 |
| 244803_at | DAP3 | Death associated protein 3 | AI335191 | 7818 | -2.309082404 | 0.019136802 |
| 218365_s_at | DARS2 | aspartyl-tRNA synthetase 2, mitochondrial | AI765051 | 55157 | 1.794931915 | 0.026568487 |
| 206588_at | DAZL | deleted in azoospermia-like | NM_001351 | 1618 | 2.015795418 | 0.01091071 |
| 205818_at | DBC1 | deleted in bladder cancer 1 | NM_014618 | 1620 | -2.590874756 | 0.038126074 |
| 238508_at | DBF4B | DBF4 homolog B (S. cerevisiae) | BG026951 | 80174 | 2.165284872 | 0.003189551 |
| 218774_at | DCPS | decapping enzyme, scavenger | NM_014026 | 28960 | 1.644619269 | 0.042039453 |
| 217973_at | DCXR | dicarbonyl/L-xylulose reductase | NM_016286 | 51181 | 1.770337495 | 0.042070957 |
| 230180_at | DDX17 | DEAD (Asp-Glu-Ala-Asp) box polypeptide 17 | AA521056 | 10521 | -2.413451139 | 0.0114719 |
| 235545_at | DEPDC1 | DEP domain containing 1 | AI810054 | 55635 | 2.90124036 | 0.038177448 |
| 222958_s_at | DEPDC1 | DEP domain containing 1 | AK000490 | 55635 | 2.933901406 | 0.015489927 |
| 232278_s_at | DEPDC1 | DEP domain containing 1 | AJ278112 | 55635 | 2.948921152 | 0.010497208 |
| 226980_at | DEPDC1B | DEP domain containing 1B | AK001166 | 55789 | 2.384218725 | 0.046265552 |
| 1561114_a_at | DEPDC4 | DEP domain containing 4 | BC039480 | 120863 | 1.85938987 | 0.00244851 |
| 218756_s_at | DHRS11 | dehydrogenase/reductase (SDR family) member 11 | NM_024308 | 79154 | 1.653516471 | 0.041427939 |
| 213229_at | DICER1 | dicer 1, ribonuclease type III | BF590131 | 23405 | -1.786107226 | 0.007821317 |
| 210802_s_at | DIMT1L | DIM1 dimethyladenosine transferase 1-like (S. cerevisiae) | BC002841 | 27292 | 1.856307728 | 0.039463513 |
| 242283_at | DNAH14 | dynein, axonemal, heavy chain 14 | AI076810 | 200095 | 2.646396675 | 0.00809327 |
| 209839_at | DNM3 | dynamin 3 | AL136712 | 26052 | -1.66203 | 0.034340144 |
| 55583_at | DOCK6 | dedicator of cytokinesis 6 | AI198543 | 57572 | 1.782444868 | 0.032839482 |
| 218627_at | DRAM1 | DNA-damage regulated autophagy modulator 1 | NM_018370 | 55332 | 2.158616096 | 0.001796852 |
| 219000_s_at | DSCC1 | defective in sister chromatid cohesion 1 homolog (S. cerevisiae) | NM_024094 | 79075 | 1.633361804 | 0.025882378 |
| 203367_at | DUSP14 | dual specificity phosphatase 14 | NM_007026 | 11072 | 1.638412992 | 0.040345592 |
| 1558739_at | DUSP16 | Dual specificity phosphatase 16 | R30807 | 80824 | -1.692774919 | 0.035778913 |
| 201537_s_at | DUSP3 | dual specificity phosphatase 3 | BC002682 | 1845 | 1.818540913 | 0.007567291 |
| 201538_s_at | DUSP3 | dual specificity phosphatase 3 | NM_004090 | 1845 | 2.109413073 | 0.015631301 |
| 228033_at | E2F7 | E2F transcription factor 7 | AI341146 | 144455 | 2.815700158 | 0.010618543 |
| 219787_s_at | ECT2 | epithelial cell transforming sequence 2 oncogene | NM_018098 | 1894 | 2.463580125 | 0.03169332 |
| 212650_at | EHBP1 | EH domain binding protein 1 | BF116032 | 23301 | -1.661913828 | 0.036524985 |
| 218935_at | EHD3 | EH-domain containing 3 | NM_014600 | 30845 | 1.628055912 | 0.022015897 |
| 229074_at | EHD4 | EH-domain containing 4 | AI692267 | 30844 | 2.474154912 | 0.010043117 |
| 228967_at | EIF1 | Eukaryotic translation initiation factor 1 | BE964053 | 10209 | -1.711765452 | 0.013281851 |
| 225164_s_at | EIF2AK4 | eukaryotic translation initiation factor 2 alpha kinase 4 | AB037759 | 440275 | 1.632911494 | 0.002767242 |
| 244660_at | ELAVL1 | ELAV (embryonic lethal, abnormal vision, Drosophila)-like 1 (Hu antigen R) | AA746320 | 1994 | -1.639979479 | 0.03393192 |
| 228260_at | ELAVL2 | ELAV (embryonic lethal, abnormal vision, Drosophila)-like 2 (Hu antigen B) | AL161628 | 1993 | -1.801106242 | 0.031421319 |
| 234464_s_at | EME1 | essential meiotic endonuclease 1 homolog 1 (S. pombe) | AK021607 | 146956 | 1.651419815 | 0.012261013 |
| 234465_at | EME1 | essential meiotic endonuclease 1 homolog 1 (S. pombe) | AK021607 | 146956 | 1.945113608 | 8.65704E-05 |
| 242443_at | EML5 | Echinoderm microtubule associated protein like 5 | AW026978 | 161436 | -1.851328872 | 0.04814206 |
| 210839_s_at | ENPP2 | ectonucleotide pyrophosphatase/phosphodiesterase 2 | D45421 | 5168 | 2.855347318 | 0.003003257 |
| 201719_s_at | EPB41L2 | erythrocyte membrane protein band 4.1-like 2 | NM_001431 | 2037 | 1.924003359 | 0.013922766 |
| 201718_s_at | EPB41L2 | erythrocyte membrane protein band 4.1-like 2 | BF511685 | 2037 | 2.050875881 | 0.041269296 |
| 203499_at | EPHA2 | EPH receptor A2 | NM_004431 | 1969 | 2.424425605 | 0.001165205 |
| 226213_at | ERBB3 | v-erb-b2 erythroblastic leukemia viral oncogene homolog 3 (avian) | AV681807 | 2065 | 1.631751611 | 0.046721113 |
| 202454_s_at | ERBB3 | v-erb-b2 erythroblastic leukemia viral oncogene homolog 3 (avian) | NM_001982 | 2065 | 1.773372824 | 0.021585988 |
| 233498_at | ERBB4 | v-erb-a erythroblastic leukemia viral oncogene homolog 4 (avian) | AK024204 | 2066 | -1.849063588 | 0.044751904 |
| 214053_at | ERBB4 | v-erb-a erythroblastic leukemia viral oncogene homolog 4 (avian) | AW772192 | 2066 | -1.829124545 | 0.03006112 |
| 219650_at | ERCC6L | excision repair cross-complementing rodent repair deficiency, complementation group 6-like | NM_017669 | 54821 | 2.322206475 | 0.009215424 |
| 203643_at | ERF | Ets2 repressor factor | NM_006494 | 2077 | 1.777399029 | 0.022192981 |
| 231852_at | ERI1 | exoribonuclease 1 | BE779448 | 90459 | 1.702944137 | 0.024116447 |
| 213365_at | ERI2 | ERI1 exoribonuclease family member 2 | N64622 | 112479 | 1.894749125 | 7.45492E-05 |
| 241252_at | ESCO2 | establishment of cohesion 1 homolog 2 (S. cerevisiae) | AI732824 | 157570 | 1.95054012 | 0.002373765 |
| 202862_at | FAH | fumarylacetoacetate hydrolase (fumarylacetoacetase) | NM_000137 | 2184 | 1.818575103 | 0.014428939 |
| 213861_s_at | FAM119B | family with sequence similarity 119, member B | N67741 | 25895 | 1.669564502 | 0.001884667 |
| 225362_at | FAM122B | family with sequence similarity 122B | AI341165 | 159090 | 2.042146025 | 0.000653455 |
| 230496_at | FAM123A | family with sequence similarity 123A | BE046923 | 219287 | -1.923919851 | 0.004101629 |
| 230067_at | FAM124A | Family with sequence similarity 124A | AA151659 | 220108 | -1.836314244 | 0.015729032 |
| 1553715_s_at | FAM195A | family with sequence similarity 195, member A | NM_032371 | 84331 | 1.626723799 | 0.027149387 |
| 225861_at | FAM195A | family with sequence similarity 195, member A | AW001250 | 84331 | 1.665957042 | 0.015711896 |
| 235193_at | FAM214A | KIAA1370 | BG036618 | 56204 | -1.696991867 | 0.002106747 |
| 227410_at | FAM43A | family with sequence similarity 43, member A | AW264102 | 131583 | 1.834704315 | 0.022474075 |
| 229518_at | FAM46B | family with sequence similarity 46, member B | AA531023 | 115572 | 2.196606334 | 0.003607025 |
| 234944_s_at | FAM54A | family with sequence similarity 54, member A | AL138828 | 113115 | 3.648781116 | 9.44681E-05 |
| 228069_at | FAM54A | family with sequence similarity 54, member A | AL138828 | 113115 | 6.662661425 | 1.18234E-05 |
| 225834_at | FAM72A /// FAM72B /// FAM72C /// FAM72D | family with sequence similarity 72, member A /// family with sequence similarity 72, member B /// family with sequence similarity 72, member C /// family with sequence similarity 72, member D | AL135396 | 652689 /// 653573 /// 653594 /// 653820 | 2.942238175 | 0.000174134 |
| 65585_at | FAM86B1 | family with sequence similarity 86, member B1 | AA527515 | 55199 /// 648808 /// 653113 /// 653333 /// 653726 /// 692099 /// 85002 | 1.831225517 | 0.005306222 |
| 223545_at | FANCD2 | Fanconi anemia, complementation group D2 | AF340183 | 2177 | 2.370963962 | 0.001560491 |
| 242560_at | FANCD2 | Fanconi anemia, complementation group D2 | AA579890 | 2177 | 3.165562166 | 0.001905195 |
| 213008_at | FANCI | Fanconi anemia, complementation group I | BG403615 | 55215 | 2.244947572 | 0.013789667 |
| 213007_at | FANCI | Fanconi anemia, complementation group I | W74442 | 55215 | 2.834618923 | 0.002722797 |
| 242711_x_at | FANCM | Fanconi anemia, complementation group M | AI745662 | 57697 | 2.027416569 | 0.012172837 |
| 234733_s_at | FANCM | Fanconi anemia, complementation group M | AK001672 | 57697 | 2.272827711 | 0.009614794 |
| 204780_s_at | FAS | Fas (TNF receptor superfamily, member 6) | AA164751 | 355 | 4.069673501 | 6.73195E-05 |
| 204781_s_at | FAS | Fas (TNF receptor superfamily, member 6) | NM_000043 | 355 | 4.25134576 | 1.59E-06 |
| 216252_x_at | FAS | Fas (TNF receptor superfamily, member 6) | Z70519 | 355 | 4.604542456 | 0.000757871 |
| 215719_x_at | FAS | Fas (TNF receptor superfamily, member 6) | X83493 | 355 | 5.266803452 | 0.000754527 |
| 1553798_a_at | FBXL13 | F-box and leucine-rich repeat protein 13 | NM_145032 | 222235 | 2.119686329 | 0.000171957 |
| 225736_at | FBXO22 | F-box protein 22 | BE966247 | 26263 | 1.64775459 | 0.011327881 |
| 225734_at | FBXO22 | F-box protein 22 | AW294765 | 26263 | 1.681736274 | 0.013331356 |
| 225737_s_at | FBXO22 | F-box protein 22 | BE966247 | 26263 | 1.714997228 | 0.021768046 |
| 207813_s_at | FDXR | ferredoxin reductase | NM_004110 | 2232 | 2.485327253 | 0.001196392 |
| 200894_s_at | FKBP4 | FK506 binding protein 4, 59kDa | AA894574 | 2288 | 1.636385188 | 0.037731517 |
| 205511_at | FLJ10038 | hypothetical protein FLJ10038 | NM_017976 | 55056 | -1.938254407 | 0.018407751 |
| 1559964_at | FLJ38717 | FLJ38717 protein | AK096036 | 401261 | -1.692335689 | 0.038131412 |
| 1555973_at | FLJ39051 | Hypothetical gene supported by AK096370 | BU617042 | 399972 | 1.699259008 | 0.00843309 |
| 242310_at | FNBP1L | Formin binding protein 1-like | AA665058 | 54874 | -2.268766374 | 0.037885934 |
| 202580_x_at | FOXM1 | forkhead box M1 | NM_021953 | 2305 | 3.421329533 | 0.003293409 |
| 1558515_at | FTX | non-protein coding RNA 182 | AK057701 | 100302692 | -2.015560584 | 0.005797926 |
| 223120_at | FUCA2 | fucosidase, alpha-L- 2, plasma | BC003060 | 2519 | 2.107723396 | 0.018162029 |
| 205565_s_at | FXN | frataxin | NM_000144 | 2395 | 1.977070879 | 0.015645752 |
| 234974_at | GALM | galactose mutarotase (aldose 1-epimerase) | AI769923 | 130589 | 1.644361478 | 0.01791861 |
| 235256_s_at | GALM | galactose mutarotase (aldose 1-epimerase) | BE788984 | 130589 | 2.055046681 | 0.008045798 |
| 1552474_a_at | GAMT | guanidinoacetate N-methyltransferase | NM_138924 | 2593 | 1.758976736 | 0.005136791 |
| 203282_at | GBE1 | glucan (1,4-alpha-), branching enzyme 1 | NM_000158 | 2632 | 1.631165408 | 0.025366736 |
| 221577_x_at | GDF15 /// LOC100292463 | growth differentiation factor 15 /// similar to growth differentiation factor 15 | AF003934 | 9518 | 4.327630501 | 0.000819992 |
| 228286_at | GEN1 | Gen homolog 1, endonuclease (Drosophila) | AK025489 | 348654 | 2.434332237 | 0.002615146 |
| 215380_s_at | GGCT | gamma-glutamylcyclotransferase | AK021779 | 79017 | 1.826839715 | 0.002149329 |
| 203560_at | GGH | gamma-glutamyl hydrolase (conjugase, folylpolygammaglutamyl hydrolase) | NM_003878 | 8836 | 2.456407638 | 0.013604819 |
| 206102_at | GINS1 | GINS complex subunit 1 (Psf1 homolog) | NM_021067 | 9837 | 2.223015448 | 0.018101999 |
| 226136_at | GLIPR1 | GLI pathogenesis-related 1 | N32834 | 11010 | 1.976751429 | 0.006042573 |
| 203159_at | GLS | glutaminase | NM_014905 | 2744 | -1.701733271 | 0.005117965 |
| 218350_s_at | GMNN | geminin, DNA replication inhibitor | NM_015895 | 51053 | 1.75686157 | 0.031248675 |
| 224634_at | GPATCH4 | G patch domain containing 4 | AI911518 | 54865 | 1.672106953 | 0.038589595 |
| 224632_at | GPATCH4 | G patch domain containing 4 | BE794289 | 54865 | 1.753959121 | 0.01912111 |
| 236024_at | GPM6A | glycoprotein M6A | AW136286 | 2823 | -1.92001872 | 0.019733976 |
| 200736_s_at | GPX1 | glutathione peroxidase 1 | NM_000581 | 2876 | 1.668820879 | 0.006806075 |
| 222830_at | GRHL1 | grainyhead-like 1 (Drosophila) | BE566136 | 29841 | -2.226678054 | 0.004784081 |
| 211520_s_at | GRIA1 | glutamate receptor, ionotropic, AMPA 1 | M64752 | 2890 | 1.706938197 | 0.040960899 |
| 223759_s_at | GSG2 | germ cell associated 2 (haspin) | AB039834 | 83903 | 2.183676089 | 0.007968479 |
| 235387_at | GSTCD | glutathione S-transferase, C-terminal domain containing | AA740875 | 79807 | 1.83849305 | 0.017828961 |
| 239390_at | GTDC1 | glycosyltransferase-like domain containing 1 | BE327650 | 79712 | -1.688178324 | 0.018143111 |
| 1555439_at | GTF3C3 | general transcription factor IIIC, polypeptide 3, 102kDa | AF465407 | 9330 | 2.054558259 | 0.010902785 |
| 204317_at | GTSE1 | G-2 and S-phase expressed 1 | BF305380 | 51512 | 2.104872766 | 0.025491808 |
| 215942_s_at | GTSE1 | G-2 and S-phase expressed 1 | BF973178 | 51512 | 2.52974064 | 0.018183013 |
| 204318_s_at | GTSE1 | G-2 and S-phase expressed 1 | NM_016426 | 51512 | 2.572992357 | 0.038961864 |
| 215599_at | GUSBP3 | glucuronidase, beta pseudogene 3 | X83300 | 11039 /// 643367 /// 643373 /// 652924 /// 653869 | -2.036415157 | 0.036930249 |
| 230664_at | H2BFXP | H2B histone family, member X, pseudogene | H09657 | 286527 | -2.003414407 | 0.01533636 |
| 223541_at | HAS3 | hyaluronan synthase 3 | AF232772 | 3038 | 1.949343112 | 0.008891399 |
| 233655_s_at | HAUS6 | HAUS augmin-like complex, subunit 6 | AK022964 | 54801 | 1.96626118 | 0.008848631 |
| 207891_s_at | HAUS7 /// TREX2 | HAUS augmin-like complex, subunit 7 /// three prime repair exonuclease 2 | NM_017518 | 11219 /// 55559 | 1.805026815 | 0.042888766 |
| 228741_s_at | HCN3 | hyperpolarization activated cyclic nucleotide-gated potassium channel 3 | AA569959 | 57657 | -1.723558344 | 0.007647204 |
| 203259_s_at | HDDC2 | HD domain containing 2 | BC001671 | 51020 | 1.65401568 | 0.009705186 |
| 203260_at | HDDC2 | HD domain containing 2 | NM_016063 | 51020 | 1.669453932 | 0.021530886 |
| 223556_at | HELLS | helicase, lymphoid-specific | AF155827 | 3070 | 1.828492081 | 0.038640453 |
| 201944_at | HEXB | hexosaminidase B (beta polypeptide) | NM_000521 | 3074 | 1.635897251 | 0.032700785 |
| 214463_x_at | HIST1H4J | histone cluster 1, H4j | NM_003541 | 8362 /// 8363 | -1.716692933 | 0.004369851 |
| 208580_x_at | HIST1H4J /// HIST1H4K | histone cluster 1, H4j /// histone cluster 1, H4k | NM_021968 | 8362 /// 8363 | -1.727713597 | 0.009030654 |
| 214918_at | HNRNPM | heterogeneous nuclear ribonucleoprotein M | AK024911 | 4670 | -1.705584667 | 0.001518461 |
| 235603_at | HNRNPU | heterogeneous nuclear ribonucleoprotein U (scaffold attachment factor A) | N95466 | 3192 | -1.756794881 | 0.041860385 |
| 205543_at | HSPA4L | heat shock 70kDa protein 4-like | NM_014278 | 22824 | 2.200861121 | 0.02596009 |
| 201841_s_at | HSPB1 | heat shock 27kDa protein 1 | NM_001540 | 3315 | 1.816789051 | 0.038817654 |
| 243027_at | IGSF5 | immunoglobulin superfamily, member 5 | AI824021 | 150084 | 1.955727223 | 0.032088612 |
| 235202_x_at | IKBIP | IKBKB interacting protein | BG498328 | 121457 | 2.472690757 | 0.026527955 |
| 236249_at | IKBIP | IKBKB interacting protein | BF057681 | 121457 | 2.676134993 | 0.005661394 |
| 227295_at | IKBIP | IKBKB interacting protein | AW182575 | 121457 | 3.44361572 | 0.007354248 |
| 231152_at | INO80D | INO80 complex subunit D | AW452971 | 54891 | -1.6872063 | 0.006788172 |
| 226450_at | INSR | insulin receptor | AV703054 | 3643 | -1.654380641 | 0.001312168 |
| 234304_s_at | IPO11 | importin 11 | AL162083 | 51194 | 1.742611755 | 0.025453153 |
| 229538_s_at | IQGAP3 | IQ motif containing GTPase activating protein 3 | AW271106 | 128239 | 2.308605795 | 0.009023509 |
| 227314_at | ITGA2 | integrin, alpha 2 (CD49B, alpha 2 subunit of VLA-2 receptor) | N95414 | 3673 | 1.715245386 | 0.039319227 |
| 221778_at | JHDM1D | jumonji C domain containing histone demethylase 1 homolog D (S. cerevisiae) | BE217882 | 80853 | -2.24505147 | 0.022334746 |
| 225806_at | JUB | jub, ajuba homolog (Xenopus laevis) | AI289311 | 84962 | 1.718565794 | 0.024759609 |
| 231855_at | KIAA1524 | KIAA1524 | AB040957 | 57650 | 2.416306386 | 0.000714534 |
| 1553810_a_at | KIAA1524 | KIAA1524 | NM_020890 | 57650 | 2.511197578 | 0.003945163 |
| 206364_at | KIF14 | kinesin family member 14 | NM_014875 | 9928 | 2.758326397 | 0.027686147 |
| 236641_at | KIF14 | kinesin family member 14 | AW183154 | 9928 | 2.994025634 | 0.034449662 |
| 219306_at | KIF15 | kinesin family member 15 | NM_020242 | 56992 | 2.791835931 | 0.005056473 |
| 221258_s_at | KIF18A | kinesin family member 18A | NM_031217 | 81930 | 2.576390924 | 0.028486911 |
| 205235_s_at | KIF20B | kinesin family member 20B | NM_016195 | 9585 | 1.925785666 | 0.011481113 |
| 202183_s_at | KIF22 | kinesin family member 22 | NM_007317 | 3835 | 1.695337384 | 0.027467447 |
| 201212_at | LGMN | legumain | D55696 | 5641 | 1.629754569 | 0.017127837 |
| 202726_at | LIG1 | ligase I, DNA, ATP-dependent | NM_000234 | 3978 | 2.027869354 | 0.00402989 |
| 235039_x_at | LIN9 | lin-9 homolog (C. elegans) | BF697734 | 286826 | 1.961761639 | 0.004099873 |
| 1560290_at | LOC100129895 | Hypothetical protein LOC100129895 | BC041650 | 23589 | -1.809059781 | 0.023114468 |
| 242193_at | LOC100130155 | hypothetical protein LOC100130155 | D59963 | 100130155 | -2.714006329 | 0.002382279 |
| 238661_at | LOC100130155 | hypothetical protein LOC100130155 | AI374740 | 100130155 | -2.633197473 | 0.003991405 |
| 238936_at | LOC100131199 | transmembrane protein 178-like | AV722628 |  | -1.708283004 | 0.045584409 |
| 228381_at | LOC100287628 | Hypothetical protein LOC100287628 | AV716964 | 80063 | 1.702177809 | 0.002671109 |
| 215109_at | LOC100288007 | hypothetical protein LOC100288007 | R02172 | 57238 | -1.714426124 | 0.020051441 |
| 215123_at | LOC100288332 /// LOC100288583 /// NPIPL3 | similar to acyl-CoA synthetase medium-chain family member 2 /// hypothetical protein LOC100288583 /// nuclear pore complex interacting protein-like 3 | AL049250 | 440345 | -2.284922522 | 0.004493942 |
| 1558750_a_at | LOC100288693 | Hypothetical protein LOC100288693 | BG109249 |  | 2.264998612 | 0.018726643 |
| 1557038_s_at | LOC100289373 | similar to hCG2041645 | AK097488 |  | 1.669120358 | 0.008582307 |
| 238953_at | LOC100506325 | Sp2 transcription factor | AA993833 | 6668 | 2.119696837 | 0.007228448 |
| 243134_at | LOC100507217 | Hypothetical LOC440309 | AW190862 | 440309 | -2.062927292 | 0.005785477 |
| 1556194_a_at | LOC100507455 | hypothetical LOC100507455 | BC042959 |  | 1.706293563 | 0.024065043 |
| 240616_at | LOC100507636 | hypothetical LOC100507636 | AV699953 |  | 1.895503893 | 0.029468971 |
| 232192_at | LOC153811 | hypothetical protein LOC153811 | AU145402 | 153811 | -2.029037201 | 0.032893333 |
| 1562527_at | LOC283027 | hypothetical protein LOC283027 | AF519622 | 283027 | 2.054666119 | 0.002694243 |
| 228160_at | LOC339290 | hypothetical LOC339290 | AI433706 | 400642 | -1.780919005 | 0.004865121 |
| 239466_at | LOC344595 | hypothetical LOC344595 | AA463827 | 344595 | 1.631414147 | 0.024887781 |
| 238790_at | LOC374443 | CLR pseudogene | BE738988 | 374443 | 1.686664588 | 0.00653144 |
| 231470_at | LOC400680 | Hypothetical gene supported by AK097381; BC040866 | BE138486 | 400680 | 3.722883126 | 0.000393342 |
| 243780_at | LOC643792 | CDNA FLJ46553 fis, clone THYMU3038879 | AW575863 | 643792 | 1.994648784 | 0.010369112 |
| 241838_at | LOC644112 | Similar to splicing factor 3b, subunit 4 | AA699443 | 644112 | 2.384438627 | 0.037022978 |
| 1560258_a_at | LOC646906 | Similar to Zinc finger protein 254 (Bone marrow zinc finger 5) (BMZF-5) (Hematopoietic cell derived zinc finger protein 1) (HD-ZNF1) | BC035780 | 646906 | 1.977766835 | 0.000666122 |
| 215215_s_at | LOC81691 | exonuclease NEF-sp | AC004381 | 81691 | 1.666093473 | 0.008435435 |
| 208107_s_at | LOC81691 | exonuclease NEF-sp | NM_030941 | 81691 | 1.772654706 | 0.003756617 |
| 206723_s_at | LPAR2 | lysophosphatidic acid receptor 2 | AF011466 | 9170 | -2.022235773 | 0.004210856 |
| 208433_s_at | LRP8 | low density lipoprotein receptor-related protein 8, apolipoprotein e receptor | NM_017522 | 7804 | -1.729255126 | 0.01419891 |
| 241599_at | LSM11 | LSM11, U7 small nuclear RNA associated | AW014922 | 134353 | 1.657646878 | 0.005113718 |
| 202736_s_at | LSM4 | LSM4 homolog, U6 small nuclear RNA associated (S. cerevisiae) | AA112507 | 25804 | 1.704058616 | 0.036219769 |
| 229193_at | LUC7L3 | LUC7-like 3 (S. cerevisiae) | AA005430 | 51747 | -2.529017259 | 0.039306674 |
| 242389_at | LUC7L3 | LUC7-like 3 (S. cerevisiae) | BE887449 | 51747 | -2.508621383 | 0.000931468 |
| 241792_x_at | LUC7L3 | LUC7-like 3 (S. cerevisiae) | N36160 | 51747 | -1.827162417 | 0.010922718 |
| 201744_s_at | LUM | lumican | NM_002345 | 4060 | 3.705693409 | 0.003417956 |
| 1554768_a_at | MAD2L1 | MAD2 mitotic arrest deficient-like 1 (yeast) | AF394735 | 4085 | 2.680174105 | 0.021487074 |
| 223578_x_at | MALAT1 | metastasis associated lung adenocarcinoma transcript 1 (non-protein coding) | AF113016 | 29005 | -1.967298945 | 0.024305321 |
| 208116_s_at | MAN1A1 | mannosidase, alpha, class 1A, member 1 | NM_005907 | 4121 | 1.628379918 | 0.038691319 |
| 1558732_at | MAP4K4 | mitogen-activated protein kinase kinase kinase kinase 4 | AK074900 | 9448 | -2.167711376 | 0.013233609 |
| 211081_s_at | MAP4K5 | mitogen-activated protein kinase kinase kinase kinase 5 | Z25426 | 11183 | -1.760804055 | 0.035754141 |
| 229664_at | MAPK8 | mitogen-activated protein kinase 8 | AI379407 | 5599 | -2.143003976 | 0.021342456 |
| 202787_s_at | MAPKAPK3 | mitogen-activated protein kinase-activated protein kinase 3 | U43784 | 7867 | 1.624816998 | 0.026195432 |
| 228468_at | MASTL | microtubule associated serine/threonine kinase-like | BF108964 | 84930 | 2.480532282 | 0.011390467 |
| 213288_at | MBOAT2 | membrane bound O-acyltransferase domain containing 2 | AI761250 | 129642 | -1.792755372 | 0.002195105 |
| 243552_at | MBTD1 | mbt domain containing 1 | AW008914 | 54799 | -1.856434886 | 0.00891117 |
| 209086_x_at | MCAM | melanoma cell adhesion molecule | BE964361 | 4162 | 2.317935199 | 0.003066627 |
| 211340_s_at | MCAM | melanoma cell adhesion molecule | M28882 | 4162 | 2.477774539 | 0.000693728 |
| 210869_s_at | MCAM | melanoma cell adhesion molecule | M29277 | 4162 | 2.578950009 | 0.006525174 |
| 209087_x_at | MCAM | melanoma cell adhesion molecule | AF089868 | 4162 | 2.662655673 | 0.001388485 |
| 220651_s_at | MCM10 | minichromosome maintenance complex component 10 | NM_018518 | 55388 | 2.562089238 | 0.030069033 |
| 224320_s_at | MCM8 | minichromosome maintenance complex component 8 | BC005170 | 84515 | 1.991938403 | 0.033921273 |
| 220122_at | MCTP1 | multiple C2 domains, transmembrane 1 | NM_024717 | 79772 | 1.909692852 | 0.028259716 |
| 235740_at | MCTP1 | multiple C2 domains, transmembrane 1 | BG250585 | 79772 | 1.948452427 | 0.028606508 |
| 229711_s_at | MDM2 | Mdm2 p53 binding protein homolog (mouse) | AA902480 | 84825 | 1.871447722 | 0.013801174 |
| 217542_at | MDM2 | Mdm2 p53 binding protein homolog (mouse) | BE930512 | 1368 | 2.040224443 | 0.000210758 |
| 204027_s_at | METTL1 | methyltransferase like 1 | NM_005371 | 4234 | 2.041600636 | 0.000329335 |
| 231736_x_at | MGST1 | microsomal glutathione S-transferase 1 | NM_020300 | 4257 | 2.322681165 | 0.035019679 |
| 1565162_s_at | MGST1 | microsomal glutathione S-transferase 1 | D16947 | 4257 | 2.649902213 | 0.027425275 |
| 218376_s_at | MICAL1 | microtubule associated monoxygenase, calponin and LIM domain containing 1 | NM_022765 | 64780 | -1.805184845 | 0.017708858 |
| 213188_s_at | MINA | MYC induced nuclear antigen | AI823896 | 84864 | 1.678278688 | 0.005312046 |
| 213189_at | MINA | MYC induced nuclear antigen | BE966695 | 84864 | 1.780598023 | 0.011936796 |
| 226630_at | MIS18BP1 | chromosome 14 open reading frame 106 | BF062175 | 55320 | 2.24674661 | 0.036337645 |
| 232940_s_at | MLL3 | myeloid/lymphoid or mixed-lineage leukemia 3 | AK025911 | 58508 | -1.689229643 | 0.046498057 |
| 230622_at | MLLT4 | myeloid/lymphoid or mixed-lineage leukemia (trithorax homolog, Drosophila); translocated to, 4 | BE552393 | 4301 | -2.462020132 | 0.045452751 |
| 224685_at | MLLT4 | myeloid/lymphoid or mixed-lineage leukemia (trithorax homolog, Drosophila); translocated to, 4 | AI675354 | 4301 | -1.69149625 | 0.021584051 |
| 223700_at | MND1 | meiotic nuclear divisions 1 homolog (S. cerevisiae) | AY028916 | 84057 | 3.536774608 | 0.001043016 |
| 219703_at | MNS1 | meiosis-specific nuclear structural 1 | NM_018365 | 55329 | 2.970820719 | 0.001659922 |
| 205429_s_at | MPP6 | membrane protein, palmitoylated 6 (MAGUK p55 subfamily member 6) | NM_016447 | 51678 | 1.959105617 | 0.009542019 |
| 205395_s_at | MRE11A | MRE11 meiotic recombination 11 homolog A (S. cerevisiae) | NM_005590 | 4361 | 2.293127668 | 0.000756942 |
| 203152_at | MRPL40 | mitochondrial ribosomal protein L40 | NM_003776 | 64976 | 1.656806551 | 0.00474561 |
| 224869_s_at | MRPS25 | mitochondrial ribosomal protein S25 | AW341440 | 64432 | 1.629327575 | 0.012664595 |
| 231094_s_at | MTHFD1L | methylenetetrahydrofolate dehydrogenase (NADP+ dependent) 1-like | AL035086 | 25902 | 1.787713494 | 0.034213576 |
| 240271_at | MTMR3 | Myotubularin related protein 3 | AL038191 | 8897 | -1.82834317 | 0.035221119 |
| 242996_at | MTRF1 | mitochondrial translational release factor 1 | AI341686 | 9617 | 2.188953784 | 0.002366218 |
| 213906_at | MYBL1 | v-myb myeloblastosis viral oncogene homolog (avian)-like 1 | AW592266 | 4603 | 2.212964959 | 0.008271947 |
| 1557720_s_at | MYO16 | myosin XVI | BC041343 | 23026 | 2.162937237 | 0.002438731 |
| 218966_at | MYO5C | myosin VC | NM_018728 | 55930 | 1.920291325 | 0.016517066 |
| 201798_s_at | MYOF | myoferlin | NM_013451 | 26509 | 2.66827357 | 0.029076335 |
| 1565743_at | N4BP2L2 | Phosphonoformate immuno-associated protein 5 | BG545582 | 10443 | -1.667965905 | 0.001495244 |
| 202944_at | NAGA | N-acetylgalactosaminidase, alpha- | NM_000262 | 4668 | 1.627673268 | 0.020987828 |
| 218189_s_at | NANS | N-acetylneuraminic acid synthase | NM_018946 | 54187 | 1.725532903 | 0.019892761 |
| 242639_at | NARG2 | NMDA receptor regulated 2 | AW340004 | 79664 | 1.713849255 | 0.01254896 |
| 231532_at | NCAM1 | Neural cell adhesion molecule 1 | BF591692 | 4684 | -2.21681108 | 0.000309399 |
| 212843_at | NCAM1 | neural cell adhesion molecule 1 | AA126505 | 4684 | -1.984728132 | 0.005314909 |
| 227394_at | NCAM1 | neural cell adhesion molecule 1 | W94001 | 4684 | -1.983303162 | 0.001176199 |
| 212949_at | NCAPH | non-SMC condensin I complex, subunit H | D38553 | 23397 | 2.307896977 | 0.039657328 |
| 225847_at | NCEH1 | neutral cholesterol ester hydrolase 1 | AB037784 | 57552 | 2.739759434 | 0.003484627 |
| 221805_at | NEFL | neurofilament, light polypeptide | AL537457 | 4747 | 6.043135903 | 0.019906534 |
| 219502_at | NEIL3 | nei endonuclease VIII-like 3 (E. coli) | NM_018248 | 55247 | 2.757337161 | 0.000463366 |
| 1552309_a_at | NEXN | nexilin (F actin binding protein) | NM_144573 | 91624 | 1.827160237 | 0.002432777 |
| 226103_at | NEXN | nexilin (F actin binding protein) | AF114264 | 91624 | 2.116070969 | 0.001880975 |
| 230791_at | NFIB | Nuclear factor I/B | AU146924 | 4781 | -2.032237123 | 0.028212313 |
| 218133_s_at | NIF3L1 | NIF3 NGG1 interacting factor 3-like 1 (S. pombe) | NM_021824 | 60491 | 1.738572957 | 0.000669013 |
| 203045_at | NINJ1 | ninjurin 1 | NM_004148 | 4814 | 1.796878244 | 0.000589981 |
| 242352_at | NIPBL | Nipped-B homolog (Drosophila) | AW272262 | 25836 | -1.644041393 | 0.045808751 |
| 205204_at | NMB | neuromedin B | NM_021077 | 4828 | 2.558754636 | 0.003587865 |
| 201577_at | NME1 | non-metastatic cells 1, protein (NM23A) expressed in | NM_000269 | 4830 | 1.784313109 | 0.019539344 |
| 201268_at | NME1-NME2 /// NME2 | NME1-NME2 readthrough /// non-metastatic cells 2, protein (NM23B) expressed in | NM_002512 | 4831 /// 654364 | 1.841839016 | 0.005150291 |
| 214321_at | NOV | nephroblastoma overexpressed gene | BF440025 | 4856 | 5.024096375 | 0.016505848 |
| 238844_s_at | NPHP1 | nephronophthisis 1 (juvenile) | BF216535 | 4867 | 1.885543387 | 0.003978616 |
| 222344_at | NREP | Chromosome 5 open reading frame 13 | AW972765 | 9315 | -1.965815073 | 0.048293153 |
| 218768_at | NUP107 | nucleoporin 107kDa | NM_020401 | 57122 | 1.809101234 | 0.005987169 |
| 225470_at | NUP35 | nucleoporin 35kDa | AL529634 | 129401 | 1.627136419 | 0.009300701 |
| 218622_at | NUP37 | nucleoporin 37kDa | NM_024057 | 79023 | 1.746630868 | 0.005276564 |
| 233085_s_at | OBFC2A | oligonucleotide/oligosaccharide-binding fold containing 2A | AV734843 | 64859 | 2.684088325 | 7.96751E-05 |
| 213599_at | OIP5 | Opa interacting protein 5 | BE045993 | 11339 | 3.920647537 | 0.001438159 |
| 219105_x_at | ORC6L | origin recognition complex, subunit 6 like (yeast) | NM_014321 | 23594 | 2.111301391 | 0.020173676 |
| 204024_at | OSGIN2 | oxidative stress induced growth inhibitor family member 2 | NM_004337 | 734 | 1.670624766 | 0.012651864 |
| 226621_at | OSMR | oncostatin M receptor | AI133452 | 9180 | 1.705134673 | 0.00771028 |
| 219148_at | PBK | PDZ binding kinase | NM_018492 | 55872 | 3.199609645 | 0.024858731 |
| 233273_at | PBX1 | Pre-B-cell leukemia transcription factor 1 | AU146834 | 5087 | -1.772321999 | 0.048902274 |
| 203557_s_at | PCBD1 | pterin-4 alpha-carbinolamine dehydratase/dimerization cofactor of hepatocyte nuclear factor 1 alpha | NM_000281 | 5092 | 2.17239271 | 0.00551912 |
| 219295_s_at | PCOLCE2 | procollagen C-endopeptidase enhancer 2 | NM_013363 | 26577 | 2.353696636 | 0.047882802 |
| 210170_at | PDLIM3 | PDZ and LIM domain 3 | BC001017 | 27295 | 1.741175457 | 0.039501907 |
| 218691_s_at | PDLIM4 | PDZ and LIM domain 4 | AF153882 | 8572 | 1.864872791 | 0.033294502 |
| 214121_x_at | PDLIM7 | PDZ and LIM domain 7 (enigma) | AA086229 | 9260 | 1.984838653 | 0.031961644 |
| 233442_at | PDZRN3 | PDZ domain containing RING finger 3 | AU147500 | 23024 | -2.072843688 | 0.015234264 |
| 1566551_at | PDZRN3 | PDZ domain containing RING finger 3 | AL137307 | 23024 | -1.853611711 | 0.029360176 |
| 200634_at | PFN1 | profilin 1 | NM_005022 | 5216 | 2.104234802 | 0.02517621 |
| 1557363_a_at | PHIP | pleckstrin homology domain interacting protein | AI969112 | 55023 | 1.764568256 | 0.04880785 |
| 218634_at | PHLDA3 | pleckstrin homology-like domain, family A, member 3 | NM_012396 | 23612 | 1.67232839 | 0.016333947 |
| 1557948_at | PHLDB3 | pleckstrin homology-like domain, family B, member 3 | BC007947 | 284345 /// 653583 | 1.863002527 | 0.016403406 |
| 235980_at | PIK3CA | Phosphoinositide-3-kinase, catalytic, alpha polypeptide | AA767763 | 5290 | -1.752119617 | 0.014858307 |
| 229713_at | PIP4K2A | Phosphatidylinositol-5-phosphate 4-kinase, type II, alpha | AW665227 | 5305 | -1.633901079 | 0.021051429 |
| 244826_at | PITPNB | Phosphatidylinositol transfer protein, beta | R24061 | 23760 | -1.933232181 | 0.033337417 |
| 226299_at | PKN3 | protein kinase N3 | NM_013355 | 29941 | 2.421463021 | 0.002538474 |
| 227148_at | PLEKHH2 | pleckstrin homology domain containing, family H (with MyTH4 domain) member 2 | AI913749 | 130271 | -1.891248085 | 0.005511276 |
| 204958_at | PLK3 | polo-like kinase 3 (Drosophila) | NM_004073 | 1263 | 1.707573845 | 0.003256121 |
| 204887_s_at | PLK4 | polo-like kinase 4 (Drosophila) | NM_014264 | 10733 | 2.47527941 | 0.005816611 |
| 204886_at | PLK4 | polo-like kinase 4 (Drosophila) | AL043646 | 10733 | 2.990376361 | 0.000914063 |
| 213030_s_at | PLXNA2 | plexin A2 | AI688418 | 5362 | -1.796780899 | 0.010052704 |
| 201695_s_at | PNP | purine nucleoside phosphorylase | NM_000270 | 4860 | 1.799209064 | 0.023350253 |
| 234749_s_at | POC1A | POC1 centriolar protein homolog A (Chlamydomonas) | AL117629 | 25886 | 2.850775321 | 0.005276344 |
| 226355_at | POC1A | POC1 centriolar protein homolog A (Chlamydomonas) | AW001089 | 25886 | 2.984504515 | 0.003289686 |
| 222564_at | POGK | pogo transposable element with KRAB domain | AB040946 | 57645 | -1.727313044 | 0.007377508 |
| 231115_at | POLH | polymerase (DNA directed), eta | AI890529 | 54676 | 1.908378208 | 0.003762212 |
| 219510_at | POLQ | polymerase (DNA directed), theta | NM_006596 | 10721 | 3.085732971 | 0.000859438 |
| 201489_at | PPIF | peptidylprolyl isomerase F | BC005020 | 10105 | 2.026513826 | 0.003160373 |
| 201490_s_at | PPIF | peptidylprolyl isomerase F | NM_005729 | 10105 | 2.198099664 | 0.015365339 |
| 235113_at | PPIL5 | peptidylprolyl isomerase (cyclophilin)-like 5 | AA742244 | 122769 | 1.841068467 | 0.009684987 |
| 212527_at | PPPDE2 | PPPDE peptidase domain containing 2 | BF057059 | 27351 | 1.626516333 | 0.01116953 |
| 218009_s_at | PRC1 | protein regulator of cytokinesis 1 | NM_003981 | 9055 | 2.195970671 | 0.034144457 |
| 205277_at | PRDM2 | PR domain containing 2, with ZNF domain | NM_012231 | 7799 | -1.800559525 | 0.010839866 |
| 200844_s_at | PRDX6 | peroxiredoxin 6 | BE869583 | 9588 | 1.64019281 | 0.015306157 |
| 236513_at | PRELID2 | Hypothetical protein MGC21644 | AW770245 | 153768 | 1.717067229 | 0.006772756 |
| 213320_at | PRMT3 | protein arginine methyltransferase 3 | AL551971 | 10196 | 1.626540901 | 0.004047694 |
| 228273_at | PRR11 | proline rich 11 | BG165011 | 55771 | 4.166673549 | 0.002342068 |
| 242875_at | PSEN1 | Presenilin 1 (Alzheimer disease 3) | AI659439 | 5663 | -1.873576694 | 0.037767014 |
| 203405_at | PSMG1 | proteasome (prosome, macropain) assembly chaperone 1 | NM_003720 | 8624 | 1.651984338 | 0.036217094 |
| 201896_s_at | PSRC1 | proline/serine-rich coiled-coil 1 | BC001425 | 84722 | 1.707806272 | 0.030126139 |
| 224937_at | PTGFRN | prostaglandin F2 receptor negative regulator | BF311866 | 5738 | 1.635921547 | 0.009749892 |
| 213362_at | PTPRD | protein tyrosine phosphatase, receptor type, D | N73931 | 5789 | -2.038155535 | 0.001857414 |
| 219412_at | RAB38 | RAB38, member RAS oncogene family | NM_022337 | 23682 | 1.939703917 | 0.01072322 |
| 223417_at | RAD18 | RAD18 homolog (S. cerevisiae) | AF169796 | 56852 | 2.844242445 | 3.61018E-06 |
| 224200_s_at | RAD18 | RAD18 homolog (S. cerevisiae) | AB035274 | 56852 | 4.01791564 | 6.45108E-06 |
| 205024_s_at | RAD51 | RAD51 homolog (RecA homolog, E. coli) (S. cerevisiae) | NM_002875 | 5888 | 2.375940878 | 0.01849127 |
| 206066_s_at | RAD51C | RAD51 homolog C (S. cerevisiae) | NM_002876 | 5889 | 1.773567571 | 0.000416558 |
| 209849_s_at | RAD51C | RAD51 homolog C (S. cerevisiae) | AF029669 | 5889 | 1.872552418 | 0.000172507 |
| 1555340_x_at | RAP1A | RAP1A, member of RAS oncogene family | AB051846 | 5906 | 130.3223823 | 2.64939E-09 |
| 1555339_at | RAP1A | RAP1A, member of RAS oncogene family | AB051846 | 5906 | 164.91688 | 1.30741E-11 |
| 238763_at | RBM20 | RNA binding motif protein 20 | AI539118 | 282996 | 2.035619806 | 0.002684894 |
| 222496_s_at | RBM47 | RNA binding motif protein 47 | AW241742 | 54502 | 1.988235684 | 0.02895357 |
| 218035_s_at | RBM47 | RNA binding motif protein 47 | NM_019027 | 54502 | 2.797602292 | 0.044121508 |
| 235511_at | RBM4B | RNA binding motif protein 4B | BE144058 | 83759 | -1.86403973 | 0.022741303 |
| 208370_s_at | RCAN1 | regulator of calcineurin 1 | NM_004414 | 1827 | 1.83436783 | 0.034579201 |
| 239169_at | RDM1 | RAD52 motif 1 | AA761980 | 201299 | 3.442714452 | 0.00076364 |
| 204365_s_at | REEP1 | receptor accessory protein 1 | NM_022912 | 65055 | -2.092019641 | 0.038618462 |
| 204127_at | RFC3 | replication factor C (activator 1) 3, 38kDa | BC000149 | 5983 | 2.759651982 | 0.000576098 |
| 204128_s_at | RFC3 | replication factor C (activator 1) 3, 38kDa | NM_002915 | 5983 | 3.199437166 | 0.006990071 |
| 242450_at | RGMB | RGM domain family, member B | AW004714 | 285704 | 1.962879044 | 0.015227642 |
| 214449_s_at | RHOQ | ras homolog gene family, member Q | NM_012249 | 23433 | 1.705967962 | 0.043831366 |
| 212122_at | RHOQ | ras homolog gene family, member Q | AW771590 | 23433 /// 284988 | 1.781602735 | 0.026161724 |
| 226456_at | RMI2 | chromosome 16 open reading frame 75 | AW138157 | 116028 | 1.861656692 | 0.021631589 |
| 203022_at | RNASEH2A | ribonuclease H2, subunit A | NM_006397 | 10535 | 1.934979038 | 0.046594799 |
| 243229_at | RNASET2 | Ribonuclease T2 | AI147535 | 8635 | 2.465350992 | 0.01917376 |
| 237062_at | RNF10 | Ring finger protein 10 | BE222109 | 9921 | -1.686364396 | 0.04915309 |
| 204040_at | RNF144A | ring finger protein 144A | NM_014746 | 9781 | -1.758851576 | 0.002412173 |
| 223824_at | RNLS | renalase, FAD-dependent amine oxidase | BC005364 | 55328 | 1.665334307 | 0.022139053 |
| 224617_at | ROD1 | ROD1 regulator of differentiation 1 (S. pombe) | AI735576 | 9991 | -1.909962595 | 0.003481408 |
| 209507_at | RPA3 | replication protein A3, 14kDa | BC005264 | 6119 | 1.858233132 | 0.049234835 |
| 207107_at | RPE65 | retinal pigment epithelium-specific protein 65kDa | NM_000329 | 6121 | 3.853954589 | 0.034048139 |
| 225541_at | RPL22L1 | ribosomal protein L22-like 1 | BE274422 | 200916 /// 440990 | 2.00109349 | 0.000674549 |
| 210115_at | RPL39L | ribosomal protein L39-like | L05096 | 116832 | 1.691982407 | 0.0222325 |
| 213427_at | RPP40 | ribonuclease P/MRP 40kDa subunit | NM_006638 | 10799 | 1.662708135 | 0.028554796 |
| 223342_at | RRM2B | ribonucleotide reductase M2 B (TP53 inducible) | AB036063 | 50484 | 1.744502362 | 0.001987898 |
| 1555501_s_at | RSRC1 | arginine/serine-rich coiled-coil 1 | BC010357 | 51319 | 1.643324755 | 0.013431444 |
| 235354_s_at | RSRC1 | arginine/serine-rich coiled-coil 1 | BG398744 | 51319 | 1.870308238 | 0.033820357 |
| 1555679_a_at | RTN4IP1 | reticulon 4 interacting protein 1 | AF439711 | 84816 | 1.852377841 | 0.005199251 |
| 200660_at | S100A11 | S100 calcium binding protein A11 | NM_005620 | 6282 | 3.373000954 | 0.002936429 |
| 228176_at | S1PR3 | sphingosine-1-phosphate receptor 3 | AA534817 | 1903 | 3.345043516 | 0.000779188 |
| 225509_at | SAP30L | SAP30-like | AI862477 | 56757 | -1.737534001 | 0.000290769 |
| 231895_at | SASS6 | spindle assembly 6 homolog (C. elegans) | AA501453 | 163786 | 1.868572957 | 0.001600851 |
| 223843_at | SCARA3 | scavenger receptor class A, member 3 | AB007830 | 51435 | 1.999128936 | 0.002925091 |
| 1569190_at | SCLT1 | sodium channel and clathrin linker 1 | BC014677 | 132320 | 1.979434168 | 0.005538613 |
| 229057_at | SCN2A | sodium channel, voltage-gated, type II, alpha subunit | BF432956 | 6326 | 2.444855305 | 0.000670904 |
| 228274_at | SDSL | serine dehydratase-like | BE963955 | 113675 | 1.682957004 | 0.019068519 |
| 213666_at | SEPT6 | septin 6 | AK026589 | 23157 | -1.688899281 | 0.000500247 |
| 1555526_a_at | SEPT6 | septin 6 | AF403061 | 23157 | -1.647499184 | 0.034575431 |
| 205352_at | SERPINI1 | serpin peptidase inhibitor, clade I (neuroserpin), member 1 | NM_005025 | 5274 | 2.268021633 | 0.028667284 |
| 218346_s_at | SESN1 | sestrin 1 | NM_014454 | 27244 | 1.658082401 | 0.011171005 |
| 223195_s_at | SESN2 | sestrin 2 | BF131886 | 83667 | 1.824034884 | 0.007958074 |
| 227041_at | SESTD1 | SEC14 and spectrin domains 1 | BE466145 | 91404 | -1.627609663 | 0.045647656 |
| 235611_at | SFRS12 | splicing factor, arginine/serine-rich 12 | AA026666 | 140890 | -1.740839394 | 0.038558205 |
| 1553690_at | SGOL1 | shugoshin-like 1 (S. pombe) | NM_138484 | 151648 | 1.659653961 | 0.032766189 |
| 231938_at | SGOL1 | Shugoshin-like 1 (S. pombe) | AK024292 | 151648 | 2.357892253 | 0.000889619 |
| 230165_at | SGOL2 | shugoshin-like 2 (S. pombe) | N31731 | 151246 | 2.260998953 | 0.047024898 |
| 219493_at | SHCBP1 | SHC SH2-domain binding protein 1 | NM_024745 | 79801 | 5.856466583 | 0.001819422 |
| 1554355_a_at | SIAE | sialic acid acetylesterase | BC040966 | 54414 | 1.89821657 | 0.003387609 |
| 217640_x_at | SKA1 | spindle and kinetochore associated complex subunit 1 | BF038461 | 220134 | 2.569440702 | 0.018967943 |
| 227165_at | SKA3 | spindle and kinetochore associated complex subunit 3 | AI829603 | 221150 | 2.093178168 | 0.038503797 |
| 1557411_s_at | SLC25A43 | solute carrier family 25, member 43 | AK094254 | 203427 | 1.913681714 | 0.045325335 |
| 204430_s_at | SLC2A5 | solute carrier family 2 (facilitated glucose/fructose transporter), member 5 | NM_003039 | 6518 | 2.096269787 | 0.002505398 |
| 203971_at | SLC31A1 | solute carrier family 31 (copper transporters), member 1 | NM_001859 | 1317 | 1.637413325 | 0.046730255 |
| 209713_s_at | SLC35D1 | solute carrier family 35 (UDP-glucuronic acid/UDP-N-acetylgalactosamine dual transporter), member D1 | AB044343 | 23169 | 1.695225623 | 0.036787031 |
| 215169_at | SLC35E2 | solute carrier family 35, member E2 | BE885244 | 9906 | -1.891667399 | 0.002410233 |
| 223748_at | SLC4A11 | solute carrier family 4, sodium borate transporter, member 11 | AF336127 | 83959 | 2.174879559 | 0.000912243 |
| 1555141_a_at | SLC9B1 | Na+/H+ exchanger domain containing 1 | BC022079 | 150159 | 1.738269922 | 0.011414235 |
| 213601_at | SLIT1 | slit homolog 1 (Drosophila) | AB011537 | 6585 | -2.304517718 | 0.027508302 |
| 1565703_at | SMAD4 | SMAD family member 4 | AL832789 | 4089 | -2.083351789 | 0.018954048 |
| 1561973_at | SMARCC2 | SWI/SNF related, matrix associated, actin dependent regulator of chromatin, subfamily c, member 2 | AL833124 | 6601 | -1.82387592 | 0.011820012 |
| 213253_at | SMC2 | structural maintenance of chromosomes 2 | AU154486 | 10592 | 2.735920204 | 0.007621604 |
| 204240_s_at | SMC2 | structural maintenance of chromosomes 2 | NM_006444 | 10592 | 3.109630784 | 0.002176646 |
| 212921_at | SMYD2 | SET and MYND domain containing 2 | AF070592 | 56950 | -1.7333547 | 0.016041895 |
| 242146_at | SNRPA1 | Small nuclear ribonucleoprotein polypeptide A' | AA872471 | 6627 | -1.862037178 | 0.044450745 |
| 1560741_at | SNRPN | small nuclear ribonucleoprotein polypeptide N | AL832250 | 6638 | -1.840439228 | 0.009970813 |
| 213456_at | SOSTDC1 | sclerostin domain containing 1 | AI927000 | 25928 | 2.513768566 | 0.00153065 |
| 204913_s_at | SOX11 | SRY (sex determining region Y)-box 11 | AI360875 | 6664 | -2.14897326 | 0.030160632 |
| 204915_s_at | SOX11 | SRY (sex determining region Y)-box 11 | AB028641 | 6664 | -1.804916412 | 0.025873804 |
| 204914_s_at | SOX11 | SRY (sex determining region Y)-box 11 | AW157202 | 6664 | -1.786449807 | 0.026266641 |
| 1557804_at | SP3 | Sp3 transcription factor | N23846 | 6670 | -1.829914268 | 0.014980727 |
| 238035_at | SP3 | Sp3 transcription factor | N66313 | 6670 | -1.710575252 | 0.0066347 |
| 203145_at | SPAG5 | sperm associated antigen 5 | NM_006461 | 10615 | 2.537801626 | 0.006003607 |
| 229331_at | SPATA18 | spermatogenesis associated 18 homolog (rat) | AI559300 | 132671 | 2.472178279 | 2.56514E-05 |
| 235572_at | SPC24 | SPC24, NDC80 kinetochore complex component, homolog (S. cerevisiae) | AI469788 | 147841 | 3.022570392 | 0.008073295 |
| 209891_at | SPC25 | SPC25, NDC80 kinetochore complex component, homolog (S. cerevisiae) | AF225416 | 57405 | 2.535127665 | 0.040836708 |
| 46256_at | SPSB3 | splA/ryanodine receptor domain and SOCS box containing 3 | AA522670 | 90864 | -1.654367607 | 0.010741923 |
| 216202_s_at | SPTLC2 | serine palmitoyltransferase, long chain base subunit 2 | U15555 | 9517 | 1.658995622 | 0.037074683 |
| 204955_at | SRPX | sushi-repeat-containing protein, X-linked | NM_006307 | 8406 | 2.07469871 | 0.010179054 |
| 230836_at | ST8SIA4 | ST8 alpha-N-acetyl-neuraminide alpha-2,8-sialyltransferase 4 | AI422986 | 7903 | -2.792707072 | 0.002093156 |
| 204595_s_at | STC1 | stanniocalcin 1 | AI300520 | 6781 | -2.162370302 | 0.012814796 |
| 218424_s_at | STEAP3 | STEAP family member 3 | NM_018234 | 55240 | 2.172860389 | 0.005006755 |
| 229513_at | STRBP | spermatid perinuclear RNA binding protein | AK025613 | 55342 | -1.821109364 | 0.034346908 |
| 224724_at | SULF2 | sulfatase 2 | AL133001 | 55959 | 2.736403559 | 0.000917887 |
| 233555_s_at | SULF2 | sulfatase 2 | AL034418 | 55959 | 2.825044662 | 0.002997892 |
| 212144_at | SUN2 | Sad1 and UNC84 domain containing 2 | AL021707 | 25777 | -1.80207565 | 0.030697474 |
| 218619_s_at | SUV39H1 | suppressor of variegation 3-9 homolog 1 (Drosophila) | NM_003173 | 6839 | 1.851499747 | 0.001074045 |
| 222566_at | SUV420H1 | suppressor of variegation 4-20 homolog 1 (Drosophila) | AA056099 | 51111 | -1.710945177 | 0.003058228 |
| 235020_at | TAF4B | TAF4b RNA polymerase II, TATA box binding protein (TBP)-associated factor, 105kDa | AI366784 | 6875 | 1.672219291 | 0.005997768 |
| 220325_at | TAF7L | TAF7-like RNA polymerase II, TATA box binding protein (TBP)-associated factor, 50kDa | NM_024885 | 54457 | 3.162496059 | 6.21729E-07 |
| 205547_s_at | TAGLN | transgelin | NM_003186 | 6876 | 2.535500444 | 0.011072201 |
| 204743_at | TAGLN3 | transgelin 3 | NM_013259 | 29114 | -1.876821094 | 0.014294395 |
| 231972_at | TBC1D7 | TBC1 domain family, member 7 | AK024681 | 51256 | -1.644080592 | 0.049442908 |
| 215164_at | TCF4 | Transcription factor 4 | AL049279 | 6925 | -2.215176102 | 0.035646396 |
| 212382_at | TCF4 | transcription factor 4 | BF433429 | 6925 | -1.912220346 | 0.011917487 |
| 228837_at | TCF4 | transcription factor 4 | BE857360 | 6925 | -1.778603926 | 0.044684331 |
| 219596_at | THAP10 | THAP domain containing 10 | NM_020147 | 56906 | 1.86720936 | 0.000496931 |
| 201449_at | TIA1 | TIA1 cytotoxic granule-associated RNA binding protein | AL567227 | 7072 | -1.691059678 | 0.016197197 |
| 213135_at | TIAM1 | T-cell lymphoma invasion and metastasis 1 | U90902 | 7074 | -1.638274978 | 0.03145571 |
| 215455_at | TIMELESS | timeless homolog (Drosophila) | AK000721 | 8914 | 1.625511073 | 0.006545892 |
| 201666_at | TIMP1 | TIMP metallopeptidase inhibitor 1 | NM_003254 | 7076 | 1.7331187 | 0.023666078 |
| 40837_at | TLE2 | transducin-like enhancer of split 2 (E(sp1) homolog, Drosophila) | M99436 | 7089 | -2.100412514 | 0.009699224 |
| 212770_at | TLE3 | transducin-like enhancer of split 3 (E(sp1) homolog, Drosophila) | AW873621 | 7090 | -1.788103646 | 0.006822637 |
| 206271_at | TLR3 | toll-like receptor 3 | NM_003265 | 7098 | 2.405986018 | 0.007553231 |
| 232068_s_at | TLR4 | toll-like receptor 4 | AF177765 | 7099 | 2.259497669 | 0.020529783 |
| 212619_at | TMEM194A | transmembrane protein 194A | AW205215 | 23306 | 2.595652536 | 0.016191913 |
| 234672_s_at | TMEM48 | transmembrane protein 48 | AL354612 | 55706 | 1.681408235 | 0.035404042 |
| 218073_s_at | TMEM48 | transmembrane protein 48 | NM_018087 | 55706 | 3.256164229 | 0.010656273 |
| 231775_at | TNFRSF10A | tumor necrosis factor receptor superfamily, member 10a | W65310 | 8797 | 1.929790683 | 0.01125936 |
| 204932_at | TNFRSF11B | tumor necrosis factor receptor superfamily, member 11b | BF433902 | 4982 | 1.987592283 | 0.031818617 |
| 210609_s_at | TP53I3 | tumor protein p53 inducible protein 3 | BC000474 | 9540 | 1.842802313 | 0.00679972 |
| 204083_s_at | TPM2 | tropomyosin 2 (beta) | NM_003289 | 7169 | 2.075746446 | 0.027738138 |
| 204079_at | TPST2 | tyrosylprotein sulfotransferase 2 | NM_003595 | 8459 | 1.858999402 | 0.045067023 |
| 230192_at | TRIM13 | tripartite motif-containing 13 | AI472310 | 10206 | -2.341020965 | 0.007846607 |
| 1569142_at | TRIM13 | tripartite motif-containing 13 | BC029514 | 10206 | -2.190444465 | 0.019964424 |
| 229943_at | TRIM13 | tripartite motif-containing 13 | BF939833 | 10206 | -2.048805375 | 0.022395676 |
| 243945_at | TRIM2 | tripartite motif-containing 2 | AI298925 | 23321 | 2.602292592 | 0.017396691 |
| 204033_at | TRIP13 | thyroid hormone receptor interactor 13 | NM_004237 | 9319 | 5.511100566 | 0.000239005 |
| 209129_at | TRIP6 | thyroid hormone receptor interactor 6 | AF000974 | 7205 | 1.689808854 | 0.002310527 |
| 227862_at | TRNP1 | TMF1-regulated nuclear protein 1 | AA037766 | 388610 | 1.800637203 | 0.029621297 |
| 205028_at | TRO | trophinin | NM_016157 | 7216 | -1.895033072 | 0.026202039 |
| 243483_at | TRPM8 | transient receptor potential cation channel, subfamily M, member 8 | AI272941 | 79054 | 2.600195988 | 0.003255043 |
| 240557_at | TSC22D2 | TSC22 domain family, member 2 | N34514 | 9819 | -2.693665957 | 0.021025587 |
| 237057_at | TSGA10 | Testis specific, 10 | AW150991 | 80705 | 2.488329971 | 0.000120288 |
| 231592_at | TSIX | XIST antisense RNA (non-protein coding) | AV646335 | 7503 | -2.170765801 | 0.039927879 |
| 208664_s_at | TTC3 | tetratricopeptide repeat domain 3 | AU131711 | 7267 | -1.786319757 | 0.006920032 |
| 204407_at | TTF2 | transcription termination factor, RNA polymerase II | AF080255 | 8458 | 3.449795708 | 0.000702598 |
| 227141_at | TYW3 | tRNA-yW synthesizing protein 3 homolog (S. cerevisiae) | AW205739 | 127253 | 1.698648489 | 0.022091688 |
| 238462_at | UBASH3B | ubiquitin associated and SH3 domain containing B | AI418293 | 84959 | 1.655224416 | 0.046746405 |
| 217100_s_at | UBXN7 | UBX domain protein 7 | AK026451 | 26043 | -1.70107163 | 0.024028074 |
| 220083_x_at | UCHL5 | ubiquitin carboxyl-terminal hydrolase L5 | NM_016017 | 51377 | 1.692852627 | 0.030121675 |
| 1555561_a_at | UGGT2 | UDP-glucose glycoprotein glucosyltransferase 2 | BC032302 | 55757 | 2.136876006 | 0.033217813 |
| 244133_at | USP49 | ubiquitin specific peptidase 49 | BE258909 | 25862 | -1.966113386 | 0.019873805 |
| 203940_s_at | VASH1 | vasohibin 1 | NM_014909 | 22846 | -1.701538816 | 0.012784127 |
| 219740_at | VASH2 | vasohibin 2 | NM_024749 | 79805 | -2.865869136 | 0.027292285 |
| 235138_at | VPS35 | Vacuolar protein sorting 35 (yeast) | AA565051 | 55737 | -2.248024498 | 0.031635248 |
| 203856_at | VRK1 | vaccinia related kinase 1 | NM_003384 | 7443 | 2.386637889 | 0.000365968 |
| 210102_at | VWA5A | von Willebrand factor A domain containing 5A | BC001234 | 4013 | 1.840193343 | 0.013617084 |
| 242957_at | VWCE | von Willebrand factor C and EGF domains | AI862096 | 220001 | 1.655299187 | 0.030485903 |
| 218512_at | WDR12 | WD repeat domain 12 | NM_018256 | 55759 | 1.635564709 | 0.02820667 |
| 214061_at | WDR67 | WD repeat domain 67 | AI017564 | 93594 | 2.364766623 | 0.004184911 |
| 1556429_a_at | WDR67 | WD repeat domain 67 | BC026969 | 93594 | 2.621368125 | 0.000824907 |
| 200670_at | XBP1 | X-box binding protein 1 | NM_005080 | 7494 | 1.812834088 | 0.028550026 |
| 205072_s_at | XRCC4 | X-ray repair complementing defective repair in Chinese hamster cells 4 | NM_022406 | 7518 | 1.718780709 | 0.02895861 |
| 205071_x_at | XRCC4 | X-ray repair complementing defective repair in Chinese hamster cells 4 | AB017445 | 7518 | 1.960822057 | 0.002981495 |
| 210813_s_at | XRCC4 | X-ray repair complementing defective repair in Chinese hamster cells 4 | BC005259 | 7518 | 2.114004402 | 0.00204244 |
| 226437_at | YIF1B | Yip1 interacting factor homolog B (S. cerevisiae) | AI079540 | 90522 | 1.734761708 | 0.011071526 |
| 217785_s_at | YKT6 | YKT6 v-SNARE homolog (S. cerevisiae) | NM_006555 | 10652 | 1.673115625 | 0.037120862 |
| 228788_at | YPEL1 | yippee-like 1 (Drosophila) | AA425358 | 29799 | -1.770869433 | 0.004057158 |
| 243417_at | ZADH2 | zinc binding alcohol dehydrogenase domain containing 2 | AA704162 | 284273 | -1.644162472 | 0.005541058 |
| 236215_at | ZC2HC1A | Chromosome 8 open reading frame 70 | AI079329 | 51101 | -2.417339958 | 0.021861039 |
| 235593_at | ZEB2 | zinc finger E-box binding homeobox 2 | AL546529 | 9839 | -2.032334494 | 0.011330315 |
| 1554638_at | ZFYVE16 | zinc finger, FYVE domain containing 16 | BC032227 | 9765 | -1.631859543 | 0.03978853 |
| 205739_x_at | ZNF107 | zinc finger protein 107 | NM_016220 | 51427 | 1.894723736 | 0.030369976 |
| 1557384_at | ZNF131 | Zinc finger protein 131 | AL832081 | 7690 | -1.86744839 | 0.006324949 |
| 222227_at | ZNF236 | zinc finger protein 236 | AK000847 | 7776 | 105.0309486 | 8.86302E-08 |
| 206261_at | ZNF239 | zinc finger protein 239 | NM_005674 | 8187 | 1.753626275 | 0.011006578 |
| 1559449_a_at | ZNF254 | Zinc finger protein 254 | BF679633 | 399655 | -1.741800399 | 0.015196219 |
| 243661_at | ZNF273 | zinc finger protein 273 | AW172914 | 10793 | 1.742457785 | 0.018042888 |
| 227613_at | ZNF331 | zinc finger protein 331 | AW450874 | 55422 | 1.777405302 | 0.001154956 |
| 235513_at | ZNF398 | Zinc finger protein 398 | AW131450 | 57541 | -1.716912594 | 0.037609892 |
| 215532_x_at | ZNF492 | zinc finger protein 492 | AB040906 | 148198 /// 649413 | 2.21922677 | 0.008788101 |
| 227195_at | ZNF503 | zinc finger protein 503 | AA603467 | 84858 | 1.752351936 | 0.020748705 |
| 230205_at | ZNF561 | zinc finger protein 561 | BF437602 | 93134 | 1.878981663 | 0.017380293 |
| 239243_at | ZNF638 | zinc finger protein 638 | AA279654 | 27332 | -2.31867109 | 0.030504284 |
| 1557270_at | ZNF69 | Zinc finger protein 69 | AA632049 | 7620 | -1.826021296 | 0.026541767 |
| 227080_at | ZNF697 | zinc finger protein 697 | AW003092 | 90874 | -1.656608097 | 0.045065986 |
| 206557_at | ZNF702P | zinc finger protein 702 (pseudogene) | NM_024924 | 79986 | 1.625364273 | 0.000342111 |
| 235079_at | ZNF704 | zinc finger protein 704 | AW265065 | 619279 | -2.675938302 | 0.044038331 |
| 235905_at | ZNF704 | zinc finger protein 704 | N74530 | 619279 | -2.318303892 | 0.025298055 |
| 223366_at | ZNF704 | zinc finger protein 704 | BC004287 | 619279 | -2.26527493 | 0.003466826 |
| 1569191_at | ZNF826 | zinc finger protein 826 | BC016785 | 664701 | 2.352519461 | 0.035576893 |
| 238952_x_at | ZNF829 | zinc finger protein 829 | BF439163 | 374899 | 1.633024335 | 0.00197662 |
| 1554445_at | ZNF85 | zinc finger protein 85 | BC008688 | 7639 | 1.815489566 | 0.002996811 |
| 1564039_at | ZSCAN23 | zinc finger and SCAN domain containing 23 | AK092117 | 222696 | -2.156331382 | 0.037445817 |
| 222606_at | ZWILCH | Zwilch, kinetochore associated, homolog (Drosophila) | AA824298 | 55055 | 1.982334691 | 0.014095806 |
| 218349_s_at | ZWILCH | Zwilch, kinetochore associated, homolog (Drosophila) | NM_017975 | 55055 | 2.033316143 | 0.028336113 |

LV 2h

| **Probe Set ID** | **Gene Symbol** | **Gene Title** | **Accession number** | **EntrezGene** | **Fold Change** | **PValue** |
| --- | --- | --- | --- | --- | --- | --- |
| 1553220_at | FAM117B | amyotrophic lateral sclerosis 2 (juvenile) chromosome region, candidate 13 | NM_173511 | 150864 | 1.83813236 | 0.002310583 |
| 236026_at | GPATCH2 | G patch domain containing 2 | AA160529 | 55105 | -2.089298842 | 0.012535744 |
| 242224_at | GPATCH2 | G patch domain containing 2 | R40111 | 55105 | -2.048607785 | 0.032981941 |
| 211696_x_at | HBB | hemoglobin, beta /// hemoglobin, beta | AF349114 | 3043 | 1.67462282 | 0.014440881 |
| 208937_s_at | ID1 | inhibitor of DNA binding 1, dominant negative helix-loop-helix protein | D13889 | 3397 | 12.10120867 | 0.00043611 |
| 201565_s_at | ID2 | inhibitor of DNA binding 2, dominant negative helix-loop-helix protein | NM_002166 | 3398 | 1.962906644 | 0.019097019 |
| 201566_x_at | ID2 | inhibitor of DNA binding 2, dominant negative helix-loop-helix protein /// inhibitor of DNA binding 2B, dominant negative helix-loop-helix protein | D13891 | 3398 /// 84099 | 2.134718644 | 0.011062319 |
| 207826_s_at | ID3 | inhibitor of DNA binding 3, dominant negative helix-loop-helix protein | NM_002167 | 3399 | 7.562096797 | 0.009779537 |
| 1556129_at | LOC642533 | Hypothetical protein FLJ31875 | AF086148 | 197320 | 1.995755339 | 0.000844292 |
| 201502_s_at | NFKBIA | nuclear factor of kappa light polypeptide gene enhancer in B-cells inhibitor, alpha | AI078167 | 4792 | 1.970596811 | 0.027368981 |
| 209181_s_at | RABGGTB | Rab geranylgeranyltransferase, beta subunit | U49245 | 5876 | 1.736175687 | 0.00883987 |
| 1555339_at | RAP1A | RAP1A, member of RAS oncogene family | AB051846 | 5906 | 94.57635114 | 5.11992E-09 |
| 1555340_x_at | RAP1A | RAP1A, member of RAS oncogene family | AB051846 | 5906 | 109.5671492 | 1.26112E-10 |
| 210466_s_at | SERBP1 | SERPINE1 mRNA binding protein 1 | BC002488 | 26135 | 1.821403159 | 0.008547039 |
| 207069_s_at | SMAD6 | SMAD, mothers against DPP homolog 6 (Drosophila) | NM_005585 | 4091 | 1.667187695 | 0.031950805 |
| 242245_at | SYDE2 | [Hypothetical protein FLJ13815](http://humanet.scbit.org/temporal_result.jsp?ID=51520) | N90719 | 84144 | 1.967359429 | 0.036986776 |
| 202644_s_at | TNFAIP3 | tumor necrosis factor, alpha-induced protein 3 | NM_006290 | 7128 | 2.716117327 | 0.01283538 |
| 242053_at | TSGA10 | Transcribed locus | AW665279 | 80705 | 2.135333622 | 8.64967E-06 |
| 222227_at | ZNF236 | zinc finger protein 236 | AK000847 | 7776 | 23.80571411 | 2.64866E-05 |

LV 5d

| **Probe Set ID** | **Gene Symbol** | **Gene Title** | **Accession number** | **Entrez Gene** | **Fold change** | **PValue** |
| --- | --- | --- | --- | --- | --- | --- |
| 1554948_at | --- | --- | BC034024 |  | -2.217351128 | 0.02213585 |
| 1558236_at | --- | --- | BC014318 |  | -1.671393895 | 0.006710182 |
| 1559006_at | --- | --- | BC007784 |  | -1.659893577 | 0.012311908 |
| 1559524_at | --- | --- | BU846215 |  | -1.832007175 | 0.015468703 |
| 214862_x_at | --- | --- | AL080082 |  | -1.730619628 | 0.022793629 |
| 215191_at | --- | --- | AW836210 |  | -1.970782411 | 0.032410917 |
| 216565_x_at | --- | --- | AL121994 |  | 1.911908839 | 0.007870438 |
| 227921_at | --- | --- | AI797678 |  | -1.838386241 | 0.044040306 |
| 228694_at | --- | --- | BE670036 |  | -1.659214634 | 0.00055595 |
| 229206_at | --- | --- | BE551650 |  | -1.807004037 | 0.033304967 |
| 230356_at | --- | --- | AW014743 |  | 2.855831045 | 0.044494328 |
| 231438_x_at | --- | --- | R20640 |  | 2.363726487 | 0.004304007 |
| 233068_at | --- | --- | AK023264 |  | -1.690091596 | 0.034386338 |
| 235028_at | --- | --- | BG288330 |  | -2.426338305 | 0.001619416 |
| 235363_at | --- | --- | BF108778 |  | -1.992784091 | 0.007456419 |
| 235534_at | --- | --- | AI624156 |  | 2.120117499 | 0.002012101 |
| 236619_at | --- | --- | AI922972 |  | -2.441188943 | 0.006000811 |
| 238178_at | --- | --- | BF110268 |  | -1.853184581 | 0.012383753 |
| 239049_at | --- | --- | BF514509 |  | -2.26739434 | 0.019099896 |
| 239735_at | --- | --- | N67106 |  | -2.220228124 | 0.018796563 |
| 241156_at | --- | --- | AI939588 |  | -1.9892425 | 0.034230766 |
| 242346_x_at | --- | --- | BF222929 |  | -1.904439824 | 0.023373929 |
| 242787_at | --- | --- | AI924134 |  | -2.524700822 | 0.004903065 |
| 228490_at | ABHD2 | abhydrolase domain containing 2 | AW292816 | 11057 | 1.651086296 | 0.008275448 |
| 218739_at | ABHD5 | abhydrolase domain containing 5 | NM_016006 | 51099 | 1.734291624 | 0.019029063 |
| 206068_s_at | ACADL | acyl-CoA dehydrogenase, long chain | AI367275 | 33 | 2.079944332 | 0.011353632 |
| 219616_at | ACSS3 | acyl-CoA synthetase short-chain family member 3 | NM_024560 | 79611 | 1.663831266 | 0.04301494 |
| 200974_at | ACTA2 | actin, alpha 2, smooth muscle, aorta | NM_001613 | 59 | 3.548696585 | 0.020757927 |
| 208637_x_at | ACTN1 | actinin, alpha 1 | BC003576 | 87 | 1.720874946 | 0.019979211 |
| 228416_at | ACVR2A | activin A receptor, type IIA | AI149508 | 92 | -1.862823511 | 0.000535912 |
| 206046_at | ADAM23 | ADAM metallopeptidase domain 23 | NM_003812 | 8745 | 2.365563224 | 0.005121259 |
| 235441_at | ADCY3 | adenylate cyclase 3 | BF217471 | 109 | -1.653382535 | 0.001253839 |
| 219361_s_at | AEN | apoptosis enhancing nuclease | NM_022767 | 64782 | 1.658592898 | 0.013702824 |
| 238889_at | AGBL5 | ATP/GTP binding protein-like 5 | AA045527 | 60509 | 1.641918196 | 0.002257386 |
| 222126_at | AGFG2 | ArfGAP with FG repeats 2 | AI247494 | 3268 | 1.634482285 | 0.040705351 |
| 1555736_a_at | AGTRAP | angiotensin II receptor-associated protein | AF165187 | 57085 | 1.825420374 | 0.01589332 |
| 235610_at | ALKBH8 | alkB, alkylation repair homolog 8 (E. coli) | AI590659 | 91801 | 1.639212257 | 0.036256707 |
| 228255_at | ALS2CR4 | amyotrophic lateral sclerosis 2 (juvenile) chromosome region, candidate 4 | AU150140 | 65062 | -1.660768045 | 0.004219583 |
| 219834_at | ALS2CR8 | amyotrophic lateral sclerosis 2 (juvenile) chromosome region, candidate 8 | NM_024744 | 79800 | 1.754454665 | 0.014599454 |
| 219082_at | AMDHD2 | amidohydrolase domain containing 2 | NM_015944 | 51005 | 1.738606949 | 0.048479447 |
| 217630_at | ANGEL2 | angel homolog 2 (Drosophila) | AI188346 | 90806 | 1.638636992 | 0.000292815 |
| 221826_at | ANGEL2 | angel homolog 2 (Drosophila) | BE671941 | 90806 | 1.655750523 | 0.018064379 |
| 226663_at | ANKRD10 | ankyrin repeat domain 10 | BE670056 | 55608 | -1.822124182 | 0.003212926 |
| 236421_at | ANKRD45 | ankyrin repeat domain 45 | AI204272 | 339416 | 1.971518223 | 0.018059281 |
| 201012_at | ANXA1 | annexin A1 | NM_000700 | 301 | 2.663313931 | 0.036123284 |
| 201590_x_at | ANXA2 | annexin A2 | NM_004039 | 302 | 2.985113774 | 0.030106363 |
| 210427_x_at | ANXA2 | annexin A2 | BC001388 | 302 | 2.832924527 | 0.032533388 |
| 213503_x_at | ANXA2 | annexin A2 | BE908217 | 302 | 2.803459521 | 0.031710523 |
| 201301_s_at | ANXA4 | annexin A4 | BC000182 | 307 | 3.036660663 | 0.00059073 |
| 201302_at | ANXA4 | annexin A4 | NM_001153 | 307 | 2.316535089 | 0.000192891 |
| 1555731_a_at | AP1S3 | adaptor-related protein complex 1, sigma 3 subunit | AF393369 | 130340 | 2.865526432 | 0.000158545 |
| 1555734_x_at | AP1S3 | adaptor-related protein complex 1, sigma 3 subunit | AF393369 | 130340 | 1.652098451 | 0.02219421 |
| 221036_s_at | APH1B | anterior pharynx defective 1 homolog B (C. elegans) | NM_031301 | 83464 | 1.716914691 | 0.035717436 |
| 214102_at | ARAP2 | ArfGAP with RhoGAP domain, ankyrin repeat and PH domain 2 | AK023737 | 116984 | -1.733580434 | 0.008415843 |
| 215931_s_at | ARFGEF2 | ADP-ribosylation factor guanine nucleotide-exchange factor 2 (brefeldin A-inhibited) | AV657604 | 10564 | 1.699067647 | 0.009894946 |
| 203910_at | ARHGAP29 | Rho GTPase activating protein 29 | NM_004815 | 9411 | 3.210689843 | 0.014113689 |
| 230047_at | ARHGAP42 | Rho GTPase activating protein 42 | BF439533 | 143872 | 1.928562607 | 0.003973778 |
| 235595_at | ARHGEF2 | Rho/Rac guanine nucleotide exchange factor (GEF) 2 | AW299534 | 9181 | -1.752886849 | 0.019667737 |
| 240008_at | ARID1B | AT rich interactive domain 1B (SWI1-like) | AI955765 | 57492 | -1.861111592 | 0.026069742 |
| 235959_at | ARID4B | AT rich interactive domain 4B (RBP1-like) | AI424238 | 51742 | -1.671222775 | 0.045727476 |
| 201659_s_at | ARL1 | ADP-ribosylation factor-like 1 | NM_001177 | 400 | 1.80367435 | 0.014221387 |
| 205020_s_at | ARL4A | ADP-ribosylation factor-like 4A | NM_005738 | 10124 | 1.835629149 | 0.016569566 |
| 222442_s_at | ARL8B | ADP-ribosylation factor-like 8B | AW055237 | 55207 | 1.730386269 | 0.016662847 |
| 212815_at | ASCC3 | activating signal cointegrator 1 complex subunit 3 | AA156961 | 10973 | 2.234719718 | 6.61533E-06 |
| 231269_at | ASCC3 | activating signal cointegrator 1 complex subunit 3 | AU153330 | 10973 | 1.951901485 | 0.000296913 |
| 232238_at | ASPM | asp (abnormal spindle) homolog, microcephaly associated (Drosophila) | AK001380 | 259266 | -3.990791683 | 0.0003971 |
| 228401_at | ATAD2 | ATPase family, AAA domain containing 2 | AI656807 | 29028 | -2.635421807 | 0.002103828 |
| 202672_s_at | ATF3 | activating transcription factor 3 | NM_001674 | 467 | 6.586445294 | 0.032089109 |
| 239825_at | ATF6 | activating transcription factor 6 | R17746 | 22926 | 1.788272519 | 0.022252296 |
| 223452_s_at | ATL3 | atlastin GTPase 3 | AL117600 | 25923 | 1.884636917 | 0.010720693 |
| 202872_at | ATP6V1C1 | ATPase, H+ transporting, lysosomal 42kDa, V1 subunit C1 | AW024925 | 528 | 1.854404111 | 0.000839901 |
| 202874_s_at | ATP6V1C1 | ATPase, H+ transporting, lysosomal 42kDa, V1 subunit C1 | NM_001695 | 528 | 1.724945685 | 0.011743783 |
| 238662_at | ATPBD4 | ATP binding domain 4 | BE884544 | 89978 | 1.697265747 | 0.015508478 |
| 208079_s_at | AURKA | aurora kinase A | NM_003158 | 6790 | -2.946243928 | 0.018121232 |
| 229715_at | B7H6 | --- | AW006182 | 374383 | 1.966011889 | 0.028577802 |
| 210534_s_at | B9D1 | B9 protein domain 1 | BC002944 | 27077 | 1.720225522 | 0.018346 |
| 219624_at | BAG4 | BCL2-associated athanogene 4 | NM_004874 | 9530 | 1.933385012 | 0.012956835 |
| 204966_at | BAI2 | brain-specific angiogenesis inhibitor 2 | NM_001703 | 576 | 1.69571801 | 0.023274455 |
| 209502_s_at | BAIAP2 | BAI1-associated protein 2 | BC002495 | 10458 | 2.140686288 | 0.002801744 |
| 233302_at | BCL11B | B-cell CLL/lymphoma 11B (zinc finger protein) | AU146285 | 64919 | -2.202856608 | 0.036159213 |
| 224520_s_at | BEST3 | bestrophin 3 | BC006440 | 144453 | -2.114526088 | 0.029276479 |
| 202094_at | BIRC5 | baculoviral IAP repeat-containing 5 | AA648913 | 332 | -5.037472094 | 0.017437084 |
| 202095_s_at | BIRC5 | baculoviral IAP repeat-containing 5 | NM_001168 | 332 | -10.00064477 | 0.00110076 |
| 210334_x_at | BIRC5 | baculoviral IAP repeat-containing 5 | AB028869 | 332 | -4.117415784 | 0.002701207 |
| 235609_at | BRIP1 | BRCA1 interacting protein C-terminal helicase 1 | BF056791 | 83990 | -4.711729811 | 0.000706911 |
| 224484_s_at | BRMS1L | breast cancer metastasis-suppressor 1-like | BC006250 | 84312 | 2.171498492 | 0.031970115 |
| 209642_at | BUB1 | budding uninhibited by benzimidazoles 1 homolog (yeast) | AF043294 | 699 | -3.247045772 | 0.041110769 |
| 1559265_at | C10orf140 | chromosome 10 open reading frame 140 | AI363206 | 387640 | -1.719518699 | 0.006836 |
| 1559266_s_at | C10orf140 | chromosome 10 open reading frame 140 | AI363206 | 387640 | -1.87998529 | 0.000387993 |
| 229801_at | C10orf47 | chromosome 10 open reading frame 47 | AI640157 | 254427 | 1.711373729 | 0.00038418 |
| 220987_s_at | C11orf17 /// NUAK2 | chromosome 11 open reading frame 17 /// NUAK family, SNF1-like kinase, 2 | NM_030952 | 56672 /// 81788 | 2.002540784 | 0.009005499 |
| 1552988_at | C11orf65 | chromosome 11 open reading frame 65 | NM_152587 | 160140 | 1.811855253 | 0.022206301 |
| 228378_at | C12orf29 | chromosome 12 open reading frame 29 | BF439204 | 91298 | 1.657995431 | 0.018558764 |
| 218374_s_at | C12orf4 | chromosome 12 open reading frame 4 | NM_020374 | 57102 | 1.982628998 | 0.007058522 |
| 226349_at | C12orf45 | chromosome 12 open reading frame 45 | BE264828 | 121053 | 1.947774907 | 0.004432878 |
| 222767_s_at | C12orf49 | chromosome 12 open reading frame 49 | AW872378 | 79794 | 1.928226173 | 0.010739573 |
| 235026_at | C12orf66 | chromosome 12 open reading frame 66 | AI885871 | 144577 | 2.011393346 | 0.011183588 |
| 219471_at | C13orf18 | chromosome 13 open reading frame 18 | NM_025113 | 80183 | 1.875107391 | 0.033819585 |
| 44790_s_at | C13orf18 | chromosome 13 open reading frame 18 | AI129310 | 80183 | 2.185612253 | 0.023525626 |
| 238647_at | C14orf28 | chromosome 14 open reading frame 28 | AA496213 | 122525 | 1.716256158 | 0.042653308 |
| 232094_at | C15orf29 | chromosome 15 open reading frame 29 | AU144048 | 79768 | -1.808386099 | 0.021406154 |
| 226456_at | C16orf75 | chromosome 16 open reading frame 75 | AW138157 | 116028 | -1.756261979 | 0.035542534 |
| 225967_s_at | C17orf89 | chromosome 17 open reading frame 89 | BF683512 | 284184 | 1.667929152 | 0.002839222 |
| 217508_s_at | C18orf25 | chromosome 18 open reading frame 25 | BE783279 | 147339 | 1.942827907 | 0.005877679 |
| 229442_at | C18orf54 | chromosome 18 open reading frame 54 | BF059556 | 162681 | -1.94929572 | 0.008693495 |
| 241733_at | C18orf54 | Chromosome 18 open reading frame 54 | AA019836 | 162681 | -2.133052632 | 0.042753721 |
| 244324_at | C18orf54 | chromosome 18 open reading frame 54 | BG283921 | 162681 | -1.848978678 | 0.017244793 |
| 228989_at | C18orf56 | chromosome 18 open reading frame 56 | AW291159 | 494514 | 2.763559005 | 3.69229E-05 |
| 229086_at | C1orf213 | chromosome 1 open reading frame 213 | AW298597 | 148898 | 2.658934832 | 0.000901315 |
| 1555225_at | C1orf43 | chromosome 1 open reading frame 43 | BC008306 | 25912 | 1.688192677 | 0.006434231 |
| 1553338_at | C1orf55 | chromosome 1 open reading frame 55 | NM_152608 | 163859 | 2.388856441 | 0.003435134 |
| 226813_at | C1orf57 | chromosome 1 open reading frame 57 | AA883980 | 84284 | 1.78594185 | 0.047433928 |
| 224443_at | C1orf97 | chromosome 1 open reading frame 97 | BC005997 | 84791 | 1.880282394 | 0.001920532 |
| 224444_s_at | C1orf97 | chromosome 1 open reading frame 97 | BC005997 | 84791 | 2.109818195 | 0.008579993 |
| 220543_at | C21orf62 | chromosome 21 open reading frame 62 | NM_019596 | 56245 | 2.500671648 | 0.028327585 |
| 227188_at | C21orf63 | chromosome 21 open reading frame 63 | AI744591 | 59271 | 2.831922247 | 0.019541539 |
| 220941_s_at | C21orf91 | chromosome 21 open reading frame 91 | NM_017447 | 54149 | 2.076203405 | 0.013680103 |
| 219662_at | C2orf49 | chromosome 2 open reading frame 49 | NM_024093 | 79074 | 1.852183 | 0.003396822 |
| 235181_at | C2orf60 | chromosome 2 open reading frame 60 | H12075 | 129450 | 1.989653042 | 0.011897927 |
| 228859_at | C4orf21 | chromosome 4 open reading frame 21 | BF056790 | 91431 | -1.916172754 | 0.033543326 |
| 224707_at | C5orf32 | chromosome 5 open reading frame 32 | AL522667 | 84418 | 1.763955109 | 0.018175356 |
| 223276_at | C5orf62 | chromosome 5 open reading frame 62 | AF313413 | 85027 | 2.329800621 | 0.004235361 |
| 218008_at | C7orf42 | chromosome 7 open reading frame 42 | NM_017994 | 55069 | 1.636272357 | 0.017164305 |
| 227534_at | C9orf21 | chromosome 9 open reading frame 21 | AI655189 | 195827 | 1.9486205 | 0.011590112 |
| 219928_s_at | CABYR | calcium binding tyrosine-(Y)-phosphorylation regulated | NM_012189 | 26256 | 2.298325529 | 0.002065364 |
| 224279_s_at | CABYR | calcium binding tyrosine-(Y)-phosphorylation regulated | AF295039 | 26256 | 1.650588612 | 0.009989609 |
| 225019_at | CAMK2D | calcium/calmodulin-dependent protein kinase II delta | BF797381 | 817 | 1.631508264 | 0.008259605 |
| 1552703_s_at | CARD16 /// CASP1 | caspase recruitment domain family, member 16 /// caspase 1, apoptosis-related cysteine peptidase (interleukin 1, beta, convertase) | NM_052889 | 114769 /// 834 | 1.850838304 | 0.027107241 |
| 202402_s_at | CARS | cysteinyl-tRNA synthetase | NM_001751 | 833 | 1.634922972 | 0.013840045 |
| 228323_at | CASC5 | cancer susceptibility candidate 5 | BF248364 | 57082 | -4.576729153 | 0.00098925 |
| 207467_x_at | CAST | calpastatin | NM_001750 | 831 | 1.981126663 | 0.00017277 |
| 208908_s_at | CAST | calpastatin | AF327443 | 831 | 1.7851842 | 0.002179058 |
| 206788_s_at | CBFB | core-binding factor, beta subunit | AF294326 | 865 | 1.794029191 | 0.010273115 |
| 226085_at | CBX5 | chromobox homolog 5 (HP1 alpha homolog, Drosophila) | AA181060 | 23468 | -2.055111064 | 0.017556134 |
| 1552326_a_at | CCDC11 | coiled-coil domain containing 11 | NM_145020 | 220136 | 1.848510412 | 0.032070112 |
| 203119_at | CCDC86 | coiled-coil domain containing 86 | NM_024098 | 79080 | 1.6283883 | 0.024005758 |
| 215343_at | CCDC88C | coiled-coil domain containing 88C | AF070587 | 440193 | 1.687834827 | 0.018214517 |
| 220094_s_at | CCDC90A | coiled-coil domain containing 90A | NM_022102 | 63933 | 1.626333702 | 0.023323635 |
| 213226_at | CCNA2 | cyclin A2 | AI346350 | 890 | -2.450813774 | 0.045137038 |
| 214710_s_at | CCNB1 | cyclin B1 | BE407516 | 891 | -3.522457523 | 0.025191181 |
| 228729_at | CCNB1 | cyclin B1 | N90191 | 891 | -3.640046665 | 0.017757555 |
| 202705_at | CCNB2 | cyclin B2 | NM_004701 | 9133 | -5.18092292 | 0.000758237 |
| 208711_s_at | CCND1 | cyclin D1 | BC000076 | 595 | 2.713339094 | 0.04501004 |
| 205034_at | CCNE2 | cyclin E2 | NM_004702 | 9134 | -2.830810044 | 0.033457542 |
| 236974_at | CCNI | cyclin I | AA808018 | 10983 | -1.684291355 | 0.014206376 |
| 208653_s_at | CD164 | CD164 molecule, sialomucin | AF263279 | 8763 | 2.883378639 | 0.008222821 |
| 204490_s_at | CD44 | CD44 molecule (Indian blood group) | M24915 | 960 | 2.78829184 | 0.00108549 |
| 209835_x_at | CD44 | CD44 molecule (Indian blood group) | BC004372 | 960 | 2.558092761 | 0.000218518 |
| 212014_x_at | CD44 | CD44 molecule (Indian blood group) | AI493245 | 960 | 2.544771399 | 0.000656271 |
| 216942_s_at | CD58 | CD58 molecule | D28586 | 965 | 1.639107464 | 0.007351357 |
| 230887_at | CDC14B | CDC14 cell division cycle 14 homolog B (S. cerevisiae) | AI921238 | 168448 /// 8555 | 2.020916568 | 0.001647636 |
| 204695_at | CDC25A | cell division cycle 25 homolog A (S. pombe) | AI343459 | 993 | -1.848605518 | 0.007740106 |
| 205167_s_at | CDC25C | cell division cycle 25 homolog C (S. pombe) | NM_001790 | 995 | -2.328070566 | 0.019203184 |
| 223307_at | CDCA3 | cell division cycle associated 3 | BC002551 | 83461 | -3.062087166 | 0.024094918 |
| 224753_at | CDCA5 | cell division cycle associated 5 | BE614410 | 113130 | -2.770497652 | 0.001936849 |
| 203213_at | CDK1 | cyclin-dependent kinase 1 | AL524035 | 983 | -5.052204206 | 0.002075691 |
| 203214_x_at | CDK1 | cyclin-dependent kinase 1 | NM_001786 | 983 | -4.320829061 | 0.003220406 |
| 210559_s_at | CDK1 | cyclin-dependent kinase 1 | D88357 | 983 | -5.462988718 | 0.001612966 |
| 202284_s_at | CDKN1A | cyclin-dependent kinase inhibitor 1A (p21, Cip1) | NM_000389 | 1026 | 3.292238137 | 3.18173E-05 |
| 204159_at | CDKN2C | cyclin-dependent kinase inhibitor 2C (p18, inhibits CDK4) | NM_001262 | 1031 | -2.652285621 | 0.009693365 |
| 211792_s_at | CDKN2C | cyclin-dependent kinase inhibitor 2C (p18, inhibits CDK4) | U17074 | 1031 | -1.726908089 | 0.003218943 |
| 212862_at | CDS2 | CDP-diacylglycerol synthase (phosphatidate cytidylyltransferase) 2 | AL568982 | 8760 | 1.87796778 | 0.00260672 |
| 228868_x_at | CDT1 | Chromatin licensing and DNA replication factor 1 | AW075105 | 81620 | -2.707749308 | 0.032838323 |
| 212501_at | CEBPB | CCAAT/enhancer binding protein (C/EBP), beta | AL564683 | 1051 | 2.887696349 | 0.005813558 |
| 204203_at | CEBPG | CCAAT/enhancer binding protein (C/EBP), gamma | NM_001806 | 1054 | 2.098006936 | 0.025738151 |
| 1554569_a_at | CELF2 | CUGBP, Elav-like family member 2 | BC036391 | 10659 | 1.839065564 | 0.009482992 |
| 204962_s_at | CENPA | centromere protein A | NM_001809 | 1058 | -4.293702108 | 0.002717095 |
| 207828_s_at | CENPF | centromere protein F, 350/400ka (mitosin) | NM_005196 | 1063 | -3.799159267 | 0.000784237 |
| 209172_s_at | CENPF | centromere protein F, 350/400ka (mitosin) | U30872 | 1063 | -4.892057653 | 0.003621244 |
| 231772_x_at | CENPH | centromere protein H | AL572471 | 64946 | -1.895220175 | 0.047919286 |
| 223513_at | CENPJ | centromere protein J | AF139625 | 55835 | -1.765342505 | 0.01978227 |
| 222848_at | CENPK | centromere protein K | BC005400 | 64105 | -3.48928621 | 0.044301758 |
| 219555_s_at | CENPN | centromere protein N | NM_018455 | 55839 | -3.007632275 | 0.002421135 |
| 228559_at | CENPN | centromere protein N | BF111626 | 55839 | -2.519870248 | 0.034485153 |
| 226118_at | CENPO | centromere protein O | BE326728 | 79172 | -1.63190098 | 0.027301569 |
| 226936_at | CENPW | centromere protein W | BG492359 | 387103 | -1.731780575 | 0.044044343 |
| 209667_at | CES2 | carboxylesterase 2 (intestine, liver) | BF033242 | 8824 | 1.723876012 | 0.002160761 |
| 209668_x_at | CES2 | carboxylesterase 2 (intestine, liver) | D50579 | 8824 | 1.835295569 | 0.000529519 |
| 210701_at | CFDP1 | craniofacial development protein 1 | D85939 | 10428 | -1.75060209 | 0.019051329 |
| 206861_s_at | CGGBP1 | CGG triplet repeat binding protein 1 | NM_003663 | 8545 | 1.68712992 | 0.010302331 |
| 238075_at | CHEK1 | checkpoint kinase 1 | AA224205 | 1111 | -2.231793538 | 0.039399801 |
| 205393_s_at | CHEK1 | CHK1 checkpoint homolog (S. pombe) | NM_001274 | 1111 | -2.043269753 | 0.026301256 |
| 1569183_a_at | CHM | choroideremia (Rab escort protein 1) | BC032237 | 1121 | 2.024278377 | 0.028884925 |
| 210123_s_at | CHRFAM7A /// CHRNA7 | CHRNA7 (cholinergic receptor, nicotinic, alpha 7, exons 5-10) and FAM7A (family with sequence similarity 7A, exons A-E) fusion /// cholinergic receptor, nicotinic, alpha 7 | U62436 | 1139 /// 652740 /// 89832 | 1.653870864 | 0.009642114 |
| 209834_at | CHST3 | carbohydrate (chondroitin 6) sulfotransferase 3 | AB017915 | 9469 | 1.747873931 | 0.026504355 |
| 32094_at | CHST3 | carbohydrate (chondroitin 6) sulfotransferase 3 | AB017915 | 9469 | 1.679715597 | 0.010909833 |
| 244275_at | CISD2 | CDGSH iron sulfur domain 2 | AI732988 | 150159 | -1.641882936 | 0.020448827 |
| 201897_s_at | CKS1B | CDC28 protein kinase regulatory subunit 1B | NM_001826 | 1163 | -1.856366787 | 0.033015718 |
| 204170_s_at | CKS2 | CDC28 protein kinase regulatory subunit 2 | NM_001827 | 1164 | -2.346167366 | 0.014040034 |
| 241403_at | CLK4 | CDC-like kinase 4 | AA468591 | 57396 | -1.795742747 | 0.029846227 |
| 227522_at | CMBL | carboxymethylenebutenolidase homolog (Pseudomonas) | AA209487 | 134147 | 2.081397041 | 8.83068E-05 |
| 1554522_at | CNNM2 | cyclin M2 | BC021222 | 54805 | 2.075378776 | 0.000508112 |
| 223796_at | CNTNAP3 | contactin associated protein-like 3 | AF333769 | 389734 /// 642342 /// 642373 /// 643792 /// 79937 | 2.234777761 | 0.001270018 |
| 244065_at | CNTNAP3B | contactin associated protein-like 3B | AW016751 | 389734 /// 642373 /// 643792 | 1.917564004 | 0.00640248 |
| 203642_s_at | COBLL1 | COBL-like 1 | NM_014900 | 22837 | 1.984431552 | 0.00361527 |
| 211981_at | COL4A1 | collagen, type IV, alpha 1 | NM_001845 | 1282 | 2.904224421 | 0.032783043 |
| 238733_at | CPM | carboxypeptidase M | AI422414 | 1368 | 2.342386298 | 0.006202282 |
| 202469_s_at | CPSF6 | cleavage and polyadenylation specific factor 6, 68kDa | AU149367 | 11052 | 1.725928233 | 0.025682132 |
| 228177_at | CREBBP | CREB binding protein | AA742293 | 1387 | -1.706479686 | 0.023548351 |
| 1554464_a_at | CRTAP | cartilage associated protein | BC008745 | 10491 | 2.006982856 | 0.024989096 |
| 209283_at | CRYAB | crystallin, alpha B | AF007162 | 1410 | 2.211831225 | 0.01382451 |
| 201160_s_at | CSDA | cold shock domain protein A | AL556190 | 8531 | 2.634061089 | 6.07041E-05 |
| 201161_s_at | CSDA | cold shock domain protein A | NM_003651 | 8531 | 2.592161485 | 2.17081E-05 |
| 1557143_at | CSMD2 | CUB and Sushi multiple domains 2 | AB067471 | 114784 | -1.632436351 | 0.046807895 |
| 218924_s_at | CTBS | chitobiase, di-N-acetyl- | NM_004388 | 1486 | 1.700975778 | 0.009364875 |
| 823_at | CX3CL1 | chemokine (C-X3-C motif) ligand 1 | U84487 | 6376 | 1.992379936 | 0.004293903 |
| 217889_s_at | CYBRD1 | cytochrome b reductase 1 | NM_024843 | 79901 | 1.719544679 | 0.001496006 |
| 207386_at | CYP7B1 | cytochrome P450, family 7, subfamily B, polypeptide 1 | NM_004820 | 9420 | 1.928870627 | 0.007995866 |
| 244803_at | DAP3 | death associated protein 3 | AI335191 | 7818 | -2.239129865 | 0.023449564 |
| 220172_at | DCAF17 | DDB1 and CUL4 associated factor 17 | NM_025000 | 80067 | 2.103682212 | 0.013681354 |
| 209231_s_at | DCTN5 | dynactin 5 (p25) | AI038068 | 84516 | 1.819422829 | 0.003833431 |
| 222678_s_at | DCUN1D1 | DCN1, defective in cullin neddylation 1, domain containing 1 (S. cerevisiae) | BF057821 | 54165 | 1.826609339 | 0.042555604 |
| 203409_at | DDB2 | damage-specific DNA binding protein 2, 48kDa | NM_000107 | 1643 | 1.680115159 | 0.023720172 |
| 209383_at | DDIT3 | DNA-damage-inducible transcript 3 | BC003637 | 1649 | 2.309706263 | 0.019029946 |
| 226980_at | DEPDC1B | DEP domain containing 1B | AK001166 | 55789 | -3.266486082 | 0.008674326 |
| 205603_s_at | DIAPH2 | diaphanous homolog 2 (Drosophila) | NM_007309 | 1730 | 1.656668791 | 0.007854156 |
| 231412_at | DKFZp686L14188 | hypothetical gene supported by BX538329 | H04388 | 441366 | 1.660642446 | 0.00780864 |
| 1556821_x_at | DLEU2 | deleted in lymphocytic leukemia 2 (non-protein coding) | H48516 | 8847 | -2.846342602 | 0.036244589 |
| 213647_at | DNA2 | DNA replication helicase 2 homolog (yeast) | D42046 | 1763 | -2.009196582 | 0.000553086 |
| 242283_at | DNAH14 | dynein, axonemal, heavy chain 14 | AI076810 | 200095 | 2.915612964 | 0.004104593 |
| 221782_at | DNAJC10 | DnaJ (Hsp40) homolog, subfamily C, member 10 | BG168666 | 54431 | 1.73260573 | 0.029111648 |
| 225195_at | DPH3 | DPH3, KTI11 homolog (S. cerevisiae) | AI825858 | 285381 | 1.631028753 | 0.021764798 |
| 1560916_a_at | DPY19L1 | dpy-19-like 1 (C. elegans) | BI461155 | 23333 | 1.867951344 | 0.005557654 |
| 207654_x_at | DR1 | down-regulator of transcription 1, TBP-binding (negative cofactor 2) | NM_001938 | 1810 | 1.733395516 | 0.020671818 |
| 209188_x_at | DR1 | down-regulator of transcription 1, TBP-binding (negative cofactor 2) | BC002809 | 1810 | 1.76830671 | 0.012560006 |
| 216652_s_at | DR1 | down-regulator of transcription 1, TBP-binding (negative cofactor 2) | AL137673 | 1810 | 1.643217266 | 0.033119332 |
| 218627_at | DRAM1 | DNA-damage regulated autophagy modulator 1 | NM_018370 | 55332 | 2.219177609 | 0.001317775 |
| 218585_s_at | DTL | denticleless homolog (Drosophila) | NM_016448 | 51514 | -4.572646638 | 0.001357479 |
| 222680_s_at | DTL | denticleless homolog (Drosophila) | AK001261 | 51514 | -2.997399314 | 0.005837639 |
| 205741_s_at | DTNA | dystrobrevin, alpha | NM_001392 | 1837 | 2.009394545 | 0.038771157 |
| 225415_at | DTX3L | deltex 3-like (Drosophila) | AA577672 | 151636 | 1.75634195 | 0.004478341 |
| 203367_at | DUSP14 | dual specificity phosphatase 14 | NM_007026 | 11072 | 1.732444721 | 0.023921611 |
| 201537_s_at | DUSP3 | dual specificity phosphatase 3 | BC002682 | 1845 | 2.321935612 | 0.000416103 |
| 201538_s_at | DUSP3 | dual specificity phosphatase 3 | NM_004090 | 1845 | 2.763315806 | 0.001692571 |
| 1554324_s_at | DYNC2LI1 | dynein, cytoplasmic 2, light intermediate chain 1 | BC040558 | 51626 | 1.647457182 | 0.004499245 |
| 227437_at | DYNLL1 | dynein, light chain, LC8-type 1 | BE044272 | 8655 | 1.65662288 | 0.008590922 |
| 219787_s_at | ECT2 | epithelial cell transforming sequence 2 oncogene | NM_018098 | 1894 | -2.344706765 | 0.041253116 |
| 225275_at | EDIL3 | EGF-like repeats and discoidin I-like domains 3 | AA053711 | 10085 | 2.345013735 | 0.028597289 |
| 219232_s_at | EGLN3 | egl nine homolog 3 (C. elegans) | NM_022073 | 112399 | -1.920335883 | 0.023370874 |
| 201693_s_at | EGR1 | early growth response 1 | AV733950 | 1958 | 4.713254023 | 0.034454342 |
| 205249_at | EGR2 | early growth response 2 | NM_000399 | 1959 | 4.866964025 | 0.049827458 |
| 45297_at | EHD2 | EH-domain containing 2 | AI417917 | 30846 | 1.742286787 | 0.008292561 |
| 209536_s_at | EHD4 | EH-domain containing 4 | AF320070 | 30844 | 1.751119213 | 0.014663518 |
| 220198_s_at | EIF5A2 | eukaryotic translation initiation factor 5A2 | NM_020390 | 56648 | 1.740001764 | 0.002693472 |
| 226099_at | ELL2 | elongation factor, RNA polymerase II, 2 | AI924426 | 22936 | 2.449976118 | 0.048764072 |
| 231930_at | ELMOD1 | ELMO/CED-12 domain containing 1 | AL359601 | 55531 | 2.749761079 | 0.00377469 |
| 1553928_at | ELMOD2 | ELMO/CED-12 domain containing 2 | NM_153702 | 255520 | 1.902074047 | 0.006065928 |
| 234464_s_at | EME1 | essential meiotic endonuclease 1 homolog 1 (S. pombe) | AK021607 | 146956 | -1.65058963 | 0.012337121 |
| 201324_at | EMP1 | epithelial membrane protein 1 | NM_001423 | 2012 | 8.308470307 | 0.027962998 |
| 203729_at | EMP3 | epithelial membrane protein 3 | NM_001425 | 2014 | 1.674044208 | 0.019541489 |
| 210839_s_at | ENPP2 | ectonucleotide pyrophosphatase/phosphodiesterase 2 | D45421 | 5168 | 2.10034068 | 0.027880618 |
| 226776_at | ENY2 | enhancer of yellow 2 homolog (Drosophila) | BF433516 | 56943 | -1.656388087 | 0.037325747 |
| 225051_at | EPB41 | erythrocyte membrane protein band 4.1 (elliptocytosis 1, RH-linked) | AA522435 | 2035 | -1.693621918 | 0.020594938 |
| 203499_at | EPHA2 | EPH receptor A2 | NM_004431 | 1969 | 2.850065567 | 0.000218034 |
| 227449_at | EPHA4 | EPH receptor A4 | AI799018 | 2043 | -2.329651884 | 0.01691303 |
| 203643_at | ERF | Ets2 repressor factor | NM_006494 | 2077 | 1.783306725 | 0.021516042 |
| 1558511_s_at | ESYT2 | extended synaptotagmin-like protein 2 | AA187963 | 57488 | 1.652550991 | 0.02848681 |
| 224699_s_at | ESYT2 | extended synaptotagmin-like protein 2 | BE385892 | 57488 | 1.70339551 | 0.012554434 |
| 201573_s_at | ETF1 | eukaryotic translation termination factor 1 | M75715 | 2107 | 1.69659506 | 0.008576381 |
| 203348_s_at | ETV5 | ets variant 5 | BF060791 | 2119 | 3.895840319 | 0.032996741 |
| 203349_s_at | ETV5 | ets variant 5 | NM_004454 | 2119 | 3.766564603 | 0.046205582 |
| 1552314_a_at | EYA3 | eyes absent homolog 3 (Drosophila) | NM_172098 | 2140 | 1.624770864 | 0.023214618 |
| 203358_s_at | EZH2 | enhancer of zeste homolog 2 (Drosophila) | NM_004456 | 2146 | -1.788512738 | 0.00607189 |
| 202345_s_at | FABP5 | fatty acid binding protein 5 (psoriasis-associated) | NM_001444 | 2171 /// 653327 | 1.837474644 | 0.007499206 |
| 202862_at | FAH | fumarylacetoacetate hydrolase (fumarylacetoacetase) | NM_000137 | 2184 | 1.806985042 | 0.015375455 |
| 241991_at | FAM122C | family with sequence similarity 122C | AI629041 | 159091 | -1.672217835 | 0.031360981 |
| 230496_at | FAM123A | family with sequence similarity 123A | BE046923 | 219287 | -2.165805217 | 0.001011793 |
| 231117_at | FAM181A | family with sequence similarity 181, member A | BE962786 | 90050 | 1.671328233 | 0.021658829 |
| 209378_s_at | FAM190B | family with sequence similarity 190, member B | AI478879 | 54462 | 1.629993315 | 0.026652268 |
| 1553715_s_at | FAM195A | family with sequence similarity 195, member A | NM_032371 | 84331 | 1.782821942 | 0.010087765 |
| 225861_at | FAM195A | family with sequence similarity 195, member A | AW001250 | 84331 | 1.745832671 | 0.009118133 |
| 223204_at | FAM198B | family with sequence similarity 198, member B | AF260333 | 51313 | 1.799853716 | 0.047107487 |
| 223791_at | FAM27A /// FAM27B /// FAM27C | family with sequence similarity 27, member A /// family with sequence similarity 27, member B /// family with sequence similarity 27, member C | BC002886 | 548321 | 1.929362765 | 0.002384205 |
| 232899_at | FAM41C /// RPL23AP7 /// RPL23AP82 | family with sequence similarity 41, member C /// ribosomal protein L23a pseudogene 7 /// ribosomal protein L23a pseudogene 82 | R17536 | 118433 /// 284593 /// 284942 /// 642257 /// 642720 /// 644033 /// 644113 /// 644964 /// 645236 /// 650103 /// 653138 /// 653346 | 2.040349102 | 0.004585321 |
| 227410_at | FAM43A | family with sequence similarity 43, member A | AW264102 | 131583 | 2.600365665 | 0.000815797 |
| 229518_at | FAM46B | family with sequence similarity 46, member B | AA531023 | 115572 | 1.824539123 | 0.021006361 |
| 221591_s_at | FAM64A | family with sequence similarity 64, member A | BC005004 | 54478 | -3.062667191 | 0.014992087 |
| 225687_at | FAM83D | family with sequence similarity 83, member D | BC001068 | 81610 | -3.784490245 | 0.001436437 |
| 65585_at | FAM86B1 | family with sequence similarity 86, member B1 | AA527515 | 55199 /// 648808 /// 653113 /// 653333 /// 653726 /// 692099 /// 85002 | 1.891005864 | 0.003597393 |
| 227951_s_at | FAM98C | family with sequence similarity 98, member C | AW338561 | 147965 | 1.737920749 | 0.002210035 |
| 223545_at | FANCD2 | Fanconi anemia, complementation group D2 | AF340183 | 2177 | -2.505206584 | 0.014440491 |
| 242560_at | FANCD2 | Fanconi anemia, complementation group D2 | AA579890 | 2177 | -5.925939157 | 0.001042439 |
| 213007_at | FANCI | Fanconi anemia, complementation group I | W74442 | 55215 | -2.255324365 | 0.015238039 |
| 213008_at | FANCI | Fanconi anemia, complementation group I | BG403615 | 55215 | -2.000283073 | 0.031773431 |
| 204780_s_at | FAS | Fas (TNF receptor superfamily, member 6) | AA164751 | 355 | 4.392612784 | 3.50584E-05 |
| 204781_s_at | FAS | Fas (TNF receptor superfamily, member 6) | NM_000043 | 355 | 3.765581087 | 5.67381E-06 |
| 215719_x_at | FAS | Fas (TNF receptor superfamily, member 6) | X83493 | 355 | 5.026032603 | 0.000988025 |
| 216252_x_at | FAS | Fas (TNF receptor superfamily, member 6) | Z70519 | 355 | 4.49339038 | 0.00088336 |
| 228427_at | FBXO16 | F-box protein 16 | BF196856 | 157574 | 1.669787578 | 0.002711808 |
| 225736_at | FBXO22 | F-box protein 22 | BE966247 | 26263 | 1.837030547 | 0.002766057 |
| 225737_s_at | FBXO22 | F-box protein 22 | BE966247 | 26263 | 1.905364474 | 0.007337151 |
| 234863_x_at | FBXO5 | F-box protein 5 | AK026197 | 26271 | -1.953836295 | 0.024391265 |
| 207813_s_at | FDXR | ferredoxin reductase | NM_004110 | 2232 | 3.482464813 | 4.02358E-05 |
| 226705_at | FGFR1 | fibroblast growth factor receptor 1 | BE467261 | 2260 | 1.65990476 | 0.036744597 |
| 1568678_s_at | FGFR1OP | FGFR1 oncogene partner | BC037785 | 11116 | 2.008318278 | 0.008518912 |
| 1556283_s_at | FGFR1OP2 | FGFR1 oncogene partner 2 | W74643 | 26127 | 1.65061664 | 0.023749173 |
| 206492_at | FHIT | fragile histidine triad gene | NM_002012 | 2272 | 1.641638615 | 0.033824612 |
| 205511_at | FLJ10038 | hypothetical protein FLJ10038 | NM_017976 | 55056 | -1.874910562 | 0.024344091 |
| 231313_at | FLJ27354 | hypothetical gene supported by AK130864 | AW134984 | 400761 | 1.815136579 | 0.027458201 |
| 1559964_at | FLJ38717 | FLJ38717 protein | AK096036 | 401261 | -1.755104645 | 0.027605928 |
| 228702_at | FLJ43663 | hypothetical LOC378805 | AL569506 | 378805 /// 641825 /// 647017 | 2.038037914 | 0.047894198 |
| 234949_at | FRG1B | FSHD region gene 1 family, member B | AL117574 | 284802 | -1.740773879 | 0.01637846 |
| 1558515_at | FTX | FTX transcript, XIST regulator (non-protein coding) | AK057701 | 100302692 | -1.677963055 | 0.034552073 |
| 205565_s_at | FXN | frataxin | NM_000144 | 2395 | 1.854259966 | 0.026846216 |
| 44654_at | G6PC3 | glucose 6 phosphatase, catalytic, 3 | AI669655 | 92579 | 1.638243916 | 0.028800093 |
| 203725_at | GADD45A | growth arrest and DNA-damage-inducible, alpha | NM_001924 | 1647 | 3.927506142 | 0.013850497 |
| 223991_s_at | GALNT2 /// LOC100132910 | UDP-N-acetyl-alpha-D-galactosamine:polypeptide N-acetylgalactosaminyltransferase 2 (GalNAc-T2) /// PRO1477 | AF130059 | 2590 | 1.981660007 | 0.016888952 |
| 1554079_at | GALNTL4 | UDP-N-acetyl-alpha-D-galactosamine:polypeptide N-acetylgalactosaminyltransferase-like 4 | BC037341 | 374378 | 1.755264098 | 0.006963519 |
| 1552474_a_at | GAMT | guanidinoacetate N-methyltransferase | NM_138924 | 2593 | 1.819857957 | 0.003295459 |
| 204471_at | GAP43 | growth associated protein 43 | NM_002045 | 2596 | 3.252461563 | 0.041109362 |
| 216963_s_at | GAP43 | growth associated protein 43 | AF279774 | 2596 | 2.94474144 | 0.014097443 |
| 204867_at | GCHFR | GTP cyclohydrolase I feedback regulator | NM_005258 | 2644 | 1.789777846 | 0.000788861 |
| 206397_x_at | GDF1 /// LASS1 | growth differentiation factor 1 /// LAG1 homolog, ceramide synthase 1 | NM_001492 | 10715 /// 2657 | 1.669105105 | 0.002891971 |
| 221577_x_at | GDF15 /// LOC100292463 | growth differentiation factor 15 /// similar to growth differentiation factor 15 | AF003934 | 9518 | 10.40510022 | 2.88153E-06 |
| 215380_s_at | GGCT | gamma-glutamylcyclotransferase | AK021779 | 79017 | 1.677740605 | 0.006889038 |
| 218719_s_at | GINS3 | GINS complex subunit 3 (Psf3 homolog) | NM_022770 | 64785 | -1.947394455 | 0.023207293 |
| 45633_at | GINS3 | GINS complex subunit 3 (Psf3 homolog) | AI421812 | 64785 | -2.274913007 | 0.010894627 |
| 225706_at | GLCCI1 | glucocorticoid induced transcript 1 | AI761989 | 113263 | -1.918072376 | 0.027361219 |
| 227525_at | GLCCI1 | glucocorticoid induced transcript 1 | AA058770 | 113263 | -2.06071026 | 0.010423665 |
| 219933_at | GLRX2 | glutaredoxin 2 | NM_016066 | 51022 | 1.669811325 | 0.032624109 |
| 241865_at | GNB1 | guanine nucleotide binding protein (G protein), beta polypeptide 1 | AI056689 | 2782 | -1.742576649 | 0.039337785 |
| 218193_s_at | GOLT1B | golgi transport 1 homolog B (S. cerevisiae) | NM_016072 | 51026 | 2.104642922 | 0.035639206 |
| 210009_s_at | GOSR2 | golgi SNAP receptor complex member 2 | AF229796 | 9570 | 1.895640968 | 0.001730643 |
| 219078_at | GPATCH2 | G patch domain containing 2 | NM_018040 | 55105 | 1.961892878 | 0.025175346 |
| 224632_at | GPATCH4 | G patch domain containing 4 | BE794289 | 54865 | 1.665761276 | 0.031589421 |
| 204984_at | GPC4 | glypican 4 | NM_001448 | 2239 | -1.683733413 | 0.005736586 |
| 223730_at | GPC6 | glypican 6 | AF111178 | 10082 | 1.625160623 | 0.008419875 |
| 230492_s_at | GPCPD1 | glycerophosphocholine phosphodiesterase GDE1 homolog (S. cerevisiae) | BE328402 | 56261 | 1.738718492 | 0.04793694 |
| 200736_s_at | GPX1 | glutathione peroxidase 1 | NM_000581 | 2876 | 1.986881452 | 0.000585872 |
| 209410_s_at | GRB10 | growth factor receptor-bound protein 10 | AF000017 | 2887 | 1.745670031 | 0.032842926 |
| 210999_s_at | GRB10 | growth factor receptor-bound protein 10 | U66065 | 2887 | 1.82604535 | 0.036463015 |
| 211520_s_at | GRIA1 | glutamate receptor, ionotropic, AMPA 1 | M64752 | 2890 | 1.867352776 | 0.018755767 |
| 206730_at | GRIA3 | glutamate receptor, ionotrophic, AMPA 3 | NM_007325 | 2892 | -1.67748922 | 0.004819719 |
| 229397_s_at | GRLF1 | Glucocorticoid receptor DNA binding factor 1 | AI275597 | 2909 | -1.640641729 | 0.007005607 |
| 225276_at | GSPT1 | G1 to S phase transition 1 | AA143579 | 2935 | -1.67318072 | 0.000696599 |
| 234975_at | GSPT1 | G1 to S phase transition 1 | BE544748 | 2935 | -1.769346841 | 0.038987913 |
| 218884_s_at | GUF1 | GUF1 GTPase homolog (S. cerevisiae) | NM_021927 | 60558 | 1.704553397 | 0.035424667 |
| 235123_at | GUSBP3 | glucuronidase, beta pseudogene 3 | AI951144 | 653188 /// 653391 | -1.665913107 | 0.018751574 |
| 215599_at | GUSBP3 | glucuronidase, beta pseudogene 3 | X83300 | 11039 /// 643367 /// 643373 /// 652924 /// 653869 | -1.979179401 | 0.044417427 |
| 235733_at | GXYLT2 | glucoside xylosyltransferase 2 | AW043921 | 151987 | 1.664932461 | 0.019480055 |
| 205436_s_at | H2AFX | H2A histone family, member X | NM_002105 | 3014 | -1.774333048 | 0.035682369 |
| 213344_s_at | H2AFX | H2A histone family, member X | H51429 | 3014 | -1.77639589 | 0.005484987 |
| 238275_at | HAP1 | huntingtin-associated protein 1 | AI809524 | 9001 | 2.50984954 | 0.004165109 |
| 223541_at | HAS3 | hyaluronan synthase 3 | AF232772 | 3038 | 2.079935557 | 0.004639697 |
| 242890_at | HELLS | helicase, lymphoid-specific | AI650364 | 3070 | -2.101648097 | 0.037799105 |
| 201944_at | HEXB | hexosaminidase B (beta polypeptide) | NM_000521 | 3074 | 2.073594434 | 0.002671051 |
| 220387_s_at | HHLA3 | HERV-H LTR-associating 3 | NM_007071 | 11147 | 2.145378111 | 0.044413204 |
| 215313_x_at | HLA-A | major histocompatibility complex, class I, A | AA573862 | 3105 | 1.661062216 | 0.041718309 |
| 216526_x_at | HLA-C | major histocompatibility complex, class I, C | AK024836 | 3107 | 2.11173913 | 0.039520288 |
| 224734_at | HMGB1 | high-mobility group box 1 | N92507 | 3146 | -1.85495499 | 0.009851484 |
| 232004_at | HNRNPR | heterogeneous nuclear ribonucleoprotein R | AK001846 | 10236 | -1.775835109 | 0.000795645 |
| 236723_at | HNRNPU | heterogeneous nuclear ribonucleoprotein U (scaffold attachment factor A) | AW196279 | 3192 | -1.981849375 | 0.000368866 |
| 235603_at | HNRNPU | heterogeneous nuclear ribonucleoprotein U (scaffold attachment factor A) | N95466 | 3192 | -1.880755387 | 0.024056206 |
| 210112_at | HPS1 | Hermansky-Pudlak syndrome 1 | U96721 | 3257 | 1.780880834 | 0.006221678 |
| 212983_at | HRAS | v-Ha-ras Harvey rat sarcoma viral oncogene homolog | NM_005343 | 3265 | 1.638360556 | 0.016147463 |
| 205543_at | HSPA4L | heat shock 70kDa protein 4-like | NM_014278 | 22824 | 3.030759768 | 0.002812359 |
| 201841_s_at | HSPB1 | heat shock 27kDa protein 1 | NM_001540 | 3315 | 1.937116161 | 0.023493264 |
| 219998_at | HSPC159 | galectin-related protein | NM_014181 | 29094 | 1.819390596 | 0.007334075 |
| 202069_s_at | IDH3A | isocitrate dehydrogenase 3 (NAD+) alpha | AI826060 | 3419 | 1.692918757 | 0.023521407 |
| 202439_s_at | IDS | iduronate 2-sulfatase | NM_000202 | 3423 | 1.784878438 | 0.004264385 |
| 206342_x_at | IDS | iduronate 2-sulfatase | NM_006123 | 3423 | 2.051611544 | 0.003653597 |
| 209417_s_at | IFI35 | interferon-induced protein 35 | BC001356 | 3430 | 2.174317184 | 0.016849912 |
| 204415_at | IFI6 | interferon, alpha-inducible protein 6 | NM_022873 | 2537 | 2.131520645 | 0.00936552 |
| 201601_x_at | IFITM1 | interferon induced transmembrane protein 1 (9-27) | NM_003641 | 8519 | 2.333175376 | 0.001531889 |
| 201315_x_at | IFITM2 | interferon induced transmembrane protein 2 (1-8D) | NM_006435 | 10581 | 2.546398613 | 0.018956396 |
| 212203_x_at | IFITM3 | interferon induced transmembrane protein 3 (1-8U) | BF338947 | 10410 | 2.872714812 | 0.012375783 |
| 243027_at | IGSF5 | immunoglobulin superfamily, member 5 | AI824021 | 150084 | 2.464016209 | 0.005465504 |
| 201887_at | IL13RA1 | interleukin 13 receptor, alpha 1 | NM_001560 | 3597 | 1.693045263 | 0.020615253 |
| 201888_s_at | IL13RA1 | interleukin 13 receptor, alpha 1 | U81379 | 3597 | 1.73003645 | 0.017656933 |
| 210904_s_at | IL13RA1 | interleukin 13 receptor, alpha 1 | U81380 | 3597 | 1.66492676 | 0.009578004 |
| 211612_s_at | IL13RA1 | interleukin 13 receptor, alpha 1 | U62858 | 3597 | 1.987640751 | 0.006056969 |
| 201626_at | INSIG1 | insulin induced gene 1 | BG292233 | 3638 | -1.794005504 | 0.044472562 |
| 204686_at | IRS1 | insulin receptor substrate 1 | NM_005544 | 3667 | 1.688396408 | 0.037275948 |
| 205483_s_at | ISG15 | ISG15 ubiquitin-like modifier | NM_005101 | 9636 | 2.35363231 | 0.048359346 |
| 209744_x_at | ITCH | itchy E3 ubiquitin protein ligase homolog (mouse) | AB056663 | 83737 | 1.746127724 | 0.009106162 |
| 214021_x_at | ITGB5 | Integrin, beta 5 | AI335208 | 3693 | 1.776837688 | 0.044431239 |
| 205816_at | ITGB8 | integrin, beta 8 | NM_002214 | 3696 | 2.072288893 | 0.015923581 |
| 227954_at | ITPRIPL2 | inositol 1,4,5-triphosphate receptor interacting protein-like 2 | AI458417 | 162073 | 1.694127912 | 0.005864365 |
| 205889_s_at | JAKMIP2 | janus kinase and microtubule interacting protein 2 | NM_014790 | 9832 | -1.787580318 | 0.004276968 |
| 1553764_a_at | JUB | jub, ajuba homolog (Xenopus laevis) | NM_032876 | 84962 | 1.745154929 | 0.005972036 |
| 225806_at | JUB | jub, ajuba homolog (Xenopus laevis) | AI289311 | 84962 | 1.829069223 | 0.013418345 |
| 238774_at | KANSL1 | KAT8 regulatory NSL complex subunit 1 | AW960454 | 284058 | -2.562388318 | 0.027037111 |
| 223790_at | KATNAL1 | katanin p60 subunit A-like 1 | BC000612 | 84056 | 1.904047532 | 0.002100215 |
| 213478_at | KAZ | kazrin | AB028949 | 23254 | 2.208644789 | 0.028202245 |
| 229144_at | KAZ | kazrin | AA989362 | 23254 | 1.890405933 | 0.02417046 |
| 206765_at | KCNJ2 | potassium inwardly-rectifying channel, subfamily J, member 2 | AF153820 | 3759 | 2.117662367 | 0.029659752 |
| 202503_s_at | KIAA0101 | KIAA0101 | NM_014736 | 9768 | -5.174122172 | 0.002280367 |
| 211713_x_at | KIAA0101 | KIAA0101 | BC005832 | 9768 | -2.097945978 | 0.033147633 |
| 231855_at | KIAA1524 | KIAA1524 | AB040957 | 57650 | -1.769556472 | 0.018830834 |
| 204444_at | KIF11 | kinesin family member 11 | NM_004523 | 3832 | -4.678928254 | 0.002403702 |
| 206364_at | KIF14 | kinesin family member 14 | NM_014875 | 9928 | -3.50215313 | 0.008015194 |
| 219306_at | KIF15 | kinesin family member 15 | NM_020242 | 56992 | -3.667066955 | 0.000685005 |
| 231875_at | KIF21A | kinesin family member 21A | AF155117 | 55605 | -1.756546493 | 0.021792856 |
| 220002_at | KIF26B | kinesin family member 26B | NM_018012 | 55083 | 1.697253096 | 0.04235941 |
| 218355_at | KIF4A | kinesin family member 4A | NM_012310 | 24137 | -3.610370439 | 0.001832712 |
| 236887_at | KIN | KIN, antigenic determinant of recA protein homolog (mouse) | AA768850 | 22944 | -1.663907481 | 0.048020546 |
| 219657_s_at | KLF3 | Kruppel-like factor 3 (basic) | NM_016531 | 51274 | 1.877049305 | 0.020012631 |
| 225963_at | KLHDC5 | kelch domain containing 5 | AW293538 | 57542 | 1.650819348 | 0.043921573 |
| 202058_s_at | KPNA1 | karyopherin alpha 1 (importin alpha 5) | BC002374 | 3836 | 1.646579005 | 0.031521288 |
| 202059_s_at | KPNA1 | karyopherin alpha 1 (importin alpha 5) | NM_002264 | 3836 | 1.841649059 | 0.025373708 |
| 233224_at | KPNA3 | karyopherin alpha 3 (importin alpha 4) | AL137645 | 3839 | -1.941172898 | 0.034274379 |
| 209653_at | KPNA4 | karyopherin alpha 4 (importin alpha 3) | U93240 | 3840 | 1.728820772 | 0.029066939 |
| 1552486_s_at | LACTB | lactamase, beta | NM_171846 | 114294 | 1.687276638 | 0.035903353 |
| 222714_s_at | LACTB2 | lactamase, beta 2 | BC000878 | 51110 | 1.885396425 | 0.00161353 |
| 211354_s_at | LEPR | leptin receptor | U52913 | 3953 | 1.831535262 | 0.038896369 |
| 211355_x_at | LEPR | leptin receptor | U52914 | 3953 | 1.805698199 | 0.026714229 |
| 211356_x_at | LEPR | leptin receptor | U66495 | 3953 | 1.982897916 | 0.033203977 |
| 208949_s_at | LGALS3 | lectin, galactoside-binding, soluble, 3 | BC001120 | 3958 /// 81625 | 2.086374127 | 0.001873652 |
| 208934_s_at | LGALS8 | lectin, galactoside-binding, soluble, 8 | AF342815 | 3964 | 1.684683889 | 0.02066682 |
| 212325_at | LIMCH1 | LIM and calponin homology domains 1 | AK027231 | 22998 | 2.170672803 | 0.043170248 |
| 1554600_s_at | LMNA | lamin A/C | BC033088 | 4000 | 2.06852648 | 0.021815189 |
| 203411_s_at | LMNA | lamin A/C | NM_005572 | 4000 | 2.046763849 | 0.028124897 |
| 238661_at | LOC100130155 | hypothetical protein LOC100130155 | AI374740 | 100130155 | -1.976002573 | 0.034234575 |
| 242193_at | LOC100130155 | hypothetical protein LOC100130155 | D59963 | 100130155 | -1.890672749 | 0.039863567 |
| 228381_at | LOC100287628 | Hypothetical protein LOC100287628 | AV716964 | 80063 | 1.791769094 | 0.001211789 |
| 215109_at | LOC100288007 | hypothetical protein LOC100288007 | R02172 | 57238 | -1.626721665 | 0.033915776 |
| 216967_at | LOC100288551 | hypothetical protein LOC100288551 | AF279774 | 2596 | 3.085722871 | 0.022234198 |
| 1558750_a_at | LOC100288693 | Hypothetical protein LOC100288693 | BG109249 |  | -2.274129039 | 0.018217356 |
| 235267_at | LOC100505881 | hypothetical LOC100505881 | AV732165 | 402560 | 1.66253621 | 0.045279526 |
| 229480_at | LOC100505895 | hypothetical LOC100505895 | AI341053 | 402560 | 1.636336299 | 0.049084533 |
| 238953_at | LOC100506325 | --- | AA993833 | 6668 | 1.816691229 | 0.028153207 |
| 1556194_a_at | LOC100507455 | hypothetical LOC100507455 | BC042959 |  | 1.81108849 | 0.013235242 |
| 240616_at | LOC100507636 | hypothetical LOC100507636 | AV699953 |  | 2.654940812 | 0.001737706 |
| 235707_at | LOC221710 | hypothetical protein LOC221710 | AW994270 | 221710 | 1.731314049 | 0.040535609 |
| 1560250_s_at | LOC284242 | hypothetical protein LOC284242 | BC035844 | 284242 | 1.964846232 | 0.04249973 |
| 228160_at | LOC339290 | hypothetical LOC339290 | AI433706 | 400642 | -1.638656849 | 0.013779465 |
| 239466_at | LOC344595 | hypothetical LOC344595 | AA463827 | 344595 | 1.697724543 | 0.016187314 |
| 235205_at | LOC346887 | similar to solute carrier family 16 (monocarboxylic acid transporters), member 14 | BF109660 | 346887 | 1.727762794 | 0.034648346 |
| 243134_at | LOC440309 | hypothetical LOC440309 | AW190862 | 440309 | -2.00515459 | 0.007649183 |
| 1560258_a_at | LOC646906 | similar to Zinc finger protein 254 | BC035780 | 646906 | -2.158531844 | 0.000190468 |
| 242313_at | LOC728730 | Hypothetical LOC728730 | BG284890 | 728730 | 2.249987828 | 0.000154593 |
| 216574_s_at | LOC729020 /// RPE | rcRPE /// ribulose-5-phosphate-3-epimerase | J04742 | 440001 /// 6120 /// 649755 | 1.702037794 | 0.01590687 |
| 230433_at | LOC729970 | similar to hCG2028352 | BE857101 | 729970 | 1.701928548 | 0.026350273 |
| 235362_at | LOC729970 | Similar to hCG2028352 | AW614589 | 729970 | 1.693449357 | 0.015881301 |
| 219019_at | LRDD | leucine-rich repeats and death domain containing | NM_018494 | 55367 | 1.662276785 | 0.003258261 |
| 221640_s_at | LRDD | leucine-rich repeats and death domain containing | AF274972 | 55367 | 1.942065265 | 0.032581957 |
| 235498_at | LRRIQ3 | leucine-rich repeats and IQ motif containing 3 | AI269596 | 127255 | 1.663600885 | 0.006646906 |
| 225593_at | LSM10 | LSM10, U7 small nuclear RNA associated | AL542359 | 84967 | 1.707389748 | 0.002981928 |
| 241792_x_at | LUC7L3 | LUC7-like 3 (S. cerevisiae) | N36160 | 51747 | -1.755875229 | 0.016508659 |
| 242389_at | LUC7L3 | LUC7-like 3 (S. cerevisiae) | BE887449 | 51747 | -1.965201555 | 0.010401859 |
| 226851_at | LYPLAL1 | lysophospholipase-like 1 | AW612657 | 127018 | 1.918816641 | 0.00082527 |
| 210943_s_at | LYST | lysosomal trafficking regulator | U84744 | 1130 | 1.718693895 | 0.026819765 |
| 203362_s_at | MAD2L1 | MAD2 mitotic arrest deficient-like 1 (yeast) | NM_002358 | 4085 | -3.334280542 | 0.02031015 |
| 223578_x_at | MALAT1 | metastasis associated lung adenocarcinoma transcript 1 (non-protein coding) | AF113016 | 29005 | -1.816317114 | 0.044400041 |
| 208116_s_at | MAN1A1 | mannosidase, alpha, class 1A, member 1 | NM_005907 | 4121 | 2.855437523 | 9.53406E-05 |
| 221760_at | MAN1A1 | Mannosidase, alpha, class 1A, member 1 | BG287153 | 4121 | 3.051147556 | 0.018373057 |
| 218918_at | MAN1C1 | mannosidase, alpha, class 1C, member 1 | NM_020379 | 57134 | 2.048807165 | 0.022617348 |
| 1558732_at | MAP4K4 | mitogen-activated protein kinase kinase kinase kinase 4 | AK074900 | 9448 | -1.821322821 | 0.04875793 |
| 235550_at | MAP9 | microtubule-associated protein 9 | AV751613 | 79884 | 2.227869624 | 0.00126487 |
| 1552263_at | MAPK1 | mitogen-activated protein kinase 1 | NM_138957 | 5594 | 2.183137406 | 0.011728033 |
| 1552264_a_at | MAPK1 | mitogen-activated protein kinase 1 | NM_138957 | 5594 | 1.687442681 | 0.027952948 |
| 208351_s_at | MAPK1 | mitogen-activated protein kinase 1 | NM_002745 | 5594 | 1.644428913 | 0.044608632 |
| 202787_s_at | MAPKAPK3 | mitogen-activated protein kinase-activated protein kinase 3 | U43784 | 7867 | 1.638254144 | 0.02400397 |
| 243552_at | MBTD1 | mbt domain containing 1 | AW008914 | 54799 | -1.645256741 | 0.030848124 |
| 232092_at | MCART1 | mitochondrial carrier triple repeat 1 | AI268054 | 92014 | 1.701569761 | 0.000933541 |
| 212246_at | MCFD2 | multiple coagulation factor deficiency 2 | BE880828 | 90411 | 2.110577628 | 0.004788223 |
| 223570_at | MCM10 | minichromosome maintenance complex component 10 | AL136840 | 55388 | -2.992347433 | 0.014693287 |
| 208795_s_at | MCM7 | minichromosome maintenance complex component 7 | D55716 | 4176 | -1.628169179 | 0.010856967 |
| 205386_s_at | MDM2 | Mdm2 p53 binding protein homolog (mouse) | NM_002392 | 4193 | 3.557603476 | 0.000730752 |
| 217373_x_at | MDM2 | Mdm2 p53 binding protein homolog (mouse) | AJ276888 | 4193 | 2.787826814 | 0.012807156 |
| 217542_at | MDM2 | Mdm2 p53 binding protein homolog (mouse) | BE930512 | 1368 | 1.720010376 | 0.002814123 |
| 225160_x_at | MDM2 | Mdm2 p53 binding protein homolog (mouse) | AI952357 | 84825 | 1.877433556 | 0.022946217 |
| 229711_s_at | MDM2 | Mdm2 p53 binding protein homolog (mouse) | AA902480 | 84825 | 2.137393838 | 0.003700266 |
| 216109_at | MED13L | Mediator complex subunit 13-like | AK025348 | 23389 | -1.743070812 | 0.03987947 |
| 214684_at | MEF2A | myocyte enhancer factor 2A | X63381 | 4205 | 1.798789007 | 0.014739123 |
| 206028_s_at | MERTK | c-mer proto-oncogene tyrosine kinase | NM_006343 | 10461 | 1.716471679 | 0.007227636 |
| 225955_at | METRNL | meteorin, glial cell differentiation regulator-like | BG231494 | 284207 /// 653506 | 1.784721226 | 0.003015843 |
| 242247_at | METT5D1 | methyltransferase 5 domain containing 1 | AL580319 | 196074 | 1.96959914 | 0.002145001 |
| 227055_at | METTL7B | methyltransferase like 7B | AI827972 | 196410 | 4.811454128 | 0.030843301 |
| 1554667_s_at | METTL8 | methyltransferase like 8 | BC025250 | 79828 | 1.68994357 | 0.031749833 |
| 218376_s_at | MICAL1 | microtubule associated monoxygenase, calponin and LIM domain containing 1 | NM_022765 | 64780 | -1.761290847 | 0.022391713 |
| 1555105_a_at | MIER1 | mesoderm induction early response 1 homolog (Xenopus laevis) | AY124188 | 57708 | 1.95986824 | 0.011190214 |
| 226630_at | MIS18BP1 | chromosome 14 open reading frame 106 | BF062175 | 55320 | -2.97450556 | 0.006507365 |
| 204423_at | MKLN1 | muskelin 1, intracellular mediator containing kelch motifs | NM_013255 | 4289 | 1.928443835 | 0.002242482 |
| 218883_s_at | MLF1IP | MLF1 interacting protein | NM_024629 | 79682 | -3.587254983 | 0.004280749 |
| 229305_at | MLF1IP | MLF1 interacting protein | AA460299 | 79682 | -4.005983702 | 0.000450586 |
| 236347_at | MMAA | methylmalonic aciduria (cobalamin deficiency) cblA type | AW300959 | 166785 | 1.803776725 | 0.012143834 |
| 205429_s_at | MPP6 | membrane protein, palmitoylated 6 (MAGUK p55 subfamily member 6) | NM_016447 | 51678 | 1.804412304 | 0.020791409 |
| 225520_at | MTHFD1L | methylenetetrahydrofolate dehydrogenase (NADP+ dependent) 1-like | AL133260 | 25902 | 2.311795616 | 0.039914338 |
| 231094_s_at | MTHFD1L | methylenetetrahydrofolate dehydrogenase (NADP+ dependent) 1-like | AL035086 | 25902 | 2.198367804 | 0.005659421 |
| 242996_at | MTRF1 | mitochondrial translational release factor 1 | AI341686 | 9617 | 1.745919648 | 0.023261598 |
| 213906_at | MYBL1 | v-myb myeloblastosis viral oncogene homolog (avian)-like 1 | AW592266 | 4603 | -2.398573247 | 0.004154003 |
| 1557720_s_at | MYO16 | myosin XVI | BC041343 | 23026 | 2.759030631 | 0.000172583 |
| 215119_at | MYO16 | myosin XVI | AI522028 | 23026 | 2.926777731 | 0.000545784 |
| 204527_at | MYO5A | myosin VA (heavy chain 12, myoxin) | NM_000259 | 4644 | 1.704319235 | 0.003594989 |
| 201798_s_at | MYOF | myoferlin | NM_013451 | 26509 | 5.508191207 | 0.000505153 |
| 217738_at | NAMPT | nicotinamide phosphoribosyltransferase | BF575514 | 10135 /// 646309 /// 651511 | 2.632735985 | 0.048187761 |
| 217739_s_at | NAMPT | nicotinamide phosphoribosyltransferase | NM_005746 | 10135 /// 646309 /// 651511 | 4.216237622 | 0.037447785 |
| 210048_at | NAPG | N-ethylmaleimide-sensitive factor attachment protein, gamma | BC001889 | 8774 | 2.042395263 | 0.006890574 |
| 201969_at | NASP | nuclear autoantigenic sperm protein (histone-binding) | AW003362 | 4678 | -1.73770541 | 0.002092108 |
| 202906_s_at | NBN | nibrin | AF049895 | 4683 | 1.825830557 | 0.034585049 |
| 231532_at | NCAM1 | Neural cell adhesion molecule 1 | BF591692 | 4684 | -1.862700806 | 0.003019162 |
| 218662_s_at | NCAPG | non-SMC condensin I complex, subunit G | NM_022346 | 64151 | -3.496004839 | 0.034390697 |
| 218663_at | NCAPG | non-SMC condensin I complex, subunit G | NM_022346 | 64151 | -3.546428012 | 0.035904988 |
| 225847_at | NCEH1 | neutral cholesterol ester hydrolase 1 | AB037784 | 57552 | 1.950025557 | 0.041587667 |
| 204162_at | NDC80 | NDC80 homolog, kinetochore complex component (S. cerevisiae) | NM_006101 | 10403 | -5.405030153 | 0.000566949 |
| 224799_at | NDFIP2 | Nedd4 family interacting protein 2 | AW290956 | 54602 | 1.874969632 | 0.010168794 |
| 202150_s_at | NEDD9 | neural precursor cell expressed, developmentally down-regulated 9 | U64317 | 4739 | 1.766213181 | 0.005416016 |
| 221805_at | NEFL | neurofilament, light polypeptide | AL537457 | 4747 | 13.36826285 | 0.001494647 |
| 221916_at | NEFL | neurofilament, light polypeptide | BF055311 | 4747 | 4.261738778 | 0.005693824 |
| 219502_at | NEIL3 | nei endonuclease VIII-like 3 (E. coli) | NM_018248 | 55247 | -2.212026853 | 0.004039258 |
| 204641_at | NEK2 | NIMA (never in mitosis gene a)-related kinase 2 | NM_002497 | 4751 | -4.581152249 | 0.00716092 |
| 1552309_a_at | NEXN | nexilin (F actin binding protein) | NM_144573 | 91624 | 1.71222262 | 0.005844042 |
| 226103_at | NEXN | nexilin (F actin binding protein) | AF114264 | 91624 | 1.845145717 | 0.008662526 |
| 230791_at | NFIB | nuclear factor I/B | AU146924 | 4781 | -2.216626761 | 0.015022957 |
| 203045_at | NINJ1 | ninjurin 1 | NM_004148 | 4814 | 1.730074697 | 0.001113435 |
| 226474_at | NLRC5 | NLR family, CARD domain containing 5 | AA005023 | 84166 | 2.036866736 | 0.034628764 |
| 203964_at | NMI | N-myc (and STAT) interactor | NM_004688 | 9111 | 1.875477093 | 0.016482285 |
| 201157_s_at | NMT1 | N-myristoyltransferase 1 | AF020500 | 4836 | 1.719593535 | 0.010181512 |
| 214321_at | NOV | nephroblastoma overexpressed gene | BF440025 | 4856 | 8.738451947 | 0.002066308 |
| 223405_at | NPL | N-acetylneuraminate pyruvate lyase (dihydrodipicolinate synthase) | AI042017 | 80896 | 1.686358481 | 0.022427319 |
| 201467_s_at | NQO1 | NAD(P)H dehydrogenase, quinone 1 | AI039874 | 1728 | 2.43911029 | 0.025277468 |
| 201468_s_at | NQO1 | NAD(P)H dehydrogenase, quinone 1 | NM_000903 | 1728 | 1.90704978 | 0.028191613 |
| 210519_s_at | NQO1 | NAD(P)H dehydrogenase, quinone 1 | BC000906 | 1728 | 2.799951024 | 0.016434051 |
| 202647_s_at | NRAS | neuroblastoma RAS viral (v-ras) oncogene homolog | NM_002524 | 4893 | 1.921622233 | 0.00713777 |
| 222344_at | NREP | neuronal regeneration related protein homolog (rat) | AW972765 | 9315 | -2.04481637 | 0.037554341 |
| 206343_s_at | NRG1 | neuregulin 1 | NM_013959 | 3084 | 3.338991843 | 0.035016256 |
| 214632_at | NRP2 | neuropilin 2 | AA295257 | 8828 | 2.717307427 | 0.013434261 |
| 1569181_x_at | NSF | N-ethylmaleimide-sensitive factor | BC017896 | 4905 | -2.215059552 | 0.043665461 |
| 223177_at | NT5DC1 | 5'-nucleotidase domain containing 1 | AL515061 | 221294 | 2.061301375 | 0.00376364 |
| 223381_at | NUF2 | NUF2, NDC80 kinetochore complex component, homolog (S. cerevisiae) | AF326731 | 83540 | -5.972608156 | 5.52784E-05 |
| 205135_s_at | NUFIP1 | nuclear fragile X mental retardation protein interacting protein 1 | AL049842 | 26747 | 1.72300131 | 0.021753826 |
| 218295_s_at | NUP50 | nucleoporin 50kDa | NM_007172 | 10762 | 1.657554541 | 0.041952376 |
| 209230_s_at | NUPR1 | nuclear protein, transcriptional regulator, 1 | AF135266 | 26471 | 2.270335588 | 0.027324757 |
| 218039_at | NUSAP1 | nucleolar and spindle associated protein 1 | NM_016359 | 51203 | -5.124683186 | 0.000556183 |
| 219978_s_at | NUSAP1 | nucleolar and spindle associated protein 1 | NM_018454 | 51203 | -4.471627228 | 0.002071132 |
| 219100_at | OBFC1 | oligonucleotide/oligosaccharide-binding fold containing 1 | NM_024928 | 79991 | 1.764010574 | 0.046475671 |
| 233085_s_at | OBFC2A | oligonucleotide/oligosaccharide-binding fold containing 2A | AV734843 | 64859 | 1.7612893 | 0.011504492 |
| 219277_s_at | OGDHL | oxoglutarate dehydrogenase-like | NM_018245 | 55753 | 1.78135875 | 0.036105807 |
| 213599_at | OIP5 | Opa interacting protein 5 | BE045993 | 11339 | -2.589979368 | 0.019009969 |
| 219105_x_at | ORC6L | origin recognition complex, subunit 6 like (yeast) | NM_014321 | 23594 | -2.102048566 | 0.020835141 |
| 209626_s_at | OSBPL3 | oxysterol binding protein-like 3 | AI202969 | 26031 | 1.896361435 | 0.016840225 |
| 1554414_a_at | OSGIN2 | oxidative stress induced growth inhibitor family member 2 | BC031054 | 734 | 2.424728492 | 0.012243904 |
| 1554008_at | OSMR | oncostatin M receptor | BC010943 | 9180 | 1.863916565 | 0.028569614 |
| 205729_at | OSMR | oncostatin M receptor | NM_003999 | 9180 | 1.959962921 | 0.017444891 |
| 226621_at | OSMR | oncostatin M receptor | AI133452 | 9180 | 2.13900164 | 0.000380607 |
| 202733_at | P4HA2 | prolyl 4-hydroxylase, alpha polypeptide II | NM_004199 | 8974 | 3.234941346 | 0.005045933 |
| 200815_s_at | PAFAH1B1 | platelet-activating factor acetylhydrolase 1b, regulatory subunit 1 (45kDa) | L13386 | 5048 | 1.85660905 | 0.04727218 |
| 210160_at | PAFAH1B2 | platelet-activating factor acetylhydrolase 1b, catalytic subunit 2 (30kDa) | BC000398 | 5049 | 1.751426691 | 0.007143811 |
| 220315_at | PARP11 | poly (ADP-ribose) polymerase family, member 11 | NM_020367 | 57097 | 2.011845146 | 0.003531911 |
| 223220_s_at | PARP9 | poly (ADP-ribose) polymerase family, member 9 | AF307338 | 83666 | 1.691793026 | 0.04551913 |
| 232144_at | PBX1 | pre-B-cell leukemia homeobox 1 | AV710542 | 5087 | -1.888456242 | 0.022069041 |
| 233273_at | PBX1 | pre-B-cell leukemia homeobox 1 | AU146834 | 5087 | -2.122790351 | 0.011816904 |
| 203557_s_at | PCBD1 | pterin-4 alpha-carbinolamine dehydratase/dimerization cofactor of hepatocyte nuclear factor 1 alpha | NM_000281 | 5092 | 1.968052444 | 0.013589215 |
| 231043_at | PCDP1 | primary ciliary dyskinesia protein 1 | AV719360 | 200373 | 1.837495782 | 0.000610733 |
| 214239_x_at | PCGF2 | polycomb group ring finger 2 | AI560455 | 7703 | -1.650466558 | 0.019815356 |
| 227935_s_at | PCGF5 | polycomb group ring finger 5 | AA522681 | 84333 | 1.724123811 | 0.009223621 |
| 211564_s_at | PDLIM4 | PDZ and LIM domain 4 | BC003096 | 8572 | 1.985384154 | 0.02068651 |
| 214175_x_at | PDLIM4 | PDZ and LIM domain 4 | AI254547 | 8572 | 1.795613249 | 0.003388392 |
| 218691_s_at | PDLIM4 | PDZ and LIM domain 4 | AF153882 | 8572 | 2.468847724 | 0.003268667 |
| 1566551_at | PDZRN3 | PDZ domain containing ring finger 3 | AL137307 | 23024 | -1.955078042 | 0.01897347 |
| 232776_at | PDZRN3 | PDZ domain containing ring finger 3 | AU145289 | 23024 | -1.840320812 | 0.046385947 |
| 233442_at | PDZRN3 | PDZ domain containing ring finger 3 | AU147500 | 23024 | -2.083886867 | 0.014594183 |
| 223469_at | PGPEP1 | pyroglutamyl-peptidase I | BC004942 | 54858 | 1.777122427 | 0.001291401 |
| 218634_at | PHLDA3 | pleckstrin homology-like domain, family A, member 3 | NM_012396 | 23612 | 2.088136445 | 0.001159003 |
| 235980_at | PIK3CA | Phosphoinositide-3-kinase, catalytic, alpha polypeptide | AA767763 | 5290 | -1.744298387 | 0.01557513 |
| 204958_at | PLK3 | polo-like kinase 3 (Drosophila) | NM_004073 | 1263 | 2.037752497 | 0.00022631 |
| 204886_at | PLK4 | polo-like kinase 4 (Drosophila) | AL043646 | 10733 | -2.442775135 | 0.005013808 |
| 204887_s_at | PLK4 | polo-like kinase 4 (Drosophila) | NM_014264 | 10733 | -2.931738979 | 0.001486089 |
| 202446_s_at | PLSCR1 | phospholipid scramblase 1 | AI825926 | 5359 | 2.018234597 | 0.017626909 |
| 227276_at | PLXDC2 | plexin domain containing 2 | AI264121 | 84898 | 2.175528158 | 0.016766016 |
| 203622_s_at | PNO1 | partner of NOB1 homolog (S. cerevisiae) | NM_020143 | 56902 | 1.779750013 | 0.010026773 |
| 218511_s_at | PNPO | pyridoxamine 5'-phosphate oxidase | NM_018129 | 55163 | 1.66009473 | 0.001396029 |
| 219380_x_at | POLH | polymerase (DNA directed), eta | NM_006502 | 5429 | 1.750794551 | 0.001135245 |
| 231115_at | POLH | polymerase (DNA directed), eta | AI890529 | 54676 | 1.93248656 | 0.003238262 |
| 233852_at | POLH | polymerase (DNA directed), eta | AK025631 | 5429 | 1.782769167 | 0.008542769 |
| 212841_s_at | PPFIBP2 | PTPRF interacting protein, binding protein 2 (liprin beta 2) | AI692180 | 8495 | 2.113986105 | 0.021062699 |
| 226907_at | PPP1R14C | protein phosphatase 1, regulatory (inhibitor) subunit 14C | N32557 | 81706 | 2.49246006 | 0.039143136 |
| 202884_s_at | PPP2R1B | protein phosphatase 2, regulatory subunit A, beta | NM_002716 | 5519 | 1.64388342 | 0.012171456 |
| 212527_at | PPPDE2 | PPPDE peptidase domain containing 2 | BF057059 | 27351 | 1.838326927 | 0.00216608 |
| 218009_s_at | PRC1 | protein regulator of cytokinesis 1 | NM_003981 | 9055 | -3.839124868 | 0.000818498 |
| 212216_at | PREPL | prolyl endopeptidase-like | AW000954 | 9581 | 1.813727181 | 0.011244843 |
| 205053_at | PRIM1 | primase, DNA, polypeptide 1 (49kDa) | NM_000946 | 5557 | -1.73905833 | 0.027915358 |
| 209799_at | PRKAA1 | protein kinase, AMP-activated, alpha 1 catalytic subunit | AF100763 | 5562 | 2.179978374 | 0.003155591 |
| 200604_s_at | PRKAR1A | protein kinase, cAMP-dependent, regulatory, type I, alpha (tissue specific extinguisher 1) | M18468 | 5573 | 1.739152602 | 0.045372394 |
| 228536_at | PRMT10 | protein arginine methyltransferase 10 (putative) | AA574240 | 90826 | 1.766427782 | 0.018124959 |
| 228273_at | PRR11 | proline rich 11 | BG165011 | 55771 | -2.576276238 | 0.032951364 |
| 203405_at | PSMG1 | proteasome (prosome, macropain) assembly chaperone 1 | NM_003720 | 8624 | 1.637477584 | 0.039304452 |
| 1555910_at | PTCD2 | pentatricopeptide repeat domain 2 | AK056761 | 79810 | 1.643357784 | 0.002476417 |
| 209815_at | PTCH1 | patched homolog 1 (Drosophila) | BG054916 | 5727 | -1.738733916 | 0.031321891 |
| 204053_x_at | PTEN | phosphatase and tensin homolog | U96180 | 5728 | 1.629114639 | 0.047163893 |
| 1559529_at | PTK2 | PTK2 protein tyrosine kinase 2 | BC043202 | 5747 | -1.700480579 | 0.035825911 |
| 40524_at | PTPN21 | protein tyrosine phosphatase, non-receptor type 21 | X79510 | 11099 | 1.658131614 | 0.017143168 |
| 208790_s_at | PTRF | polymerase I and transcript release factor | AF312393 | 284119 | 1.790062817 | 0.036450062 |
| 203554_x_at | PTTG1 | pituitary tumor-transforming 1 | NM_004219 | 9232 | -2.395746612 | 0.011223319 |
| 227718_at | PURB | purine-rich element binding protein B | BF337790 | 5814 | 2.293272679 | 0.000813885 |
| 1558290_a_at | PVT1 | Pvt1 oncogene (non-protein coding) | BG200951 | 441378 /// 5820 | 2.633508172 | 0.010428383 |
| 207018_s_at | RAB27B | RAB27B, member RAS oncogene family | NM_004163 | 5874 | 1.718277101 | 0.035717062 |
| 204214_s_at | RAB32 | RAB32, member RAS oncogene family | NM_006834 | 10981 | 1.628061945 | 0.037695494 |
| 219412_at | RAB38 | RAB38, member RAS oncogene family | NM_022337 | 23682 | 1.687185741 | 0.038689783 |
| 244170_at | RAB3C | RAB3C, member RAS oncogene family | H05254 | 115827 | 1.97132827 | 0.001173456 |
| 202374_s_at | RAB3GAP2 | RAB3 GTPase activating protein subunit 2 (non-catalytic) | NM_012414 | 25782 | 1.850522257 | 0.009542172 |
| 230266_at | RAB7B | RAB7B, member RAS oncogene family | AI127991 | 338382 | 2.717039656 | 0.025190089 |
| 219210_s_at | RAB8B | RAB8B, member RAS oncogene family | NM_016530 | 51762 | 1.79986215 | 0.028818978 |
| 203223_at | RABEP1 | rabaptin, RAB GTPase binding effector protein 1 | NM_004703 | 9135 | 1.724928605 | 0.023060451 |
| 213982_s_at | RABGAP1L | RAB GTPase activating protein 1-like | BG107203 | 9910 | 1.990163468 | 0.009587052 |
| 222077_s_at | RACGAP1 | Rac GTPase activating protein 1 | AU153848 | 29127 | -2.552523523 | 0.020309113 |
| 205024_s_at | RAD51 | RAD51 homolog (RecA homolog, E. coli) (S. cerevisiae) | NM_002875 | 5888 | -3.518482667 | 0.017714227 |
| 204146_at | RAD51AP1 | RAD51 associated protein 1 | BE966146 | 10635 | -3.203945254 | 0.009210749 |
| 209849_s_at | RAD51C | RAD51 homolog C (S. cerevisiae) | AF029669 | 5889 | 1.808096156 | 0.00032115 |
| 221738_at | RALGAPB | Ral GTPase activating protein, beta subunit (non-catalytic) | BG236163 | 57148 | 1.817636529 | 0.005945933 |
| 224678_at | RALGAPB | Ral GTPase activating protein, beta subunit (non-catalytic) | AL132998 | 57148 | 1.801155371 | 0.009450552 |
| 1555339_at | RAP1A | RAP1A, member of RAS oncogene family | AB051846 | 5906 | 164.9036341 | 8.74449E-10 |
| 1555340_x_at | RAP1A | RAP1A, member of RAS oncogene family | AB051846 | 5906 | 176.0335401 | 1.30782E-11 |
| 243673_at | RBM41 | RNA binding motif protein 41 | BG536728 | 55285 | -1.973481949 | 2.82685E-05 |
| 237702_at | RBM45 | RNA binding motif protein 45 | AW451271 | 129831 | 1.639932373 | 0.016401318 |
| 218035_s_at | RBM47 | RNA binding motif protein 47 | NM_019027 | 54502 | 2.933672299 | 0.035954002 |
| 222496_s_at | RBM47 | RNA binding motif protein 47 | AW241742 | 54502 | 2.202982046 | 0.013459977 |
| 209488_s_at | RBPMS | RNA binding protein with multiple splicing | D84109 | 11030 | 2.502681121 | 0.046329005 |
| 208370_s_at | RCAN1 | regulator of calcineurin 1 | NM_004414 | 1827 | 1.967088641 | 0.019786107 |
| 208872_s_at | REEP5 | receptor accessory protein 5 | AA814140 | 7905 | 1.67511484 | 0.012818622 |
| 223989_s_at | REXO2 | REX2, RNA exonuclease 2 homolog (S. cerevisiae) | BC003502 | 25996 | 1.647439289 | 0.000289703 |
| 239142_at | RFESD | Rieske (Fe-S) domain containing | AI082852 | 317671 | 2.014214779 | 0.002112423 |
| 203225_s_at | RFK | riboflavin kinase | NM_018339 | 55312 | 1.737848411 | 0.006909088 |
| 238810_at | RFX3 | regulatory factor X, 3 (influences HLA class II expression) | H97074 | 5991 | 2.399683054 | 0.024227532 |
| 227339_at | RGMB | RGM domain family, member B | BE206621 | 285704 | 1.946757102 | 0.033341661 |
| 242450_at | RGMB | RGM domain family, member B | AW004714 | 285704 | 2.610848689 | 0.00109897 |
| 226945_at | RHBDD1 | rhomboid domain containing 1 | AI356895 | 84236 | 1.836346041 | 0.010398484 |
| 214449_s_at | RHOQ | ras homolog gene family, member Q | NM_012249 | 23433 | 1.784894771 | 0.029996937 |
| 226365_at | RMI2 | --- | W03242 | 10571 /// 643998 | -2.040567189 | 0.027005441 |
| 219104_at | RNF141 | ring finger protein 141 | NM_016422 | 50862 | 1.818135941 | 0.002909041 |
| 235492_at | RNF217 | ring finger protein 217 | AI888256 | 154214 | 1.925528214 | 0.006860149 |
| 221909_at | RNFT2 | ring finger protein, transmembrane 2 | AW299700 | 84900 | 1.632626611 | 0.032132435 |
| 202684_s_at | RNMT | RNA (guanine-7-) methyltransferase | AB020966 | 8731 | 2.26695127 | 0.003381898 |
| 221634_at | RPL23AP7 | ribosomal protein L23a pseudogene 7 | BC000596 | 118433 | 1.692325747 | 0.005615558 |
| 213427_at | RPP40 | ribonuclease P/MRP 40kDa subunit | NM_006638 | 10799 | 1.647266327 | 0.031293865 |
| 236002_at | RPS2 | ribosomal protein S2 | BE178502 | 6187 | -1.626870793 | 0.040655457 |
| 212590_at | RRAS2 | related RAS viral (r-ras) oncogene homolog 2 | AI431643 | 22800 | 1.671539842 | 0.012800912 |
| 201477_s_at | RRM1 | ribonucleotide reductase M1 | NM_001033 | 6240 | -1.649925668 | 0.014375629 |
| 201890_at | RRM2 | ribonucleotide reductase M2 | BE966236 | 6241 | -5.966494362 | 0.003642023 |
| 209773_s_at | RRM2 | ribonucleotide reductase M2 | BC001886 | 6241 | -4.245117084 | 0.043555164 |
| 223342_at | RRM2B | ribonucleotide reductase M2 B (TP53 inducible) | AB036063 | 50484 | 1.729028601 | 0.002276425 |
| 203594_at | RTCD1 | RNA terminal phosphate cyclase domain 1 | NM_003729 | 8634 | 1.629684852 | 0.000116069 |
| 230469_at | RTKN2 | rhotekin 2 | AW665138 | 219790 | -3.323557646 | 0.024816994 |
| 200660_at | S100A11 | S100 calcium binding protein A11 | NM_005620 | 6282 | 3.535258815 | 0.002152173 |
| 209686_at | S100B | S100 calcium binding protein B | BC001766 | 6285 | 1.817873491 | 0.04001119 |
| 212845_at | SAMD4A | sterile alpha motif domain containing 4A | AB028976 | 23034 | 1.685547779 | 0.0467793 |
| 223843_at | SCARA3 | scavenger receptor class A, member 3 | AB007830 | 51435 | 1.809787636 | 0.009046865 |
| 204035_at | SCG2 | secretogranin II | NM_003469 | 7857 | 3.219546231 | 0.012100235 |
| 206381_at | SCN2A | sodium channel, voltage-gated, type II, alpha subunit | NM_021007 | 6326 | 1.924304242 | 0.00026347 |
| 229057_at | SCN2A | sodium channel, voltage-gated, type II, alpha subunit | BF432956 | 6326 | 3.11432378 | 4.78848E-05 |
| 235225_at | SCN2B | sodium channel, voltage-gated, type II, beta | AA447729 | 6327 | 1.961140444 | 0.002737005 |
| 241436_at | SCNN1G | sodium channel, nonvoltage-gated 1, gamma | AI985987 | 6340 | 2.915284803 | 0.024188725 |
| 228274_at | SDSL | serine dehydratase-like | BE963955 | 113675 | 1.845079516 | 0.006915622 |
| 202083_s_at | SEC14L1 | SEC14-like 1 (S. cerevisiae) | AI017770 | 6397 | 1.681487636 | 0.045259581 |
| 212902_at | SEC24A | SEC24 family, member A (S. cerevisiae) | BE645231 | 10802 | 1.714640489 | 0.019925023 |
| 244841_at | SEC24A | SEC24 family, member A (S. cerevisiae) | AI080364 | 10802 | 1.763365461 | 0.009363349 |
| 223225_s_at | SEH1L | SEH1-like (S. cerevisiae) | AI201534 | 81929 | 1.804354656 | 0.024578205 |
| 215204_at | SENP6 | SUMO1/sentrin specific peptidase 6 | AU147295 | 26054 | -2.082006987 | 0.033846396 |
| 208940_at | SEPHS1 | selenophosphate synthetase 1 | AI885670 | 22929 | -1.737506813 | 0.000185453 |
| 1553167_a_at | SEPSECS | Sep (O-phosphoserine) tRNA:Sec (selenocysteine) tRNA synthase | BC023539 | 51091 | 2.15077595 | 0.004751348 |
| 231730_at | SEPSECS | Sep (O-phosphoserine) tRNA:Sec (selenocysteine) tRNA synthase | NM_016955 | 51091 | 2.351380786 | 0.000997886 |
| 223394_at | SERTAD1 | SERTA domain containing 1 | BC002670 | 29950 | 2.22965855 | 0.029523079 |
| 223195_s_at | SESN2 | sestrin 2 | BF131886 | 83667 | 2.365462515 | 0.000377964 |
| 223196_s_at | SESN2 | sestrin 2 | AL136551 | 83667 | 2.044845351 | 0.01557473 |
| 217226_s_at | SFXN3 | sideroflexin 3 | M95929 | 81855 | 1.735061726 | 0.017052674 |
| 205120_s_at | SGCB | sarcoglycan, beta (43kDa dystrophin-associated glycoprotein) | U29586 | 6443 | 1.658184579 | 0.012684891 |
| 230165_at | SGOL2 | shugoshin-like 2 (S. pombe) | N31731 | 151246 | -2.29508915 | 0.043445279 |
| 229978_at | SHISA9 | shisa homolog 9 (Xenopus laevis) | AU143918 | 440338 | 2.739442762 | 0.023329264 |
| 224391_s_at | SIAE | sialic acid acetylesterase | AF303378 | 54414 | 1.66023425 | 0.024035534 |
| 204157_s_at | SIK3 | SIK family kinase 3 | NM_025164 | 23387 | 2.006657481 | 0.019392754 |
| 225639_at | SKAP2 | src kinase associated phosphoprotein 2 | N21390 | 8935 | 1.82385079 | 0.035289847 |
| 224845_s_at | SLAIN2 | SLAIN motif family, member 2 | AU159395 | 57606 | 1.661290907 | 0.000110535 |
| 204404_at | SLC12A2 | solute carrier family 12 (sodium/potassium/chloride transporters), member 2 | NM_001046 | 6558 | 1.981869518 | 0.032055441 |
| 205896_at | SLC22A4 | solute carrier family 22 (organic cation/ergothioneine transporter), member 4 | NM_003059 | 6583 | 1.845550077 | 0.047191024 |
| 1557411_s_at | SLC25A43 | solute carrier family 25, member 43 | AK094254 | 203427 | 1.981916306 | 0.035737938 |
| 228181_at | SLC30A1 | solute carrier family 30 (zinc transporter), member 1 | AI553933 | 7779 | 2.431818664 | 0.011683259 |
| 229500_at | SLC30A9 | solute carrier family 30 (zinc transporter), member 9 | AI609256 | 10463 | 1.672351227 | 0.00033862 |
| 209713_s_at | SLC35D1 | solute carrier family 35 (UDP-glucuronic acid/UDP-N-acetylgalactosamine dual transporter), member D1 | AB044343 | 23169 | 1.83666433 | 0.017771866 |
| 1568623_a_at | SLC35E4 | solute carrier family 35, member E4 | BC040191 | 339665 | 1.668402679 | 0.009483523 |
| 214830_at | SLC38A6 | solute carrier family 38, member 6 | AI537540 | 145389 | 1.631739564 | 0.038695623 |
| 200924_s_at | SLC3A2 | solute carrier family 3 (activators of dibasic and neutral amino acid transport), member 2 | NM_002394 | 6520 | 1.720066277 | 0.036941639 |
| 243894_at | SLC41A2 | solute carrier family 41, member 2 | BG169689 | 84102 | 2.177706467 | 0.005851726 |
| 219525_at | SLC47A1 | solute carrier family 47, member 1 | NM_018242 | 55244 | 2.1113699 | 0.028834891 |
| 223748_at | SLC4A11 | solute carrier family 4, sodium borate transporter, member 11 | AF336127 | 83959 | 2.194821172 | 0.00081703 |
| 1555141_a_at | SLC9B1 | Na+/H+ exchanger domain containing 1 | BC022079 | 150159 | 1.817314448 | 0.006848891 |
| 1565703_at | SMAD4 | SMAD family member 4 | AL832789 | 4089 | -2.026660612 | 0.02334291 |
| 227719_at | SMAD9 | SMAD family member 9 | AA934610 | 4093 | -2.13904324 | 0.012219395 |
| 1561973_at | SMARCC2 | SWI/SNF related, matrix associated, actin dependent regulator of chromatin, subfamily c, member 2 | AL833124 | 6601 | -1.686317466 | 0.02610482 |
| 213253_at | SMC2 | structural maintenance of chromosomes 2 | AU154486 | 10592 | -2.356569398 | 0.020374398 |
| 201664_at | SMC4 | structural maintenance of chromosomes 4 | AL136877 | 10051 | -1.631096553 | 0.006263948 |
| 1557987_at | SMG1P1 | smg-1 homolog, phosphatidylinositol 3-kinase-related kinase pseudogene 1 | BC042832 | 641298 | -1.658805443 | 0.037032885 |
| 222784_at | SMOC1 | SPARC related modular calcium binding 1 | AJ249900 | 64093 | -2.106824596 | 0.029605308 |
| 233713_at | SMYD2 | SET and MYND domain containing 2 | AK022181 | 56950 | -2.091995403 | 0.043659491 |
| 212921_at | SMYD2 | SET and MYND domain containing 2 | AF070592 | 56950 | -1.803842283 | 0.010473322 |
| 222286_at | SNAPC3 | small nuclear RNA activating complex, polypeptide 3, 50kDa | R43279 | 6619 | -1.636283747 | 0.025083386 |
| 207474_at | SNRK | SNF related kinase | NM_017719 | 54861 | -1.708799831 | 0.003278271 |
| 1560741_at | SNRPN | small nuclear ribonucleoprotein polypeptide N | AL832250 | 6638 | -1.730137295 | 0.018944911 |
| 214708_at | SNTB1 | syntrophin, beta 1 (dystrophin-associated protein A1, 59kDa, basic component 1) | BG484314 | 6641 | 2.138936496 | 0.018241183 |
| 218404_at | SNX10 | sorting nexin 10 | NM_013322 | 29887 | 2.792034226 | 0.001023329 |
| 1552792_at | SOCS4 | suppressor of cytokine signaling 4 | NM_080867 | 122809 | 2.234915016 | 0.002983144 |
| 212807_s_at | SORT1 | sortilin 1 | BF447105 | 6272 | 1.776746967 | 0.007977796 |
| 233369_at | SOS2 | son of sevenless homolog 2 (Drosophila) | AU146027 | 6655 | -1.945938738 | 0.024144905 |
| 224178_s_at | SOX6 | SRY (sex determining region Y)-box 6 | AL136780 | 55553 | 1.891921535 | 0.020477579 |
| 1557804_at | SP3 | Sp3 transcription factor | N23846 | 6670 | -1.87149382 | 0.012011001 |
| 203145_at | SPAG5 | sperm associated antigen 5 | NM_006461 | 10615 | -2.16517615 | 0.019508781 |
| 229331_at | SPATA18 | spermatogenesis associated 18 homolog (rat) | AI559300 | 132671 | 3.063992401 | 1.30211E-06 |
| 214965_at | SPATA2L | spermatogenesis associated 2-like | AF070574 | 124044 | 1.75708788 | 0.045951107 |
| 209891_at | SPC25 | SPC25, NDC80 kinetochore complex component, homolog (S. cerevisiae) | AF225416 | 57405 | -3.266560566 | 0.011228605 |
| 213993_at | SPON1 | spondin 1, extracellular matrix protein | AI885290 | 10418 | -1.739647977 | 0.018190375 |
| 204011_at | SPRY2 | sprouty homolog 2 (Drosophila) | NM_005842 | 10253 | 1.747010873 | 0.031861925 |
| 201471_s_at | SQSTM1 | sequestosome 1 | NM_003900 | 8878 | 1.84517814 | 0.003223798 |
| 224130_s_at | SRA1 | steroid receptor RNA activator 1 | AF293026 | 10011 | 1.776264188 | 0.009205012 |
| 224864_at | SRA1 | steroid receptor RNA activator 1 | BG397813 | 10011 | 1.660947099 | 0.003594726 |
| 210959_s_at | SRD5A1 | steroid-5-alpha-reductase, alpha polypeptide 1 (3-oxo-5 alpha-steroid delta 4-dehydrogenase alpha 1) | AF113128 | 6715 | 2.26917685 | 0.005417418 |
| 204955_at | SRPX | sushi-repeat-containing protein, X-linked | NM_006307 | 8406 | 2.130859212 | 0.008035726 |
| 225252_at | SRXN1 | sulfiredoxin 1 homolog (S. cerevisiae) | AL121758 | 140809 | 1.752644612 | 0.04845297 |
| 238861_at | SSBP2 | single-stranded DNA binding protein 2 | AI692322 | 23635 | -1.737460335 | 0.023478547 |
| 200889_s_at | SSR1 | signal sequence receptor, alpha | AI016620 | 6745 | 1.660064939 | 0.023859403 |
| 222411_s_at | SSR3 | signal sequence receptor, gamma (translocon-associated protein gamma) | AW087870 | 6747 | 1.653308033 | 0.037611242 |
| 1552738_a_at | ST7L | suppression of tumorigenicity 7 like | NM_138729 | 54879 | 1.630800231 | 0.01409652 |
| 210073_at | ST8SIA1 | ST8 alpha-N-acetyl-neuraminide alpha-2,8-sialyltransferase 1 | L32867 | 6489 | 2.133549163 | 0.007597946 |
| 222801_s_at | STAG3L4 | stromal antigen 3-like 4 | BF056088 | 64940 | 1.656874057 | 0.007341642 |
| 215044_s_at | STAM2 | signal transducing adaptor molecule (SH3 domain and ITAM motif) 2 | AI571996 | 10254 | 2.517680933 | 0.003543794 |
| 200887_s_at | STAT1 | signal transducer and activator of transcription 1, 91kDa | NM_007315 | 6772 | 1.721730204 | 0.005842839 |
| 218424_s_at | STEAP3 | STEAP family member 3 | NM_018234 | 55240 | 2.154587175 | 0.005422678 |
| 205339_at | STIL | SCL/TAL1 interrupting locus | NM_003035 | 6491 | -2.694718305 | 0.013775119 |
| 239353_at | STK32A | serine/threonine kinase 32A | AW204033 | 202374 | 2.216102334 | 0.02189532 |
| 223852_s_at | STK40 | serine/threonine kinase 40 | BC005169 | 83931 | 1.752465246 | 0.036422174 |
| 224724_at | SULF2 | sulfatase 2 | AL133001 | 55959 | 3.558899296 | 7.81695E-05 |
| 233555_s_at | SULF2 | sulfatase 2 | AL034418 | 55959 | 3.161702163 | 0.001250397 |
| 209306_s_at | SWAP70 | SWAP switching B-cell complex 70kDa subunit | AI139569 | 23075 | 1.636719097 | 0.027992862 |
| 217833_at | SYNCRIP | synaptotagmin binding, cytoplasmic RNA interacting protein | AL520908 | 10492 | -1.647942773 | 0.012326051 |
| 236146_at | SYNCRIP | synaptotagmin binding, cytoplasmic RNA interacting protein | BF593158 | 10492 | -1.676208664 | 0.001909827 |
| 220325_at | TAF7L | TAF7-like RNA polymerase II, TATA box binding protein (TBP)-associated factor, 50kDa | NM_024885 | 54457 | 2.582097788 | 1.05321E-05 |
| 202307_s_at | TAP1 | transporter 1, ATP-binding cassette, sub-family B (MDR/TAP) | NM_000593 | 6890 | 2.15102868 | 0.010823207 |
| 1557609_s_at | TBC1D12 | TBC1 domain family, member 12 | AW188458 | 23232 | 1.720935588 | 0.013809439 |
| 205796_at | TCP11L1 | t-complex 11 (mouse)-like 1 | NM_018393 | 55346 | 1.644900424 | 0.015515054 |
| 243198_at | TEX9 | testis expressed 9 | AA020920 | 374618 | 1.718641068 | 0.01628768 |
| 209909_s_at | TGFB2 | transforming growth factor, beta 2 | M19154 | 7043 | 1.859652052 | 0.018769116 |
| 220407_s_at | TGFB2 | transforming growth factor, beta 2 | NM_003238 | 7042 | 1.66697185 | 0.027874969 |
| 1558922_at | TIA1 | TIA1 cytotoxic granule-associated RNA binding protein | CA776505 | 7072 | -2.326109724 | 0.029545625 |
| 201449_at | TIA1 | TIA1 cytotoxic granule-associated RNA binding protein | AL567227 | 7072 | -1.769197786 | 0.009766529 |
| 219099_at | TIGAR | chromosome 12 open reading frame 5 | NM_020375 | 57103 | 2.538806742 | 0.000240293 |
| 201666_at | TIMP1 | TIMP metallopeptidase inhibitor 1 | NM_003254 | 7076 | 1.840820437 | 0.013119252 |
| 203167_at | TIMP2 | TIMP metallopeptidase inhibitor 2 | NM_003255 | 7077 | 1.857336428 | 0.04684989 |
| 212770_at | TLE3 | transducin-like enhancer of split 3 (E(sp1) homolog, Drosophila) | AW873621 | 7090 | -1.676470446 | 0.014541966 |
| 206271_at | TLR3 | toll-like receptor 3 | NM_003265 | 7098 | 2.2981949 | 0.010749381 |
| 232068_s_at | TLR4 | toll-like receptor 4 | AF177765 | 7099 | 1.6 | 0.048563 |
| 213882_at | TM2D1 | TM2 domain containing 1 | AA012917 | 83941 | -1.917673131 | 0.01557426 |
| 202195_s_at | TMED5 | transmembrane emp24 protein transport domain containing 5 | NM_016040 | 50999 | 1.637735222 | 0.030198333 |
| 224321_at | TMEFF2 | transmembrane protein with EGF-like and two follistatin-like domains 2 | AB004064 | 23671 | -1.823213489 | 0.047815139 |
| 1554076_s_at | TMEM136 | transmembrane protein 136 | BC015232 | 219902 | 1.690885643 | 0.009969716 |
| 228505_s_at | TMEM170A | transmembrane protein 170A | N49836 | 124491 | 1.72944822 | 0.004387292 |
| 219253_at | TMEM185B | transmembrane protein 185B (pseudogene) | NM_024121 | 79134 | 1.889785998 | 0.002698802 |
| 218113_at | TMEM2 | transmembrane protein 2 | NM_013390 | 23670 | -1.72709168 | 0.007865885 |
| 239776_at | TMEM232 | transmembrane protein 232 | AI027091 | 642987 | 2.041819434 | 0.000335179 |
| 227936_at | TMEM68 | transmembrane protein 68 | AI671172 | 137695 | 1.627661131 | 0.018464142 |
| 225411_at | TMEM87B | transmembrane protein 87B | AI769794 | 84910 | 1.767897545 | 0.019282413 |
| 225412_at | TMEM87B | transmembrane protein 87B | AA761169 | 84910 | 1.688796957 | 0.012949856 |
| 219701_at | TMOD2 | tropomodulin 2 (neuronal) | NM_014548 | 29767 | 1.851422408 | 0.03159312 |
| 203432_at | TMPO | thymopoietin | AW272611 | 7112 | -1.769555042 | 0.012337697 |
| 224944_at | TMPO | thymopoietin | AL566034 | 7112 | -1.937531662 | 0.029517903 |
| 224397_s_at | TMTC1 | transmembrane and tetratricopeptide repeat containing 1 | AF319520 | 653626 /// 83857 | 2.1238572 | 0.017237527 |
| 231775_at | TNFRSF10A | tumor necrosis factor receptor superfamily, member 10a | W65310 | 8797 | 2.435555324 | 0.001099138 |
| 210405_x_at | TNFRSF10B | tumor necrosis factor receptor superfamily, member 10b | AF153687 | 8795 | 2.460215795 | 0.000127955 |
| 227345_at | TNFRSF10D | tumor necrosis factor receptor superfamily, member 10d, decoy with truncated death domain | AI738556 | 8793 | 2.450350746 | 0.042967486 |
| 204932_at | TNFRSF11B | tumor necrosis factor receptor superfamily, member 11b | BF433902 | 4982 | 2.219032845 | 0.014254488 |
| 207643_s_at | TNFRSF1A | tumor necrosis factor receptor superfamily, member 1A | NM_001065 | 7132 | 1.711326455 | 0.001632001 |
| 213109_at | TNIK | TRAF2 and NCK interacting kinase | N25621 | 23043 | 1.659560298 | 0.018093412 |
| 201291_s_at | TOP2A | topoisomerase (DNA) II alpha 170kDa | AU159942 | 7153 | -6.779445989 | 0.001816706 |
| 201746_at | TP53 | tumor protein p53 | NM_000546 | 7157 | 1.784469432 | 0.04744 |
| 210609_s_at | TP53I3 | tumor protein p53 inducible protein 3 | BC000474 | 9540 | 2.002530109 | 0.002598508 |
| 204140_at | TPST1 | tyrosylprotein sulfotransferase 1 | NM_003596 | 8460 | 1.629119535 | 0.031000578 |
| 204079_at | TPST2 | tyrosylprotein sulfotransferase 2 | NM_003595 | 8459 | 2.051893433 | 0.02200168 |
| 218403_at | TRIAP1 | TP53 regulated inhibitor of apoptosis 1 | NM_016399 | 51499 | 1.801932726 | 0.00039988 |
| 218145_at | TRIB3 | tribbles homolog 3 (Drosophila) | NM_021158 | 57761 | 2.002224411 | 0.015885731 |
| 203148_s_at | TRIM14 | tripartite motif-containing 14 | NM_014788 | 9830 | 1.893629774 | 0.04025617 |
| 1569316_at | TRIM24 | tripartite motif-containing 24 | BC009590 | 8805 | -1.686991196 | 0.039480781 |
| 227801_at | TRIM59 | tripartite motif-containing 59 | N90779 | 286827 | -1.937022739 | 0.008806752 |
| 209778_at | TRIP11 | thyroid hormone receptor interactor 11 | AF007217 | 9321 | 1.754672467 | 0.02469658 |
| 227862_at | TRNP1 | TMF1-regulated nuclear protein 1 | AA037766 | 388610 | 1.923731584 | 0.016756237 |
| 243483_at | TRPM8 | transient receptor potential cation channel, subfamily M, member 8 | AI272941 | 79054 | 2.054770908 | 0.021090351 |
| 238462_at | UBASH3B | ubiquitin associated and SH3 domain containing B | AI418293 | 84959 | 1.658909672 | 0.045865325 |
| 238587_at | UBASH3B | ubiquitin associated and SH3 domain containing B | AI927919 | 84959 | 1.715058366 | 0.020274171 |
| 202954_at | UBE2C | ubiquitin-conjugating enzyme E2C | NM_007019 | 11065 | -4.922284352 | 0.000916442 |
| 225787_at | UBE2F | ubiquitin-conjugating enzyme E2F (putative) | AI799788 | 140739 | 1.755429062 | 0.003913072 |
| 231948_s_at | UBE2F | ubiquitin-conjugating enzyme E2F (putative) | BG171548 | 140739 | 1.66885896 | 0.002616891 |
| 223229_at | UBE2T | ubiquitin-conjugating enzyme E2T (putative) | AB032931 | 29089 | -3.212559785 | 0.001249363 |
| 205687_at | UBFD1 | ubiquitin family domain containing 1 | NM_019116 | 56061 | 1.784469432 | 0.011767548 |
| 222989_s_at | UBQLN1 | ubiquilin 1 | NM_013438 | 29979 | 1.650652166 | 0.005622142 |
| 225655_at | UHRF1 | ubiquitin-like with PHD and ring finger domains 1 | AK025578 | 29128 | -2.911815624 | 0.009783977 |
| 1568781_at | UROS | uroporphyrinogen III synthase | BC024936 | 7390 | 1.869443557 | 0.013880859 |
| 202412_s_at | USP1 | ubiquitin specific peptidase 1 | AW499935 | 7398 | -1.730543035 | 0.03451894 |
| 231990_at | USP15 | ubiquitin specific peptidase 15 | AK023703 | 9958 | 1.626102335 | 0.026553115 |
| 226652_at | USP3 | ubiquitin specific peptidase 3 | AA523542 | 9960 | -1.714497471 | 0.007252558 |
| 223602_at | USP30 | ubiquitin specific peptidase 30 | BC004868 | 84749 | 1.933027361 | 0.004317968 |
| 224978_s_at | USP36 | ubiquitin specific peptidase 36 | BF430981 | 57602 | 1.721310652 | 0.029522645 |
| 228050_at | UTP15 | UTP15, U3 small nucleolar ribonucleoprotein, homolog (S. cerevisiae) | AA046406 | 84135 | 1.720497518 | 0.005249386 |
| 231697_s_at | VMP1 | vacuole membrane protein 1 | AV660825 | 81671 | 2.170562363 | 0.044812168 |
| 235138_at | VPS35 | vacuolar protein sorting 35 homolog (S. cerevisiae) | AA565051 | 55737 | -2.183084617 | 0.037674872 |
| 242957_at | VWCE | von Willebrand factor C and EGF domains | AI862096 | 220001 | 1.760040389 | 0.016516054 |
| 243087_at | WDR63 | WD repeat domain 63 | AI860874 | 126820 | 4.37474589 | 0.00030606 |
| 239680_at | WDR76 | WD repeat domain 76 | AI220472 | 79968 | -3.093660718 | 0.03470999 |
| 200670_at | XBP1 | X-box binding protein 1 | NM_005080 | 7494 | 1.881829763 | 0.020762022 |
| 206536_s_at | XIAP | X-linked inhibitor of apoptosis | U32974 | 331 | 1.87038244 | 0.035516864 |
| 218479_s_at | XPO4 | exportin 4 | NM_022459 | 64328 | 1.87038249 | 0.037579792 |
| 1555785_a_at | XRN1 | 5'-3' exoribonuclease 1 | AY137776 | 54464 | 1.947212337 | 0.028740998 |
| 226437_at | YIF1B | Yip1 interacting factor homolog B (S. cerevisiae) | AI079540 | 90522 | 1.708774091 | 0.01315091 |
| 217785_s_at | YKT6 | YKT6 v-SNARE homolog (S. cerevisiae) | NM_006555 | 10652 | 2.167415083 | 0.002956576 |
| 1564053_a_at | YTHDF3 | YTH domain family, member 3 | AK093081 | 253943 | 2.387418258 | 0.003084514 |
| 1554973_a_at | ZBTB26 | zinc finger and BTB domain containing 26 | BC018748 | 57684 | 1.693231264 | 0.01150652 |
| 222730_s_at | ZDHHC2 | zinc finger, DHHC-type containing 2 | AI814257 | 51201 | 1.814922421 | 0.002986549 |
| 238791_at | ZNF100 | zinc finger protein 100 | AA282536 | 163227 | -1.750007333 | 0.00011913 |
| 1557384_at | ZNF131 | Zinc finger protein 131 | AL832081 | 7690 | -1.750855204 | 0.012933976 |
| 238631_at | ZNF140 | Zinc finger protein 140 | AA490928 | 7699 | -1.722761925 | 0.011218149 |
| 222227_at | ZNF236 | zinc finger protein 236 | AK000847 | 7776 | 3.798565789 | 0.037703346 |
| 206261_at | ZNF239 | zinc finger protein 239 | NM_005674 | 8187 | 1.664687816 | 0.019576444 |
| 1559449_a_at | ZNF254 | Zinc finger protein 254 | BF679633 | 399655 | -1.842592941 | 0.0082841 |
| 206448_at | ZNF365 | zinc finger protein 365 | NM_014951 | 22891 | 1.669049261 | 0.004641675 |
| 229551_x_at | ZNF367 | zinc finger protein 367 | N62196 | 195828 | -3.377285113 | 0.003620389 |
| 235513_at | ZNF398 | zinc finger protein 398 | AW131450 | 57541 | -1.696527553 | 0.041631567 |
| 226113_at | ZNF436 | zinc finger protein 436 | AB051497 | 80818 | 1.701205477 | 0.003723531 |
| 226114_at | ZNF436 | zinc finger protein 436 | AI829509 | 80818 | 1.655983851 | 0.00375893 |
| 208081_s_at | ZNF442 | zinc finger protein 442 | NM_030824 | 79973 | 1.941634963 | 0.048011506 |
| 1552427_at | ZNF485 | zinc finger protein 485 | NM_145312 | 220992 | 1.641880715 | 0.006767679 |
| 1557283_a_at | ZNF519 | zinc finger protein 519 | BC037787 | 162655 | -1.669582025 | 0.006225362 |
| 230205_at | ZNF561 | zinc finger protein 561 | BF437602 | 93134 | 2.293363449 | 0.002632833 |
| 232641_at | ZNF596 | zinc finger protein 596 | AC004908 | 169270 | 1.704101994 | 0.013307748 |
| 1557270_at | ZNF69 | zinc finger protein 69 | AA632049 | 7620 | -1.763761867 | 0.035508563 |
| 223366_at | ZNF704 | zinc finger protein 704 | BC004287 | 619279 | -1.729466127 | 0.039459033 |
| 241906_at | ZNF708 | zinc finger protein 708 | BF001773 | 7562 | -2.03885347 | 0.0238838 |
| 204026_s_at | ZWINT | ZW10 interactor | NM_007057 | 11130 | -3.644267402 | 0.003922773 |

**Table S2.** Full list of modulated genes scoring a p value ≤ 0.05 and belonging to IPA categories scoring a Fisher test ≤ 0.05. Genes and categories were recollected into three main biological functions: a) cell cycle and DNA damage, b) immune response and c) trafficking and remodeling.

1. cell cycle and DNA damage

|  | **2h** | | | **5d** | | |
| --- | --- | --- | --- | --- | --- | --- |
| ***genes*** | **HD-HAd** | **HD-CAV-2** | **LV** | **HD-HAd** | **HD-CAV-2** | **LV** |
| ***ABCD3*** | -2.38 | - | - | -2.10 | - | - |
| ***ACTN1*** | - | - | - | 1.76 | 1.98 | 1.72 |
| ***ACTN4*** | - | - | - | 1.86 | - | - |
| ***ACVR2A*** | - | - | - | - | - | -1.86 |
| ***ADRB1*** | -1.93 | - | - | - | - | - |
| ***AEN*** | - | - | - | - | - | 1.66 |
| ***AGRN*** | - | - | - | 1.64 | - | - |
| ***AGTRAP*** | - | - | - | - | 1.64 | 1.83 |
| ***AJUBA*** | - | - | - | - | 1.72 | 1.74 |
| ***AKR1C1/AKR1C2*** | - | - | - | - | -2.70 | - |
| ***AKT1*** | - | - | - | 1.72 | - | - |
| ***AKT1S1*** | - | - | - | 1.73 | - | - |
| ***AKT3*** | - | - | - | - | -1.77 | - |
| ***ALKBH8*** | - | - | - | - | - | 1.64 |
| ***ALS2*** | -2.14 | - | - | - | - | - |
| ***ANXA1*** | - | - | - | - | - | 2.66 |
| ***ANXA2*** | - | - | - | - | - | 2.99 |
| ***APC*** | -2.26 | - | - | - | - | - |
| ***APH1B*** | - | - | - | - | - | 1.72 |
| ***API5*** | - | - | - | -1.71 | - | - |
| ***AR*** | - | - | - | 1.96 | - | - |
| ***ARHGAP35*** | - | - | - | - | - | -1.64 |
| ***ARHGEF2*** | - | - | - | - | - | -1.69 |
| ***ARHGEF7*** | 2.59 | - | - | - | - | - |
| ***ARID1A*** | - | - | - | 2.31 | - | - |
| ***ARID1B*** | - | - | - | - | -2.39 | - |
| ***ARL1*** | - | - | - | - | - | 1.80 |
| ***ARL8B*** | - | - | - | - | - | 1.73 |
| ***ARNTL*** | -1.83 | - | - | - | - | - |
| ***ASAH1*** | -1.78 | - | - | - | - | - |
| ***ASPM*** | - | - | - | - | - | -3.99 |
| ***ATAD2*** | - | - | - | - | - | -2.64 |
| ***ATF3*** | - | - | - | - | - | 6.59 |
| ***ATXN3*** | - | - | - | - | -1.66 | - |
| ***AURKA*** | - | - | - | - | - | -2.95 |
| ***BAG4*** | - | - | - | - | - | 1.93 |
| ***BANF1*** | - | - | - | 1.99 | - | - |
| ***BCL10*** | -1.96 | - | - | - | - | - |
| ***BCL11B*** | - | - | - | - | -4.73 | - |
| ***BCL2L12*** | - | - | - | - | 1.70 | - |
| ***BIRC5*** | - | - | - | - | 2.13 | -10.00 |
| ***BLM*** | - | - | - | - | 1.97 | - |
| ***BMP2K*** | -1.78 | - | - | - | - | - |
| ***BOK*** | - | - | - | 1.90 | - | - |
| ***BRAF*** | -1.97 | - | - | - | - | - |
| ***BRCA2*** | - | - | - | - | 3.25 | - |
| ***BRD7*** | - | - | - | -1.76 | - | - |
| ***BRIP1*** | - | - | - | - | - | -4.71 |
| ***BUB1*** | - | - | - | - | - | -3.20 |
| ***C11orf82*** | - | - | - | - | 4.88 | - |
| ***C6orf108*** | - | - | - | - | 1.65 | - |
| ***CACNB3*** | - | - | - | - | -1.78 | - |
| ***CALR*** | - | - | - | 1.76 | - | - |
| ***CAMK1*** | - | - | - | - | 1.66 | - |
| ***CAMK2D*** | - | - | - | - | - | 1.63 |
| ***CAP2*** | -1.78 | - | - | - | - | - |
| ***CAPNS1*** | - | - | - | 1.64 | - | - |
| ***CARS*** | - | - | - | - | - | 1.63 |
| ***CASP1*** | - | - | - | - | 1.81 | 1.85 |
| ***CAST*** | - | - | - | - | - | 1.98 |
| ***CBFB*** | - | - | - | - | - | 1.79 |
| ***CBX4*** | - | - | - | 3.02 | - | - |
| ***CBX5*** | - | - | - | - | -1.94 | -2.06 |
| ***CCDC86*** | - | - | - | - | - | 1.63 |
| ***CCDC99*** | - | - | - | - | 1.62 | - |
| ***CCNA2*** | - | - | - | - | - | -2.45 |
| ***CCNB1*** | - | - | - | - | - | -3.64 |
| ***CCNB2*** | - | - | - | - | - | -5.18 |
| ***CCND1*** | - | - | - | - | - | 2.71 |
| ***CCNE1*** | - | - | - | - | 1.88 | - |
| ***CCNE2*** | - | - | - | - | - | -2.83 |
| ***CD164*** | - | - | - | - | - | 2.88 |
| ***CD44*** | - | - | - | - | 1.85 | 2.55 |
| ***CD46*** | - | - | - | - | -1.70 | - |
| ***CD58*** | - | - | - | - | 1.78 | 1.64 |
| ***CDC14B*** | - | - | - | - | - | 2.02 |
| ***CDC25A*** | - | - | - | - | 1.69 | -1.85 |
| ***CDC25B*** | - | - | - | - | 2.32 | - |
| ***CDC25C*** | - | - | - | - | 2.62 | -2.32 |
| ***CDCA5*** | - | - | - | - | 2.42 | -2.77 |
| ***CDK1*** | - | - | - | - | - | -5.46 |
| ***CDKN1A*** | - | - | - | 2.03 | 2.45 | 3.29 |
| ***CDKN1C*** | -2.12 | - | - | - | -2.31 | - |
| ***CDKN2C*** | - | - | - | - | - | -2.65 |
| ***CDT1*** | - | - | - | - | 1.71 | -2.71 |
| ***CEBPB*** | - | - | - | - | - | 2.89 |
| ***CEBPG*** | - | - | - | - | - | 2.10 |
| ***CENPA*** | - | - | - | - | - | -4.29 |
| ***CENPF*** | - | - | - | - | 2.21 | -3.80 |
| ***CENPH*** | - | - | - | - | 2.40 | -1.90 |
| ***CENPI*** | - | - | - | - | 2.04 | - |
| ***CENPJ*** | - | - | - | - | - | -1.77 |
| ***CENPW*** | - | - | - | - | 2.88 | -1.73 |
| ***CEP135*** | - | - | - | - | 2.08 | - |
| ***CEP72*** | - | - | - | - | 2.05 | - |
| ***CERK*** | - | - | - | - | -1.70 | - |
| ***CES2*** | - | - | - | - | - | 1.83 |
| ***CFDP1*** | - | - | - | - | - | -1.75 |
| ***CFI*** | - | - | - | - | 4.39 | - |
| ***CFL1*** | - | - | - | 1.99 | - | - |
| ***CHAF1A*** | - | - | - | - | 1.80 | - |
| ***CHEK1*** | - | - | - | - | 2.26 | -2.04 |
| ***CHEK2*** | - | - | - | - | 3.62 | - |
| ***CHRNA7*** | - | - | - | - | - | 1.65 |
| ***CKS1B*** | - | - | - | - | 2.74 | -1.86 |
| ***CKS2*** | - | - | - | - | 2.21 | -2.35 |
| ***CLIP1*** | - | - | - | - | -1.83 | - |
| ***CNR1*** | -2.10 | - | - | -2.20 | - | - |
| ***COL4A1*** | - | - | - | - | - | 2.90 |
| ***COX11*** | - | - | - | - | 2.04 | - |
| ***CREBBP*** | - | - | - | - | - | -1.71 |
| ***CRYAB*** | - | - | - | 1.94 | 2.00 | 2.21 |
| ***CSDA*** | - | - | - | - | 2.18 | 2.63 |
| ***CSRNP3*** | - | - | - | - | -1.79 | - |
| ***CTSC*** | - | - | - | - | 2.99 | - |
| ***CTSL2*** | - | - | - | - | 2.87 | - |
| ***CX3CL1*** | - | - | - | - | 1.77 | 1.99 |
| ***CYP7B1*** | - | - | - | - | - | 1.93 |
| ***DBC1*** | - | - | - | - | -2.59 | - |
| ***DBF4B*** | - | - | - | - | 2.17 | - |
| ***DCAF7*** | -1.76 | - | - | - | - | - |
| ***DCTN1*** | - | - | - | 2.09 | - | - |
| ***DDB2*** | - | - | - | - | - | 1.68 |
| ***DDIT3*** | - | - | - | - | - | 2.31 |
| ***DDX17*** | -2.20 | - | - | - | -2.40 | - |
| ***DEGS1*** | -1.95 | - | - | - | - | - |
| ***DEPDC1*** | - | - | - | - | 2.95 | - |
| ***DIAPH2*** | - | - | - | - | - | 1.66 |
| ***DICER1*** | - | - | - | - | -1.79 | - |
| ***DLG1*** | -1.76 | - | - | - | - | - |
| ***DNA2*** | - | - | - | - | - | -2.01 |
| ***DNM3*** | - | - | - | - | -1.66 | - |
| ***DRAM1*** | - | - | - | - | 2.16 | 2.22 |
| ***DSCC1*** | - | - | - | - | 1.63 | - |
| ***DTL*** | - | - | - | - | - | -4.57 |
| ***DTX3L*** | - | - | - | - | - | 1.76 |
| ***DUSP14*** | - | - | - | - | 1.64 | 1.73 |
| ***DUSP3*** | - | - | - | - | 1.82 | 2.32 |
| ***DYRK2*** | -1.83 | - | - | - | - | - |
| ***E2F7*** | - | - | - | - | 2.82 | - |
| ***ECT2*** | - | - | - | - | 2.46 | -2.34 |
| ***EDIL3*** | - | - | - | - | - | 2.35 |
| ***EFEMP1*** | - | - | - | 2.16 | - | - |
| ***EFNA5*** | 2.33 | - | - | - | - | - |
| ***EGLN3*** | - | - | - | - | - | -1.92 |
| ***EGR1*** | - | - | - | - | - | 4.71 |
| ***EGR2*** | - | - | - | - | - | 4.87 |
| ***EHD4*** | - | - | - | - | 2.47 | 1.75 |
| ***EHMT2*** | - | - | - | 1.77 | - | - |
| ***EIF1*** | - | - | - | - | -1.71 | - |
| ***EIF2AK4*** | - | - | - | - | 1.63 | - |
| ***EIF2C2*** | -1.95 | - | - | - | - | - |
| ***EIF5A2*** | - | - | - | - | - | 1.74 |
| ***EIF5B*** | - | - | - | -1.66 | - | - |
| ***ELAVL1*** | - | - | - | - | -1.64 | - |
| ***EME1*** | - | - | - | - | 1.95 | -1.65 |
| ***EMP1*** | - | - | - | - | - | 8.31 |
| ***EMP3*** | - | - | - | 2.21 | - | 1.67 |
| ***ENPP2*** | - | - | - | - | 2.86 | 2.10 |
| ***EPB41L3*** | -1.94 | - | - | - | - | - |
| ***EPHA2*** | - | - | - | 1.94 | 2.42 | 2.85 |
| ***EPHA4*** | - | - | - | - | - | -2.33 |
| ***ERBB3*** | - | - | - | - | 1.77 | - |
| ***ERBB4*** | - | - | - | - | -1.83 | - |
| ***ERCC6L*** | - | - | - | - | 2.32 | - |
| ***ERF*** | - | - | - | - | 1.78 | 1.78 |
| ***ERI1*** | - | - | - | - | 1.70 | - |
| ***ESCO2*** | - | - | - | - | 1.95 | - |
| ***ETV5*** | - | - | - | - | - | 3.90 |
| ***EYA3*** | - | - | - | - | - | 1.62 |
| ***EZH2*** | - | - | - | - | - | -1.79 |
| ***FABP5*** | - | - | - | - | - | 1.84 |
| ***FAH*** | - | - | - | - | 1.82 | 1.81 |
| ***FAM176C*** | - | - | - | - | - | 2.80 |
| ***FANCD2*** | - | - | - | - | 2.37 | -5.92 |
| ***FANCI*** | - | - | - | - | 2.83 | -2.25 |
| ***FANCM*** | - | - | - | - | 2.27 | - |
| ***FAS*** | - | - | - | - | 4.25 | 3.76 |
| ***FBXO5*** | - | - | - | - | - | -1.95 |
| ***FDFT1*** | -1.84 | - | - | - | - | - |
| ***FDXR*** | - | - | - | - | 2.49 | 3.48 |
| ***FGFR1*** | - | - | - | - | - | 1.66 |
| ***FGFR1OP*** | - | - | - | - | - | 2.01 |
| ***FHIT*** | - | - | - | - | - | 1.64 |
| ***FHL1*** | -1.92 | - | - | - | - | - |
| ***FIP1L1*** | -2.00 | - | - | - | - | - |
| ***FKBP4*** | - | - | - | 1.63 | 1.64 | - |
| ***FOLR1*** | 1.63 | - | - | 1.84 | - | - |
| ***FOXM1*** | - | - | - | - | 3.42 | - |
| ***FSCN1*** | - | - | - | 1.75 | - | - |
| ***FXN*** | - | - | - | - | 1.98 | 1.85 |
| ***GADD45A*** | - | - | - | - | - | 3.93 |
| ***GALNT2*** | - | - | - | - | - | 1.98 |
| ***GAP43*** | - | - | - | - | - | 2.94 |
| ***GAS6*** | - | - | - | 1.92 | - | - |
| ***GDF15*** | - | - | - | - | 4.33 | 10.40 |
| ***GINS1*** | - | - | - | - | 2.22 | - |
| ***GLIPR1*** | - | - | - | - | 1.98 | - |
| ***GMNN*** | - | - | - | - | 1.76 | - |
| ***GNAI2*** | - | - | - | 1.68 | - | - |
| ***GNAS*** | -1.85 | - | - | - | - | - |
| ***GPC4*** | - | - | - | - | - | -1.68 |
| ***GPM6A*** | - | - | - | - | -1.92 | - |
| ***GPX1*** | - | - | - | - | 1.67 | 1.98 |
| ***GRB10*** | - | - | - | - | - | 1.75 |
| ***GRIA1*** | - | - | - | - | 1.71 | 1.87 |
| ***GRIA3*** | - | - | - | - | - | -1.68 |
| ***GRINA*** | - | - | - | 1.70 | - | - |
| ***GSG2*** | - | - | - | - | 2.18 | - |
| ***GSPT1*** | - | - | - | - | - | -1.67 |
| ***GSTM1*** | - | - | - | 1.68 | - | - |
| ***H2AFX*** | - | - | - | - | - | -1.77 |
| ***HAP1*** | - | - | - | - | - | 2.50 |
| ***HAS3*** | - | - | - | - | 1.95 | 2.08 |
| ***HAUS6*** | - | - | - | - | 1.97 | - |
| ***HBB*** | - | - | 1.67 | - | - | - |
| ***HELLS*** | - | - | - | - | 1.83 | - |
| ***HEXB*** | - | - | - | - | 1.64 | 2.07 |
| ***HIP1*** | -1.75 | - | - | - | - | - |
| ***HLA-A*** | - | - | - | - | - | 1.66 |
| ***HLA-C*** | - | - | - | - | - | 2.11 |
| ***HMGB1*** | - | - | - | - | - | -1.85 |
| ***HNRNPM*** | - | - | - | - | -1.71 | - |
| ***HNRNPR*** | - | - | - | - | - | -1.78 |
| ***HNRNPU*** | - | - | - | - | -1.76 | -1.88 |
| ***HPRT1*** | -1.91 | - | - | - | - | - |
| ***HRAS*** | - | - | - | - | - | 1.64 |
| ***HSP90AA1*** | -1.95 | - | - | - | - | - |
| ***HSPB1*** | - | - | - | - | 1.82 | 1.94 |
| ***ID1*** | 14.10 | - | 12.10 | - | - | - |
| ***ID2*** | 2.30 | - | 2.13 | - | - | - |
| ***ID3*** | 15.30 | - | 7.56 | - | - | - |
| ***IFI6*** | - | - | - | - | - | 2.13 |
| ***IFITM1*** | - | - | - | - | - | 2.33 |
| ***IFITM3*** | - | - | - | - | - | 2.87 |
| ***IGFBP5*** | - | - | - | 1.97 | - | - |
| ***IKBIP*** | - | - | - | - | 2.68 | - |
| ***IL13RA1*** | - | - | - | - | - | 1.98 |
| ***ILF3*** | -1.88 | - | - | - | - | - |
| ***ILK*** | - | - | - | 1.80 | - | - |
| ***INSIG1*** | -3.04 | - | - | - | - | -1.79 |
| ***INSR*** | - | - | - | - | -1.65 | - |
| ***IQGAP3*** | - | - | - | - | 2.31 | - |
| ***IRF3*** | - | - | - | 1.94 | - | - |
| ***IRS1*** | - | - | - | - | - | 1.69 |
| ***ISG15*** | - | - | - | - | - | 2.35 |
| ***ITCH*** | - | - | - | - | - | 1.75 |
| ***ITGA2*** | - | - | - | - | 1.72 | - |
| ***ITGB5*** | - | - | - | - | - | 1.78 |
| ***ITGB8*** | -2.15 | - | - | - | - | 2.07 |
| ***JAG1*** | -1.90 | - | - | - | - | - |
| ***KCNJ2*** | - | - | - | - | - | 2.12 |
| ***KCNIP2*** | 1.74 | - | - | 1.94 | - | - |
| ***KCNMA1*** | 1.96 | - | - | - | - | - |
| ***KIAA0101*** | - | - | - | - | - | -5.17 |
| ***KIF11*** | - | - | - | - | - | -4.68 |
| ***KIF14*** | - | - | - | - | 2.76 | -3.50 |
| ***KIF15*** | - | - | - | - | 2.79 | -3.67 |
| ***KIF18A*** | - | - | - | - | 2.58 | - |
| ***KIF1A*** | -1.68 | - | - | - | - | - |
| ***KIF20B*** | - | - | - | - | 1.93 | - |
| ***KIF22*** | - | - | - | - | 1.70 | - |
| ***KIF4A*** | - | - | - | - | - | -3.61 |
| ***KIN*** | - | - | - | - | - | -1.66 |
| ***KLF3*** | - | - | - | - | - | 1.88 |
| ***KLF6*** | -1.69 | - | - | - | - | - |
| ***KPNA1*** | - | - | - | - | - | 1.84 |
| ***LATS2*** | -1.63 | - | - | - | - | - |
| ***LDLR*** | -2.39 | - | - | - | - | - |
| ***LEPR*** | 2.05 | - | - | - | - | 1.80 |
| ***LGALS3*** | - | - | - | - | - | 2.09 |
| ***LGALS8*** | - | - | - | - | - | 1.68 |
| ***LGMN*** | - | - | - | - | 1.63 | - |
| ***LIG1*** | - | - | - | - | 2.03 | - |
| ***LIN9*** | - | - | - | - | 1.96 | - |
| ***LMNA*** | - | - | - | - | - | 2.07 |
| ***LPAR2*** | - | - | - | - | -2.02 | - |
| ***LRP1*** | - | - | - | 1.82 | - | - |
| ***LRP8*** | - | - | - | - | -1.73 | - |
| ***LSM10*** | - | - | - | - | - | 1.71 |
| ***LSM11*** | - | - | - | - | 1.66 | - |
| ***LUC7L3*** | - | - | - | -1.77 | -2.53 | -1.96 |
| ***LUM*** | - | - | - | - | 3.71 | - |
| ***MAD2L1*** | - | - | - | - | 2.68 | -3.33 |
| ***MAP1B*** | -2.07 | - | - | - | - | - |
| ***MAP4K4*** | - | - | - | - | -2.17 | -1.82 |
| ***MAP9*** | - | - | - | - | - | 2.23 |
| ***MAPK1*** | - | - | - | - | - | 2.18 |
| ***MAPK8*** | - | - | - | - | -2.14 | - |
| ***MASTL*** | - | - | - | - | 2.48 | - |
| ***MCAM*** | - | - | - | - | 2.48 | - |
| ***MCFD2*** | - | - | - | - | - | 2.11 |
| ***MCM10*** | - | - | - | - | - | - |
| ***MCM7*** | - | - | - | - | - | -1.63 |
| ***MDK*** | - | - | - | 2.56 | - | - |
| ***MDM2*** | 1.77 | - | - | - | 2.04 | 3.56 |
| ***MEF2A*** | - | - | - | - | - | 1.80 |
| ***MERTK*** | - | - | - | - | - | 1.72 |
| ***MGST1*** | - | - | - | - | 2.65 | - |
| ***MICAL1*** | - | - | - | - | -1.81 | -1.76 |
| ***MINA*** | - | - | - | - | 1.68 | - |
| ***MLL*** | -1.76 | - | - | - | - | - |
| ***MLL3*** | -1.85 | - | - | - | -1.69 | - |
| ***MRE11A*** | - | - | - | - | 2.29 | - |
| ***MYBL1*** | - | - | - | - | 2.21 | -2.40 |
| ***MYC*** | -2.02 | - | - | - | - | - |
| ***MYO16*** | - | - | - | - | 2.16 | 2.76 |
| ***MYO6*** | -1.81 | - | - | - | - | - |
| ***MYOF*** | - | - | - | - | 2.67 | 5.51 |
| ***NAB1*** | -2.46 | - | - | - | - | - |
| ***NAGA*** | - | - | - | - | 1.63 | - |
| ***NAMPT*** | - | - | - | - | - | 4.21 |
| ***NASP*** | - | - | - | - | - | -1.74 |
| ***NBN*** | - | - | - | - | - | 1.83 |
| ***NAV1*** | -2.20 | - | - | - | - | - |
| ***NCAM1*** | - | - | - | - | -2.22 | -1.86 |
| ***NCAPG*** | - | - | - | - | - | -3.50 |
| ***NCAPH*** | - | - | - | - | 2.31 | - |
| ***NDC80*** | - | - | - | - | - | -5.41 |
| ***NEDD9*** | - | - | - | - | - | 1.77 |
| ***NEFL*** | - | - | - | - | 6.04 | 13.37 |
| ***NEIL3*** | - | - | - | - | 2.76 | -2.21 |
| ***NEK2*** | - | - | - | - | - | -4.58 |
| ***NF1*** | -1.69 | - | - | - | - | - |
| ***NFIB*** | -1.91 | - | - | - | - | - |
| ***NFKBIA*** | - | - | 1.97 | - | - | - |
| ***NFYA*** | -1.63 | - | - | - | - | - |
| ***NIPBL*** | - | - | - | - | -1.64 | - |
| ***NLK*** | -1.68 | - | - | - | - | - |
| ***NMB*** | - | - | - | - | 2.56 | - |
| ***NME1*** | - | - | - | - | 1.78 | - |
| ***NME2*** | - | - | - | - | 1.84 | - |
| ***NMI*** | -1.70 | - | - | - | - | 1.87 |
| ***NOG*** | 3.19 | - | - | - | - | - |
| ***NOV*** | - | - | - | - | 5.02 | 8.74 |
| ***NQO1*** | - | - | - | - | - | 2.80 |
| ***NR4A2*** | - | - | - | -1.73 | - | - |
| ***NRAS*** | - | - | - | - | - | 1.92 |
| ***NREP*** | - | - | - | - | -2.03 | - |
| ***NRG1*** | - | - | - | - | - | 3.33 |
| ***NRP2*** | - | - | - | - | - | 2.72 |
| ***NUAK2*** | - | - | - | 1.69 | - | 2.00 |
| ***NUCKS1*** | - | - | - | -2.15 | - | - |
| ***NUF2*** | - | - | - | - | - | -5.97 |
| ***NUP107*** | - | - | - | - | 1.81 | - |
| ***NUPR1*** | - | - | - | - | - | 2.27 |
| ***NUSAP1*** | - | - | - | - | - | -5.12 |
| ***OBFC1*** | - | - | - | - | - | 1.76 |
| ***OBFC2A*** | - | - | - | - | 2.68 | 1.76 |
| ***ORC6*** | - | - | - | - | 2.10 | -2.10 |
| ***OSMR*** | - | - | - | - | 1.71 | 2.14 |
| ***P4HB*** | -1.71 | - | - | - | - | - |
| ***PAFAH1B1*** | - | - | - | - | - | 1.86 |
| ***PAFAH1B2*** | - | - | - | - | - | 1.75 |
| ***PALLD*** | -1.80 | - | - | - | - | - |
| ***PBK*** | - | - | - | - | 3.20 | - |
| ***PCGF2*** | - | - | - | - | - | -1.65 |
| ***PDE1A*** | - | - | - | -1.94 | - | - |
| ***PDLIM4*** | - | - | - | - | 1.86 | 1.99 |
| ***PFN1*** | - | - | - | 2.56 | 2.10 | - |
| ***PHIP*** | - | - | - | - | 1.76 | - |
| ***PHLDA3*** | - | - | - | - | 1.67 | 2.09 |
| ***PIDD*** | - | - | - | - | - | 1.66 |
| ***PIGA*** | -1.64 | - | - | - | - | - |
| ***PIK3CA*** | - | - | - | - | -1.75 | -1.74 |
| ***PIK3R1*** | - | - | - | -1.66 | - | - |
| ***PIP4K2A*** | 1.77 | - | - | -1.99 | -1.63 | - |
| ***PKD1*** | - | - | - | 1.64 | - | - |
| ***PKM2*** | - | - | - | 2.70 | - | - |
| ***PLD3*** | - | - | - | 2.21 | - | - |
| ***PLK3*** | - | - | - | - | 1.71 | 2.04 |
| ***PLK4*** | - | - | - | - | 2.99 | -2.93 |
| ***PLSCR1*** | - | - | - | - | - | 2.02 |
| ***PMF1*** | - | - | - | 1.71 | 1.66 | - |
| ***PNN*** | - | - | - | -1.80 | - | - |
| ***PNP*** | - | - | - | - | 1.80 | - |
| ***PNPLA6*** | - | - | - | 1.70 | - | - |
| ***POLH*** | - | - | - | - | 1.91 | 1.75 |
| ***POLQ*** | - | - | - | - | 3.09 | - |
| ***PPIF*** | - | - | - | - | 2.03 | - |
| ***PPP2R1B*** | - | - | - | - | - | 1.64 |
| ***PRAF2*** | - | - | - | 1.64 | - | - |
| ***PRC1*** | - | - | - | - | 2.20 | -3.83 |
| ***PRDM2*** | 1.96 | - | - | - | -1.80 | - |
| ***PRDX6*** | - | - | - | - | 1.64 | - |
| ***PRKAA1*** | - | - | - | - | - | 2.18 |
| ***PRKAR1A*** | - | - | - | - | - | 1.74 |
| ***PRPF4B*** | -1.74 | - | - | - | - | - |
| ***PSRC1*** | - | - | - | - | 1.71 | - |
| ***PTCH1*** | - | - | - | - | - | -1.74 |
| ***PTEN*** | - | - | - | - | - | 1.63 |
| ***PTK2*** | -1.72 | - | - | - | - | -1.70 |
| ***PTPN11*** | -1.91 | - | - | - | - | - |
| ***PTTG1*** | - | - | - | - | - | -2.40 |
| ***RAB32*** | - | - | - | - | - | 1.63 |
| ***RABGGTB*** | - | - | 1.74 | - | - | - |
| ***RACGAP1*** | - | - | - | - | - | -2.55 |
| ***RAD18*** | - | - | - | - | 2.84 | - |
| ***RAD51*** | - | - | - | - | 2.38 | -3.52 |
| ***RAD51AP1*** | - | - | - | - | - | -3.20 |
| ***RAD51C*** | - | - | - | - | 1.87 | 1.81 |
| ***RANBP2*** | -1.81 | - | - | - | - | - |
| ***RAP1A*** | - | - | 109.60 | - | 164.92 | 176.00 |
| ***RBBP4*** | -1.77 | - | - | - | - | - |
| ***RCAN1*** | - | - | - | - | 1.83 | 1.97 |
| ***REXO2*** | - | - | - | - | - | 1.65 |
| ***RFC3*** | -2.40 | - | - | - | 2.76 | - |
| ***RFK*** | - | - | - | - | - | 1.74 |
| ***RGS6*** | 1.84 | - | - | - | - | - |
| ***RHOA*** | -2.04 | - | - | - | - | - |
| ***RHOB*** | -1.71 | - | - | - | - | - |
| ***RNMT*** | - | - | - | - | - | 2.27 |
| ***RPA3*** | - | - | - | - | 1.86 | - |
| ***RPL23A*** | - | - | - | -1.66 | - | - |
| ***RRAS2*** | - | - | - | - | - | 1.67 |
| ***RRM1*** | - | - | - | - | - | -1.65 |
| ***RRM2*** | - | - | - | - | - | -5.97 |
| ***RRM2B*** | - | - | - | - | 1.74 | 1.73 |
| ***RTKN2*** | - | - | - | - | - | -3.32 |
| ***S100A11*** | - | - | - | - | 3.37 | 3.54 |
| ***S100B*** | - | - | - | - | - | 1.82 |
| ***S1PR3*** | - | - | - | - | 3.35 | - |
| ***SASS6*** | - | - | - | - | 1.87 | - |
| ***SCG2*** | - | - | - | - | - | 3.22 |
| ***SCN2A*** | - | - | - | - | 2.44 | 3.11 |
| ***SEPT6*** | -1.82 | - | - | - | -1.69 | - |
| ***SERBP1*** | - | - | 1.82 | - | - | - |
| ***SERPINE2*** | -1.81 | - | - | -1.74 | - | - |
| ***SERPINI1*** | - | - | - | - | 2.27 | - |
| ***SERTAD1*** | - | - | - | - | - | 2.23 |
| ***SESN1*** | - | - | - | - | 1.66 | - |
| ***SETD8*** | -1.76 | - | - | - | - | - |
| ***SFR1*** | -2.43 | - | - | - | - | - |
| ***SGCB*** | - | - | - | - | - | 1.66 |
| ***SGOL1*** | - | - | - | - | 2.36 | - |
| ***SGOL2*** | - | - | - | - | 2.26 | -2.30 |
| ***SGPP1*** | -1.86 | - | - | - | - | - |
| ***SIAE*** | - | - | - | - | 1.90 | 1.66 |
| ***SIN3A*** | - | - | - | -1.74 | - | - |
| ***SIN3B*** | - | - | - | 1.90 | - | - |
| ***SKA1*** | - | - | - | - | 2.57 | - |
| ***SKA3*** | - | - | - | - | 2.09 | - |
| ***SKAP2*** | - | - | - | - | - | 1.82 |
| ***SLC12A2*** | - | - | - | - | - | 1.98 |
| ***SLC30A1*** | - | - | - | - | - | 2.43 |
| ***SLC30A9*** | - | - | - | - | - | 1.67 |
| ***SLC31A1*** | - | - | - | - | 1.64 | - |
| ***SLC3A2*** | - | - | - | - | - | 1.72 |
| ***SLC47A1*** | - | - | - | - | - | 2.11 |
| ***SMAD4*** | - | - | - | - | -2.08 | -2.03 |
| ***SMAD6*** | - | - | 1.67 | - | - | - |
| ***SMC2*** | - | - | - | - | 3.11 | -2.36 |
| ***SMC4*** | - | - | - | - | - | -1.63 |
| ***SMYD2*** | - | - | - | - | -1.73 | -1.80 |
| ***SNRK*** | - | - | - | - | - | -1.71 |
| ***SNRPA1*** | - | - | - | - | -1.86 | - |
| ***SNRPN*** | -1.67 | - | - | - | -1.84 | -1.73 |
| ***SORT1*** | - | - | - | - | - | 1.78 |
| ***SOX11*** | - | - | - | - | -1.80 | - |
| ***SOX6*** | - | - | - | - | - | 1.89 |
| ***SP3*** | - | - | - | - | -1.71 | - |
| ***SPC25*** | - | - | - | - | 2.54 | -3.26 |
| ***SPRY2*** | -1.71 | - | - | - | - | 1.75 |
| ***SPTAN1*** | - | - | - | 2.28 | - | - |
| ***SQSTM1*** | - | - | - | - | - | 1.85 |
| ***SRA1*** | - | - | - | - | - | 1.66 |
| ***SRPX*** | - | - | - | - | 2.07 | 2.13 |
| ***SRXN1*** | - | - | - | - | - | 1.75 |
| ***SSBP2*** | - | - | - | - | - | -1.74 |
| ***SSR1*** | - | - | - | - | - | 1.66 |
| ***ST7L*** | - | - | - | - | - | 1.63 |
| ***ST8SIA1*** | -2.70 | - | - | - | - | 2.13 |
| ***ST8SIA4*** | - | - | - | - | -2.79 | - |
| ***STAM2*** | - | - | - | - | - | 2.52 |
| ***STAT1*** | - | - | - | - | - | 1.72 |
| ***STAT3*** | -1.64 | - | - | - | - | - |
| ***STC1*** | - | - | - | - | -2.16 | - |
| ***STEAP3*** | - | - | - | - | 2.17 | 2.15 |
| ***STIL*** | - | - | - | - | - | -2.69 |
| ***SULF2*** | - | - | - | - | 2.74 | 3.56 |
| ***SUN2*** | - | - | - | - | -1.80 | - |
| ***SUV39H1*** | - | - | - | - | 1.85 | - |
| ***SUV420H1*** | - | - | - | - | -1.71 | - |
| ***SWAP70*** | - | - | - | - | - | 1.63 |
| ***TAF4B*** | - | - | - | - | 1.67 | - |
| ***TAP1*** | - | - | - | - | - | 2.15 |
| ***TCF4*** | - | - | - | - | -1.91 | - |
| ***TCF7L2*** | -1.74 | - | - | - | - | - |
| ***TGFB2*** | - | - | - | - | - | 1.86 |
| ***TGFBR3*** | -1.92 | - | - | - | - | - |
| ***TIA1*** | - | - | - | - | -1.69 | -1.77 |
| ***TIAM1*** | - | - | - | - | -1.64 | - |
| ***TIMELESS*** | - | - | - | - | 1.63 | - |
| ***TIMP1*** | - | - | - | 1.68 | 1.73 | 1.84 |
| ***TIMP2*** | - | - | - | - | - | 1.85 |
| ***TLR3*** | - | - | - | - | 2.41 | 2.30 |
| ***TLR4*** | - | - | - | - | 2.26 | 1.60 |
| ***TM2D1*** | - | - | - | - | - | -1.92 |
| ***TMEFF2*** | - | - | - | - | - | -1.82 |
| ***TMEM48*** | - | - | - | - | 3.26 | - |
| ***TMPO*** | - | - | - | - | - | -1.77 |
| ***TNFAIP3*** | - | - | 2.72 | - | - | - |
| ***TNFRSF10A*** | - | - | - | - | 1.93 | 2.44 |
| ***TNFRSF10B*** | - | - | - | - | - | 2.46 |
| ***TNFRSF10D*** | - | - | - | - | - | 2.45 |
| ***TNFRSF11B*** | - | - | - | - | 1.99 | 2.22 |
| ***TNFRSF1A*** | - | - | - | - | - | 1.71 |
| ***TNIK*** | - | - | - | - | - | 1.66 |
| ***TOP2A*** | - | - | - | - | - | -6.78 |
| ***TP53*** | - | - | - | 1.81 | - | 1.78 |
| ***TPM2*** | - | - | - | - | 2.08 | - |
| ***TPR*** | -1.72 | - | - | -2.04 | - | - |
| ***TRAF4*** | -1.85 | - | - | - | - | - |
| ***TRIAP1*** | - | - | - | - | - | 1.80 |
| ***TRIB3*** | - | - | - | - | - | 2.00 |
| ***TRIM13*** | - | - | - | - | -2.34 | - |
| ***TRIM2*** | - | - | - | - | 2.60 | - |
| ***TRIM24*** | - | - | - | -1.65 | - | -1.69 |
| ***TRIM28*** | - | - | - | 1.79 | - | - |
| ***TRIO*** | -1.65 | - | - | - | - | - |
| ***TRIP13*** | - | - | - | - | 5.51 | - |
| ***TRIP6*** | - | - | - | - | 1.69 | - |
| ***TRO*** | - | - | - | - | -1.90 | - |
| ***TRPM8*** | - | - | - | - | 2.60 | 2.05 |
| ***TRPS1*** | -2.08 | - | - | - | - | - |
| ***TSC22D1*** | -2.55 | - | - | - | - | - |
| ***TSGA10*** | - | - | 2.14 | - | - | - |
| ***TSIX*** | - | - | - | - | -2.17 | - |
| ***UBA1*** | - | - | - | 2.35 | - | - |
| ***UBE2C*** | - | - | - | - | - | -4.92 |
| ***UBE2T*** | - | - | - | - | - | -3.20 |
| ***UBQLN1*** | - | - | - | - | - | 1.65 |
| ***UHMK1*** | -1.68 | - | - | - | - | - |
| ***UHRF1*** | - | - | - | - | - | -2.91 |
| ***USP1*** | -1.68 | - | - | - | - | -1.73 |
| ***USP21*** | - | - | - | 1.79 | - | - |
| ***USP3*** | - | - | - | - | - | -1.71 |
| ***USP36*** | - | - | - | - | - | 1.72 |
| ***VASH1*** | - | - | - | - | -1.70 | - |
| ***VASH2*** | - | - | - | - | -2.87 | - |
| ***VMP1*** | - | - | - | - | - | 2.10 |
| ***WASF2*** | -1.71 | - | - | - | - | - |
| ***WBP2*** | - | - | - | 1.73 | - | - |
| ***WDR12*** | - | - | - | - | 1.64 | - |
| ***XBP1*** | - | - | - | - | 1.81 | 1.88 |
| ***XIAP*** | - | - | - | - | - | 1.87 |
| ***XPO1*** | -2.44 | - | - | - | - | - |
| ***XRCC4*** | - | - | - | - | 2.11 | - |
| ***XRN1*** | - | - | - | - | - | 1.94 |
| ***ZEB2*** | - | - | - | - | -2.03 | - |
| ***ZFHX3*** | - | - | - | 1.97 | - | - |
| ***ZFYVE16*** | - | - | - | - | -1.63 | - |
| ***ZNF346*** | 1.66 | - | - | - | - | - |
| ***ZNF365*** | - | - | - | - | - | 1.67 |
| ***ZWILCH*** | -1.91 | - | - | - | 1.98 | - |
| ***ZWINT*** | - | - | - | - | - | -3.64 |
| ***ZYX*** | - | - | - | 2.12 | - | - |

b) immune response

|  | **2h** | | | **5d** | | |
| --- | --- | --- | --- | --- | --- | --- |
| ***genes*** | **HD-HAd** | **HD-CAV-2** | **LV** | **HD-HAd** | **HD-CAV-2** | **LV** |
| ***ACVR2A*** | - | - | - | - | - | -1.86 |
| ***ADORA2A*** | 1.70 | - | - | - | - | - |
| ***ANXA1*** | - | - | - | - | - | 2.66 |
| ***ANXA2*** | - | - | - | - | - | 2.99 |
| ***ANXA4*** | - | - | - | - | 2.06 | 2.30 |
| ***APC*** | -2.26 | - | - | - | - | - |
| ***ARNTL*** | -1.83 | - | - | - | - | - |
| ***ATF3*** | - | - | - | - | - | 6.59 |
| ***AURKA*** | - | - | - | - | - | -2.95 |
| ***BCL10*** | -1.96 | - | - | - | - | - |
| ***BCL11B*** | - | - | - | - | -4.73 | - |
| ***BIRC5*** | - | - | - | - | 2.13 | -10.00 |
| ***BLM*** | - | - | - | - | 1.97 | - |
| ***BRIP1*** | - | - | - | - | - | -4.71 |
| ***BUB1*** | - | - | - | - | - | -3.24 |
| ***C12orf5*** | - | - | - | - | 1.85 | - |
| ***CASP1*** | - | - | - | - | 1.81 | 1.85 |
| ***CBFB*** | - | - | - | - | - | 1.79 |
| ***CCNB1*** | - | - | - | - | - | -3.64 |
| ***CCNB2*** | - | - | - | - | - | -5.18 |
| ***CCND1*** | - | - | - | - | - | 2.71 |
| ***CD44*** | - | - | - | - | 1.85 | 2.55 |
| ***CD58*** | - | - | - | - | 1.78 | 1.64 |
| ***CDC25A*** | - | - | - | - | 1.69 | -1.85 |
| ***CDK1*** | - | - | - | - | - | -5.46 |
| ***CDKN1A*** | - | - | - | 2.03 | 2.45 | 3.29 |
| ***CDKN2C*** | - | - | - | - | - | -1.72 |
| ***CEBPB*** | - | - | - | - | - | 2.89 |
| ***CHEK1*** | - | - | - | - | 2.26 | -2.04 |
| ***CHRNA7*** | - | - | - | - | - | 1.65 |
| ***CHST3*** | - | - | - | - | 1.75 | 1.68 |
| ***COL4A1*** | - | - | - | - | - | 2.90 |
| ***CREBBP*** | - | - | - | - | - | -1.71 |
| ***CX3CL1*** | - | - | - | - | 1.77 | 1.99 |
| ***DDB2*** | - | - | - | - | - | 1.68 |
| ***DDIT3*** | - | - | - | - | - | 2.31 |
| ***DICER1*** | - | - | - | - | -1.80 | - |
| ***DLEU2*** | -2.58 | - | - | - | - | -2.85 |
| ***DUSP3*** | - | - | - | - | 1.82 | 2.32 |
| ***EGR1*** | - | - | - | - | - | 4.71 |
| ***EPHA2*** | - | - | - | 1.94 | 2.42 | 2.85 |
| ***EPHA4*** | - | - | - | - | - | -2.33 |
| ***EZH2*** | - | - | - | - | - | -1.79 |
| ***FABP5*** | - | - | - | - | - | 1.84 |
| ***FANCD2*** | - | - | - | - | 2.37 | -5.92 |
| ***FAS*** | - | - | - | - | 4.25 | 3.76 |
| ***FGFR1*** | - | - | - | - | - | 1.66 |
| ***FHIT*** | - | - | - | - | - | 1.64 |
| ***G6PC3*** | - | - | - | - | - | 1.64 |
| ***GADD45A*** | - | - | - | - | - | 3.93 |
| ***GDF15*** | - | - | - | - | 4.33 | 10.40 |
| ***H2AFX*** | - | - | - | - | - | -1.77 |
| ***HAS3*** |  |  |  |  | 1.95 | 2.08 |
| ***HBB*** | - | - | 1.67 | - | - | - |
| ***HEXB*** | - | - | - | - | 1.64 | 2.07 |
| ***HIP1*** | -1.75 | - | - | - | - | - |
| ***HLA-A*** | - | - | - | - | - | 1.66 |
| ***HLA-C*** | - | - | - | - | - | 2.11 |
| ***HMGB1*** | - | - | - | - | - | -1.85 |
| ***HRAS*** | - | - | - | - | - | 1.64 |
| ***HSP90AA1*** | -1.95 | - | - | - | - | - |
| ***ID1*** | 14.10 | - | 12.10 | - | - | - |
| ***ID2*** | 2.30 | - | 2.13 | - | - | - |
| ***ID3*** | 15.30 | - | 7.56 | - | - | - |
| ***IFI35*** | - | - | - | - | - | 2.17 |
| ***IFI6*** | - | - | - | - | - | 2.13 |
| ***IFITM1*** | - | - | - | - | - | 2.33 |
| ***IFITM2*** | - | - | - | - | - | 2.55 |
| ***IFITM3*** | - | - | - | - | - | 2.87 |
| ***IKBIP*** | - | - | - | - | 2.68 | - |
| ***IL13RA1*** | - | - | - | - | - | 1.66 |
| ***ISG15*** | - | - | - | - | - | 2.35 |
| ***ITGA2*** | - | - | - | - | 1.72 | - |
| ***ITGB8*** | -2.15 | - | - | - | - | 2.07 |
| ***JAG1*** | -1.90 | - | - | - | - | - |
| ***KIAA0101*** | - | - | - | - | - | -5.17 |
| ***KIF11*** | - | - | - | - | - | -4.68 |
| ***KLF3*** | - | - | - | - | - | 1.88 |
| ***LEPR*** | 2.05 | - | - | - | - | 1.80 |
| ***LGALS3*** | - | - | - | - | - | 2.09 |
| ***LGALS8*** | - | - | - | - | - | 1.68 |
| ***LYST*** | - | - | - | - | - | 1.72 |
| ***MAD2L1*** | - | - | - | - | 2.68 | -3.33 |
| ***MAP4K4*** | - | - | - | - | -2.17 | -1.82 |
| ***MAPK1*** | - | - | - | - | - | 2.18 |
| ***MAPK8*** | - | - | - | - | -2.14 | - |
| ***MBTD1*** | - | - | - | - | -1.86 | -1.65 |
| ***MDM2*** | 1.77 | - | - | - | 2.04 | 3.56 |
| ***MERTK*** | - | - | - | - | - | 1.72 |
| ***MLL*** | -1.76 | - | - | - | - | - |
| ***MLL3*** | -1.85 | - | - | - | -1.69 | - |
| ***MLLT10*** | -1.84 | - | - | - | - | - |
| ***MYBL1*** | - | - | - | - | 2.21 | -2.40 |
| ***MYC*** | -2.02 | - | - | - | - | - |
| ***NBN*** | - | - | - | - | - | 1.83 |
| ***NCAM1*** | - | - | - | - | -2.22 | -1.86 |
| ***NEDD9*** | - | - | - | - | - | 1.77 |
| ***NEIL3*** | - | - | - | - | 2.76 | -2.21 |
| ***NEXN*** | -1.69 | - | - | - | 2.12 | 1.71 |
| ***NFKBIA*** | - | - | 1.97 | - | - | - |
| ***NFYA*** | -1.63 | - | - | - | - | - |
| ***NLRC5*** | - | - | - | - | - | 2.03 |
| ***NME1*** | - | - | - | - | 1.78 | - |
| ***NOG*** | 3.19 | - | - | - | - | - |
| ***NQO1*** | - | - | - | - | - | 2.80 |
| ***NRAS*** | - | - | - | - | - | 1.92 |
| ***OSMR*** | - | - | - | - | 1.71 | 2.14 |
| ***PALLD*** | -1.80 | - | - | - | - | - |
| ***PCGF2*** | - | - | - | - | - | -1.65 |
| ***PFN1*** | - | - | - | 2.56 | 2.10 | - |
| ***PIK3CA*** | - | - | - | - | -1.75 | -1.74 |
| ***PIK3R1*** | - | - | - | -1.66 | - | - |
| ***PLSCR1*** | - | - | - | - | - | 2.02 |
| ***PNP*** | - | - | - | - | 1.80 | - |
| ***PRDX6*** | - | - | - | - | 1.64 | - |
| ***PRIM1*** | - | - | - | - | - | -1.74 |
| ***PRKAA1*** | - | - | - | - | - | 2.18 |
| ***PTEN*** | - | - | - | - | - | 1.63 |
| ***PTK2*** | -1.72 | - | - | - | - | -1.70 |
| ***PTPN11*** | -1.91 | - | - | - | - | - |
| ***PTTG1*** | - | - | - | - | - | -2.40 |
| ***RABEP1*** | - | - | - | - | - | 1.72 |
| ***RAD51*** | - | - | - | - | 2.38 | -3.52 |
| ***RAD51C*** | - | - | - | - | 1.87 | 1.81 |
| ***RAP1A*** | - | - | 109.60 | - | 164.92 | 176.00 |
| ***RHOA*** | -2.04 | - | - | - | - | - |
| ***RHOB*** | -1.71 | - | - | - | - | - |
| ***RRM1*** | - | - | - | - | - | -1.65 |
| ***RRM2*** | - | - | - | - | - | -5.97 |
| ***RRM2B*** | - | - | - | - | 1.74 | 1.73 |
| ***SIAE*** | - | - | - | - | 1.90 | 1.66 |
| ***SMAD4*** | - | - | - | - | -2.08 | -2.03 |
| ***SMAD6*** | - | - | 1.67 | - | - | - |
| ***SPEN*** | -1.65 | - | - | - | - | - |
| ***SRPX*** | - | - | - | - | 2.07 | 2.13 |
| ***SSBP2*** | - | - | - | - | - | -1.74 |
| ***ST8SIA1*** | -2.70 | - | - | - | - | 2.13 |
| ***STAM2*** | - | - | - | - | - | 2.52 |
| ***STAT1*** | - | - | - | - | - | 1.72 |
| ***STAT3*** | -1.64 | - | - | - | - | - |
| ***STEAP3*** | - | - | - | - | 2.17 | 2.15 |
| ***SWAP70*** | - | - | - | - | - | 1.64 |
| ***TAP1*** | - | - | - | - | - | 2.15 |
| ***TIA1*** | - | - | - | - | -1.69 | -1.77 |
| ***TIMP1*** | - | - | - | 1.68 | 1.73 | 1.84 |
| ***TIMP2*** | - | - | - | - | - | 1.85 |
| ***TLR3*** | - | - | - | - | 2.41 | 2.30 |
| ***TLR4*** | - | - | - | - | 2.26 | 1.60 |
| ***TMPO*** | - | - | - | - | - | -1.77 |
| ***TNFAIP3*** | - | - | 2.72 | - | - | - |
| ***TNFRSF10A*** | - | - | - | - | 1.93 | 2.44 |
| ***TNFRSF10B*** | - | - | - | - | - | 2.46 |
| ***TNFRSF11B*** | - | - | - | - | 1.99 | 2.22 |
| ***TNFRSF1A*** | - | - | - | - | - | 1.71 |
| ***TNIK*** | - | - | - | - | - | 1.66 |
| ***TOP2A*** | - | - | - | - | - | -6.78 |
| ***TP53*** | - | - | - | 1.81 | - | 1.78 |
| ***WASF2*** | -1.71 | - | - | - | - | - |
| ***WWP1*** | -1.76 | - | - | - | - | - |
| ***XBP1*** | - | - | - | - | 1.81 | 1.88 |
| ***XIAP*** | - | - | - | - | - | 1.87 |

c) trafficking and remodeling.

|  | **2h** | | | **5d** | | |
| --- | --- | --- | --- | --- | --- | --- |
| ***genes*** | **HD-HAd** | **HD-CAV-2** | **LV** | **HD-HAd** | **HD-CAV-2** | **LV** |
| ***ABCD3*** | -2.38 | - | - | -2.10 | - | - |
| ***ACTN1*** | - | - | - | 1.76 | 1.98 | 1.72 |
| ***ACTN4*** | - | - | - | 1.86 | - | - |
| ***ACVR2A*** | - | - | - | - | - | -1.86 |
| ***ADRB1*** | -1.93 | - | - | - | - | - |
| ***AGRN*** | - | - | - | 1.64 | - | - |
| ***AJUBA*** | - | - | - | - | 1.70 | 1.80 |
| ***AKT1*** | - | - | - | 1.72 | - | - |
| ***AKT1S1*** | - | - | - | 1.73 | - | - |
| ***AKT3*** | - | - | - | - | -1.77 | - |
| ***ALS2*** | -2.14 | - | - | - | - | - |
| ***ANXA1*** | - | - | - | - | - | 2.66 |
| ***ANXA2*** | - | - | - | - | - | 2.99 |
| ***AP2M1*** | - | - | - | 1.78 | - | - |
| ***AP3B2*** | - | - | - | 1.63 | - | - |
| ***APC*** | -2.26 | - | - | - | - | - |
| ***AQP4*** | -1.79 | - | - | - | - | - |
| ***AR*** | - | - | - | 1.96 | - | - |
| ***ARHGAP29*** | -3.40 | - | - | - | - | 3.21 |
| ***ARHGEF2*** | - | - | - | - | - | -1.75 |
| ***ARHGEF7*** | 2.59 | - | - | - | - | - |
| ***ARID1A*** | - | - | - | 2.31 | - | - |
| ***ARL1*** | - | - | - | - | - | 1.80 |
| ***ARL8B*** | - | - | - | - | - | 1.73 |
| ***ARNTL*** | -1.83 | - | - | - | - | - |
| ***ASAH1*** | -1.78 | - | - | - | - | - |
| ***ASAP1*** | -1.72 | - | - | - | - | - |
| ***ATAD2*** | - | - | - | - | - | -2.64 |
| ***ATF3*** | - | - | - | - | - | 6.59 |
| ***ATL3*** | - | - | - | - | - | 1.88 |
| ***ATP8A1*** | - | - | - | - | -2.57 | - |
| ***AURKA*** | - | - | - | - | - | -2.95 |
| ***BAG4*** | - | - | - | - | - | 1.93 |
| ***BAIAP2*** | - | - | - | - | - | 2.14 |
| ***BANF1*** | - | - | - | 1.99 | - | - |
| ***BCL10*** | -1.96 | - | - | - | - | - |
| ***BCL11B*** | - | - | - | - | -4.73 | - |
| ***BIRC5*** | - | - | - | - | 2.13 | -10.00 |
| ***BLM*** | - | - | - | - | 1.97 | - |
| ***BMP2K*** | -1.79 | - | - | - | - | - |
| ***BRAF*** | -1.97 | - | - | - | - | - |
| ***BRCA2*** | - | - | - | - | 3.25 | - |
| ***BRD7*** | - | - | - | -1.76 | - | - |
| ***BUB1*** | - | - | - | - | - | -3.20 |
| ***C12orf5*** | - | - | - | - | 1.85 | - |
| ***CALR*** | - | - | - | 1.76 | - | - |
| ***CAP2*** | -1.78 | - | - | - | - | - |
| ***CAPNS1*** | - | - | - | 1.64 | - | - |
| ***CASP1*** | - | - | - | - | 1.81 | 1.85 |
| ***CAST*** | - | - | - | - | - | 1.98 |
| ***CBFB*** | - | - | - | - | - | 1.79 |
| ***CBX5*** | - | - | - | - | -1.94 | -2.06 |
| ***CCNA2*** | - | - | - | - | - | -2.45 |
| ***CCNB1*** | - | - | - | - | - | -3.52 |
| ***CCNB2*** | - | - | - | - | - | -5.18 |
| ***CCND1*** | - | - | - | - | - | 2.71 |
| ***CCNE1*** | - | - | - | - | 1.88 | - |
| ***CCNE2*** | - | - | - | - | - | -2.83 |
| ***CD164*** | - | - | - | - | - | 2.88 |
| ***CD44*** | - | - | - | - | 1.85 | 2.78 |
| ***CDC14B*** | - | - | - | - | - | 2.02 |
| ***CDC25A*** | - | - | - | - | 1.69 | -1.85 |
| ***CDC25B*** | - | - | - | - | 2.32 | - |
| ***CDC25C*** | - | - | - | - | 2.62 | -2.32 |
| ***CDCA5*** | - | - | - | - | 2.42 | -2.77 |
| ***CDK1*** | - | - | - | - | - | -5.46 |
| ***CDK16*** | - | - | - | 2.07 | - | - |
| ***CDK2AP2*** | - | - | - | - | 1.94 | - |
| ***CDKN1A*** | - | - | - | 2.03 | 2.45 | 3.29 |
| ***CDKN1C*** | -2.12 | - | - | - | -2.31 | - |
| ***CDT1*** | - | - | - | - | 1.71 | -2.71 |
| ***CEBPB*** | - | - | - | - | - | 2.89 |
| ***CENPA*** | - | - | - | - | - | -4.29 |
| ***CENPF*** | - | - | - | - | 2.21 | -3.80 |
| ***CENPH*** | - | - | - | - | 2.40 | -1.90 |
| ***CENPJ*** | - | - | - | - | - | -1.77 |
| ***CENPW*** | - | - | - | - | 2.88 | -1.73 |
| ***CEP135*** | - | - | - | - | 2.08 | - |
| ***CEP70*** | 1.65 | - | - | - | - | - |
| ***CEP72*** | - | - | - | - | 2.05 | - |
| ***CES2*** | - | - | - | - | - | 1.72 |
| ***CFL1*** | - | - | - | 1.99 | - | - |
| ***CHAF1A*** | - | - | - | - | 1.80 | - |
| ***CHDH*** | -2.14 | - | - | - | - | - |
| ***CHEK1*** | - | - | - | - | 2.26 | -2.04 |
| ***CHEK2*** | - | - | - | - | 3.62 | - |
| ***CISD2*** | - | - | - | - | - | -1.64 |
| ***CKS1B*** | - | - | - | - | 2.74 | -1.86 |
| ***CKS2*** | - | - | - | - | 2.21 | -2.35 |
| ***CLIP1*** | - | - | - | - | -1.83 | - |
| ***CLTC*** | -2.20 | - | - | - | - | - |
| ***CNR1*** | -2.10 | - | - | -2.20 | - | - |
| ***CNTN2*** | - | - | - | - | -1.80 | - |
| ***COL5A2*** | - | - | - | - | 2.16 | - |
| ***CPLX1*** | - | - | - | 3.44 | - | - |
| ***CREBBP*** | - | - | - | - | - | -1.71 |
| ***CRYAB*** | - | - | - | 1.94 | 2.00 | 2.21 |
| ***CSDA*** | - | - | - | - | 2.18 | 2.60 |
| ***CTSL2*** | - | - | - | - | 2.87 | - |
| ***CUX1*** | - | - | - | 2.01 | - | - |
| ***CX3CL1*** | - | - | - | - | 1.77 | 1.99 |
| ***CYBRD1*** | - | - | - | - | - | 1.72 |
| ***DBC1*** | - | - | - | - | -2.59 | - |
| ***DCTN1*** | - | - | - | 2.09 | - | - |
| ***DDB2*** | - | - | - | - | - | 1.68 |
| ***DDIT3*** | - | - | - | - | - | 2.31 |
| ***DDX17*** | -2.20 | - | - | - | -2.40 | - |
| ***DDX39B*** | - | - | - | 2.12 | - | - |
| ***DEGS1*** | -1.95 | - | - | - | - | - |
| ***DHCR7*** | -2.00 | - | - | - | - | - |
| ***DIAPH2*** | - | - | - | - | - | 1.66 |
| ***DICER1*** | - | - | - | - | -1.79 | - |
| ***DIO2*** | 3.75 | - | - | - | - | - |
| ***DLG1*** | -1.76 | - | - | - | - | - |
| ***DNM3*** | - | - | - | - | -1.70 | - |
| ***DOK3*** | 1.72 | - | - | - | - | - |
| ***DRAM1*** | - | - | - | - | 2.16 | 2.22 |
| ***DTL*** | - | - | - | - | - | -4.57 |
| ***DTNA*** | -2.32 | - | - | - | - | 2.01 |
| ***ECT2*** | - | - | - | - | 2.46 | -2.34 |
| ***EFEMP1*** | - | - | - | 2.16 | - | - |
| ***EFNA5*** | 2.33 | - | - | - | - | - |
| ***EGLN3*** | - | - | - | - | - | -1.92 |
| ***EGR1*** | - | - | - | - | - | 4.71 |
| ***EGR2*** | - | - | - | - | - | 4.87 |
| ***EHD2*** | - | - | - | 1.75 | - | 1.74 |
| ***EHD4*** | - | - | - | - | 2.50 | 1.80 |
| ***EHMT2*** | - | - | - | 1.77 | - | - |
| ***EIF1*** | - | - | - | - | -1.71 | - |
| ***EIF2C2*** | -1.95 | - | - | - | - | - |
| ***EIF5A2*** | - | - | - | - | - | 1.74 |
| ***ELAVL1*** | - | - | - | - | -1.64 | - |
| ***ENPP2*** | - | - | - | - | 2.86 | 2.10 |
| ***EPB41*** | - | - | - | - | - | -1.69 |
| ***EPB41L3*** | -1.94 | - | - | - | - | - |
| ***EPHA2*** | - | - | - | 1.94 | 2.42 | 2.85 |
| ***EPHA4*** | - | - | - | - | - | -2.30 |
| ***ERBB3*** | - | - | - | - | 1.77 | - |
| ***ERBB4*** | - | - | - | - | -1.83 | - |
| ***ERCC6L*** | - | - | - | - | 2.32 | - |
| ***ERF*** | - | - | - | - | 1.78 | 1.78 |
| ***ESCO2*** | - | - | - | - | 1.95 | - |
| ***EZH2*** | - | - | - | - | - | -1.79 |
| ***FABP5*** | - | - | - | - | - | 1.84 |
| ***FAH*** | - | - | - | - | 1.82 | 1.81 |
| ***FANCD2*** | - | - | - | - | 2.37 | -5.92 |
| ***FAS*** | - | - | - | - | 4.25 | 4.39 |
| ***FBXO5*** | - | - | - | - | - | -1.95 |
| ***FDFT1*** | -1.84 | - | - | - | - | - |
| ***FDXR*** | - | - | - | - | 2.49 | 3.48 |
| ***FERMT2*** | -1.86 | - | - | - | - | - |
| ***FGFR1*** | - | - | - | - | - | 1.66 |
| ***FHIT*** | - | - | - | - | - | 1.64 |
| ***FHL1*** | -1.92 | - | - | - | - | - |
| ***FHL1*** | -1.90 | - | - | - | - | - |
| ***FKBP4*** | - | - | - | 1.63 | 1.64 | - |
| ***FOLR1*** | 1.63 | - | - | 1.84 | - | - |
| ***FOXM1*** | - | - | - | - | 3.42 | - |
| ***FSCN1*** | - | - | - | 1.75 | - | - |
| ***FXN*** | - | - | - | - | 1.98 | 1.85 |
| ***FXYD1*** | - | - | - | 1.74 | - | - |
| ***GADD45A*** | - | - | - | - | - | 3.93 |
| ***GALNT2*** | - | - | - | - | - | - |
| ***GALNT2*** | - | - | - | - | - | 1.98 |
| ***GAP43*** | - | - | - | - | - | 3.25 |
| ***GAS6*** | - | - | - | 1.92 | - | - |
| ***GDF15*** | - | - | - | - | 4.33 | 10.40 |
| ***GMNN*** | - | - | - | - | 1.76 | - |
| ***GNAI2*** | - | - | - | 1.68 | - | - |
| ***GNAS*** | -1.85 | - | - | - | - | - |
| ***GPX1*** | - | - | - | - | 1.67 | 1.98 |
| ***GRB10*** | - | - | - | - | - | 1.75 |
| ***GRIA1*** | - | - | - | - | 1.71 | 1.87 |
| ***GSTM1*** | - | - | - | 1.68 | - | - |
| ***H2AFX*** | - | - | - | - | - | -1.77 |
| ***HAS3*** | - | - | - | - | 1.95 | 2.08 |
| ***HAUS6*** | - | - | - | - | 1.97 | - |
| ***HBB*** | - | - | 1.67 | - | - | - |
| ***HELLS*** | - | - | - | - | 1.83 | - |
| ***HEXB*** | - | - | - | - | 1.64 | 2.07 |
| ***HIP1*** | -1.75 | - | - | - | - | - |
| ***HLA-C*** | - | - | - | - | - | 2.11 |
| ***HMGB1*** | - | - | - | - | - | -1.85 |
| ***HPRT1*** | -1.91 | - | - | - | - | - |
| ***HRAS*** | - | - | - | - | - | 1.64 |
| ***HSP90AA1*** | -1.95 | - | - | - | - | - |
| ***ID1*** | 14.10 | - | 12.10 | - | - | - |
| ***ID2*** | 2.30 | - | 2.13 | - | - | - |
| ***ID3*** | 15.30 | - | 7.56 | - | - | - |
| ***IGFBP5*** | - | - | - | 1.97 | - | - |
| ***ILK*** | - | - | - | 1.80 | - | - |
| ***INSIG1*** | -3.04 | - | - | - | - | -1.79 |
| ***INSR*** | - | - | - | - | -1.65 | - |
| ***IQGAP1*** | -1.77 | - | - | - | - | - |
| ***IRS1*** | - | - | - | - | - | 1.69 |
| ***ITCH*** | - | - | - | - | - | 1.75 |
| ***ITGA2*** | - | - | - | - | 1.72 | - |
| ***ITGB8*** | -2.15 | - | - | - | - | 2.07 |
| ***JAG1*** | -1.90 | - | - | - | - | - |
| ***KCNIP2*** | 1.74 | - | - | 1.94 | - | - |
| ***KCNJ2*** | - | - | - | - | - | 2.12 |
| ***KCNMA1*** | 1.96 | - | - | - | - | - |
| ***KIAA0101*** | - | - | - | - | - | -5.17 |
| ***KIF11*** | - | - | - | - | - | -4.68 |
| ***KIF14*** | - | - | - | - | 2.76 | -3.50 |
| ***KIF18A*** | - | - | - | - | 2.58 | - |
| ***KIF20B*** | - | - | - | - | 1.93 | - |
| ***KIF22*** | - | - | - | - | 1.70 | - |
| ***KIF4A*** | - | - | - | - | - | -3.61 |
| ***KIN*** | - | - | - | - | - | -1.66 |
| ***KLF6*** | -1.69 | - | - | - | - | - |
| ***LATS2*** | -1.63 | - | - | - | - | - |
| ***LDLR*** | -2.39 | - | - | - | - | - |
| ***LEPR*** | 2.05 | - | - | - | - | 1.83 |
| ***LGALS3*** | - | - | - | - | - | 2.09 |
| ***LGALS8*** | - | - | - | - | - | 1.68 |
| ***LIG1*** | - | - | - | - | 2.03 | - |
| ***LIN9*** | - | - | - | - | 1.96 | - |
| ***LMNA*** | - | - | - | - | - | 2.07 |
| ***LPAR2*** | - | - | - | - | -2.02 | - |
| ***LPXN*** | -1.68 | - | - | - | - | - |
| ***LRP1*** | - | - | - | 1.82 | - | - |
| ***LRP8*** | - | - | - | - | -1.73 | - |
| ***LUM*** | - | - | - | - | 3.71 | - |
| ***LYST*** | - | - | - | - | - | 1.72 |
| ***MAD2L1*** | - | - | - | - | 2.68 | -3.33 |
| ***MAP1A*** | - | - | - | 1.82 | - | - |
| ***MAP1B*** | -2.07 | - | - | - | - | - |
| ***MAP4K4*** | - | - | - | - | -2.17 | -1.82 |
| ***MAP9*** | - | - | - | - | - | 2.23 |
| ***MAPK1*** | - | - | - | - | - | 2.18 |
| ***MAPK8*** | - | - | - | - | -2.14 | - |
| ***MAPKAPK3*** | - | - | - | - | 1.62 | 1.64 |
| ***MASTL*** | - | - | - | - | 2.48 | - |
| ***MBTD1*** | - | - | - | - | -1.86 | -1.65 |
| ***MCAM*** | - | - | - | - | 2.48 | - |
| ***MDK*** | - | - | - | 2.56 | - | - |
| ***MDM2*** | 1.77 | - | - | - | 2.04 | 3.56 |
| ***MERTK*** | - | - | - | - | - | 1.72 |
| ***MINA*** | - | - | - | - | 1.68 | - |
| ***MLL*** | -1.76 | - | - | - | - | - |
| ***MLLT10*** | -1.84 | - | - | - | - | - |
| ***MLLT4*** | - | - | - | - | -1.69 | - |
| ***MRE11A*** | - | - | - | - | 2.29 | - |
| ***MYBL1*** | - | - | - | - | 2.21 | -2.40 |
| ***MYC*** | -2.02 | - | - | - | - | - |
| ***MYO10*** | 2.09 | - | - | - | - | - |
| ***MYO5A*** | - | - | - | - | - | 1.70 |
| ***MYO6*** | -1.81 | - | - | - | - | - |
| ***MYOF*** | - | - | - | - | 2.67 | 5.51 |
| ***NAB1*** | -2.46 | - | - | - | - | - |
| ***NAMPT*** | - | - | - | - | - | 2.63 |
| ***NAV1*** | -2.20 | - | - | - | - | - |
| ***NBN*** | - | - | - | - | - | 1.83 |
| ***NCAM1*** | - | - | - | - | -2.22 | -1.86 |
| ***NCAPG*** | - | - | - | - | - | -3.50 |
| ***NCAPH*** | - | - | - | - | 2.31 | - |
| ***NDC80*** | - | - | - | - | - | -5.41 |
| ***NEDD9*** | - | - | - | - | - | 1.77 |
| ***NEFL*** | - | - | - | - | 6.04 | 13.37 |
| ***NEK2*** | - | - | - | - | - | -4.58 |
| ***NEXN*** | -1.69 | - | - | - | 2.12 | 1.71 |
| ***NF1*** | -1.69 | - | - | - | - | - |
| ***NFIB*** | -1.91 | - | - | - | - | - |
| ***NFKBIA*** | - | - | 1.97 | - | - | - |
| ***NFYA*** | -1.63 | - | - | - | - | - |
| ***NIPBL*** | - | - | - | - | -1.64 | - |
| ***NLK*** | -1.68 | - | - | - | - | - |
| ***NME1*** | - | - | - | - | 1.78 | - |
| ***NME2*** | - | - | - | - | 1.84 | - |
| ***NMI*** | -1.70 | - | - | - | - | 1.90 |
| ***NOG*** | 3.19 | - | - | - | - | - |
| ***NOV*** | - | - | - | - | 5.02 | 8.74 |
| ***NPHP1*** | - | - | - | - | 1.89 | - |
| ***NQO1*** | - | - | - | - | - | 2.44 |
| ***NR4A2*** | - | - | - | -1.73 | - | - |
| ***NRAS*** | - | - | - | - | - | 1.92 |
| ***NREP*** | - | - | - | - | -2.03 | - |
| ***NRG1*** | - | - | - | - | - | 3.33 |
| ***NRP2*** | - | - | - | - | - | 2.72 |
| ***NUAK2*** | - | - | - | 1.69 | - | 2.00 |
| ***NUF2*** | - | - | - | - | - | -5.97 |
| ***NUP107*** | - | - | - | - | 1.81 | - |
| ***NUP160*** | -1.71 | - | - | - | - | - |
| ***NUPR1*** | - | - | - | - | - | 2.27 |
| ***NUSAP1*** | - | - | - | - | - | -5.12 |
| ***OSMR*** | - | - | - | - | 1.71 | 1.86 |
| ***PAFAH1B1*** | - | - | - | - | - | 1.86 |
| ***PALLD*** | -1.80 | - | - | - | - | - |
| ***PBK*** | - | - | - | - | 3.20 | - |
| ***PCGF2*** | - | - | - | - | - | -1.65 |
| ***PDLIM4*** | - | - | - | - | 1.86 | 1.99 |
| ***PDLIM7*** | - | - | - | 2.04 | 1.98 | - |
| ***PFN1*** | - | - | - | 2.56 | 2.10 | - |
| ***PHIP*** | - | - | - | - | 1.76 | - |
| ***PIGA*** | -1.64 | - | - | - | - | - |
| ***PIK3CA*** | - | - | - | - | -1.75 | -1.74 |
| ***PIK3R1*** | - | - | - | -1.66 | - | - |
| ***PIP4K2A*** | 1.77 | - | - | -1.99 | -1.63 | - |
| ***PKD1*** | - | - | - | 1.64 | - | - |
| ***PKM2*** | - | - | - | 2.70 | - | - |
| ***PLD3*** | - | - | - | 2.21 | - | - |
| ***PLDN*** | -1.70 | - | - | - | - | - |
| ***PLEKHA1*** | -1.68 | - | - | - | - | - |
| ***PLEKHA8*** | - | - | - | -2.55 | - | - |
| ***PLK3*** | - | - | - | - | 1.71 | 2.04 |
| ***PLK4*** | - | - | - | - | 2.99 | -2.44 |
| ***PLSCR1*** | - | - | - | - | - | 2.02 |
| ***PMF1*** | - | - | - | 1.71 | 1.66 | - |
| ***PNN*** | - | - | - | -1.80 | - | - |
| ***PNP*** | - | - | - | - | 1.80 | - |
| ***PODXL2*** | - | - | - | 1.87 | - | - |
| ***POLH*** | - | - | - | - | 1.91 | 1.75 |
| ***PPME1*** | - | - | - | 1.71 | - | - |
| ***PRC1*** | - | - | - | - | 2.20 | -3.83 |
| ***PRDX6*** | - | - | - | - | 1.64 | - |
| ***PRKAA1*** | - | - | - | - | - | 2.18 |
| ***PRKAR1A*** | - | - | - | - | - | 1.74 |
| ***PRPF4B*** | -1.74 | - | - | - | - | - |
| ***PSRC1*** | - | - | - | - | 1.71 | - |
| ***PTCD2*** | - | - | - | - | - | 1.64 |
| ***PTCH1*** | - | - | - | - | - | -1.74 |
| ***PTEN*** | - | - | - | - | - | 1.63 |
| ***PTK2*** | -1.72 | - | - | - | - | -1.70 |
| ***PTMS*** | - | - | - | 2.02 | - | - |
| ***PTPN11*** | -1.91 | - | - | - | - | - |
| ***PTTG1*** | - | - | - | - | - | -2.40 |
| ***RAB27B*** | - | - | - | - | - | 1.72 |
| ***RAB2A*** | - | - | - | -1.74 | - | - |
| ***RAB38*** | - | - | - | - | 1.94 | 1.69 |
| ***RAB8B*** | - | - | - | - | - | 1.80 |
| ***RACGAP1*** | - | - | - | - | - | -2.55 |
| ***RAD18*** | - | - | - | - | 2.84 | - |
| ***RAD51*** | - | - | - | - | 2.38 | -3.52 |
| ***RANBP2*** | -1.81 | - | - | - | - | - |
| ***RAP1A*** | - | - | 109.60 | - | 164.92 | 176.00 |
| ***RAP2A*** | - | - | - | -1.72 | - | - |
| ***RBBP4*** | -1.77 | - | - | - | - | - |
| ***RCAN1*** | - | - | - | - | 1.83 | 1.97 |
| ***RDX*** | - | - | - | -1.70 | - | - |
| ***RGS6*** | 1.84 | - | - | - | - | - |
| ***RHOA*** | -2.04 | - | - | - | - | - |
| ***RHOB*** | -1.71 | - | - | - | - | - |
| ***RHOJ*** | -1.82 | - | - | -1.73 | - | - |
| ***RHOQ*** | -1.78 | - | - | - | 1.78 | 1.78 |
| ***RNF144A*** | - | - | - | - | -1.76 | - |
| ***RRAS2*** | - | - | - | - | - | 1.67 |
| ***RRM1*** | - | - | - | - | - | -1.65 |
| ***RRM2*** | - | - | - | - | - | -5.97 |
| ***RRM2B*** | - | - | - | - | 1.74 | 1.73 |
| ***S100B*** | - | - | - | - | - | 1.82 |
| ***S1PR3*** | - | - | - | - | 3.35 | - |
| ***SASS6*** | - | - | - | - | 1.87 | - |
| ***SCAMP1*** | -1.76 | - | - | - | - | - |
| ***SCNN1B*** | - | - | - | 2.20 | - | - |
| ***SEH1L*** | - | - | - | - | - | 1.80 |
| ***SEPN1*** | - | - | - | 2.11 | - | - |
| ***SEPT6*** | -1.82 | - | - | - | -1.69 | - |
| ***SERPINE2*** | -1.81 | - | - | -1.74 | - | - |
| ***SERTAD1*** | - | - | - | - | - | 2.23 |
| ***SESN2*** | - | - | - | - | 1.82 | 2.37 |
| ***SEZ6L*** | -2.52 | - | - | - | - | - |
| ***SGOL1*** | - | - | - | - | 2.36 | - |
| ***SGOL2*** | - | - | - | - | 2.26 | -2.30 |
| ***SGPP1*** | -1.86 | - | - | - | - | - |
| ***SIN3A*** | - | - | - | -1.74 | - | - |
| ***SIN3B*** | - | - | - | 1.90 | - | - |
| ***SKA1*** | - | - | - | - | 2.57 | - |
| ***SKA3*** | - | - | - | - | 2.09 | - |
| ***SLC16A3*** | - | - | - | 2.33 | - | - |
| ***SLC25A1*** | - | - | - | 1.67 | - | - |
| ***SLC30A1*** | - | - | - | - | - | 2.43 |
| ***SLC31A1*** | - | - | - | - | 1.64 | - |
| ***SLC3A2*** | - | - | - | - | - | 1.72 |
| ***SMAD4*** | - | - | - | - | -2.08 | -2.03 |
| ***SMAD6*** | - | - | 1.67 | - | - | - |
| ***SMC2*** | - | - | - | - | 3.11 | -2.36 |
| ***SMC4*** | - | - | - | - | - | -1.63 |
| ***SNX10*** | - | - | - | - | - | 2.79 |
| ***SORT1*** | - | - | - | - | - | 1.78 |
| ***SOX6*** | - | - | - | - | - | 1.89 |
| ***SP3*** | - | - | - | - | -1.71 | - |
| ***SPC25*** | - | - | - | - | 2.54 | -3.27 |
| ***SPEN*** | -1.65 | - | - | - | - | - |
| ***SPRY2*** | -1.71 | - | - | - | - | 1.75 |
| ***SPTAN1*** | - | - | - | 2.28 | - | - |
| ***SQSTM1*** | - | - | - | - | - | 1.85 |
| ***SRCIN1*** | 1.70 | - | - | - | - | - |
| ***SRSF7*** | - | - | - | - | - | - |
| ***SSBP2*** | - | - | - | - | - | -1.74 |
| ***ST8SIA1*** | -2.70 | - | - | - | - | 2.13 |
| ***STAT1*** | - | - | - | - | - | 1.72 |
| ***STAT3*** | -1.64 | - | - | - | - | - |
| ***STAU2*** | 1.71 | - | - | - | - | - |
| ***STC1*** | - | - | - | - | -2.16 | - |
| ***STEAP3*** | - | - | - | - | 2.17 | 2.15 |
| ***STRN4*** | - | - | - | 1.64 | - | - |
| ***SUN2*** | - | - | - | - | -1.80 | - |
| ***SUV39H1*** | - | - | - | - | 1.85 | - |
| ***SYN2*** | - | - | - | 2.11 | - | - |
| ***TAGLN*** | - | - | - | - | 2.54 | - |
| ***TAP1*** | - | - | - | - | - | 2.15 |
| ***TCF4*** | - | - | - | - | -1.91 | - |
| ***TCF7L2*** | -1.74 | - | - | - | - | - |
| ***TGFB2*** | - | - | - | - | - | 1.86 |
| ***TGFBR3*** | -1.92 | - | - | - | - | - |
| ***TIAM1*** | - | - | - | - | -1.64 | - |
| ***TIMELESS*** | - | - | - | - | 1.63 | - |
| ***TIMP1*** | - | - | - | 1.68 | 1.73 | 1.84 |
| ***TIMP2*** | - | - | - | - | - | - |
| ***TIMP2*** | - | - | - | - | - | 1.85 |
| ***TLK1*** | -2.36 | - | - | - | - | - |
| ***TLR4*** | - | - | - | - | 2.26 | 1.60 |
| ***TMEFF2*** | - | - | - | - | - | -1.82 |
| ***TMEM48*** | - | - | - | - | 3.26 | - |
| ***TMOD2*** | - | - | - | - | - | 1.85 |
| ***TNFAIP3*** | - | - | 2.72 | - | - | - |
| ***TNFRSF10A*** | - | - | - | - | 1.93 | 2.44 |
| ***TNFRSF10B*** | - | - | - | - | - | 2.46 |
| ***TNFRSF11B*** | - | - | - | - | 1.99 | 2.22 |
| ***TNFRSF1A*** | - | - | - | - | - | 1.71 |
| ***TNIK*** | - | - | - | - | - | 1.66 |
| ***TOP2A*** | - | - | - | - | - | -6.78 |
| ***TP53*** | - | - | - | 1.81 | - | 1.78 |
| ***TPM2*** | - | - | - | - | 2.08 | - |
| ***TPR*** | -1.72 | - | - | -2.04 | - | - |
| ***TRAF4*** | -1.85 | - | - | - | - | - |
| ***TRIB3*** | - | - | - | - | - | 2.00 |
| ***TRIM24*** | - | - | - | -1.65 | - | -1.69 |
| ***TRIO*** | -1.65 | - | - | - | - | - |
| ***TRIP6*** | - | - | - | - | 1.69 | - |
| ***TRPS1*** | -2.08 | - | - | - | - | - |
| ***TTYH3*** | - | - | - | 1.72 | - | - |
| ***TXN*** | - | - | - | -1.72 | - | - |
| ***UBA1*** | - | - | - | 2.35 | - | - |
| ***UBE2C*** | - | - | - | - | - | -4.92 |
| ***UHMK1*** | -1.68 | - | - | - | - | - |
| ***UHRF1*** | - | - | - | - | - | -2.91 |
| ***USP1*** | -1.68 | - | - | - | - | -1.73 |
| ***USP21*** | - | - | - | 1.79 | - | - |
| ***VAMP2*** | - | - | - | 1.93 | - | - |
| ***VANGL2*** | - | - | - | 1.63 | - | - |
| ***VMP1*** | - | - | - | - | - | 2.10 |
| ***WASF2*** | -1.71 | - | - | - | - | - |
| ***WBP2*** | - | - | - | 1.73 | - | - |
| ***WDR12*** | - | - | - | - | 1.64 | - |
| ***WWP1*** | -1.76 | - | - | - | - | - |
| ***XBP1*** | - | - | - | - | 1.81 | 1.88 |
| ***XIAP*** | - | - | - | - | - | 1.87 |
| ***XPO1*** | -2.44 | - | - | - | - | - |
| ***XRCC4*** | - | - | - | - | 1.96 | - |
| ***ZEB2*** | - | - | - | - | -2.03 | - |
| ***ZFHX3*** | - | - | - | 1.97 | - | - |
| ***ZWINT*** | - | - | - | - | - | -3.64 |
| ***ZYX*** | - | - | - | 2.12 | - | - |
